# Upgrading ketone synthesis direct from carboxylic acids and organohalides

**DOI:** 10.1038/s41467-020-17224-2

**Published:** 2020-07-03

**Authors:** Rehanguli Ruzi, Kai Liu, Chengjian Zhu, Jin Xie

**Affiliations:** 1 State Key Laboratory of Coordination Chemistry, Jiangsu Key Laboratory of Advanced Organic Materials, Chemistry and Biomedicine Innovation Center (ChemBIC), School of Chemistry and Chemical Engineering, Nanjing University, Nanjing, 210023 China; 2 College of Chemistry and Molecular Engineering, Zhengzhou University, Zhengzhou, 450001 China

**Keywords:** Catalytic mechanisms, Homogeneous catalysis, Synthetic chemistry methodology

## Abstract

The ketone functional group has a unique reactivity in organic chemistry and is associated with a number of useful reactions. Catalytic methods for ketone synthesis are continually being developed. Here, we report a photoredox, nickel and phosphoranyl radical synergistic cross-electrophile coupling of commercially available chemicals, aromatic acids and aryl/alkyl bromides. This allows for concise synthesis of highly functionalized ketones directly, without the preparation of activated carbonyl intermediates or organometallic compounds, and thus complements the conventional Weinreb ketone synthesis. Use of the appropriate photocatalyst, ligand amount and solvents can match the reaction rate required by any simple catalytic cycle. The practicality and synthetic robustness of the reaction are illustrated by the facile synthesis of complex ketones from readily available feedstock chemicals.

## Abstract (web summary)

Due to their abundance and importance in organic chemistry, development of methods for ketone synthesis is essential. Here, the authors report a photoredox, nickel and phosphoranyl radical synergistic cross-electrophile coupling of aromatic acids and aryl/alkyl bromides to directly synthesise ketones.

## Introduction

**Fig. 1:**
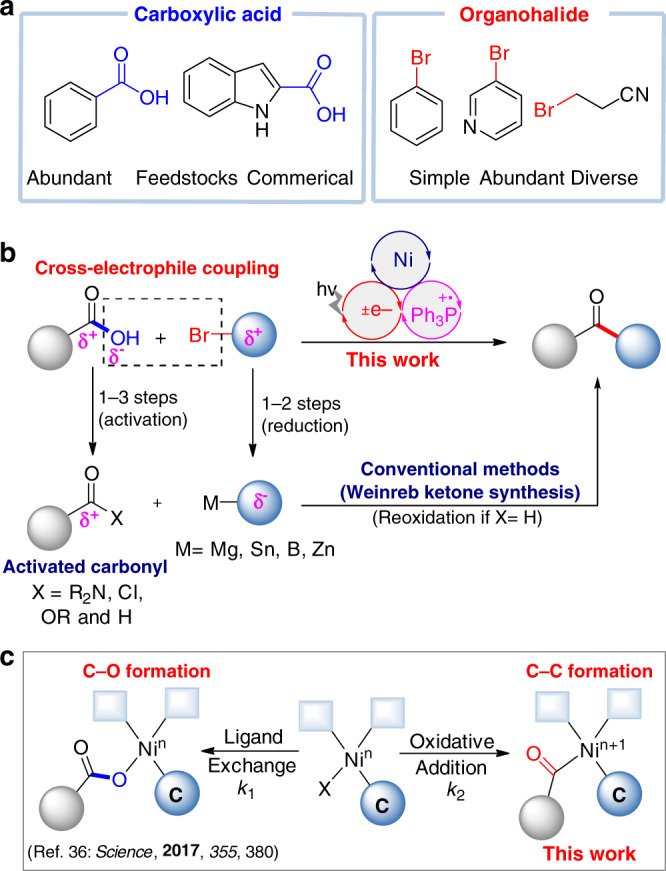
Catalytic cross-electrophile coupling between carboxylic acids and organohalides. **a** The abundant feedstock chemicals in synthetic lab. **b** Direct cross-electrophile coupling of acid and organohalides. **c** Key challenge: C–O versus C–C formation.

Ketones play a prominent role in organic chemistry. The ketone moiety is extremely common in natural products and pharmaceuticals^1^ and in dyes, fragrancies and flavors^2^. It is also a versatile reaction center in organic synthesis^3^. Many frequently used reactions, including the Mannich reaction, Wittig reaction, Grignard reaction, Passerini reaction, Baeyer–Villiger oxidation, and Wolff–Kishner–Huang reduction describe a wide array of transformations of ketones. The development of a practical route to ketones from feedstock chemicals has long been a subject of interest^4–12^. Carboxylic acids and organohalides are commercially abundant and structurally diverse, bench-stable feedstock chemicals commonly used in organic synthesis (Fig. 1a). When producing ketones from carboxylic acids and organohalides, the stoichiometric approach requires preparation of necessary intermediates such as amides or aldehydes and Grignard reagents^13,14^. If aldehydes are employed, reoxidation is necessary^15^. Catalytic strategies for production of ketones rely on transition metal-catalyzed carbon–carbon coupling between activated carbonyls such as acid chlorides or anhydrides with organometallic reagents (Fig. 1b)^16–19^. However, activated carbonyls are generally prepared in as many as three steps from carboxylic acids and organometallics are obtained typically by metalation of organohalides^20^, which can often lead to poor functional group compatibility or a lengthy functional group protection/deprotection process.

In recent years, nickel-catalyzed cross-electrophile coupling has attracted considerable attention^21–30^. The carbon–carbon coupling arises from two different electrophiles in the presence of stoichiometric reductant. We posited that a carboxylic acid could be directly used as a latent electrophile (C-terminus) rather than a nucleophile (O-terminus) in cross-coupling. This could simplify and upgrade ketone synthesis from carboxylic acids and organic halides^13^. Very recently, our group and Doyle et al. reported an elegant photoredox-promoted mild deoxygenation of carboxylic acids generating acyl radicals^31–35^. Since photoredox and nickel-catalyzed C–O bond formation between carboxylic acids and aromatic bromides has been reported^36^ to achieve the desired cross-electrophile coupling, acyl radical oxidative addition by a metallaphotoredox pathway^37–41^ is essential to suppress the C–O bond formation (Fig. 1c). Alternatively, a judicious strategy reported by Gong et al. is conversion of carboxylic acid into anhydrides in situ to suppress the C–O coupling^22,23^. However, the use of free carboxylic acids as acyl radical precursors is a challenge in the oxidative addition step as a result of the strong bond dissociation energy of the C–O bond (106 kcal mol^−1^)^33^.

**Fig. 2:**
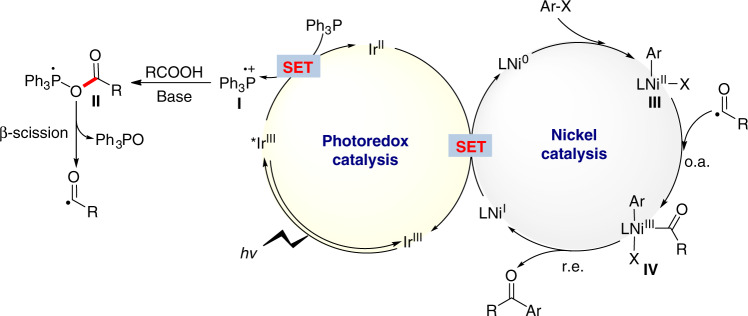
Proposed mechanism. Mechanistic proposal for cross-electrophile coupling of acid and aryl bromides. o.a. oxidative addition, r.e. reductive elimination.

A proposed mechanism for the designed metallaphotoredox cross-electrophile coupling is shown in Fig. 2. The photoexcited *[Ir(dF(CF_3_)ppy)_2_(dtbbpy)]PF_6_ [^1/2^*E*_red_ (*Ir^III^/Ir^II^) = +1.21 V vs SCE, *τ* = 2.3 μs]^38^ causes single-electron transfer (SET) oxidation of triphenylphosphine (*E*_red_ = +0.98V vs SCE)^31^, as indicated by our Stern–Volmer experimental results (see Supplementary Fig. 7). The triphenylphosphine radical cation (**I**) generated recombines with the carboxylate anion to form a phosphoranyl radical intermediate (**II**). Owing to the strong affinity between the phosphoranyl radical and oxygen, a facile β-scission of the radical species (**II**) occurs, giving rise to a nucleophilic acyl radical^42^, which can undergo oxidative addition to the resulting aryl-Ni^II^ species (**III**) giving the Ni^III^ species (**IV**)^43–45^. Finally, reductive elimination from the Ni^III^ intermediate (**IV**) can generate the desired cross-electrophile coupling product, R-CO-Ar. A second SET event from Ir^II^ ([Ir^III^/Ir^II^] = −1.37 V vs SCE), leading to an Ni^I^ species completes both catalytic cycles. The synergistic combination of photoredox with nickel catalysis proposed in Fig. 2 is a subject of continuing research^46–58^, and several challenges remain in this cross-electrophile coupling and must be addressed: (1) Efficient control of the matching of the C–O bond cleavage and the radical addition to the nickel center; (2) weakening of the interference of stoichiometric triphenylphosphine in the nickel catalytic unit; and (3) use of appropriate ligands and solvents to significantly suppress the C–O bond formation. Herein, we report a formal cross-electrophile coupling of carboxylic acids with aryl or alkyl halides enabled by photoredox and nickel catalysis, and phosphoranyl radical synergistic chemistry, leading to concise synthesis of ketones (Fig. 1b).

## Results

### Reaction optimization

**Fig. 3:**
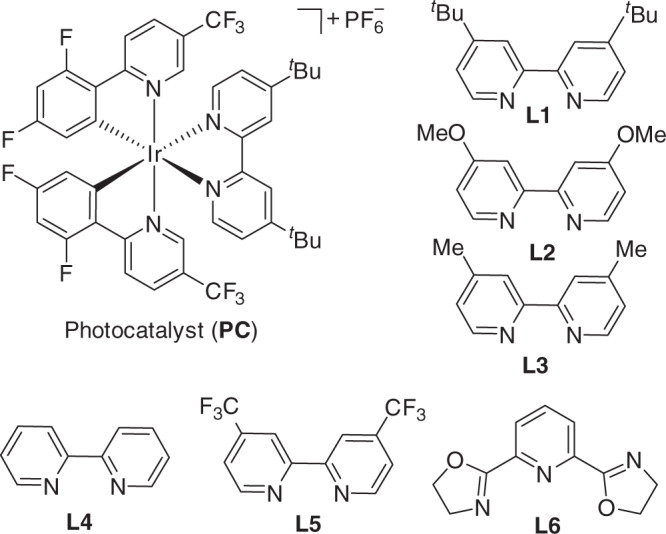
Photocatalysts and ligands. Catalysts and ligands for cross-electrophile coupling of acid and aryl bromides.

Our investigation of this cross-electrophile coupling began with the reaction of 4-methyl-benzoic acid (**1**) with 5-bromo-2-(trifluoromethyl) pyridine (**2**), and the representative results are presented in Table 1. The optimized reaction conditions include 2 mol% [Ir{dF(CF_3_)ppy}_2_{dtbbpy}]PF_6_, 3 mol% NiBr_2_.dme together with 5 mol% 4,4′-di-*tert*-butyl-2,2′-bipyridine (**L1**, Fig. 3) and 1.5 equiv Ph_3_P with a mixed DMF-CH_3_CN solvent (entry 1, Table 1). Under the standard conditions, the desired cross-electrophile coupling product (**3**) can be obtained in 82% yield while the yield of the C–O coupling process, giving **3**′ is suppressed to 16%. We found the loading amount of ligand plays an important role in the control of the C–C and C–O bond formation (entries 2–4, Table 1). An increased or decreased loading amount of 4,4′-di-*tert*-butyl-2,2’-bipyridine can facilitate the formation of the C–O coupling by-product (**3**′). Screening of different ligands and solvents indicated that both a ligand effect and a solvent effect are crucial for a successful cross-electrophile coupling (entries 5–11, Table 1). We speculated that only compatible consecutive steps with well-matched rates would benefit this cross-electrophile coupling. In the absence of either photocatalyst, NiBr_2_·dme, triphenylphosphine or light irradiation, the model reaction failed to occur. The quantum yield of the model reaction was determined to be 0.35, arguing against a radical chain pathway.

**Table 1:** Optimization of the reaction conditions.


Entry	Variation of standard conditions	Isolated yield: 3 (3′)
1	None	82% (16%)
2	10 mol% **L1**	46% (37%)
3	15 mol% **L1**	23% (53%)
4	3 mol% of **L1**	19% (10%)
5	5 mol% **L2**	31% (33%)
6	5 mol% **L3**	42% (28%)
7	5 mol% **L4**	35% (40%)
8	5 mol% **L5**	nd
9	5 mol% **L6**	nd
10	DCM instead of DMF/MeCN	nd
11	DMA instead of DMF/MeCN	25% (17%)
12	no PC or NiBr_2_ or Ph_3_P or light	nd

Standard conditions: photocatalyst (2 mol%), NiBr_2_·dme (3 mol%), **L1** (5 mol%), **1a** (0.2 mmol), **2a** (0.4 mmol), Ph_3_P (0.3 mmol), K_3_PO_4_ (0.2 mmol), Cs_2_CO_3_ (0.2 mmol), DMF-CH_3_CN (2.0 mL, v/v = 1:1), blue LEDs, ambient temperature, 20 h.

*DMF* *N*,*N*-dimethylformamide; *DMA* *N,N*-dimethylacetamide, *DCM* dichloromethane, *DME* 1,2-dimethoxyethane, *nd* not detected.

### Substrate scope

**Fig. 4:**
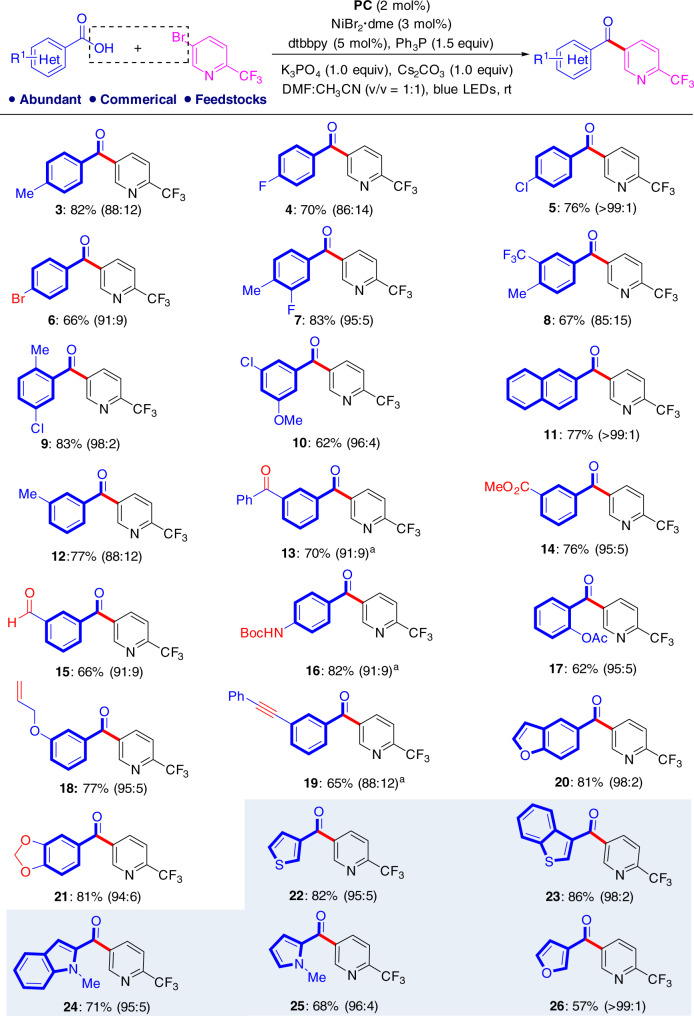
Carboxylic acid scope at a 0.2 mmol scale under standard conditions. The isolated yield of ketone is given for product and the GC ratio of ketone and ester is given in parenthesis. ^a^The ratio of ketone and ester is calculated based on isolation.

With the optimized conditions in hand, we investigated the scope of the cross-electrophile coupling reaction with regard to aromatic carboxylic acids, and obtained the results in Fig. 4. In general, this protocol is highly efficient and has a broad substrate scope. The electron-rich and electron-poor functional groups on the *ortho*-, *meta*-, and *para*-positions on the phenyl groups of the aromatic acids have little influence on the coupling process and the desired ketones (**3–21**) are formed in 62–83% yield. A series of useful functional groups, such as bromine (**6**), reactive carbonyl groups (**13–16**), a terminal alkene (**18**), an internal alkyne (**19**) and an acetal (**21**) tolerate the reaction conditions well. Some of these functional groups have difficulty surviving the conventional Weinreb ketone synthesis method toward Grignard reagents. Hetero-aromatic carboxylic acids are satisfactory starting materials and can uniformly produce the synthetically valuable diheteroaromatic ketones (**22–26**) with moderate to good yields. However, examination of the reaction of aliphatic carboxylic acids under these standard conditions showed that only a trace amount of the desired product can be produced while both decarboxylative C–C coupling and direct C–O coupling can occur.

**Fig. 5:**
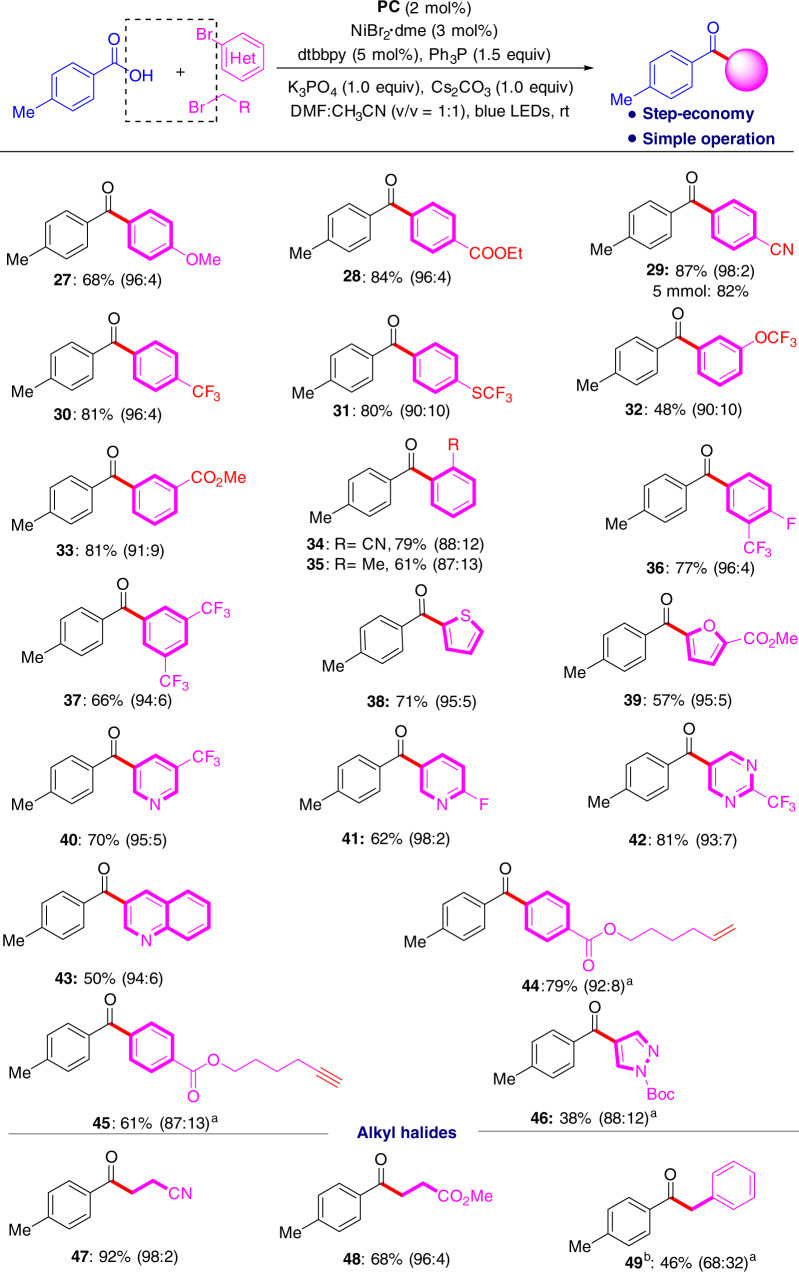
Organohalide scope on a 0.2 mmol scale under standard conditions. The isolated yield of ketone is given and the GC ratio of ketone and ester is given in parenthesis. ^a^The ratio of ketone and ester is calculated based on isolation. ^b^Benzyl chloride was used.

Subsequently, we studied the substrate scope of aromatic bromides (Fig. 5) and found that many commercially available aromatic bromides can be used to deliver the desired ketones (**27–46**) in good yields. The excellent functional group tolerance of –COOR (**28, 33, 44, 45**), –CN (**29, 34**), terminal unsaturated chemical bonds (**43, 44**), and heteroarenes (**38–43**, **46**) support the practicality of the reaction. With this strategy, it is also very easy to construct fluorine- and fluoroalkyl-containing diaryl ketones (**30–32, 36, 37, 39–42**) with acceptable yields. Several alkyl halides also serve as coupling partners in this cross-electrophile coupling reaction, leading to functionalized ketones (**47–49**) in good yields (up to 92%). When benzyl chloride was employed, 46% yield of ketone (**49**) was obtained and a significant amount of by-product ester was formed possibly because of the nucleophilic substitution side reaction. This coupling reaction can allow for the construction of highly functionalized ketones in an operationally simple, step-economical and gram-scale reaction (**29**, 5 mmol scale).

### Synthetic application

**Fig. 6:**
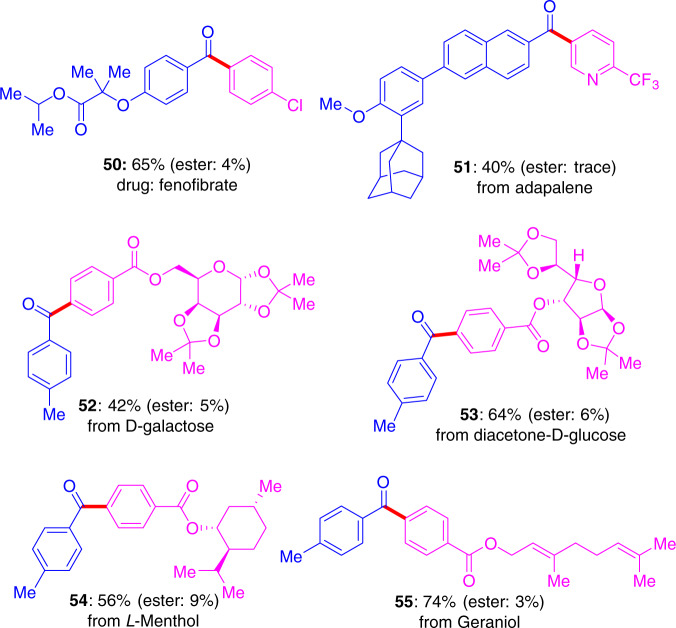
Concise synthesis of complex ketones. Carboxylic acids are blue and aromatic bromides are pink. The isolated yield of by-product ester is given in parenthesis.

To further demonstrate the synthetic robustness of the reaction, we applied the strategy for the construction of a series of complex ketones from carboxylic acids and aromatic bromides (Fig. 6). Fenofibrate (**50**) is a drug used to adjust lipid levels and blood viscosity and it could be prepared in one step in 65% yield. The complex ketones (**50–55**) can be obtained in synthetically useful yields. The precise cross-electrophile coupling also allows for introduction of functional groups at an early synthetic stage to limit the number of synthetic steps thus improving the efficiency.

### Mechanism of stoichiometric reactions

**Fig. 7:**
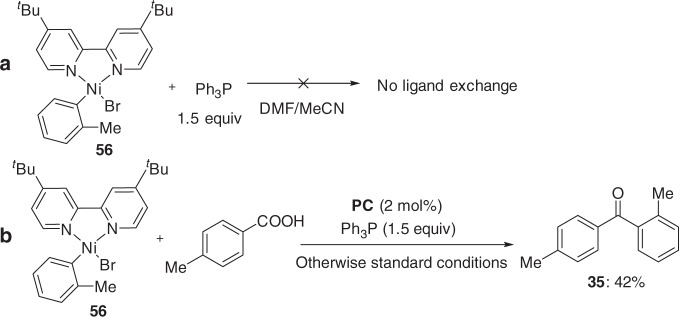
Mechanistic studies. **a** The reaction of Ph_3_P with Ar–Ni(II) intermediates. **b** Stoichiometric reactions of Ar–Ni(II) intermediates.

We performed the stoichiometric reactions of Ar–Ni(II) intermediate (**56**) with 1.5 equiv. Ph_3_P in DMF/MeCN. Interestingly, no ligand exchange was observed by ^31^P NMR analysis (Fig. 7, also see Supplementary Fig. 10). Treatment of Ar–Ni(II) intermediate (**56**) under the photoredox conditions, led to the desired deoxygenative C–C coupling product (**35**), which was obtained in 42% yield, further supporting the proposed mechanism (Fig. 7).

## Discussion

We have developed a cross-electrophile coupling between aromatic carboxylic acids and organic bromides, inexpensive and abundant feedstock chemicals, enabled by photoredox and a nickel and phosphoranyl radical synergistic combination, affording a wide array of structurally diverse ketones with excellent functional group compatibility. This strategy for ketone synthesis can significantly improve the synthetic efficiency and step-economy, and it also opens a door to construct highly functionalized or complex ketones which are still difficult to prepare by a conventional Weinreb ketone synthesis. We found that the use of appropriate ligand loading amount (5 mol% of 4,4′-di-*tert*-butyl-2,2′-bipyridine), mixed solvents (DMF/CH_3_CN) and combined inorganic bases (K_3_PO_4_ and Cs_2_CO_3_) is crucial to achieve the desired C–C bond formation reactions, affording the desired ketone products. The employment of more or less of ligand results in a sharply decreased yield of the ketone product and an increased yield of the ester by-product. Use of combined bases and mixed solvents would improve the deprotonation of carboxylic acids to expedite the acyl radical generation and use of the precise amount of a ligand would promote the acyl radical oxidative addition to the arylnickel (**II**) species. We speculated that a facile C–O bond cleavage and subsequent rapid acyl radical oxidative addition rate can control the selective C–C bond formation. We believe this cross-electrophile coupling strategy of carboxylic acids and organic halides will upgrade the synthesis of ketones with great potential application in organic synthesis, drug discovery and optochemical biology given the importance and ubiquity of ketones.

## Methods

### General procedure for cross-electrophile coupling of carboxylic acids and organohalides

Preparation of Ni-based catalyst solution: In the nitrogen-filled glove box, a stirring bar, NiBr_2_·dme (1.9 mg, 3.0 mol%), 4,4′-di-*tert*-butyl-2,2′-bipyridine (2.7 mg, 5.0 mol%) and CH_3_CN/DMF (2.0 mL, *V*/*V* = 1:1) were successively added to an oven-dried vial (8 mL screw-cap threaded). The vial was then sealed with a Teflon-lined plastic screw-cap and stirred until the resulting mixture become homogeneous (about 20 min).

Photocatalyst Ir[dF(CF_3_)ppy]_2_(dtbbpy)PF_6_ (4.5 mg, 2 mol%), aromatic carboxylic acid (0.2 mmol, 1.0 equiv), aryl bromide (0.4 mmol, 2.0 equiv), Ph_3_P (78.6 mg, 0.3 mmol, 1.5 equiv), anhydrous powder K_3_PO_4_ (42.4 mg, 0.2 mmol, 1.0 equiv), and anhydrous powder Cs_2_CO_3_ (65.0 mg, 0.2 mmol, 1.0 equiv) were added to an oven-dried 10 mL Schlenk tube equipped with a magnetic stirring bar. The tube was evacuated and backfilled with argon three times. Subsequently, the nickel-catalyst solution was transferred into this Schlenk tube under argon. The tube was then sealed and placed ~5 cm from 2 × 45 W blue LEDs. The reaction mixture was stirred for 20–36 h at room temperature (air-condition was used to keep the temperature is 25 °C or so). After completion, the reaction mixture was removed from the light, diluted with water and the aqueous layer was extracted with EtOAc (3 × 2.0 mL). The combined organic layers were washed with brine, dried over anhydrous Na_2_SO_4_, filtered, and concentrated. The residue was purified by flash chromatography on silica gel to afford the corresponding ketone products.

## Supplementary information

Supplementary Information

## Data Availability

The authors declare that all other data supporting the findings of this study are available within the article and Supplementary Information files, and also are available from the corresponding author upon reasonable request.
