# Supplementary material for: Upgrading ketone synthesis direct from carboxylic acids and organohalides
Source: Nat Commun. 2020 Jul 3;11:3312. doi: 10.1038/s41467-020-17224-2 (PMC7334210; doi:10.1038/s41467-020-17224-2)
Supplement: Supplementary file 1 — Supplementary Information [file 41467_2020_17224_MOESM1_ESM.pdf]

SUPPLEMENTARY INFORMATION

Upgrading Ketone Synthesis Direct from Carboxylic Acids and Organohalides

Ruzi et al.

Supplementary Methods

General information

All the reactions were carried out in Schlenk tubes under nitrogen atmosphere. Commercially available aryl, alkyl halides and carboxylic acids are used from commercial resource without further purification. Ni-catalysts were purchased from Strem or Aldrich and all ligands were purchased from J&K. K_3PO_4 was purchased from Aldrich and other bases purchased from Energy chemical (China). Solvents were purchased from J&K. All other reagents and solvents were obtained from commercial suppliers and used without further purification.

Analytical Methods. All reactions were carried out under argon atmosphere unless otherwise noted. Reactions were monitored by TLC on silica gel plates (GF254), and the analytical thin-layer chromatography (TLC) was performed on precoated, glass-backed silica gel plates. ^{1}H NMR, ^{13}C NMR and ^{19}F NMR spectra were recorded on a Bruker AVANCE III-400 or 500 spectrometer at room temperature. Chemical shifts (δ) are reported in ppm downfield from tetramethylsilane. Abbreviations for signal couplings are: s, singlet; d, doublet; t, triplet; m, multiple. GC-MS analyses were performed on a GC-MS with an EI mode. High resolution mass spectra were obtained using an Agilent 6210 Series TOF LC-MS equipped with electrospray ionization (ESI) probe operating in positive ion mode. Melting points (mp) were determined with a digital electrothermal apparatus without further correction. IR spectra were recorded on a Thermo Scientific Nicolet 380 FT-IR spectrometer. The 45 W blue LED lamps (λ_{max} = 455 nm) were purchased from Kessil (A360NE/WE).

Optimization of the reaction conditions

Supplementary Table 1. Screening of photocatalysts

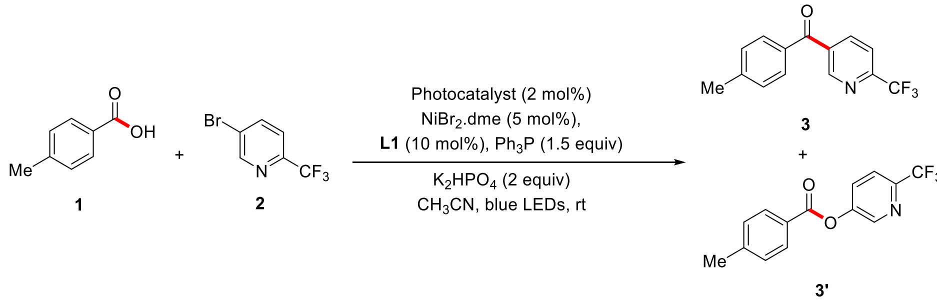

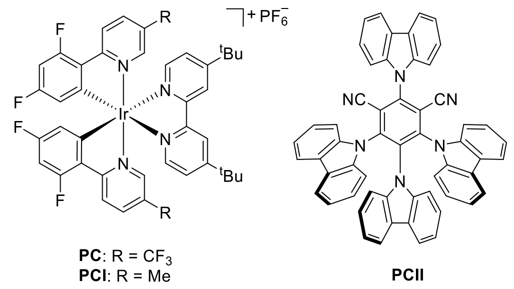

Entry	Photocatalyst	**3** (**3'**)
1	**PC**	18% (9%)
2	**PCI**	11% (10%)
3	**PCII**	nd

Reaction conditions: **1** (0.2 mmol), **2** (0.4 mmol), Ph_3P (1.5 equiv), $NiBr_2$•dme (5 mol%), **L1** = 4,4'-di-*tert*-butyl-2,2'-bipyridine (10 mol%), photocatalyst (2 mol%), K_2HPO_4 (2 equiv), CH_3CN (2 mL), blue LEDs, 25 °C, 20 h; isolated yield.

Supplementary Table 2. Screening of solvents

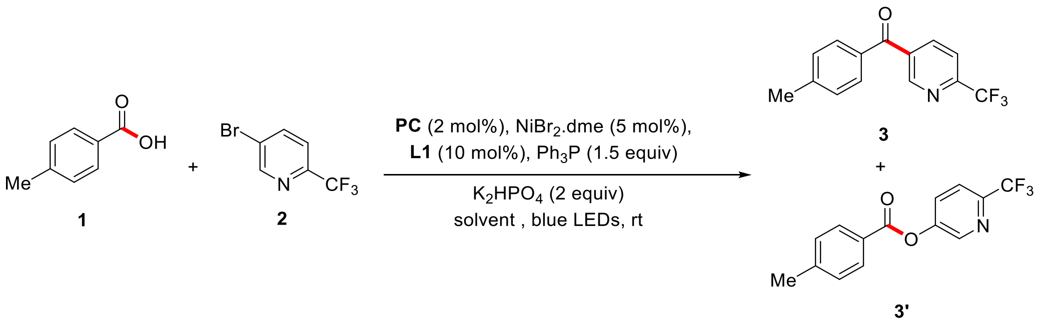

Entry	Solvent	**3** (**3'**)
1	DMF	23% (13%)
2	DMA	14% (19%)

3	DCM	nd

Reaction conditions: **1** (0.2 mmol), **2** (0.4 mmol), **PC** (2 mol%), Ph_3P (1.5 equiv), $NiBr_2$•dme (5 mol%), **L1** (10 mol%), K_2HPO_4 (2 equiv), solvent (2 mL), blue LEDs, 25°C, 20 h. **L1** = 4,4'-di-*tert*-butyl-2,2'-bipyridine. DMF = *N*, *N*-dimethylformamide, DMA = *N*, *N*-dimethylacetamide, DCM = dichloromethane; isolated yield.

Supplementary Table 3. Screening of bases

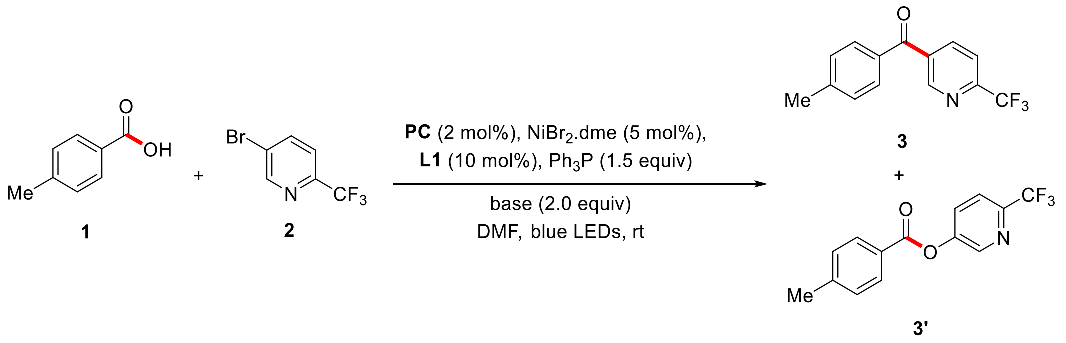

Entry	Base	**3 (3')**
1	K_2CO_3	23% (17%)
2	Cs_2CO_3	20% (8%)
3	K_3PO_4	26% (18%)
4	NaOAc	11% (9%)
5	2,6-Lutidine	nd
6	Triethylamine	nd

Reaction conditions: **1** (0.2 mmol), **2** (0.4 mmol), **PC** (2 mol%), $NiBr_2$•dme (5 mol%), **L1** (10 mol%), Ph_3P (1.5 equiv), base (2 equiv), DMF (2 mL); blue LEDs, 25 °C, 20 h. **L1** = 4,4'-di-*tert*-butyl-2,2'-bipyridine. DMF = *N*, *N*-dimethylformamide; isolated yield.

Supplementary Table 4. Screening for mixed bases and solvents

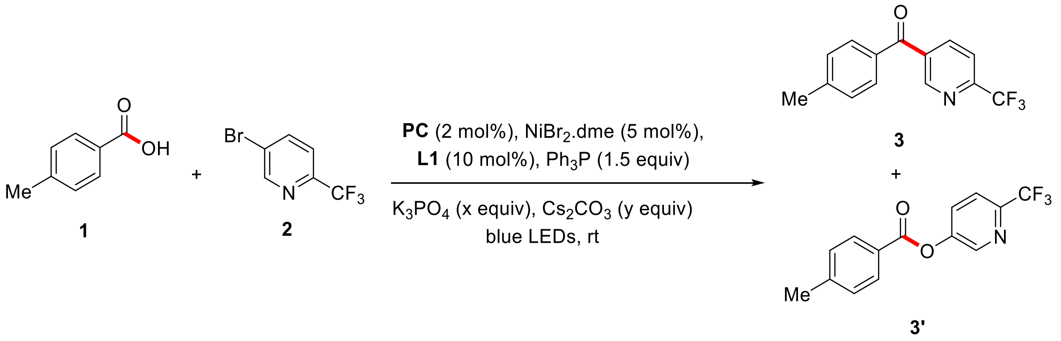

Entry	Solvent	Base	**3** (**3'**)
1	CH_3CN/DMF (V:V = 1:1)	K_3PO_4	30% (33%)
2	CH_3CN/DMF (V:V = 1:2)	K_3PO_4	27% (20%)
3	CH_3CN/DMF (V:V = 1:4)	K_3PO_4	25% (28%)
4	CH_3CN/DMF (V:V = 1:1)	K_3PO_4 (1 equiv)+Cs_2CO_3 (1 equiv)	46% (37%)
5	CH_3CN/DMF (V:V = 1:1)	K_3PO_4 (1 equiv)+Cs_2CO_3 (2 equiv)	nd

Reaction conditions: **1** (0.2 mmol), **2** (0.4 mmol), Ph_3P (1.5 equiv), **PC** (2 mol%), $NiBr_2$•dme (5 mol%), **L1** (10 mol%), DMF/CH_3CN (2 mL), blue LEDs, 25 °C, 20 h. **L1** = 4,4'-di-*tert*-butyl-2,2'-bipyridine. DMF = *N*, *N*-dimethylformamide; isolated yield.

Supplementary Table 5. Screening for amount of **L1**

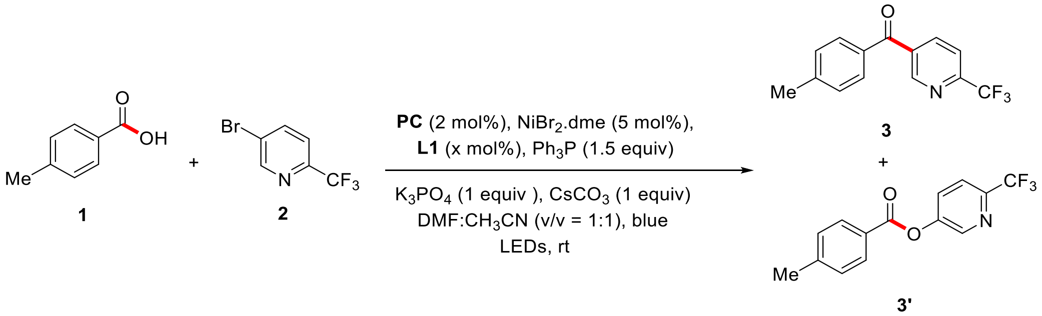

Entry	**L1** (x mol%)	**3** (**3'**)
1	15 mol%	23% (53%)
2	7.5 mol%	55% (24%)
3	5 mol %	71% (19%)
4[a]	5 mol%	82% (16%)
5[a]	10 mol%	46% (37%)

6[a]	15 mol%	23% (53%)
7[a]	3 mol%	19% (10%)

Reaction conditions: **1** (0.2 mmol), **2** (0.4 mmol), Ph_3P (1.5 equiv), K_3PO_4 (1 equiv), Cs_2CO_3 (1 equiv), $NiBr_2$•dme (5 mol%), **L1** (x mol%), DMF/CH_3CN (2 mL, V/V = 1:1), blue LEDs, 25 °C, 20 h. **L1** = 4,4'-di-*tert*-butyl-2,2'-bipyridine. DMF = *N*, *N*-dimethylformamide; isolated yield. [a]$NiBr_2$•dme (3 mol%).

Supplementary Table 6. Screening of other ligands

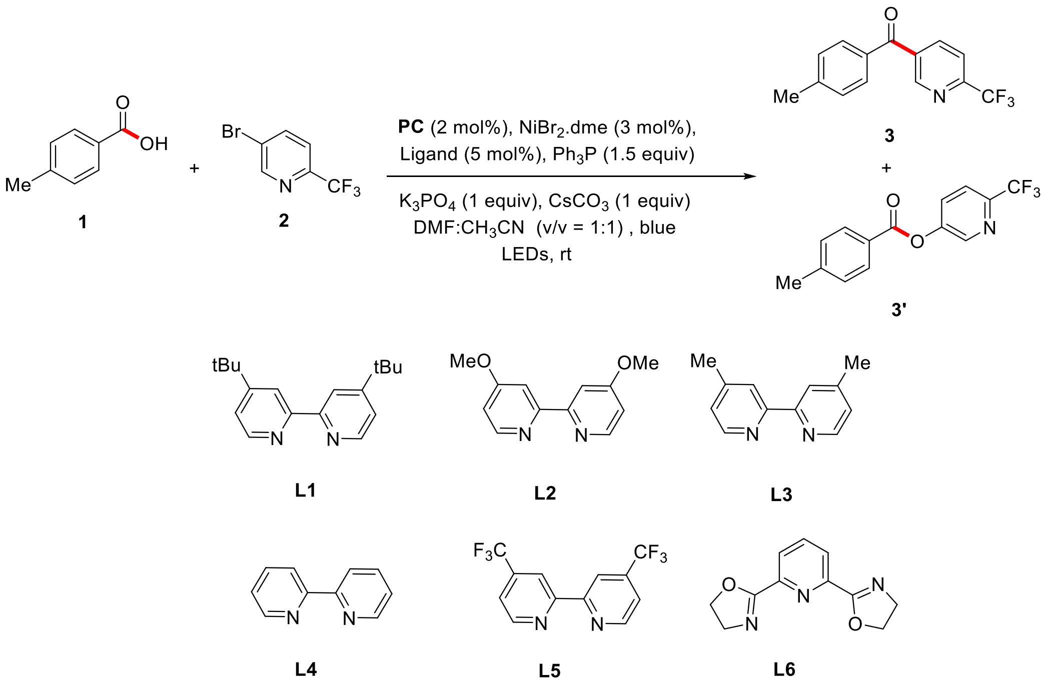

Entry	Ligand	**3 (3')**
1	**L1**	82% (16%)
2	**L2**	31% (33%)
3	**L3**	42% (28%)
4	**L4**	35% (40%)
5	**L5**	nd
6	**L6**	nd

Reaction conditions: **1** (0.2 mmol), **2** (0.4 mmol), Ph_3P (1.5 equiv), K_3PO_4 (1 equiv), Cs_2CO_3 (1 equiv), ligand (5 mol%) , DMF:CH_3CN (2 mL, V/V = 1:1), blue LEDs, 25 °C, 20 h. DMF = *N*, *N*-dimethylformamide, DMA = *N*, *N*-dimethylacetamide, DCM = dichloromethane; isolated yield.

Supplementary Table 7. Control experiments

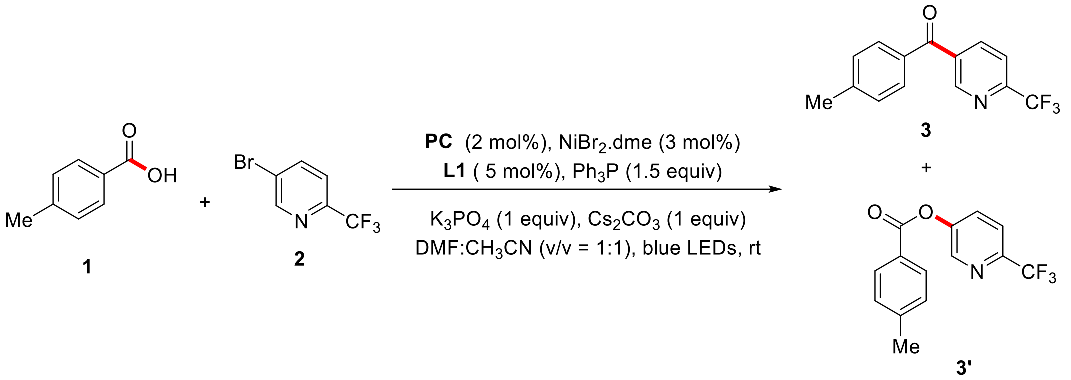

Entry	Variation of conditions	**3** (**3’**)
1	no **PC**	nd
2	no $NiBr_2$•dtbbpy	nd
3	no Ph_3P	nd
4	no light	nd

Reaction conditions: **1** (0.2 mmol), **2** (0.4 mmol), Ph_3P (1.5 equiv), K_3PO_4 (1 equiv), Cs_2CO_3 (1 equiv), DMF:CH_3CN (2 mL, V/V = 1:1), blue LEDs, 25 °C, 20 h. DMF = N, N-dimethylformamide, DMA = *N, N*-dimethylacetamide, DCM= dichloromethane.

Supplementary Table 8. The initial testes for aliphatic carboxylic acids

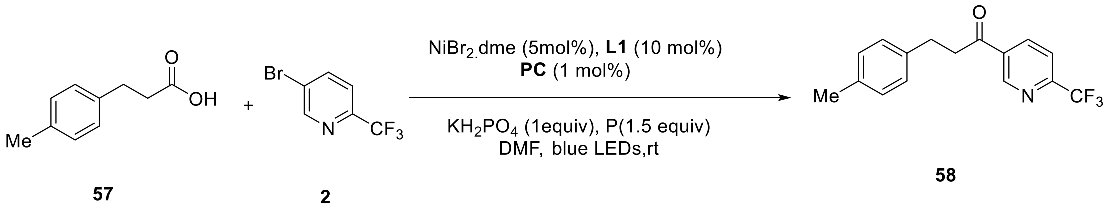

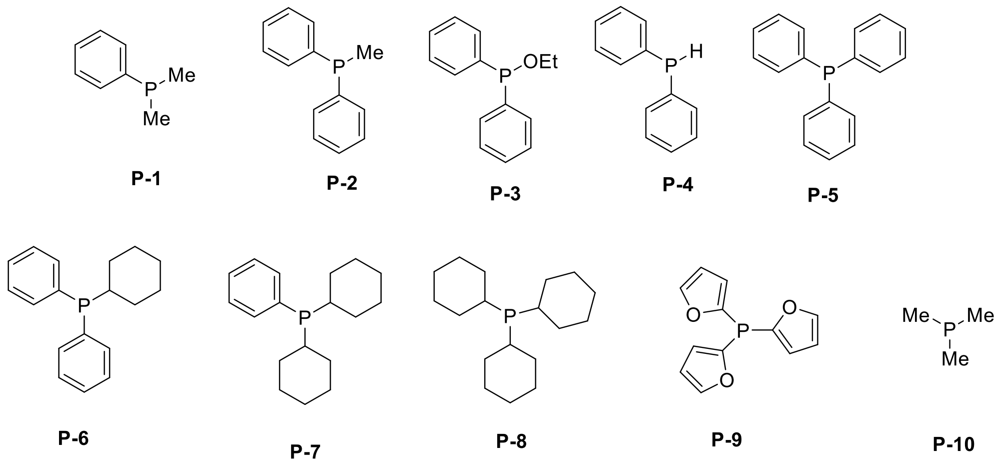

Entry	Phosphine	Result
1	**P-1**	nd
2	**P-2**	nd
3	**P-3**	nd
4	**P-4**	nd
5	**P-5**	nd
6	**P-6**	trace
7	**P-7**	trace
8	**P-8**	nd
9	**P-9**	nd
10	**P-10**	nd

Reaction conditions: **57** (0.2 mmol), **2** (0.4 mmol), phosphine reagent (1.5 equiv), K_2HPO_4 (1 equiv), DMF (2 mL), blue LEDs, 25 °C, 20 h. DMF = *N*, *N*-dimethylformamide.

General procedure for the reaction conditions

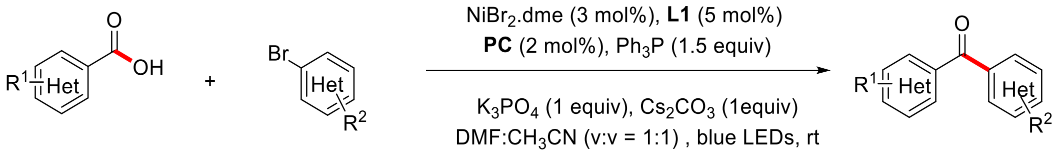

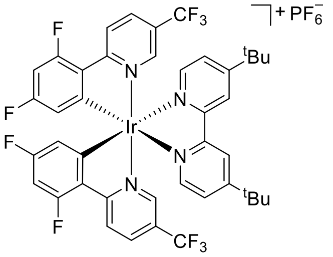

PC

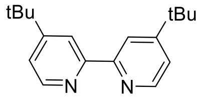

L1

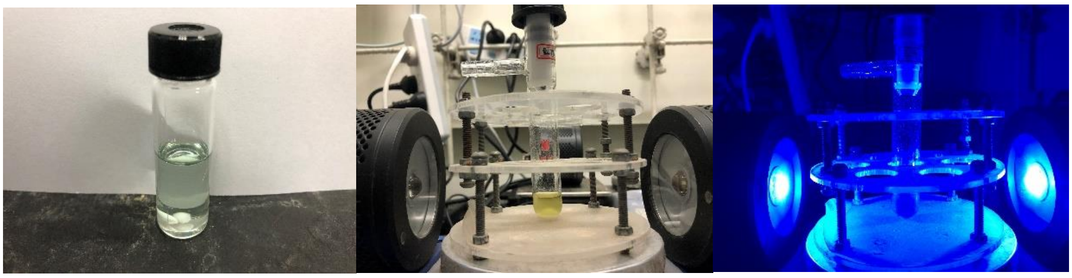

Ni precatalyst　　　　air-condition in fume hood

Supplementary Figure 1. Reaction set-up

In the nitrogen-filled glove box, an oven-dried vial (8 mL screw-cap threaded) was successively added stirring bar, $NiBr_2$•dme (1.9 mg, 3.0 mol%), 4,4'-di-tertbutyl-2,2'-bipyridine (2.7 mg, 5.0 mol%) and CH_3CN/DMF (2.0 mL, V/V = 1:1), and then it was sealed with a Teflon-lined plastic screw-cap and stirred until the resulting mixture became homogenous solution (about 20 min).

An oven-dried 10 mL Schlenk tube equipped with a magnetic stir bar was added photocatalyst Ir[dF(CF_3)ppy]$_2$(dtbbpy)PF_6 (4.5 mg, 2 mol%), aromatic carboxylic acid (0.2 mmol, 1.0 equiv), aryl bromide (0.4 mmol, 2.0 equiv), Ph_3P (78.6 mg, 0.3 mmol, 1.5 equiv), anhydrous powder K_3PO_4 (42.4 mg, 0.2 mmol, 1.0 equiv), and anhydrous powder Cs_2CO_3 (65.0 mg, 0.2 mmol, 1.0 equiv). Subsequently, the nickel-catalyst solution was transferred into the Schlenk tube under argon. The tube was evacuated and backfilled with argon three times. The tube was then sealed and was placed at a distance (app. 5 cm) from 2 x 45 W blue LEDs, and the mixture was stirred for 20-36 h at room temperature (air-condition was used to keep the temperature is 25 ^{o}C or so). After completion, the reaction mixture was removed from the light, diluted with water and EtOAc, and then aqueous layer was extracted with three portions of EtOAc. The combined organic layers were washed with brine, dried over anhydrous Na_2SO_4, filtered, and concentrated. The residue was purified by flash chromatography on silica gel to afford the corresponding desired ketone product.

Procedure for the gram-scale reaction

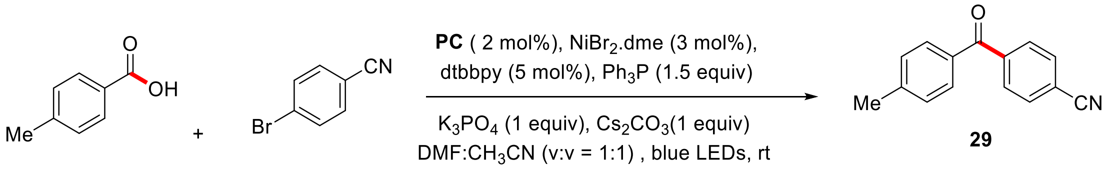

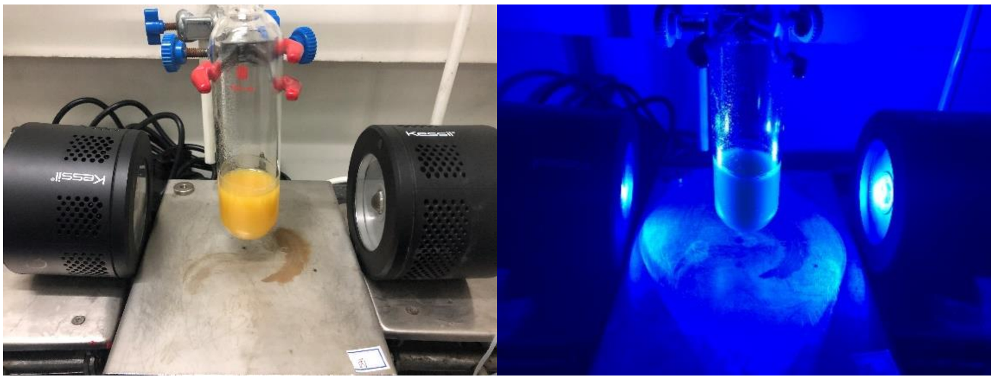

Supplementary Figure 2. Reaction set-up

In the nitrogen-filled glove box, an oven-dried 50 mL round bottom flask was added $NiBr_2$•dme (46.2 mg, 3.0 mol%) and 4,4'-di-tertbutyl-2,2'-bipyridine (67.0 mg, 5.0 mol%) and dissolved in CH_3CN:DMF (25.0 mL, V/V = 1:1). The round bottom flask was sealed and stirred until the resulting mixture became homogenous solution (about 30 min). An oven-dried 100 mL Schlenk tube equipped with a magnetic with stir bar was added aromatic 4-methylbenzoic acid (680.0 mg, 5 mmol), 4-bromobenzonitrile (1.82 g, 10 mmol), Ph_3P (1.97 g, 7.5 mmol), anhydrous K_3PO_4 (1.06 mg, 5.0 mmol), anhydrous Cs_2CO_3 (1.63 g, 5 mmol) and Ir[dF(CF_3)ppy]$_2$(dtbbpy)PF_6 (56.1 mg, 1.0 mol%) under argon. Subsequently, the homogenous solution was syringed into the tube under nitrogen. The tube was then sealed and was placed at a distance (app. 5 cm) from 2 x 45 W blue LEDs, and the mixture was stirred for 20 h at room temperature (air-condition was used to keep the temperature is 25 °C or so). After completion, the reaction mixture was removed from the light, diluted with water and EtOAc, and then aqueous layer was extracted with three portions of EtOAc. The combined organic layers were washed with brine, dried over anhydrous Na_2SO_4, filtered, and concentrated. The residue was purified by flash chromatography (eluent: PE: EA = 10:1) on silica gel to afford the desired ketone **29** in 906 mg with 82% yield.

Mechanistic investigations

Control experiment with additives

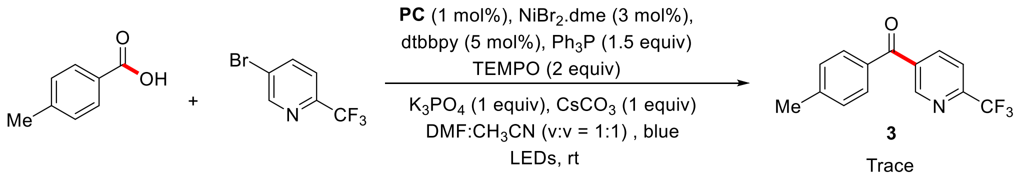

In the nitrogen-filled glove box, an oven-dried vial (8 mL screw-cap threaded) was added NiBr$_2$•dme (1.9 mg, 3.0 mol%), 4,4'-di-tertbutyl-2,2'-bipyridine (2.7 mg, 5.0 mol%) and dissolved in CH$_3$CN:DMF (2.0 mL, V/V = 1:1). The vial was sealed with a Teflon-lined plastic screw-cap and NiBr$_2$•dtbbpy precatalyst can be formed in situ until homogenous (about 20 min). An oven-dried 10 mL Schlenk tube equipped a magnetic with stir bar as well as photocatalyst Ir[dF(CF$_3$)ppy]$_2$(dtbbpy)PF$_6$ (4.5 mg, 2 mol%), 4-methylbenzoic acid (27.2 mg, 0.2 mmol), 5-bromo-2- (trifluoromethyl)pyridine (90.4 mg, 0.4 mmol), Ph$_3$P (78.6 mg, 0.3 mmol, 1.5 equiv), anhydrous powder K$_3$PO$_4$ (42.4 mg, 0.2 mmol, 1.0 equiv), anhydrous powder Cs$_2$CO$_3$ (65.0 mg, 0.2 mmol, 1.0 equiv), and TEMPO (62.4 mg, 0.4 mmol) under argon. Subsequently, the homogenous solution was syringed into the tube under argon. The tube was then sealed and was placed at a distance (app. 5 cm) from 2 x 45 W blue LEDs, and the mixture was stirred for 20 h at room temperature (air-condition was used to keep the temperature is 25 °C or so). After completion, the reaction mixture was removed from the light. The corresponding product **3** was obtained in trace yield by TLC analysis. Meanwhile, the product 2,2,6,6-tetramethylpiperidin-1-yl 4-methylbenzoate could be detected by ESI-HRMS, it further demonstrates the generation of acyl radical during the reaction process.

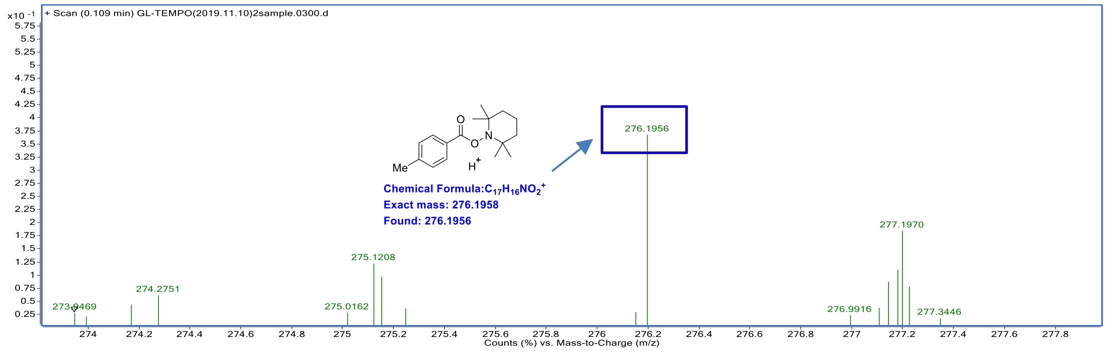

Supplementary Figure 3. The HRMS -ESI spectra.

Quantum yield measurement

The quantum yield (ϕ) was determined by the known ferrioxalate actinometry method. A ferrioxalate actinometry solution was prepared by following the Hammond variation of the Hatchard and Parker procedure outlined in Handbook of Photochemistry. The actinometry synthetic application solutions (1 mL) were irradiated with two 45 W blue LEDs for 0 s, 30 s, 60 s, 90 s and 120 s. The UV-Vis spectra are shown in **Supplementary Figure 4**. Based on the data, we got the graph **Supplementary Figure 5** between the number of moles of products (y axis) and time (x axis). Then, the irradiated light intensity was estimated to 6.6571E^{-8} einstein/s by using $K_3[Fe(C_2O_4)_3]$ as an actinometer. For five clean tubes, according to the general procedure, the 0.2 mmol scale model reaction solution was irradiated with two 45 W blue LEDs for specified time intervals (0 min, 30 min, 60 min, 90 min and 120 min). The moles of products formed were determined by GC yield with acetophenone as reference standard. The number of moles of products (y axis) per unit time is related to the number of photons (x axis, calculated from the light intensity) **Supplementary Figure 6**. The slope gives the quantum yield (Φ) of the photoreaction, 0.3505 (35%).

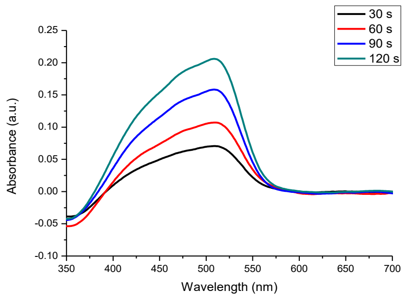

Supplementary Figure 4. The UV-Vis absorption spectra of actinometry solution

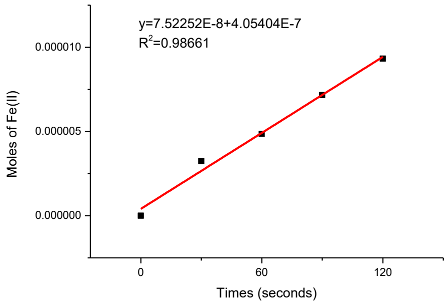

Supplementary Figure 5. The data of UV-Vis absorption of actinometry solution

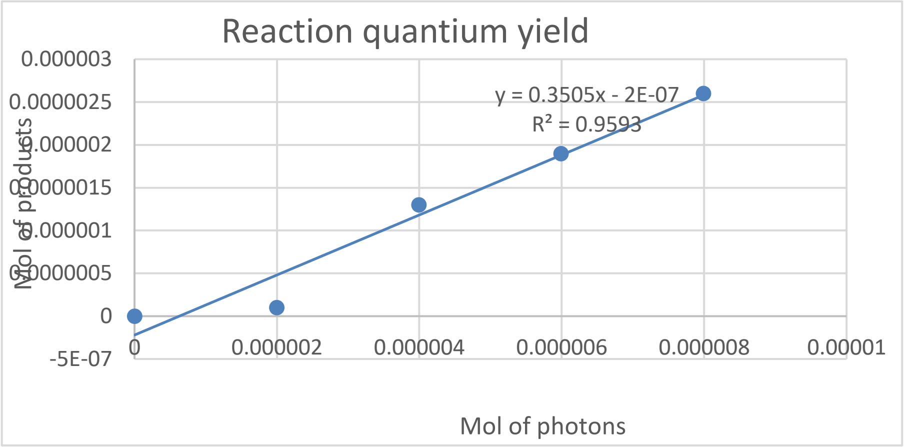

Supplementary Figure 6. Data of quantum yield measurement.

In order to determine whether a radical-chain reaction is involved, the quantum yield measurement was conducted, and gives the quantum yield (Φ) of the photoreaction, 35%. Thus, a radical chain pathway is less likely.

Luminescence quenching experiment

The luminescence quenching experiment was taken using a Cary Eclipse fluorescence spectrophotometer (Varian, USA). The experiments were carried out in 1 x 10^{-6} mol/L of $[Ir\{dF(CF_3)ppy\}_2\{dtbbpy\}]PF_6$ in CH_3CN at 25 °C. The emission intensity was collected at 475 nm. The concentrations of quenchers (**1** and Ph_3P) in DCM were 0, 3, 6, 9, 12, 15 mM.

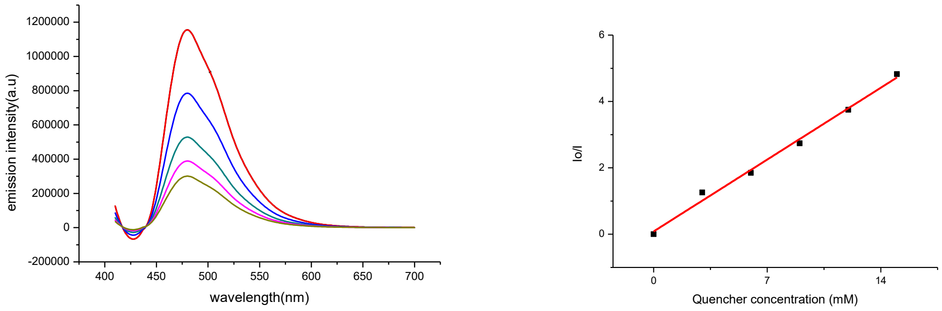

Supplementary Figure 7. Luminescence quenching of $[Ir\{dF(CF_3)ppy\}_2\{dtbbpy\}]PF_6$ by Ph_3P

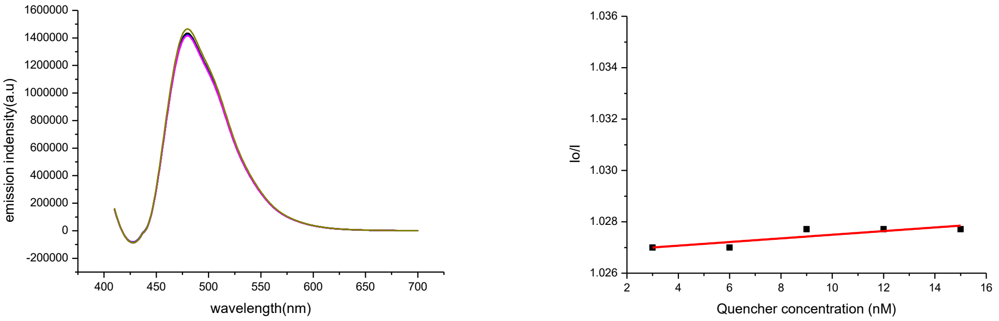

Supplementary Figure 8. Luminescence quenching of [Ir{dF(CF_3)ppy}$_2${dtbbpy}]PF_6 by acid **1**

To determine whether a reductive or oxidative quenching cycle is operative in the reaction, fluorescence quenching studies were conducted. Based on the above data, photoexcited [Ir{dF(CF_3)ppy}$_2${dtbbpy}]PF_6* can be quenched by Ph_3P, involving a reductive quenching cycle.

Stoichiometric reactions for the Ar-Ni(II) intermediates

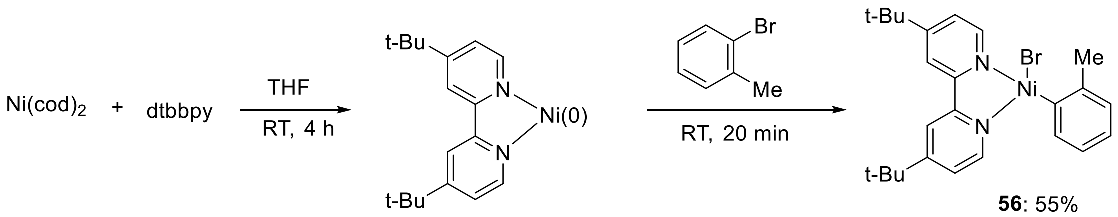

56: 55%

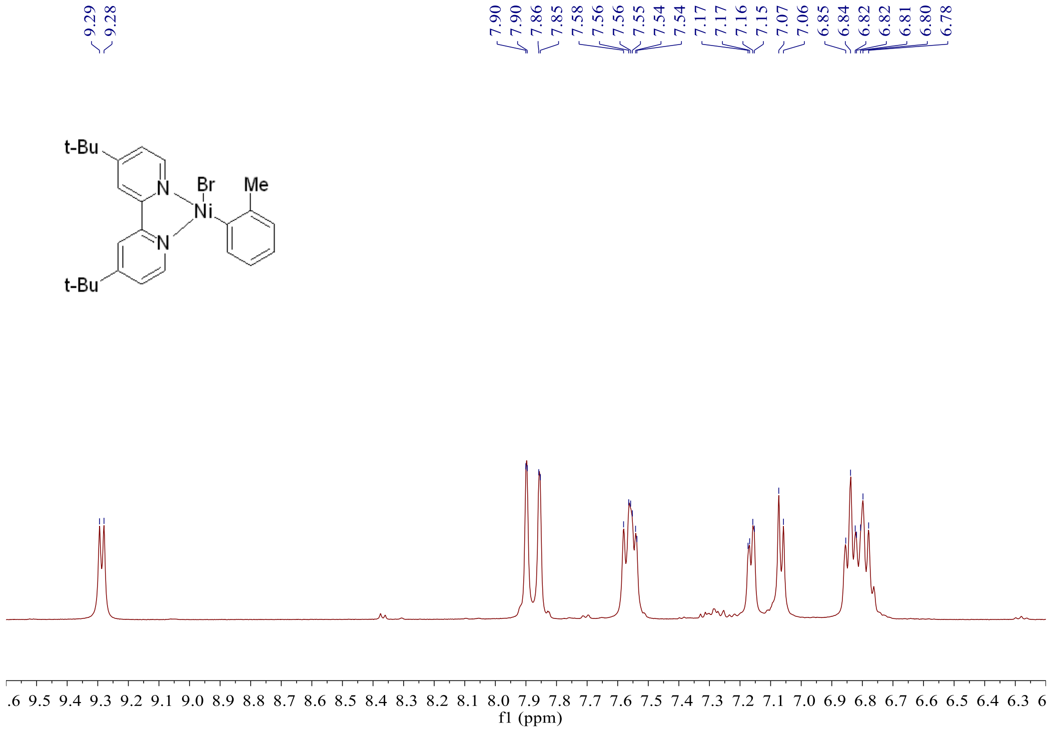

Supplementary Figure 9. The ^{1}H NMR spectrum of aryl-Ni(II) complex **56**.

According to the reported literature[1], in the nitrogen-filled glove box, an oven-dried 8 mL screw-cap vial equipped with stir bar was charged with $Ni(cod)_2$ (275 mg, 1.0 mmol), 4,4′-di-*tert*-butyl- 2,2′-pyridine (268 mg, 1.0 mmol) and THF (5 mL). The vial was sealed (deep purple resulting solution) and stirred for 4 hours at ambient temperature. 2-Bromotoluene (0.5 mL, 4.15 mmol) was added and then the vial was continually stirred for 20 min. The resulting solution turned dark red. Subsequently, the solution was triturated with pentane (10 mL). The precipitate was filtered, washed with pentane and residual solvent was removed under vacuum to give the title compound **56** as a light red powder 275 mg, 55% yield. The title compound was used for spectroscopic studies without further purification.

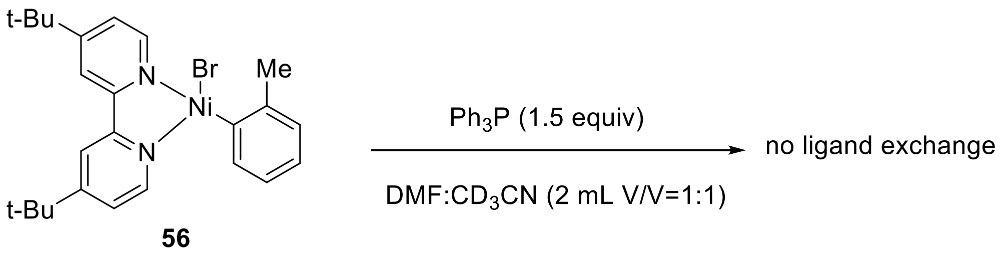

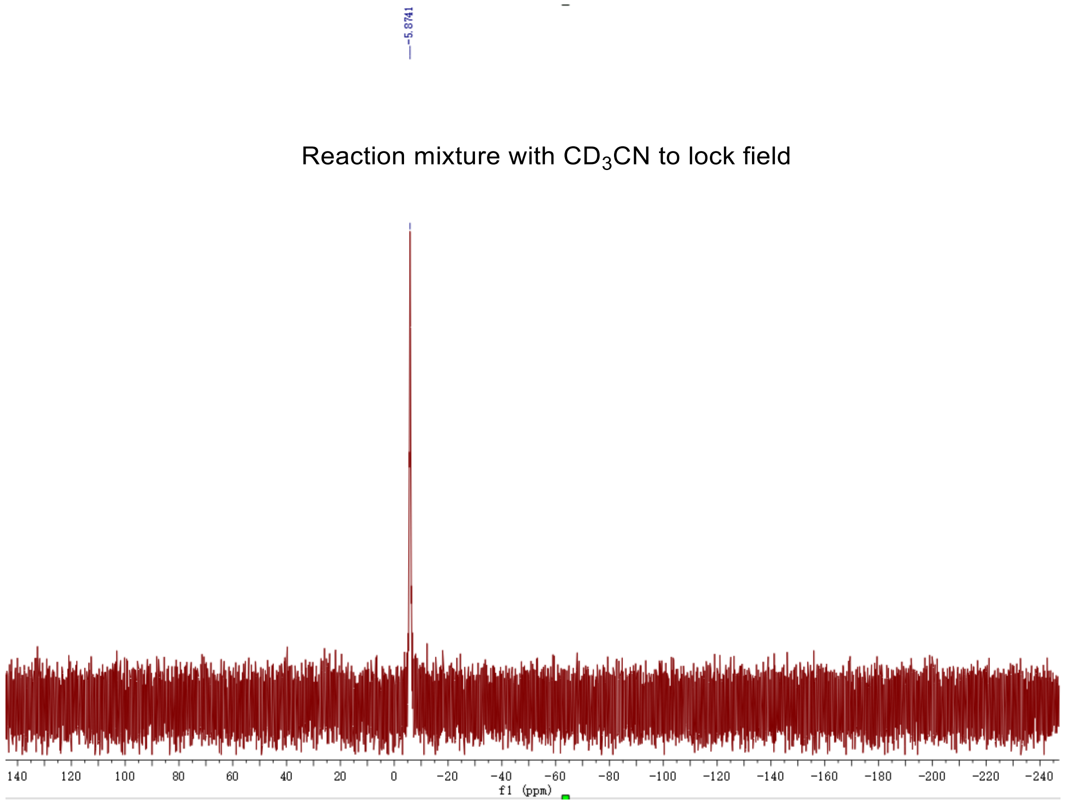

Supplementary Figure 10. ^{31}P NMR of reaction mixture

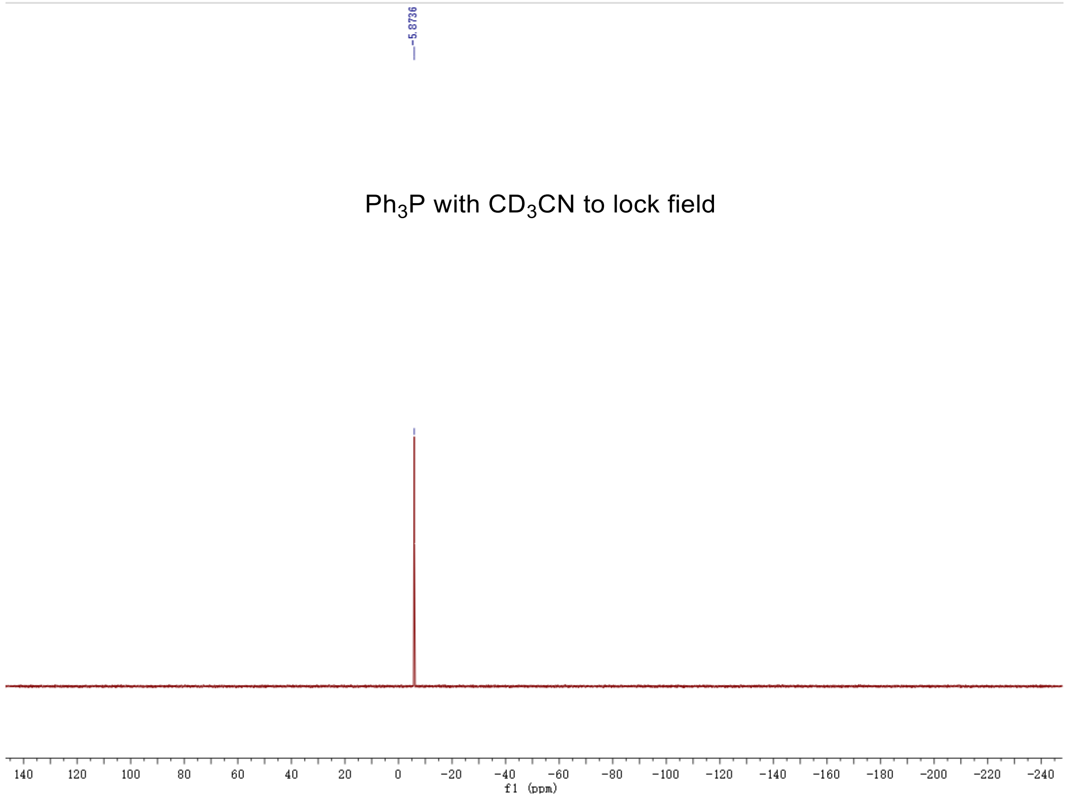

Supplementary Figure 11. ^{31}P NMR of Ph_3P

To a nitrogen-filled 10 mL Schlenk tube equipped with a magnetic stir bar was added **56** (49.8 mg, 0.1 mmol) and triphenylphosphine (39.3 mg, 0.15 mmol). Subsequently, the tube was evacuated and backfilled with Ar (three times). The solvent DMF(d7):CD_3CN (2.0 mL, V/V=1:1) was added by syringe under Ar. The tube was then sealed and the mixture was stirred for 20 h at room temperature. The reaction mixture was monitored by ^{31}P NMR in situ under argon. ^{31}P NMR (162 MHz, DMF-d_7) δ -5.87. It suggested that the large excess of Ph_3P didn't result in a ligand exchange process in the presence of Ar-Ni(II) intermediate.

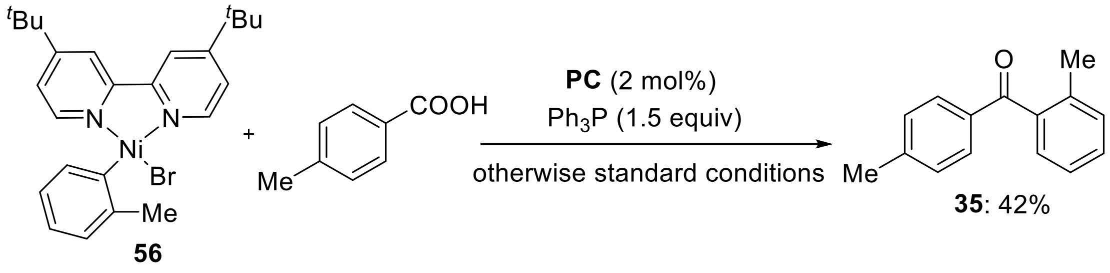

To a nitrogen-filled 10 mL Schlenk tube equipped with a magnetic stir bar was added 4-methylbenzoic acid (13.6 mg, 0.1 mmol), **56** (99.6 mg, 0.2 mmol) and triphenylphosphine (39.3 mg, 0.15 mmol), anhydrous powder K_3PO_4 (21.2 mg, 0.1 mmol) and anhydrous powder Cs_2CO_3 (32.5 mg, 0.1 mmol). Subsequently, the tube was evacuated and backfilled with Ar (three times). The solvent DMF/CH_3CN (2.0 mL, V/V = 1:1) was added by syringe under Ar. The tube was then sealed and the mixture was stirred for 20 h at room temperature. The reaction mixture was removed from the light, diluted with water and EtOAc, and then aqueous layer was extracted with three portions of EtOAc. The combined organic layers were washed with brine, dried over anhydrous Na_2SO_4, filtered, and concentrated. The residue was purified by flash chromatography on silica gel to afford **35**, 8.8 mg, 42% yield.

Characterization data of products

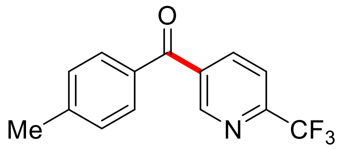

p-tolyl(6-(trifluoromethyl)pyridin-3-yl)methanone 3

The reaction was carried out according to the general procedure on 0.2 mmol scale (20 h). The crude product was purified by flash chromatography on SiO_2 (eluent: PE:EA = 100:1) to afford **3**, 43.5 mg, 82%, light yellow solid, mp = 85-87 °C, Rf = 0.3 (PE/EA = 50:1). ^{1}H NMR (400 MHz, $CDCl_3$) δ 9.0 - 8.95 (m, 1H), 8.21 - 8.16 (m, 1H), 7.76 (d, J = 8.0 Hz, 1H), 7.66 (d, J = 8.0 Hz, 2H), 7.27 (d, J = 8.0 Hz, 2H), 2.40 (s, 3H). ^{13}C NMR (100 MHz, $CDCl_3$) δ 192.1, 149.6, 149.3 (q, J= 35.0 Hz), 144.0, 137.6, 135.0, 132.6, 129.3, 128.5, 120.1 (q, J= 273.0 Hz), 119.2 (q, J= 2.0 Hz), 20.8. ^{19}F NMR (376 MHz, Chloroform-*d*) δ -68.2. HRMS (ESI) Calculated for $C_{14}H_{11}F_3NO$ $[M+H]^+$: 266.0787, found: 266.0789. IR ν (neat, cm^{-1}): 3053.8, 2306.1, 1664.2, 1421.8, 1264.1, 1084.6, 895.9, 730.8.

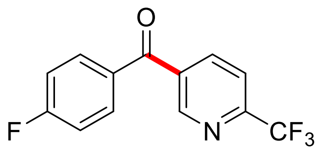

(4-fluorophenyl)(6-(trifluoromethyl)pyridin-3-yl)methanone 4

The reaction was carried out according to the general procedure on 0.2 mmol scale (36 h). The crude product was purified by flash chromatography on SiO_2 (eluent: PE:EA = 100:1) to afford **4**, 37.7 mg, 70%, light yellow solid, mp = 67-69 °C, Rf = 0.4 (PE/EA = 20:1). ^{1}H NMR (400 MHz, $CDCl_3$) δ 8.97 (d, *J* = 4.0 Hz, 1H), 8.18 (dd, *J* = 8.0, 4.0 Hz, 1H), 7.83-7.75 (m, 3H), 7.19-7.11 (m, 2H).^{13}C NMR (100 MHz, Chloroform-*d*) δ 192.0, 166.1(d, *J* = 255.0 Hz), 150.7 (q, *J* = 35.0 Hz), 150.5, 138.6, 135.5, 132.8 (d, *J* = 9.0 Hz), 132.3 (d, *J* = 3.0 Hz), 124.6 (q, *J* = 273.0 Hz), 120.4 (d, *J* = 2.0 Hz), 116.2 (d, *J* = 22.0 Hz). ^{19}F NMR (471 MHz, $CDCl_3$) δ -68.2, -103.1. HRMS (ESI) Calculated for $C_{13}H_8F_4NO$ $[M+H]^+$: 270.0537, found: 270.0532. IR *v* (neat, cm^{-1}): 3077.3, 2923.9, 2348.6, 1666.5, 1598.4, 1332.3, 1306.7, 1151.8, 1083.8, 951.7, 749.6, 612.0.

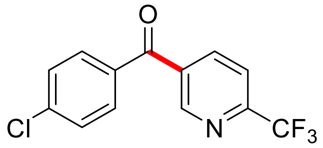

(4-chlorophenyl)(6-(trifluoromethyl)pyridin-3-yl)methanone 5

The reaction was carried out according to the general procedure on 0.2 mmol scale (36 h). The crude product was purified by flash chromatography on SiO_2 (eluent: PE:EA = 100:1) to afford **5**, 43.3 mg, 76%, light yellow solid, mp = 125-127 °C, Rf = 0.3 (PE/EA = 50:1). ^{1}H NMR (400 MHz, $CDCl_3$) δ 9.06 - 8.04 (m, 1H), 8.27 (dd, *J* = 8.0 Hz, *J* = 4.0 Hz, 1H), 7.86 (d, *J* = 8.0 Hz, 2H), 7.79 (d, *J* = 8.0 Hz, 2H), 7.53 (d, *J* = 8.0 Hz, 2H). ^{13}C NMR (100 MHz, $CDCl_3$) δ 192.3, 151.1 (q, *J* = 35.0 Hz), 140.5, 138.6, 135.3, 134.3, 131.4, 129.3, 121.1 (q, *J* = 273.0 Hz), 120.4 (q, *J* = 3.0 Hz). ^{19}F NMR (376 MHz, Chloroform-*d*) δ -68.2. HRMS (ESI) Calculated for $C_{13}H_8ClF_3NO$ $[M+H]^+$: 286.0241, found: 286.0248. IR *v* (neat, cm^{-1}): 3068.2, 2921.0, 1655.8, 1584.5, 1330.1, 1281.6, 1142.6, 1084.3, 930.6, 784.0.

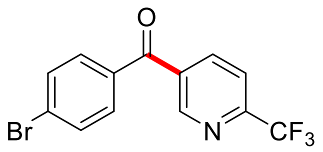

(4-bromophenyl)(6-(trifluoromethyl)pyridin-3-yl)methanone 6

The reaction was carried out according to the general procedure on 0.2 mmol scale (36 h). The crude product was purified by flash chromatography on SiO_2 (eluent: PE:EA = 100:1) to afford **6**, 43.3 mg, 66%, light yellow solid, mp = 130-132 °C, Rf = 0.3 (PE/EA = 50:1). ^{1}H NMR (400 MHz, $CDCl_3$) δ 8.99 - 8.95 (m, 1H), 8.19 (dd, *J* = 8.0, 4.0 Hz, 1H), 7.78 (d, *J* = 8.0 Hz, 1H), 7.63 (s, 4H). ^{13}C NMR (100 MHz, Chloroform-*d*) δ 192.5, 150.6, 149.9 (q, *J* = 23.0 Hz), 138.6, 135.2, 134.7, 133.8, 132.3, 131.5, 130.1, 129.2, 128.9, 120.5 (q, *J* = 273.0 Hz), 120.4 (q, *J* = 2.0 Hz). ^{19}F NMR (376 MHz, Chloroform-*d*) δ -68.2. HRMS (ESI) Calculated for $C_{13}H_8BrF_3NO$ $[M+H]^+$: 329.9736, found: 329.9736. IR *v* (neat, cm^{-1}): 2921.3, 2851.1, 1655.5, 1585.4, 1334.1, 1247.8, 1125.7, 1070.1, 862.7, 707.9, 477.0.

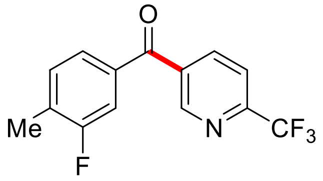

(3-fluoro-4-methylphenyl)(6-(trifluoromethyl)pyridin-3-yl)methanone 7

The reaction was carried out according to the general procedure on 0.2 mmol scale (20 h). The crude product was purified by flash chromatography on SiO_2 (eluent: PE:EA = 100:1) to afford **7**, 47.0 mg, 83%, light yellow solid, mp = 85-87 °C, Rf = 0.4 (PE/EA =20:1). ^{1}H NMR (500 MHz, Chloroform-*d*) δ 9.08 - 9.05 (m, 1H), 8.28 (dd, *J* = 10.0 Hz, *J* = 5.0 Hz, 1H), 7.87 (d, *J* = 10.0 Hz, 1H), 7.53 - 7.49 (m, 2H), 7.37 (t, *J* = 10.0 Hz, 1H), 2.41 (d, *J* = 5.0 Hz, 3H). ^{13}C NMR (125 MHz, Chloroform-*d*) δ 192.0, 161.22 (d, *J* = 246.3 Hz), 150.9 (q, *J* = 35.0 Hz), 150.5, 138.6, 135.5 (d, *J* = 6.3 Hz), 132.0 (d, *J* = 17.5 Hz), 131.9 (d, *J* = 5.0 Hz), 125.9 (d, *J* = 2.5 Hz), 121.1 (q, *J* = 272.5 Hz), 120.3 (q, *J* = 2.5 Hz), 116.3 (d, *J* = 23.8 Hz), 15.0 (d, *J* = 3.8 Hz). ^{19}F NMR (471MHz, $CDCl_3$) δ -68.2, -115.0. HRMS (ESI) Calculated for $C_{14}H_9F_4NNaO$ $[M+Na]^+$: 306.0512, found: 306.0509. IR *v* (neat, cm^{-1}): 2356.7, 1662.1, 1607.8, 1420.6, 1264.6, 1148.2, 904.9, 724.8, 649.9.

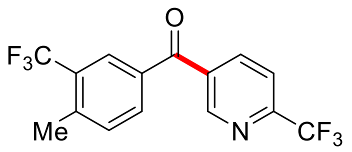

(4-methyl-3-(trifluoromethyl)phenyl)(6-(trifluoromethyl)pyridin-3-yl)methanone 8

The reaction was carried out according to the general procedure on 0.2 mmol scale (36 h). The crude product was purified by flash chromatography on SiO_2 (eluent: PE:EA = 100:1) to afford **8,** 45.0 mg, 67%, light yellow solid, mp = 78-80 ºC, Rf = 0.3 (PE/EA = 50:1). ^{1}H NMR (400 MHz, $CDCl_3$) δ 8.98 (d, *J* = 4.0 Hz, 1H), 8.20 (dd, *J* = 8.0, 4.0 Hz, 1H), 8.03 (d, *J* = 4.0 Hz, 1H), 7.80 (dd, *J* = 8.0, 4.0 Hz, 2H), 7.41 (d, *J* = 8.0 Hz, 1H), 2.54 (s, 3H). ^{13}C NMR (125 MHz, $CDCl_3$) δ 192.0, 150.6 (q, *J* = 35.0 Hz), 143.3, 138.6, 135.1, 133.8, 133.0, 132.6, 129.9 (q, *J* = 31.3 Hz), 127.4 (q, *J* = 5.0 Hz), 123.7 (q, *J* = 273.0 Hz), 121.1 (q, *J* = 273.0 Hz), 120.5 (q, *J* = 2.5 Hz), 20.0. ^{19}F NMR (471 MHz, $CDCl_3$) δ -62.1, -68.2. HRMS (ESI) Calculated for $C_{15}H_{10}F_6NO$ $[M+H]^+$: 334.0661, found: 334.0661. IR *v* (neat, cm^{-1}): 3053.7, 2879.5, 1717.3, 1645.7, 1362.4, 1264.3, 1165.0, 1098.1, 907.5, 728.0.

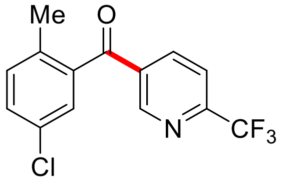

(5-chloro-2-methylphenyl)(6-(trifluoromethyl)pyridin-3-yl)methanone 9

The reaction was carried out according to the general procedure on 0.2 mmol scale (20 h). The crude product was purified by flash chromatography on SiO_2 (eluent: PE:EA = 100:1) to afford **9**, 50.0 mg, 83%, light yellow oil, Rf = 0.3 (PE/EA = 50:1). ^{1}H NMR (500 MHz, Chloroform-*d*) δ 9.06 - 9.04 (m, 1H), 8.30 (dd, *J* = 10.0 Hz, 5.0 Hz, 1H), 7.87 - 7.84 (m, 1H), 7.46 (dd, *J* = 10.0 Hz, 5.0 Hz, 1H), 7.33 - 7.31 (m, 2H), 2.37 (s, 3H). ^{13}C NMR (125 MHz, $CDCl_3$) δ 194.1, 151.3 (q, *J* = 35.0 Hz), 151.2, 138.7, 137.8, 136.1, 134.8, 133.1, 131.62, 128.7, 121.0 (q, *J* = 272.5 Hz), 120.4 (q, *J* = 2.5 Hz), 19.7. ^{19}F NMR (471 MHz, Chloroform-*d*) δ -68.2. HRMS (ESI) Calculated for $C_{14}H_{10}ClF_3NO$ $[M+H]^+$: 300.0398, found: 300.0399. IR *v* (neat, cm^{-1}): 3006.0, 2349.6, 1657.9, 1433.0, 1275.7, 1082.7, 764.1.

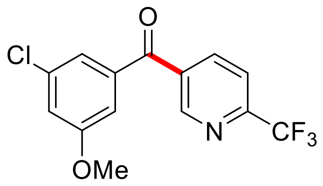

(3-chloro-5-methoxyphenyl)(6-(trifluoromethyl)pyridin-3-yl)methanone 10

The reaction was carried out according to the general procedure on 0.2 mmol scale (36 h). The crude product was purified by flash chromatography on SiO_2 (eluent: PE:EA = 100:1) to afford **10**, 39.1 mg, 62%, light yellow oil, Rf = 0.4 (PE/EA = 20:1). ^{1}H NMR (400 MHz, Chloroform-*d*) δ 9.08 - 9.06 (m, 1H), 8.29 - 8.25 (m, 1H), 7.86 (dd, *J* = 8.0 Hz, *J* = 4.0 Hz, 1H), 7.31 (t, *J* = 4.0 Hz, 1H), 7.25 - 7.23 (m, 1H), 7.20 - 7.18 (m, 1H), 3.88 (s, 3H). ^{13}C NMR (125 MHz, Chloroform-*d*) δ 192.1, 160.6, 151.0 (q, *J* = 35.0 Hz), 138.7, 138.2, 135.7, 135.0, 122.5, 121.1 (q, *J* = 273.0 Hz), 120.0 (q, *J* = 2.5 Hz), 119.7, 113.5, 55.9. ^{19}F NMR (471 MHz, $CDCl_3$) δ -68.2. HRMS (ESI) Calculated for $C_{14}H_{10}ClF_3NO_2$ $[M+H]^+$: 316.0347, found: 316.0348. IR *v* (neat, cm^{-1}): 2960.0, 1717.4, 1636.1, 1576.3, 1404.8, 1262.0, 1230.0, 1089.0, 1043.3, 877.6, 750.7.

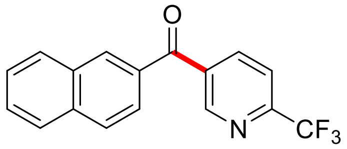

naphthalen-2-yl(6-(trifluoromethyl)pyridin-3-yl)methanone 11

The reaction was carried out according to the general procedure on 0.2 mmol scale (20 h). The crude product was purified by flash chromatography on SiO_2 (eluent: PE:EA = 100:1) to afford **11**, 46.4 mg, 77%, light yellow solid, mp = 75-77 °C, Rf = 0.4 (PE/EA = 20:1). ^{1}H NMR (500 MHz, Chloroform-*d*) δ 9.18 - 7.16 (m, 1H), 8.37 (dd, *J* = 10.0 Hz, 5.0 Hz, 1H), 8.28 (s, 1H), 8.04 - 7.96 (m, 4H), 7.91 (d, *J* = 10.0 Hz, 1H), 7.71 - 7.68 (m, 1H), 7.65 – 7.61 (m, 1H). ^{13}C NMR (125 MHz, Chloroform-*d*) δ 193.5, 150.7, 150.8 (q, *J* = 35.0 Hz) 138.8, 136.0, 135.8, 133.3, 132.6, 132.2, 129.6, 129.2, 129.1, 128.0, 127.4, 125.0, 121.2 (q, *J* = 273.0 Hz), 120.4 (q, *J* = 2.5 Hz). ^{19}F NMR (471 MHz, Chloroform-*d*) δ -68.1. Calculated for $C_{14}H_{10}ClF_3NO_2$ $[M+H]^+$: 302.0787, found: 302.0790. IR *v* (neat, cm^{-1}): 3363.6, 2920.3, 1741.3, 1658.1, 1332.6, 1140.1, 1083.1, 765.5, 725.6, 442.6.

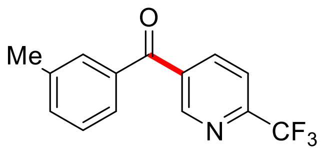

m-tolyl(6-(trifluoromethyl)pyridin-3-yl)methanone 12

The reaction was carried out according to the general procedure on 0.2 mmol scale (20 h). The crude product was purified by flash chromatography on SiO_2 (eluent: PE:EA = 100:1) to afford **12**, 40.8 mg, 77%, light yellow solid, mp = 65-67 ºC, Rf = 0.4 (PE/EA =20:1). ^{1}H NMR (500 MHz, Chloroform-*d*) δ 9.08 - 9.07 (m, 1H), 8.29 (dd, *J* = 10.0 Hz, *J* = 5.0 Hz, 1H), 7.86 (d, *J* = 10.0 Hz, 1H), 7.66 (s, 1H), 7.61 (d, *J* = 10.0 Hz, 1H), 7.50 (d, *J* = 10.0 Hz, 1H), 7.46 - 7.42 (m, 1H), 2.46 (s, 3H). ^{13}C NMR (125 MHz, Chloroform-*d*) δ 193.7, 150.7, 150.4 (q, *J* = 35.0 Hz), 138.9, 138.7, 136.1, 135.8, 134.6, 130.5, 128.7, 127.4, 121.2 (q, *J* = 272.5 Hz), 120.3 (q, *J* = 5.0 Hz), 21.3. ^{19}F NMR (471 MHz, Chloroform-*d*) δ -68.2. HRMS (ESI) Calculated for $C_{14}H_{10}F_4NO$ $[M+H]^+$: 266.0787, found: 266.0793. IR *v* (neat, cm^{-1}): 3063.1, 2923.1, 1665.5, 1596.4, 1331.6, 1287.3, 1139.1, 1082.9, 715.2, 686.9.

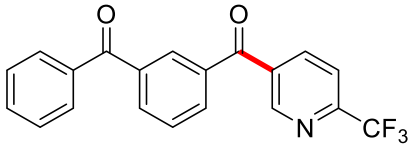

(3-benzoylphenyl)(6-(trifluoromethyl)pyridin-3-yl)methanone 13

The reaction was carried out according to the general procedure on 0.2 mmol scale (20 h). The crude product was purified by flash chromatography on SiO_2 (eluent: PE:EA = 100:1) to afford **13**, 49.7 mg, 70%, light yellow solid, mp = 151-153 ºC, Rf = 0.4 (PE/EA = 20:1). ^{1}H NMR (500 MHz, Chloroform-*d*) δ 9.14 - 9.11 (m, 1H), 8.35 - 8.30 (m, 1H), 8.25 - 8.22 (m, 1H), 8.14 - 8.10 (m, 1H), 8.08 - 8.05 (m, 1H), 7.90 - 7.86 (m, 1H), 7.86 - 7.82 (m, 2H), 7.72 (t, *J* = 10.0 Hz, 1H), 7.68 - 7.63 (m, 1H), 7.57 - 7.51 (m, 2H). ^{13}C NMR (125 MHz, Chloroform-*d*) δ 195.3, 192.8, 151.9 (q, *J* = 35.0 Hz), 150.7, 138.7, 138.4, 136.7, 136.2, 135.0, 134.7, 133.3, 133.2, 131.1, 130.1, 129.1, 128.6, 121.6 (q, *J* = 273.0 Hz), 120.4 (q, *J* = 2.5 Hz). ^{19}F NMR (376 MHz, Chloroform-*d*) δ -68.2. HRMS (ESI) Calculated for $C_{20}H_{13}F_3NO_2$ $[M+H]^+$: 356.0893, found: 356.0897. IR *v*

(neat, cm^{-1}): 3065.2, 2921.6, 1662.1, 1578.7, 1483.6, 1332.0, 1258.1, 1083.3, 958.6, 914.1, 714.2, 610.3.

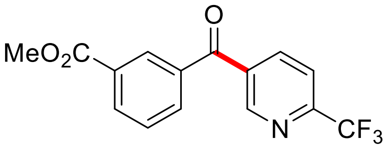

methyl 3-(6-(trifluoromethyl)nicotinoyl)benzoate 14

The reaction was carried out according to the general procedure on 0.2 mmol scale (36 h). The crude product was purified by flash chromatography on SiO_2 (eluent: PE:EA = 100:1) to afford **14**, 46.0 mg, 76%, light yellow oil, Rf = 0.2 (PE/EA = 20:1). ^{1}H NMR (500 MHz, Chloroform-*d*) δ 9.10 - 9.08 (m, 1H), 8.46 - 8.43 (m, 1H), 8.37 - 8.33 (m, 1H), 8.31 (dd, *J* = 10.0, 5.0 Hz, 1H), 8.06 (dt, *J* = 10.0, 5.0 Hz, 1H), 7.90 - 7.85 (m, 1H), 7.67 (t, *J* = 10.0 Hz, 1H), 3.97 (s, 3H). ^{13}C NMR (100 MHz, Chloroform-*d*) δ 192.7, 165.8, 150.8 (q, *J* = 34.0 Hz), 138.7, 136.3, 135.0, 134.5, 133.9, 133.6, 130.9, 129.2, 120.2 (q, *J* = 2.0 Hz), 120.1 (q, *J* = 273.0 Hz), 52.6. ^{19}F NMR (376 MHz, Chloroform-*d*) δ -68.2. HRMS (ESI) Calculated for $C_{15}H_{11}F_3NO_3$ $[M+H]^+$: 310.0686, found: 310.0692. IR *v* (neat, cm^{-1}): 3394.5, 2985.5, 2877.4, 2349.2, 1772.3, 1717.3, 1645.7, 1398.6, 1296.2, 1165.1, 1067.3, 892.4, 764.9, 665.2.

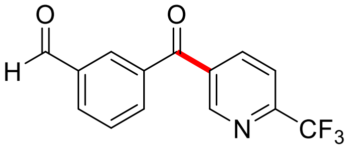

3-(6-(trifluoromethyl)nicotinoyl)benzaldehyde 15

The reaction was carried out according to the general procedure on 0.2 mmol scale (36 h). The crude product was purified by flash chromatography on SiO_2 (eluent: PE:EA = 100:1) to afford **15**, 37.0 mg, 66%, light yellow oil, Rf = 0.3 (PE/EA =10:1). ^{1}H NMR (400 MHz, Chloroform-*d*) δ 10.04 (s, 1H), 9.04 - 9.00 (m, 1H), 8.25 - 8.22 (m, 2H), 8.13 (dt, *J* = 8.0, 4.0 Hz, 1H), 8.04 (d, *J* = 8.0, 4.0 Hz, 1H), 7.83 - 7.79 (m, 1H), 7.69 (t, *J* = 8.0 Hz, 1H). ^{13}C NMR (100 MHz, Chloroform-*d*) δ 192.4, 190.9, 150.7, 150.3 (q, *J* = 35.0 Hz), 138.7, 136.9, 136.8, 135.2, 134.9, 134.1, 130.9, 129.9, 121.1 (q, *J* = 273.0 Hz), 120.5 (q, *J* = 3.0 Hz). ^{19}F NMR (376 MHz, Chloroform-*d*) δ -68.2. HRMS (ESI)

Calculated for $C_{14}H_9F_3NO_2$ $[M+H]^+$: 280.0580, found: 280.0581. IR v (neat, cm^{-1}): 2925.1, 1702.1, 1668.8, 1598.4, 1331.9, 1132.2, 1082.7, 937.4, 859.7, 731.7.

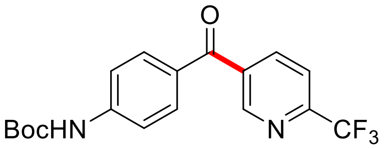

tert-butyl (4-(6-(trifluoromethyl)nicotinoyl)phenyl)carbamate 16

The reaction was carried out according to the general procedure on 0.2 mmol scale (20 h). The crude product was purified by flash chromatography on SiO_2 (eluent: PE:EA = 100:1) to afford **16**, 60.0 mg, 82%, light yellow solid, mp = 108-110 ℃, Rf = 0.3 (PE/EA = 5:1). ^1H NMR (400 MHz, Chloroform-*d*) δ 8.97 - 8.94 (m, 1H), 8.17- 8.13 (m, 1H), 7.78 - 7.70 (m, 3H), 7.49 - 7.44 (m, 2H), 6.91 (s, 1H), 1.46 (s, 9H). ^{13}C NMR (100 MHz, Chloroform-*d*) δ 192.1, 152.1, 150.4, 150.3 (q, J= 35.0 Hz), 143.9, 138.5, 136.2, 131.8, 131.3, 130.2, 121.5 (q, J= 273.0 Hz), 120.0 (q, J= 2.0 Hz), 117.6, 81.6, 28.2. ^{19}FNMR (376 MHz, Chloroform-*d*) δ -68.1. HRMS (ESI) Calculated for $C_{18}H_{18}F_3N_2O_3$ $[M+H]^+$: 367.1264, found: 367.1272. IR v (neat, cm^{-1}): 3396.0, 2985.5, 2879.6, 1724.7, 1644.5, 1527.2, 1400.0, 1366.5, 1232.1, 1164.8, 1097.8, 933.3, 890.4, 764.4.

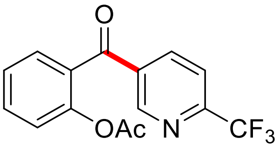

2-(6-(trifluoromethyl)nicotinoyl)phenyl acetate 17

The reaction was carried out according to the general procedure on 0.2 mmol scale (36 h). The crude product was purified by flash chromatography on SiO_2 (eluent: PE:EA = 100:1) to afford **17**, 38.3 mg, 62%, yellow oil, Rf = 0.3 (PE/EA = 10:1). ^1H NMR (400 MHz, Chloroform-*d*) δ 9.02 - 8.98 (s, 1H), 8.19 - 8.14 (m, 1H), 7.73 (dd, J = 8.0, 4.0 Hz, 1H), 7.60 - 7.54 (m, 1H), 7.44 (dd, J = 8.0, 4.0 Hz, 1H), 7.34 - 7.28 (m, 1H), 7.19 - 7.16 (m, 1H), 1.99 (s, 3H). ^{13}C NMR (100 MHz, Chloroform-*d*) δ 191.9, 169.1, 150.9 (q, J= 35.0 Hz), 151.0, 148.9, 138.6, 135.2, 133.7, 130.5, 13.0, 126.0, 123.5, 121.1 (q, J= 273.0 Hz), 120.2 (q, J= 3.0 Hz), 20.7. ^{19}F NMR (376 MHz, Chloroform-*d*) δ -68.2. HRMS (ESI) Calculated for $C_{15}H_{11}F_3NO_3$ $[M+H]^+$: 310.0686, found: 310.0683. IR v

(neat, cm^{-1}): 3056.6, 2920.6, 2349.2, 1767.4, 1674.3, 1604.2, 1333.0, 1275.1, 1082.8, 909.0, 766.9.

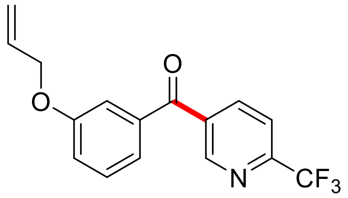

(3-(allyloxy)phenyl)(6-(trifluoromethyl)pyridin-3-yl)methanone 18

The reaction was carried out according to the general procedure on 0.2 mmol scale (20 h). The crude product was purified by flash chromatography on SiO_2 (eluent: PE:EA = 100:1) to afford **18**, 47.3 mg, 77%, yellow oil, Rf = 0.4 (PE/EA = 20:1). ^{1}H NMR (400 MHz, Chloroform-*d*) δ 9.00 - 8.98 (m, 1H), 8.21 - 8.17 (m, 1H), 7.76 (d, *J* = 8.0, 4.0 Hz, 1H), 7.38 - 7.29 (m, 1H), 7.27 - 7.23 (m, 1H), 7.17 - 7.12 (m, 1H), 6.03 - 5.92 (m, 2H), 5.39 - 5.32 (m, 1H), 5.27 - 5.22 (m, 1H), 4.53 (dt, *J* = 8.0, 4.0 Hz, 2H). ^{13}C NMR (100 MHz, Chloroform-*d*) δ 193.2, 158.9, 150.7 (q, *J* = 35.0 Hz), 150.7 138.6, 137.2, 135.6, 132.6, 129.8, 123.0, 121.1 (q, *J* = 273.0 Hz), 120.9, 120.2 (q, *J* = 3.0 Hz), 118.2, 115.2, 69.1. ^{19}F NMR (376 MHz, Chloroform-*d*) δ -68.2. HRMS (ESI) Calculated for $C_{16}H_{13}F_3NO_2$ $[M+H]^+$: 308.0893, found: 308.0896. IR *v* (neat, cm^{-1}): 2922.1, 1666.2, 1580.2, 1383.2, 1331.7, 1282.7, 1141.8, 1083.3, 960.8, 847.9, 783.2, 737.1.

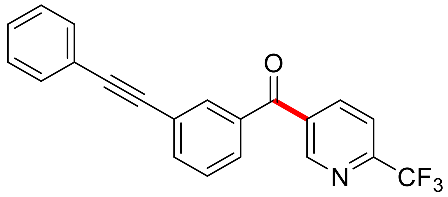

(3-(phenylethynyl)phenyl)(6-(trifluoromethyl)pyridin-3-yl)methanone 19

The reaction was carried out according to the general procedure on 0.2 mmol scale (20 h). The crude product was purified by flash chromatography on SiO_2 (eluent: PE:EA = 100:1) to afford **19**, 45.6 mg, 65%, light yellow solid, mp = 139-141 °C, Rf= 0.5 (PE/EA = 20:1). ^{1}H NMR (400 MHz, Chloroform-*d*) δ 9.04 - 9.01 (m, 1H), 8.23 (dd, *J* = 8.0, 4.0 Hz, 1H), 7.87 (t, *J* = 4.0 Hz, 1H), 7.81 - 7.77 (m, 1H), 7.76 - 7.70 (m, 2H), 7.49 - 7.45 (m, 3H), 7.30 - 7.28 (m, 3H). ^{13}C NMR (100 MHz, Chloroform-*d*) δ 192.8, 150.7, 150.8 (q, *J* = 35.0 Hz), 138.71, 136.5, 136.2, 135.2, 133.0, 131.7, 129.4, 129.1, 128.8, 128.5, 124.4, 122.5, 120.4 (q, *J* = 2.0 Hz), 91.2, 121.1 (q, *J* = 273.0 Hz), 87.7. ^{19}F NMR

(376 MHz, Chloroform-*d*) δ -68.2. HRMS (ESI) Calculated for $C_{21}H_{13}F_3NO$ $[M+H]^+$: 352.0944, found: 352.0948. IR *v* (neat, cm^{-1}): 3065.4, 2923.2, 1657.1, 1573.9, 1443.6, 1330.1, 1263.6, 1130.1, 1082.9, 981.0, 861.6, 853.9, 755.0, 733.0, 691.2, 537.9.

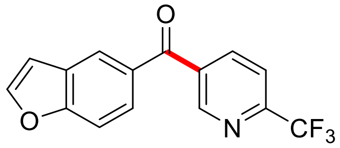

benzofuran-5-yl(6-(trifluoromethyl)pyridin-3-yl)methanone 20

The reaction was carried out according to the general procedure on 0.2 mmol scale (36 h). The crude product was purified by flash chromatography on SiO_2 (eluent: PE:EA = 100:1) to afford **20**, 47.0 mg, 81%, light yellow solid, mp = 121-123 ºC, Rf = 0.4 (PE/EA =20:1). ^{1}H NMR (400 MHz, Chloroform-*d*) δ 9.02 - 8.98 (m, 1H), 8.23 - 8.19 (m, 1H), 8.01 (d, *J* = 4.0 Hz, 1H), 7.79 - 7.76 (m, 2H), 7.68 (dd, *J* = 8.0, 4.0 Hz, 1H), 7.59 - 7.53 (m, 1H), 6.81 (dd, *J* = 8.0, 4.0 Hz, 1H). ^{13}C NMR (100 MHz, Chloroform-*d*) δ 193.2, 157.8, 150.6, 150.0 (q, *J* = 35.0 Hz), 147.0, 138.6, 136.3, 131.4, 127.8, 126.6, 124.7, 121.2 (q, *J* = 273.0 Hz), 120.3 (q, *J* = 3.0 Hz), 112.0, 107.3. ^{19}F NMR (376 MHz, Chloroform-*d*) δ -68.1. HRMS (ESI) Calculated for $C_{15}H_9F_3NO_2$ $[M+H]^+$: 292.0580, found: 292.0584. IR *v* (neat, cm^{-1}): 3053.3, 2253.6, 2253.6, 1663.8, 1422.3, 1330.5, 1264.4, 1083.3, 906.5, 726.6.

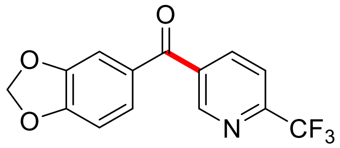

benzo[d][1,3]dioxol-5-yl(6-(trifluoromethyl)pyridin-3-yl)methanone 21

The reaction was carried out according to the general procedure on 0.2 mmol scale (20 h). The crude product was purified by flash chromatography on SiO_2 (eluent: PE:EA = 100:1) to afford **21**, 47.8 mg, 81%, light yellow solid, mp = 75-77 ºC, Rf = 0.4 (PE/EA = 20:1). ^{1}H NMR (400 MHz, Chloroform-*d*) δ 8.99 - 8.92 (m, 1H), 8.17 - 8.12 (m, 1H), 7.76 (dd, *J* = 8.0, 4.0 Hz, 1H), 7.32 (d, *J* = 4.0 Hz, 1H), 7.27 (dd, *J* = 8.0, 4.0 Hz, 1H), 6.83 (d, *J* = 8.0 Hz, 1H), 6.04 (s, 2H). ^{13}C NMR (100 MHz, Chloroform-*d*) δ 191.7, 152.7, 150.3, 150.2 (q, *J* = 35.0 Hz), 148.6, 148.5, 138.4, 136.3, 136.1, 130.7, 127.4, 121.2 (q, *J* = 273.0 Hz), 120.2 (q, *J* = 2.0 Hz), 109.4, 108.1, 102.3. ^{19}F NMR (376 MHz,

Chloroform-*d*) δ -68.2. HRMS (ESI) Calculated for $C_{14}H_9F_3NO_3$ [M+H]$^+$: 296.0529, found: 296.0536. IR *v* (neat, cm^{-1}): 3054.2, 2307.8, 1653.0, 1443.4, 1264.1, 932.1, 730.8.

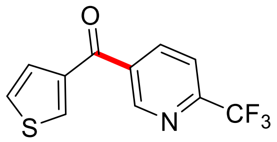

thiophen-3-yl(6-(trifluoromethyl)pyridin-3-yl)methanone 22

The reaction was carried out according to the general procedure on 0.2 mmol scale (36 h). The crude product was purified by flash chromatography on SiO_2 (eluent: PE:EA=100:1) to afford **22**, 42.1 mg, 82%, light yellow solid, mp = 109-111 °C, Rf = 0.5 (PE/EA =20:1). ^{1}H NMR (400 MHz, Chloroform-*d*) δ 9.07 - 9.04 (m, 1H), 8.25 - 8.21 (m, 1H), 7.93 - 7.90 (m, 1H), 7.80 - 7.75 (m, 1H), 7.55 (dd, *J* = 8.0, 4.0 Hz, 1H), 7.41 - 7.37 (m, 1H). ^{13}C NMR (100 MHz, Chloroform-*d*) δ 186.6, 150.6 (q, *J* = 35.0 Hz), 150.1, 140.3, 138.1, 136.6, 135.2, 128.1, 127.4, 121.1 (q, *J* = 273.0 Hz), 120.4 (q, *J* = 2.0 Hz). HRMS (ESI) Calculated for $C_{11}H_7F_3NOS$ [M+H]$^+$: 258.0195, found: 258.0195. IR *v* (neat, cm^{-1}): 3054.2, 2349.4, 1656.9, 1421.5, 1264.2, 1084.1, 908.2, 728.9.

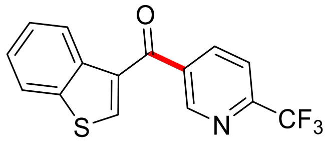

benzo[b]thiophen-3-yl(6-(trifluoromethyl)pyridin-3-yl)methanone 23

The reaction was carried out according to the general procedure on 0.2 mmol scale (20 h). The crude product was purified by flash chromatography on SiO_2 (eluent: PE:EA = 100:1) to afford **23**, 53.0 mg, 86%, light yellow solid, mp = 113-115 °C, Rf = 0.5 (PE/EA = 20:1). ^{1}H NMR (400 MHz, Chloroform-*d*) δ 9.06 - 9.02 (m, 1H), 8.57 - 8.53 (m, 1H), 8.22 (dd, *J* = 8.0, 4.0 Hz, 1H), 7.95 (s, 1H), 7.86 - 7.82 (m, 1H), 7.77 (d, *J* = 8.0 Hz, 1H), 7.49 - 7.39 (m, H). ^{13}C NMR (100 MHz, Chloroform-*d*) δ 187.3, 150.1, 149.8 (q, *J* = 35.0 Hz), 140.3, 140.1, 138.2, 137.3, 136.8, 134.0, 126.3, 126.2, 125.2, 122.49 120.7 (q, *J* = 272.0 Hz), 120.3 (q, *J* = 3.0 Hz). ^{19}F NMR (376 MHz, Chloroform-*d*) δ -68.1.

HRMS (ESI) Calculated for $C_{15}H_9F_3NOS$ [M+H]$^+$: 308.0351, found: 308.0356. IR v (neat, cm^{-1}): 3053.9, 1649.8, 1422.5, 1264.3, 907.4, 727.8.

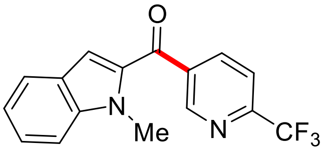

(1-methyl-1H-indol-2-yl)(6-(trifluoromethyl)pyridin-3-yl)methanone 24

The reaction was carried out according to the general procedure on 0.2 mmol scale (20 h). The crude product was purified by flash chromatography on SiO_2 (eluent: PE:EA = 100:1) to afford **24**, 43.2 mg, 71%, light yellow solid, mp = 85-87 °C, Rf = 0.4 (PE/EA =20:1). ^{1}H NMR (500 MHz, $CDCl_3$) δ 9.22 - 9.20 (m, 1H), 8.37 (dd, J = 10.0, 5.0 Hz, 1H), 7.87 (d, J = 10.0 Hz,1H), 7.72 (d, J = 10.0 Hz, 1H), 7.50 - 7.47 (m, 1H), 7.25 - 7.21 (m, 2H), 7.05 (s, 1H), 4.19 (s, 3H). ^{13}C NMR (100 MHz, Chloroform-d) δ 185.0, 150.3, 150.2 (q, J = 35.0 Hz), 140.9, 138.3, 137.5, 133.8, 127.2, 125.8, 123.4, 121.4, 120.3 (q, J = 2.0 Hz), 118.5 (q, J = 273.0 Hz), 116.4, 110.5, 32.2. ^{19}F NMR (376 MHz, Chloroform-d) δ -68.1. HRMS (ESI) Calculated for $C_{16}H_{12}F_3N_2O$ [M+H]$^+$: 305.0896, found: 305.0897. IR v (neat, cm^{-1}): 3359.4, 2920.1, 2349.2, 1637.8, 1510.3, 1331.5, 1140.7, 1082.7, 939.7, 741.0, 510.3.

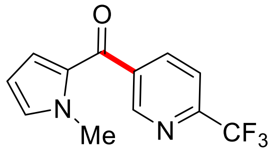

(1-methyl-1H-pyrrol-2-yl)(6-(trifluoromethyl)pyridin-3-yl)methanone 25

The reaction was carried out according to the general procedure on 0.2 mmol scale (20 h). The crude product was purified by flash chromatography on SiO_2 (eluent: PE:EA = 100:1) to afford **25**, 68%, 34.5 mg, light yellow solid, mp = 64-66 °C, Rf = 0.4 (PE/EA = 20:1). ^{1}H NMR (400 MHz, Chloroform-d) δ 9.03 - 8.89 (m, 1H), 8.18 - 8.14 (m, 1H), 7.73 (dd, J = 8.0, 4.0 Hz, 1H), 6.94 (t, J = 4.0 Hz, 1H), 6.66 - 6.63 (m, 1H), 6.15 - 6.13 (m, 1H), 3.99 (s, 3H). ^{13}C NMR (100 MHz, Chloroform-d) δ 182.0, 149.9, 149.7 (q, J = 35.0 Hz), 137.8, 133.1, 129.8, 123.9, 121.3 (q, J = 273.0 Hz), 120.1 (q, J = 3.0 Hz), 109.2, 37.6. ^{19}F NMR (376 MHz, Chloroform-d) δ -68.1. HRMS (ESI) Calculated for

$C_{12}H_{10}F_3N_2O$ [M+H]$^+$: 255.0740, found: 255.0747. IR v (neat, cm^{-1}): 3110.6, 2928.1, 1626.4, 1428.4, 1405.5, 1329.2, 1139.6, 915.6, 736.5.

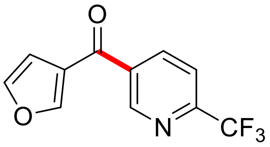

benzo[b]thiophen-3-yl(6-(trifluoromethyl)pyridin-3-yl)methanone 26

The reaction was carried out according to the general procedure on 0.2 mmol scale (36 h). The crude product was purified by flash chromatography on SiO_2 (eluent: PE:EA = 100:1) to afford **26**, 57%, 27.5 mg, light yellow solid, mp = 93-95 °C, Rf = 0.3 (PE/EA = 20:1). ^{1}H NMR (400 MHz, Chloroform-*d*) δ 9.09 - 9.05 (m, 1H), 8.26 - 8.22 (m, 1H), 7.91 (t, *J* = 4.0 Hz, 1H), 7.79 - 7.76 (m, 1H), 7.50 (t, *J* = 4.0 Hz, 1H), 6.87 - 6.84 (s, 1H). ^{13}C NMR (100 MHz, Chloroform-*d*) δ 186.1, 150.4 (q, *J* = 35.0 Hz) 149.6, 149.1, 144.8, 137.6, 136.5, 122.1 (q, *J* = 273.0 Hz), 120.7 (q, *J* = 3.0 Hz), 109.7. ^{19}F NMR (376 MHz, Chloroform-*d*) δ -68.2. HRMS (ESI) Calculated for $C_{11}H_7F_3NO_2$ [M+H]$^+$: 242.0423, found: 242.0425. IR v (neat, cm^{-1}): 3361.8, 3100.4, 2920.9, 1646.1, 1514.7, 1333.0, 1183.2, 1145.0, 887.2, 739.5, 587.2.

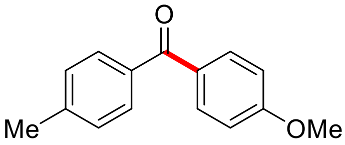

(4-methoxyphenyl)(p-tolyl)methanone 27

The reaction was carried out according to the general procedure on 0.2 mmol scale (20 h). The crude product was purified by flash chromatography on SiO_2 (eluent: PE:EA = 100:1) to afford **27**, 30.7 mg, 68%, light yellow solid, Rf = 0.4 (PE/EA = 10:1). ^{1}H NMR (400 MHz, Chloroform-*d*) δ 7.73 (d, *J* = 8.9 Hz, 2H), 7.59 (d, *J* = 8.1 Hz, 2H), 7.18 (d, *J* = 7.9 Hz, 2H), 6.87 (d, *J* = 8.9 Hz, 2H), 3.79 (s, 3H), 2.35 (s, 3H). ^{13}C NMR (100 MHz, Chloroform-*d*) δ 195.4, 163.0, 142.6, 135.5, 132.4, 130.5, 130.0, 128.9, 113.5, 55.5, 21.6. These data are in agreement with those reported previously in the literature.[3]

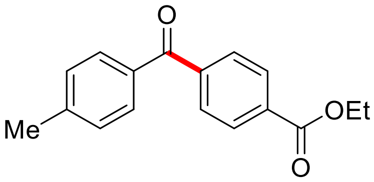

ethyl 4-(4-methylbenzoyl)benzoate 28

The reaction was carried out according to the general procedure on 0.2 mmol scale (20 h). The crude product was purified by flash chromatography on SiO_2 (eluent: PE:EA = 100:1) to afford **28**, 45.0 mg, 84%, white solid, Rf = 0.2 (PE/EA = 20:1). ^1H NMR (400 MHz, $CDCl_3$) δ 8.15 (d, *J* = 8.0 Hz, 2 H), 7.81 (d, *J* = 8.0 Hz, 2H), 7.72 (d, *J* = 8.0 Hz, 2H), 7.29 (d, *J* = 8.0 Hz, 2H), 4.42 (q, *J* = 8.0 Hz, 2H), 2.45 (s, 3H), 1.44 (t, *J* = 8.0 Hz, 3H). ^{13}C NMR (100 MHz, $CDCl_3$) δ 195.8, 165.9, 143.9, 141.6, 134.3, 133.4, 131.1, 130.3, 129.6, 129.4, 129.2, 121.7, 61.4, 21.7, 14.3. These data are in agreement with those reported previously in the literature.[2]

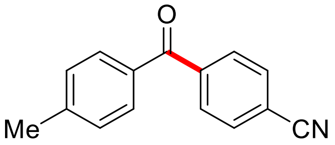

4-(4-methylbenzoyl)benzonitrile 29

The reaction was carried out according to the general procedure on 0.2 mmol scale (20 h). The crude product was purified by flash chromatography on SiO_2 (eluent: PE:EA = 100:1) to afford **29**, 38.5 mg, 87%, white solid, Rf = 0.3 (PE/EA = 5:1). ^1H NMR (500 MHz, Chloroform-*d*) δ 7.87 (d, *J* = 8.0 Hz, 2H), 7.80 (d, *J* = 8.0 Hz, 2H), 7.72 (d, *J* = 8.0 Hz, 2H), 7.33 (d, *J* = 8.0 Hz, 2H), 2.48 (s, 3H). ^{13}C NMR (125 MHz, Chloroform-*d*) δ 194.8, 144.4, 141.7, 133.7, 132.1, 130.3, 130.1, 129.4, 118.1, 115.41, 21.8. These data are in agreement with those reported previously in the literature.[3]

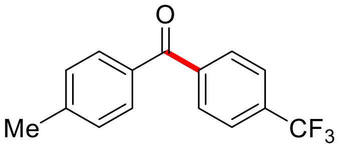

p-tolyl(4-(trifluoromethyl)phenyl)methanone 30

The reaction was carried out according to the general procedure on 0.2 mmol scale (20 h). The crude product was purified by flash chromatography on SiO_2 (eluent: PE:EA = 100:1) to afford **30**, 42.8 mg, 81%, white solid, Rf = 0.4 (PE/EA = 20:1). ^1H NMR (400

MHz, $CDCl_3$) δ 7.87 (d, J = 8.0 Hz, 2H), 7.75 - 7.71 (m, 4H), 7.30 (d, J = 8.0 Hz, 2H), 2.45 (s, 3H). ^{13}C NMR (100 MHz, Chloroform-*d*) δ 195.3, 144.1, 141.1, 133.4 (q, J = 32.0 Hz), 134.1, 130.3, 130.0, 129.2, 125.6 (q, J = 271.0 Hz), 125.3 (q, J = 3.0 Hz). ^{19}F NMR (376 MHz, Chloroform-*d*) δ -63.0. These data are in agreement with those reported previously in the literature.[4]

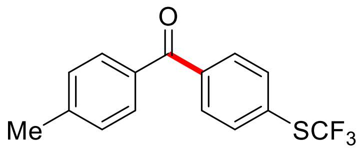

p-tolyl(4-((trifluoromethyl)thio)phenyl)methanone 31

The reaction was carried out according to the general procedure on 0.2 mmol scale (20 h). The crude product was purified by flash chromatography on SiO_2 (eluent: PE:EA = 100:1) to afford **31**, 47.3 mg, 80%, white solid, mp = 121-123 °C, Rf = 0.4 (PE/EA = 20:1). ^{1}H NMR (400 MHz, Chloroform-*d*) δ 7.81 (d, J = 8.0 Hz, 2 H), 7.75 (d, J = 8.0 Hz, 2H), 7.72 (d, J = 8.0 Hz, 2H), 7.30 (d, J = 8.0 Hz, 2H), 2.45 (s, 3H). ^{13}C NMR (100 MHz, $CDCl_3$) δ 195.3, 144.0, 139.9, 135.5, 134.1, 130.6, 130.3, 129.2, 129.4 (q, J = 308.0 Hz), 21.7. ^{19}F NMR (376 MHz, Chloroform-*d*) δ -41.8. HRMS (ESI) Calculated for $C_{15}H_{12}F_3OS$ $[M+H]^+$: 297.0555, found: 297.0562. IR v (neat, cm^{-1}): 2253.4, 1657.1, 1607.3, 1395.5, 1280.1, 1083.1, 902.7, 722.5, 649.5.

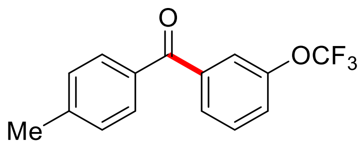

p-tolyl(3-(trifluoromethoxy)phenyl)methanone 32

The reaction was carried out according to the general procedure on 0.2 mmol scale (20 h). The crude product was purified by flash chromatography on SiO_2 (eluent: PE:EA = 100:1) to afford **32**. 26.9 mg, 48%, light yellow solid, mp = 117-119 °C, Rf = 0.5 (PE/EA =20:1). ^{1}H NMR (400 MHz, Chloroform-*d*) δ 7.71 (d, J = 8.0 Hz, 3H), 7.65 - 7.62 (m, 1H), 7.52 (d, J = 8.0 Hz, 1H), 7.45 - 7.41 (m, 1H), 7.30 (d, J = 8.0 Hz, 2H), 2.45 (s, 1H). ^{13}C NMR (100 MHz, $CDCl_3$) δ 194.7, 149.1, 143.9, 139.8, 134.1, 130.3, 129.8, 129.2, 128.2, 124.5, 122.23, 120.4 (q, J = 256.0 Hz), 21.7. ^{19}F NMR (400 MHz, $CDCl_3$)

δ -57.9. HRMS (ESI) Calculated for $C_{15}H_{12}F_3O_2$ $[M+H]^+$: 281.0784, found: 281.0785. IR ν (neat, cm^{-1}): 3054.1, 2253.2, 1648.1, 1601.8, 1421.3, 1264.2, 906.9, 728.2.

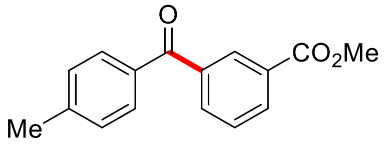

methyl 3-(4-methylbenzoyl)benzoate 33

The reaction was carried out according to the general procedure on 0.2 mmol scale (20 h).the crude product was purified by flash chromatography on SiO_2 (eluent: PE:EA = 100:1) to afford **33**, 41.1 mg, 81%, light yellow solid, mp = 115-117 °C, Rf = 0.3 (PE/EA = 20:1). ^{1}H NMR (400 MHz, $CDCl_3$) δ 8.42 (t, J = 4.0 Hz, 1H), 8.25 (dt, J = 8.0, 4.0 Hz, 1H), 7.99 (dt, J = 8.0, 4.0 Hz, 1H), 7.72 (d, J = 8.0 Hz, 2H), 7.58 (t, J = 8.0 Hz, 1H), 7.30 (d, J = 8.0 Hz, 2H), 3.94 (s, 3H), 2.46 (s, 3H). ^{13}C NMR (100 MHz, $CDCl_3$) δ 195.5, 166.4, 143.7, 138.3, 134.4, 134.0, 133.0, 130.9, 130.3, 129.2, 128.6, 52.4, 21.7. HRMS (ESI) Calculated for $C_{16}H_{15}O_3$ $[M+H]^+$: 255.1016, found: 255.1019. IR ν (neat, cm^{-1}): 2982.5, 1718.8, 1659.0, 1605.9, 1446.5, 1267.1, 1103.2, 1020.4, 907.1, 736.7.

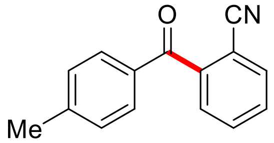

2-(4-methylbenzoyl)benzonitrile 34

The reaction was carried out according to the general procedure on 0.2 mmol scale (20 h). The crude product was purified by flash chromatography on SiO_2 (eluent: PE:EA = 100:1) to afford **34**, 35.0 mg, 79%, light yellow oil, Rf = 0.2 (PE/EA = 20:1). ^{1}H NMR (400 MHz, Chloroform-*d*) δ 7.85 -7.81 (m, 1H), 7.72 (d, J = 8.0 Hz, 2H), 7.70 - 7.66 (m, 1H), 7.65 - 7.62 (m, 1H), 7.30 (d, J = 8.0 Hz, 2H), 2.45 (s, 3H). ^{13}C NMR (100 MHz, Chloroform-*d*) δ 193.5, 145.1, 142.0, 134.1, 133.4, 132.1, 131.1, 130.5, 129.9, 129.4, 117.1, 111.9, 21.8. HRMS (ESI) Calculated for $C_{15}H_{12}NO$ $[M+H]^+$: 222.0913, found: 222.0918. IR ν (neat, cm^{-1}): 2921.7, 2228.0, 1657.9, 1603.1, 1409.0, 1292.6, 1184.1, 931.0, 837.3, 760.0, 670.3, 584.0.

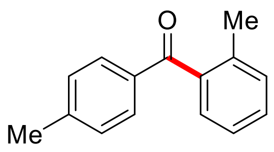

***o-tolyl(p-tolyl)methanone* 35**

The reaction was carried out according to the general procedure on 0.2 mmol scale (20 h). The crude product was purified by flash chromatography on SiO_2 (eluent: PE:EA = 10 The reaction was carried out according to the general procedure on 0.2 mmol scale (20 h). The crude product was purified by flash chromatography on SiO_2 (eluent: PE:EA = 100:1) to afford **35**, 25.8 mg, 61%, light yellow oil, Rf = 0.2 (PE/EA = 20:1). ^{1}H NMR (400 MHz, Chloroform-*d*) δ 7.62 (d, *J* = 8.0 Hz, 2H), 7.32 - 7.27 (m, 1H), 7.25 - 7.10 (m, 5H), 2.34 (s, 3H), 2.23 (s, 3H). ^{13}C NMR (100 MHz, Chloroform-*d*) δ 198.4, 144.1, 139.0, 136.5, 135.2, 130.9, 130.3, 130.0, 129.2, 128.3, 125.2, 21.7, 19.9. These data are in agreement with those reported previously in the literature.[2]

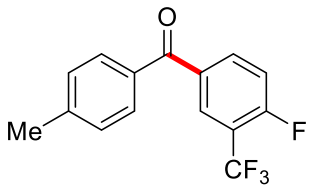

(4-fluoro-3-(trifluoromethyl)phenyl)(p-tolyl)methanone 36

The reaction was carried out according to the general procedure on 0.2 mmol scale (20 h). The crude product was purified by flash chromatography on SiO_2 (eluent: PE:EA = 100:1) to afford **36**, 43.4 mg, 77%, white solid, mp = 120-122 °C, Rf = 0.3 (PE/EA = 20:1). ^{1}H NMR (400 MHz, Chloroform-*d*) δ 8.11 - 8.06 (m, 1H), 8.03 - 7.99 (m, 1H), 7.68 (d, *J* = 8.0 Hz, 2H), 7.34 - 7.28 (m, 3H), 2.46 (s, 3H). ^{13}C NMR (100 MHz, Chloroform-*d*) δ 193.6, 161.9 (d, *J* = 264.0 Hz), 144.1, 135.8 (d, *J* = 10.0 Hz), 134.3, 133.9, 130.1, 129.3, 122.1 (d, *J* = 272.0 Hz), 118.7 (q, *J* = 20.0 Hz), 117.0 (d, *J* = 21.0 Hz), 21.7. ^{19}F NMR (376 MHz, Chloroform-*d*) δ -61.6, -108.2. HRMS (ESI) Calculated for $C_{15}H_{11}F_4O$ $[M+H]^+$: 283.0741, found: 283.0742. IR *v* (neat, cm^{-1}): 3362.5, 2851.1, 2349.2, 1651.2, 1470.6, 1274.4, 1133.6, 964.5, 725.6.

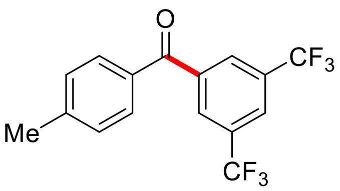

(3,5-bis(trifluoromethyl)phenyl)(p-tolyl)methanone 37

The reaction was carried out according to the general procedure on 0.2 mmol scale (20 h). The crude product was purified by flash chromatography on SiO_2 (eluent: PE:EA = 100:1) to afford **37**, 43.8 mg, 66%, light yellow liquid, Rf = 0.4 (PE/EA = 10:1). ^1H NMR (400 MHz, $CDCl_3$) δ 8.15 - 8.12 (m, 2H), 8.02 - 7.98 (m, 1H), 7.62 (d, J = 8.0 Hz, 2H), 7.27 (d, J = 8.0 Hz, 2H), 2.40 (s, 3H). ^{13}C NMR (100 MHz, $CDCl_3$) δ 193.3, 144.8, 139.6, 133.3, 132.0 (q, J = 34.0 Hz), 130.2, 129.7, 129.6, 125.4 (q, J = 3.0 Hz), 122.9 (q, J = 272.0 Hz), 21.6. These data are in agreement with those reported previously in the literature.[5]

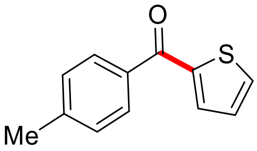

thiophen-2-yl(p-tolyl)methanone 38

The reaction was carried out according to the general procedure on 0.2 mmol scale (20 h). The crude product was purified by flash chromatography on SiO_2 (eluent: PE:EA = 100:1) to afford **38**. 28.7 mg, 71%, light yellow solid, Rf = 0.4 (PE/EA = 10:1). ^1H NMR (400 MHz, $CDCl_3$) δ 7.71 (d, J = 8.0 Hz, 2H), 7.62 (dd, J = 8.0, 4.0 Hz, 1H), 7.57 (dd, J = 8.0 Hz, 1H), 7.22 (d, J = 8.0 Hz, 2H), 7.09 - 7.07 (m, 1H), 2.36 (s, 3H). ^{13}C NMR (100 MHz, $CDCl_3$) δ 187.9, 143.8, 143.1, 135.4, 134.5, 133.8, 129.4, 129.1, 127.9, 21.7. These data are in agreement with those reported previously in the literature.[5]

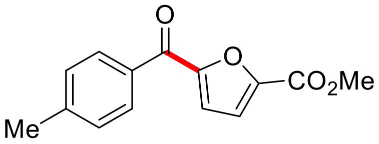

methyl 5-(4-methylbenzoyl)furan-2-carboxylate 39

The reaction was carried out according to the general procedure on 0.2 mmol scale (20 h). The crude product was purified by flash chromatography on SiO_2 (eluent: PE:EA = 20:1) to afford **39**, 27.8 mg, 57%, white solid, mp = 118-120 ºC, Rf = 0.3 (PE/EA =

5:1). ^{1}H NMR (400 MHz, $CDCl_3$) δ 7.93 (d, J = 8.0 Hz, 2H), 7.26 (d, J = 8.0 Hz, 2H), 7.21 (s, 2H), 3.88 (s, 3H), 2.38 (s, 3H). ^{13}C NMR (100 MHz, $CDCl_3$) δ 181.9, 158.7, 154.2, 146.6, 144.3, 133.6, 129.9, 129.4, 119.6, 118.5, 52.4, 21.8. HRMS (ESI) Calculated for $C_{14}H_{13}O_4$ $[M+H]^+$: 245.0808, found: 245.0814. IR v (neat, cm^{-1}): 3361.0, 3149.5, 2920.3, 2349.1, 1731.9, 1649.3, 1606.7, 1408.9, 1276.7, 883.9, 764.5.

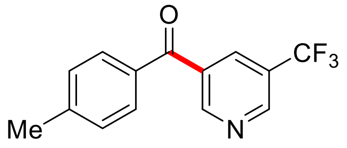

p-tolyl(5-(trifluoromethyl)pyridin-3-yl)methanone 40

The reaction was carried out according to the general procedure on 0.2 mmol scale (20 h). The crude product was purified by flash chromatography on SiO_2 (eluent: PE:EA = 100:1) to afford **40**, 37.1 mg, 70%, light yellow solid, mp = 74-76 °C, Rf = 0.4 (PE/EA = 20:1). ^{1}H NMR (400 MHz, $CDCl_3$) δ 9.14 - 8.93 (m, 2H), 8.29 - 8.22 (m, 1H), 7.65 (d, J = 8.0 Hz, 2H), 7.27 (d, J = 8.0 Hz, 2H), 2.40 (s, 3H). ^{13}C NMR (100 MHz, $CDCl_3$) δ 192.9, 153.5, 149.2 (q, J = 4.0 Hz), 144.9, 134.2 (q, J = 3.0 Hz), 133.4 (d, J = 2.0 Hz), 130.2, 129.6, 123.1 (q, J = 271.0 Hz), 21.8. ^{19}F NMR (376 MHz, $CDCl_3$) δ -62.5. HRMS (ESI) Calculated for $C_{14}H_{11}F_3NO$ $[M+H]^+$: 266.0787, found: 266.0789. IR v (neat, cm^{-1}): 3057.9, 2924.7, 1660.8, 1604.2, 1292.4, 1130.5, 1026.6, 916.2, 751.0.

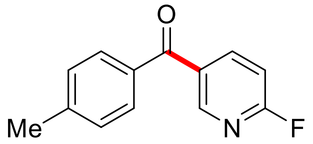

(6-fluoropyridin-3-yl)(p-tolyl)methanone 41

The reaction was carried out according to the general procedure on 0.2 mmol scale (20 h). The crude product was purified by flash chromatography on SiO_2 (eluent: PE:EA = 100:1) to afford **41**, 26.7 mg, 62%, light yellow solid, mp = 116-118 °C, Rf = 0.4 (PE/EA = 10:1). ^{1}H NMR (400 MHz, $CDCl_3$) δ 8.58 (d, J = 4.0 Hz, 1H), 8.21 - 8.15 (m, 1H), 7.63 (d, J = 8.0 Hz, 2H), 7.25 (d, J = 8.0 Hz, 2H), 7.01 - 6.96 (m, 1H), 2.38 (s, 3H). ^{13}C NMR (100 MHz, $CDCl_3$) δ 193.0, 165.1 (d, J = 244.0 Hz), 150.2 (d, J = 16.0 Hz), 144.3, 142.8 (d, J = 9.0 Hz), 134.0, 131.8 (d, J = 5.0 Hz), 130.1, 129.4, 109.7 (d, J = 37.0 Hz). ^{19}F NMR (376 MHz, Chloroform-d) δ -61.9. HRMS (ESI) Calculated for $C_{13}H_{11}FNO$

$[M+H]^+$: 216.0819, found: 216.0827. IR *v* (neat, cm^{-1}): 2922.0, 2348.9, 1646.4, 1587.3, 1480.5, 1371.7, 1253.1, 763.3.

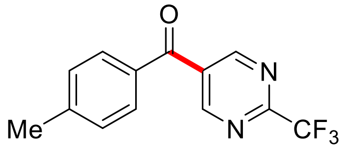

p-tolyl(2-(trifluoromethyl)pyrimidin-5-yl)methanone 42

The reaction was carried out according to the general procedure on 0.2 mmol scale (20 h). The crude product was purified by flash chromatography on SiO_2 (eluent: PE:EA = 100:1) to afford **42**, 43.1 mg, 81%, light yellow solid, mp = 77-79 °C, Rf = 0.3 (PE/EA = 20:1). ^{1}H NMR (400 MHz, Chloroform-*d*) δ 9.22 (s, 2H), 7.75 (d, *J* = 8.0 Hz, 2H), 7.38 (d, *J* = 8.0 Hz, 2H), 2.49 (s, 3H). ^{13}C NMR (100 MHz, Chloroform-*d*) δ 190.6, 158.5, 158.1 (q, *J* = 37.0 Hz), 145.8, 132.8, 130.2, 129.9, 119.3 (q, *J* = 274.0 Hz), 21.9. ^{19}F NMR (376 MHz, Chloroform-*d*) δ -70.4. HRMS (ESI) Calculated for $C_{13}H_{10}F_3N_2O$ $[M+H]^+$: 267.0740, found: 267.0741. IR *v* (neat, cm^{-1}): 3064.1, 1628.2, 1425.6, 1083.4, 715.1.

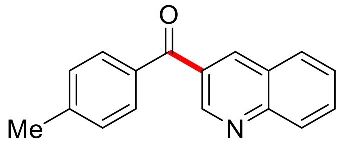

quinolin-3-yl(p-tolyl)methanone 43

The reaction was carried out according to the general procedure on 0.2 mmol scale (20 h). The crude product was purified by flash chromatography on SiO_2 (eluent: PE:EA = 100:1) to afford **43**, 25.0 mg, 50%, light yellow solid, mp = 115-117 °C, Rf = 0.3 (PE/EA = 10:1). ^{1}H NMR (400 MHz, Chloroform-*d*) δ 9.21 (d, *J* = 4.0 Hz, 1H), 8.45 (d, *J* = 4.0 Hz, 1H), 8.10 (d, *J* = 8.0 Hz, 1H), 7.82 (d, *J* = 8.0 Hz, 1H), 7.78 - 7.72 (m, 1H), 7.69 (d, *J* = 8.0 Hz, 2H), 7.57 - 7.50 (m, 1H), 7.25 (d, *J* = 8.0 Hz, 2H), 2.38 (s, 3H). ^{13}C NMR (100 MHz, Chloroform-*d*) δ 194.5, 150.4, 149.4, 144.1, 138.6, 134.4, 131.7, 130.5, 130.3, 129.5, 129.4, 129.1, 127.5, 126.7, 21.7. HRMS (ESI) Calculated for $C_{17}H_{14}NO$ $[M+H]^+$: 248.1070, found: 248.1075. IR *v* (neat, cm^{-1}): 3061.4, 2922.0, 2349.3, 1688.4, 1606.4, 1461.1, 1367.1, 1288.6, 1120.3, 747.2.

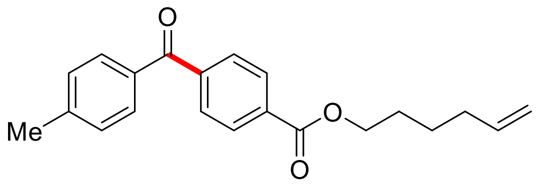

hex-5-en-1-yl 4-(4-methylbenzoyl)benzoate 44

The reaction was carried out according to the general procedure on 0.2 mmol scale (20 h). The crude product was purified by flash chromatography on SiO_2 (eluent: PE:EA = 100:1) to afford **44**, 50.9 mg, 79%, light yellow solid, mp = 124-126 °C, Rf= 0.3 (PE/EA = 10:1). ^{1}H NMR (400 MHz, $CDCl_3$) δ 8.14 (d, J = 8.0 Hz, 2H), 7.82 (d, J = 8.0 Hz, 2H), 7.72 (d, J = 8.0 Hz, 2H), 7.30 (d, J = 8.0 Hz, 2H), 5.88 - 5.78 (m, 1H), 5.07 - 4.98 (m, 2H), 4.39 - 4.35 (m, 2H), 2.45 (s, 3H), 2.17 - 2.12 (m, 2H), 1.85 - 1.78 (m, 2H), 1.61 - 1.53 (m, 2H). ^{13}C NMR (100 MHz, $CDCl_3$) δ 195.8, 165.9, 143.9, 141.7, 138.3, 134.3, 133.3, 130.4, 129.6, 129.4, 129.2, 115.0, 65.4, 33.3, 28.1, 25.3, 21.7. HRMS (ESI) Calculated for $C_{21}H_{23}O_3$ $[M+H]^+$: 323.1642, found: 323.1650. IR v (neat, cm^{-1}): 2926.9, 2349.2, 1719.7, 1659.5, 1605.9, 1404.3, 1268.7, 1103.8, 929.7, 737.3.

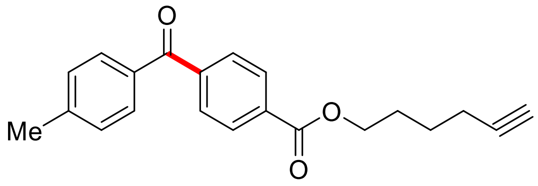

hex-5-yn-1-yl 4-(4-methylbenzoyl)benzoate 45

The reaction was carried out according to the general procedure on 0.2 mmol scale (20 h). The crude product was purified by flash chromatography on SiO_2 (eluent: PE:EA = 100:1) to afford **45**, 39.0 mg, 61%, yellow solid, mp = 110-112 °C, Rf = 0.3 (PE/EA = 20:1). ^{1}H NMR (500 MHz, $CDCl_3$) δ 8.16 (d, J = 10.0 Hz, 2H), 7.84 (d, J = 10.0 Hz, 2H), 7.74 (d, J = 10.0 Hz, 2H), 7.32 (d, J = 10.0 Hz, 2H), 4.42 (t, J = 5.0 Hz, 2H), 2.47 (s, 3H), 2.35 - 2.30 (m, 2H), 2.01 (t, J = 5.0 Hz, 1H), 1.99 - 1.93 (m, 2H), 1.77 - 1.71 (m, 2H). ^{13}C NMR (125 MHz, $CDCl_3$) δ 195.8, 165.9, 143.9, 141.7, 134.3, 133.2, 130.4, 129.7, 129.4, 129.2, 83.8, 68.9, 64.9, 27.7, 25.0, 21.7, 18.2. HRMS (ESI) Calculated for $C_{21}H_{21}O_3$ $[M+H]^+$: 321.1485, found: 321.1486. IR v (neat, cm^{-1}): 3290.9, 2954.2, 2349.2, 1720.2, 1656.3, 1604.8, 1403.9, 1311.17, 1274.4, 1102.9, 930.4, 750.8.

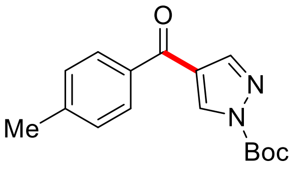

tert-butyl 4-(4-methylbenzoyl)-1H-pyrazole-1-carboxylate 46

The reaction was carried out according to the general procedure on 0.2 mmol scale (20 h). The crude product was purified by flash chromatography on SiO_2 (eluent: PE:EA = 100:1) to afford **46**, 22.0 mg, 38%, light yellow solid, mp = 119-121 °C, Rf = 0.3 (PE/EA = 10:1). ^{1}H NMR (400 MHz, Chloroform-*d*) δ 8.48 (s, 1H), 8.07 (s, 1H), 7.70 (d, J = 8.0 Hz, 2H), 7.24 (d, J = 8.0 Hz, 2H), 2.37 (s, 3H), 1.61 (s, 9H). ^{13}C NMR (100 MHz, Chloroform-*d*) δ 186.9, 146.0, 143.4, 142.8, 134.5, 133.0, 128.4, 128.1, 123.3, 85.9, 26.9, 20.6. HRMS (ESI) Calculated for $C_{16}H_{19}N_2O_3$ [M+H]$^+$: 287.1390, found: 287.1398. IR v (neat, cm^{-1}): 2981.3, 2349.4, 1753.9, 1647.8, 1552.8, 1393.1, 1256.7, 1146.5, 960.0, 840.8, 752.0.

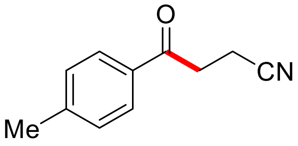

4-oxo-4-(p-tolyl)butanenitrile 47

The reaction was carried out according to the general procedure on 0.2 mmol scale (20 h). The crude product was purified by flash chromatography on SiO_2 (eluent: PE:EA = 100:1) to afford **47**, 31.8 mg, 92%, colorless oil, Rf = 0.3 (PE/EA = 5:1). ^{1}H NMR (500 MHz, Chloroform-*d*) δ 7.87 (d, J = 10.0 Hz, 2H), 7.31 (d, J = 10.0 Hz, 2H), 3.39 - 3.36 (m, 2H), 2.80 - 2.77 (m, 2H), 2.45 (s, 3H). ^{13}C NMR (125 MHz, Chloroform-*d*) δ 195.0, 144.9, 133.2, 130.2, 129.6, 129.2, 128.1, 119.4, 34.1, 21.8, 11.8. HRMS (ESI) Calculated for $C_{11}H_{12}NO$ [M+H]$^+$: 174.0913, found: 174.0912. IR v (neat, cm^{-1}): 2921.4, 2251.0, 1679.5, 1606.3, 1421.6, 1279.9, 1206.0, 1183.4, 903.0, 773.7, 722.9, 649.4, 462.9.

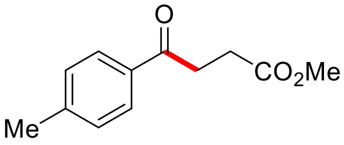

methyl 4-oxo-4-(p-tolyl)butanoate 48

The reaction was carried out according to the general procedure on 0.2 mmol scale (20 h). The crude product was purified by flash chromatography on SiO_2 (eluent: PE:EA = 100:1) to afford **48**, 28.0 mg, 68%, colorless oil, Rf = 0.3 (PE/EA = 20:1). ^{1}H NMR (400 MHz, Chloroform-*d*) δ 7.89 (d, *J* = 8.0 Hz, 2H), 7.27 - 7.25 (m, 2H), 3.71 (s, 3H), 3.30 (t, *J* = 8.0 Hz, 2H), 2.76 (t, *J* = 8.0 Hz, 2H), 2.41 (s, 3H). ^{13}C NMR (100 MHz, Chloroform-*d*) δ 197.7, 173.5, 144.1, 134.1, 129.3, 128.2, 51.8, 33.3, 28.1, 21.7. HRMS (ESI) Calculated for $C_{12}H_{15}O_3$ $[M+H]^+$: 207.1016, found: 207.1004. IR *ν* (neat, cm^{-1}): 2920.4,2349.2, 1735.3, 1681.4, 1607.0, 1436.8, 1221.1, 1121.4, 810.4, 732.7, 554.9.

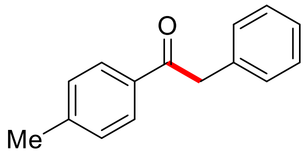

2-phenyl-1-(p-tolyl)ethan-1-one 49

In the nitrogen-filled glove box, an oven-dried 8 mL screw-cap vial was successively added stirring bar, $NiBr_2$•dme (3.1 mg, 5.0 mol%), 4,4’-di-tertbutyl-2,2’-bipyridine (5.3 mg, 10.0 mol%) and DMF (2.0 mL), and then it was sealed and stirred until the resulting mixture became homogenous solution (about 20 min). Subsequently, the nickel-catalyst stock solution was syringed into an oven-dried 10 mL Schlenk tube which equipped a magnetic stir bar as well as photocatalyst $Ir[dF(CF_3)ppy]_2(dtbbpy)PF_6$ (2.2 mg, 1 mol%), aromatic carboxylic acid (27.2 mg, 0.2 mmol), (chloromethyl)benzene (50.8 mg, 0.4 mmol), Ph_3P (78.6 mg, 0.3 mmol), Et_3N (20.2 mg, 0.2 mmol). The Schlenk tube was evacuated and backfilled with argon three times. The Schlenk tube was then sealed and was placed at a distance (app. 5 cm) from 2 x 45 W blue LEDs, and the mixture was stirred for 20 h at room temperature (air-condition was used to keep the temperature is 25 °C or so). After completion, the reaction mixture was removed from the light, diluted with water and EtOAc, and then aqueous layer was extracted with three portions of EtOAc. The combined organic layers were washed with brine, dried over anhydrous Na_2SO_4, filtered, and concentrated. The crude product was purified by flash chromatography on SiO_2 (eluent: PE:EA = 100:1) to afford **49**, 19.0 mg, 46%, light yellow solid, Rf = 0.6 (PE/EA = 20:1). ^{1}H NMR (400 MHz, Chloroform-*d*) δ 7.91 (d, *J* = 8.0 Hz, 2H), 7.44 - 7.12 (m, 7H), 4.26 (s, 2H), 2.40 (s, 3H). ^{13}C NMR (100 MHz,

Chloroform-*d*) δ 197.3, 144.0, 134.8, 134.2, 129.4, 129.3, 128.8, 128.6, 126.8, 45.4, 21.7. These data are in agreement with those reported previously in the literature.[6]

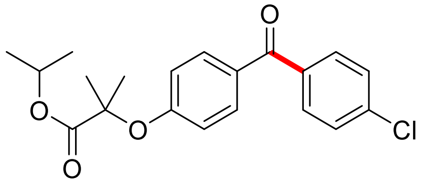

isopropyl 2-(4-(4-chlorobenzoyl)phenoxy)-2-methylpropanoate 50

The reaction was carried out according to the general procedure on 0.2 mmol scale (20 h). The crude product was purified by flash chromatography on SiO_2 (eluent: PE:EA = 100:1) to afford **50**, 46.8 mg, 65%, light yellow solid, Rf = 0.3 (PE/EA = 10:1). ^{1}H NMR (400 MHz, Chloroform-*d*) δ 7.69 - 7.59 (m, 4H), 7.37 (d, *J* = 8.0 Hz, 2H), 6.79 (d, *J* = 8.0 Hz, 2H), 5.05 - 4.98 (m, 1H), 1.59 (s, 6H), 1.14 (s, 3H), 1.12 (s, 3H). ^{13}C NMR (100 MHz, Chloroform-*d*) δ 193.2, 172.1, 158.7, 137.3, 135.4, 130.9, 130.1, 129.2, 127.5, 116.3, 78.4, 68.3, 24.4, 20.5. These data are in agreement with those reported previously in the literature.[7]

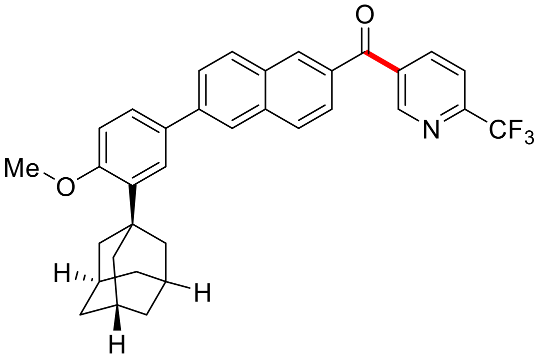

(6-(3-((1r,3R,5S)-adamantan-1-yl)-4-methoxyphenyl)naphthalen-2-yl)(6 (trifluoromethyl)pyridin-3-yl)methanone 51

The reaction was carried out according to the general procedure on 0.2 mmol scale (36 h). The crude product was purified by flash chromatography on SiO_2 (eluent: PE:EA = 100:1) to afford **51**, 43.3 mg, 40%, yellow solid, mp = 225-227 °C, Rf = 0.3 (PE/EA = 20:1). ^{1}H NMR (500 MHz, Chloroform-*d*) δ 9.18 (s, 1H), 8.37 (d, *J* = 9.7 Hz, 1H), 8.28 (s, 1H), 8.09 (s, 1H), 8.05 (d, *J* = 8.6 Hz, 1H), 8.02 - 7.99 (m, 2H), 7.92 (d, *J* = 8.0 Hz, 1H), 7.88 (d, *J* = 8.5 Hz, 1H), 7.65 (s, 1H), 7.60 - 7.58 (m, 1H), 7.04 (d, *J* = 8.5 Hz, 1H), 3.94 (s, 3H), 2.22 (s, 6H), 2.14 (s, 3H), 1.83 (s, 6H). ^{13}C NMR (100 MHz,

Chloroform-*d*) δ 193.3, 159.2, 150.7, 150.3 (q, *J* = 35.0 Hz), 142.4, 139.1, 138.7, 136.3, 136.2, 132.8, 132.5, 132.2, 130.9, 130.0, 129.1, 127.1, 126.0, 125.8, 125.4, 124.8, 123.0 (q, *J* = 273.0 Hz), 120.4 (q, *J* = 2.0 Hz), 112.2, 55.2, 40.6, 37.2, 37.1, 29.7, 29.3, 29.1. ^{19}F NMR (376 MHz, Chloroform-*d*) δ -68.1. HRMS (ESI) Calculated for $C_{34}H_{31}F_3NO_2$ $[M+H]^+$: 542.2301, found: 542.2307. IR *v* (neat, cm^{-1}): 2908.6, 2253.7, 1660.4, 1472.2, 1264.5, 1072.9, 905.2, 725.8, 646.8.

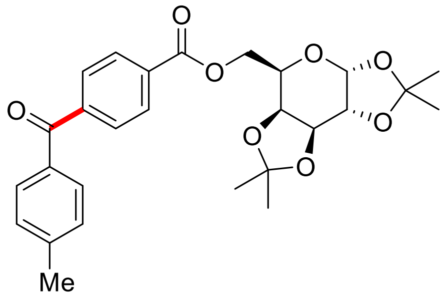

1-(4-(4-methylbenzoyl)phenyl)-2-((3aR,5R,5aS,8aS,8bR)-2,2,7,7-tetramethyltetrahydro-5H-bis([1,3]dioxolo)[4,5-b:4',5'-d]pyran-5-yl)ethan-1-one 52

The reaction was carried out according to the general procedure on 0.2 mmol scale (36 h). The crude product was purified by flash chromatography on SiO_2 (eluent: PE:EA = 50:1) to afford **52**, 39.2 mg, 42%, colorless oil, Rf = 0.3 (PE/EA = 5:1). ^{1}H NMR (400 MHz, Chloroform-*d*) δ 8.08 (d, *J* = 8.0 Hz, 2H), 7.73 (d, *J* = 8.0 Hz, 2H), 7.64 (d, *J* = 8.0 Hz, 2H), 7.22 (d, *J* = 8.0 Hz, 2H), 5.52 - 4.47 (m, 1H), 4.44 - 4.36 (m, 1H), 4.31 - 4.24 (m, 2H), 4.15 - 4.12 (m, 1H), 2.38 (s, 3H), 1.45 (s, 3H), 1.41 (s, 3H), 1.29 (s, 3H), 1.27 (s, 3H). ^{13}C NMR (100 MHz, Chloroform-*d*) δ 195.8, 165.7, 143.9, 141.8, 134.3, 132.9, 130.4, 129.6, 129.6, 129.2, 109.8, 108.8, 96.3, 71.1, 70.8, 70.5, 66.1, 64.3, 26.0, 26.0, 25.0, 24.5, 21.7. HRMS (ESI) Calculated for $C_{27}H_{31}O_8$ $[M+H]^+$: 483.2013, found: 483.2016. IR *v* (neat, cm^{-1}): 2987.7, 2922.9, 1723.4, 1660.2, 1606.1, 1405.1, 1271.7, 1211.7, 1102.8, 1069.6, 1006.4, 960.2, 765.2.

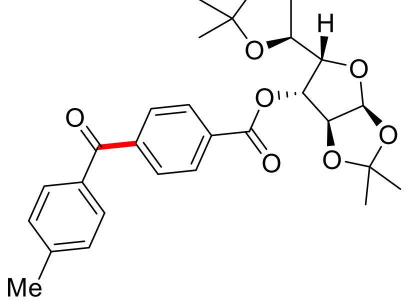

(3aS,5S,6R,6aS)-5-((S)-2,2-dimethyl-1,3-dioxolan-4-yl)-2,2-dimethyltetrahydrofuro[2,3-d][1,3]dioxol-6-yl 4-(4-methylbenzoyl)benzoate 53

The reaction was carried out according to the general procedure on 0.2 mmol scale (36 h). The crude product was purified by flash chromatography on SiO_2 (eluent: PE:EA = 100:1) to afford **53**. 61.2 mg, 64 %, colorless oil, Rf = 0.4 (PE/EA = 5:1). ^{1}H NMR (400 MHz, Chloroform-*d*) δ 8.05 (d, *J* = 8.0 Hz, 2H), 7.75 (d, *J* = 8.0 Hz, 2H), 7.64 (d, *J* = 8.0 Hz, 2H), 7.23 (d, *J* = 8.0 Hz, 2H), 5.90 (d, *J* = 4.0 Hz, 1H), 5.46 (d, *J* = 4.0 Hz, 1H), 4.59 (d, *J* = 4.0 Hz, 1H), 4.31 - 4.24 (m, 2H), 4.08 - 4.00 (m, 2H), 2.38 (s, 3H), 1.49 (s, 3H), 1.35 (s, 3H), 1.26 (s, 3H), 1.21 (s, 3H). ^{13}C NMR (100 MHz, Chloroform-*d*) δ 195.5, 164.5, 144.1, 142.2, 134.1, 132.3, 130.3, 129.7, 129.6, 129.2, 112.4, 109.5, 105.1, 83.4, 80.0, 72.6, 67.4, 26.9, 26.7, 26.2, 25.2, 21.7. HRMS (ESI) Calculated for $C_{27}H_{31}O_8$ $[M+H]^+$: 483.2013, found: 483.2019. IR *v* (neat, cm^{-1}): 2928.4, 1718.9, 1659.4, 1312.3, 737.1.

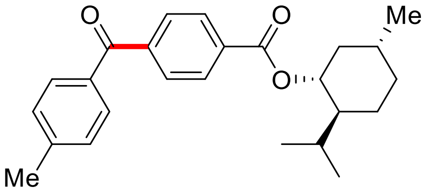

(1R,2S,5R)-2-isopropyl-5-methylcyclohexyl 4-(4-methylbenzoyl)benzoate 54

The reaction was carried out according to the general procedure on 0.2 mmol scale (36 h). The crude product was purified by flash chromatography on SiO_2 (eluent: PE:EA = 100:1) to afford **54**, 42.3 mg, 56%, colorless oil, Rf = 0.3 (PE/EA = 5:1). ^{1}H NMR (400 MHz, Chloroform-*d*) δ 8.06 (d, *J* = 8.0 Hz, 2H), 7.74 (d, *J* = 8.0 Hz, 2H), 7.65 (d, *J* = 8.0 Hz, 2H), 7.22 (d, *J* = 8.0 Hz, 2H), 4.94 - 4.86 (m, 1H), 2.37 (s, 3H), 2.10 - 2.03 (m, 1H), 1.93 - 1.85 (m, 1H), 1.71 - 1.63 (m, 2H), 1.57 - 1.47 (m, 3H), 1.10 - 1.00 (m, 2H), 0.89 - 0.84 (m, 7H), 0.74 (d, *J* = 8.0 Hz, 3H). ^{13}C NMR (100 MHz, Chloroform-*d*) δ 195.8, 165.4, 143.9, 141.6, 134.3, 133.7, 130.4, 129.6, 129.4, 129.1, 75.4, 47.3, 40.9, 34.3, 31.5, 26.6, 23.6, 22.1, 21.7, 20.8, 16.5. HRMS (ESI) Calculated for $C_{25}H_{31}O_3$ $[M+H]^+$: 379.2268, found: 379.2272. IR *v* (neat, cm^{-1}): 2955.9, 2869.4, 1715.4, 1661.1, 1455.5, 1272.0, 1104.1, 914.7, 784.1.

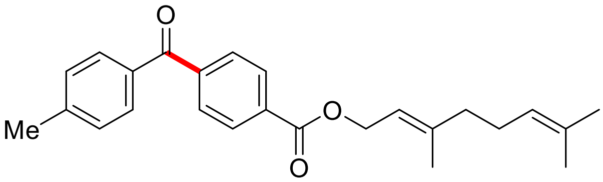

(E)-3,7-dimethylocta-2,6-dien-1-yl 4-(4-methylbenzoyl)benzoate 55

The reaction was carried out according to the general procedure on 0.2 mmol scale (36 h). The crude product was purified by flash chromatography on SiO_2 (eluent: PE:EA = 100:1) to afford **55**, 55.6 mg, 74%, colorless oil, Rf = 0.4 (PE/EA = 20:1). ^{1}H NMR (400 MHz, Chloroform-*d*) δ 8.07 (d, *J* = 8.0 Hz, 2H), 7.73 (d, *J* = 8.0 Hz, 2H), 7.64 (d, *J* = 8.0 Hz, 2H), 7.22 (d, *J* = 8.0 Hz, 2H), 5.46 - 5.38 (m, 1H), 5.06 - 4.98 (m, 1H), 4.81 (d, *J* = 8.0 Hz, 2H), 2.37 (s, 3H), 2.08 - 1.98 (m, 4H), 1.71 (s, 3H), 1.61 (s, 3H), 1.54 (s, 3H). ^{13}C NMR (100 MHz, Chloroform-*d*) δ 195.8, 165.9, 143.9, 142.8, 141.6, 134.3, 133.4, 131.9, 130.3, 129.6, 129.5, 129.1, 123.7, 118.1, 62.3, 39.6, 26.3, 25.7, 21.7, 17.7, 16.6. HRMS (ESI) Calculated for $C_{25}H_{29}O_3$ $[M+H]^+$: 377.2111, found: 377.2112. IR *ν* (neat, cm^{-1}): 2923.1, 2852.8, 1720.0, 1659.9, 1606.1, 1404.9, 1272.3, 1101.5, 930.2, 764.0.

Synthesis of starting materials

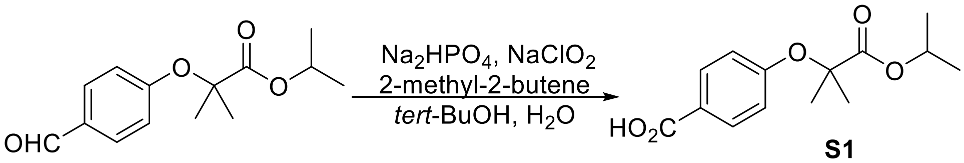

S1

Following the general procedure of reported literature,[7] to a solution of isopropyl 2-(4-formylphenoxy)-2-methylpropanoate[6] (0.89 g, 3.6 mmol), NaH_2PO_4 (432 mg, 3.6 mmol), 2-methyl-2-butene (1.2 g, 15.8 mmol) in *tert*-BuOH (20 mL) and water (4 mL) was added $NaClO_2$ (3.4 g, 12.2 mmol) and the mixture was stirred for 50 min at room temperature. The reaction mixture was adjusted to pH of 4 by addition of 1 M HCl. The aqueous layer was extracted with CH_2Cl_2. The organic layers were combined, washed with brine, dried over anhydrous Na_2SO_4. Purification by flash chromatography (PE/EA = 20:1/5:1), afforded the corresponding product **S1**.

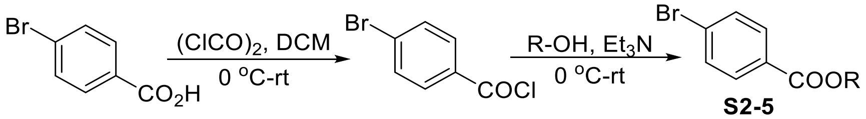

S2-5

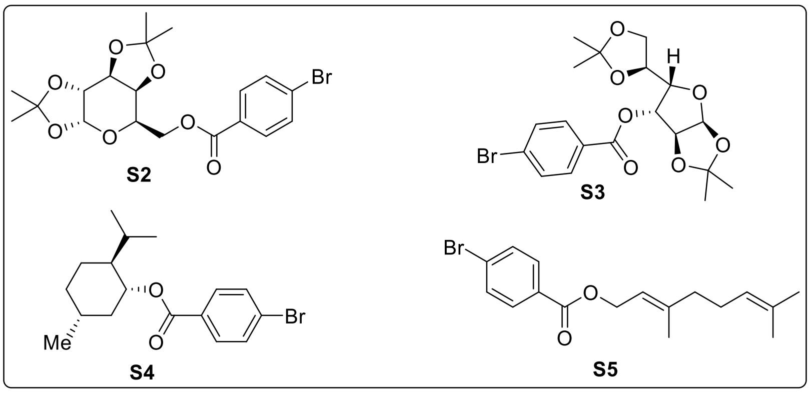

Prepared according to general procedure:[8] to a stirred solution of bromobenzoic acid (10 mmol, 1.0 eq) in DCM (30 mL) and DMF (0.15 mL) under argon was added oxalyl chloride (13.0 mmol, 1.3 eq) by syringe. The reaction mixture was stirred for 1 h at 0 ºC and then warmed up to room temperature. After 3 h, a clear light-yellow solution was obtained and all of the volatiles were removed under vacuum. The residue was then dissolved in DCM (10 mL) and added to a mixture of an alcohol (7.2 mmol, 1.0 eq), DMAP (0.72 mmol, 0.1 eq) and Et_3N (15 mmol, 2.0 eq) in DCM (10 mL). The reaction was allowed to stirred overnight at room temperature. Then, the mixture was quenched upon addition of NH_4Cl (aq. 10%), and extracted with DCM (3x). The organic phase was washed with brine, concentrated and purified by silica gel flash chromatography to give the corresponding substituted aryl bromides **S2-5**.

Preliminary investigation of other carboxylic acids

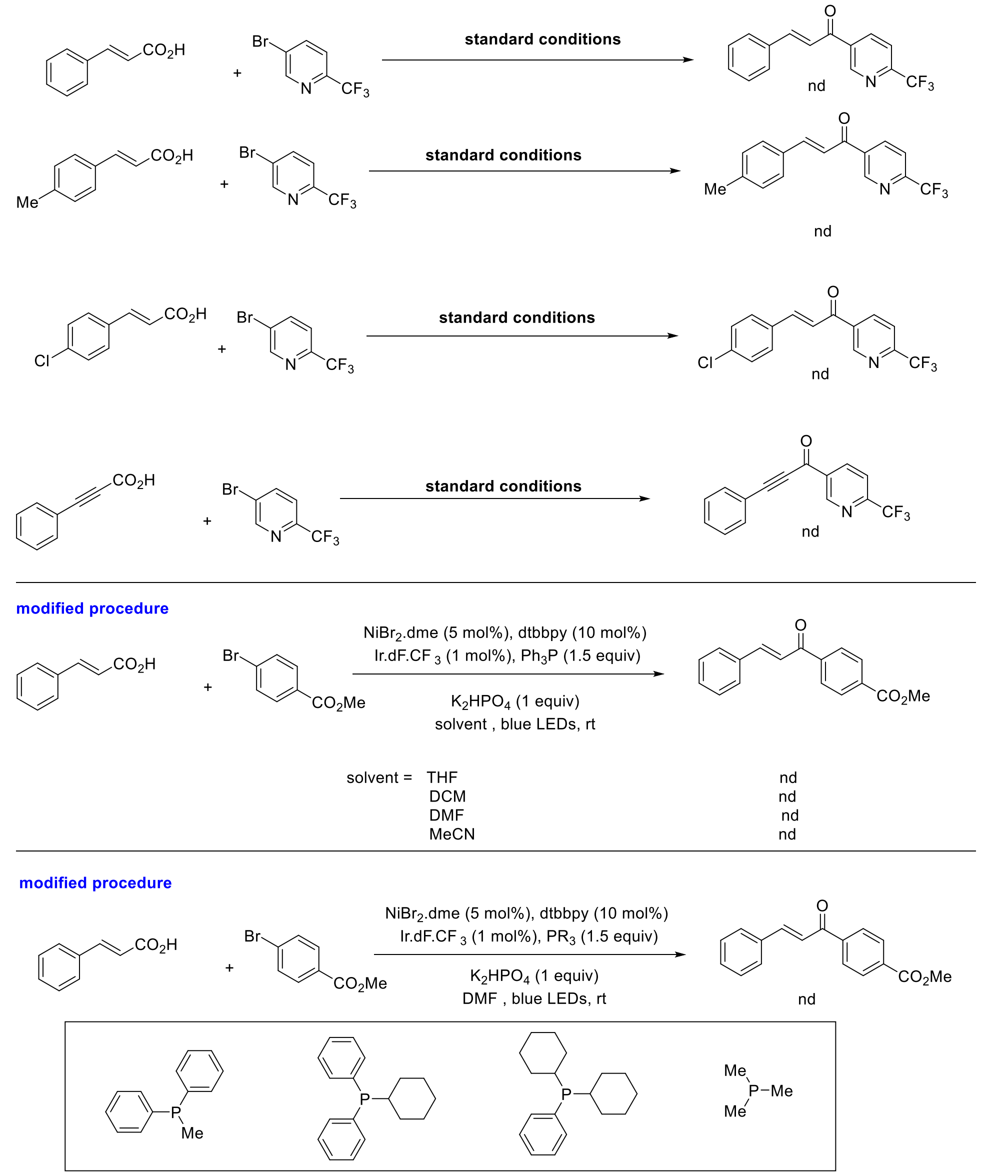

Supplementary figure 12. Preliminary investigation of other carboxylic acids

Copies of ^{1}H NMR, ^{13}C NMR, ^{19}F NMR spectra

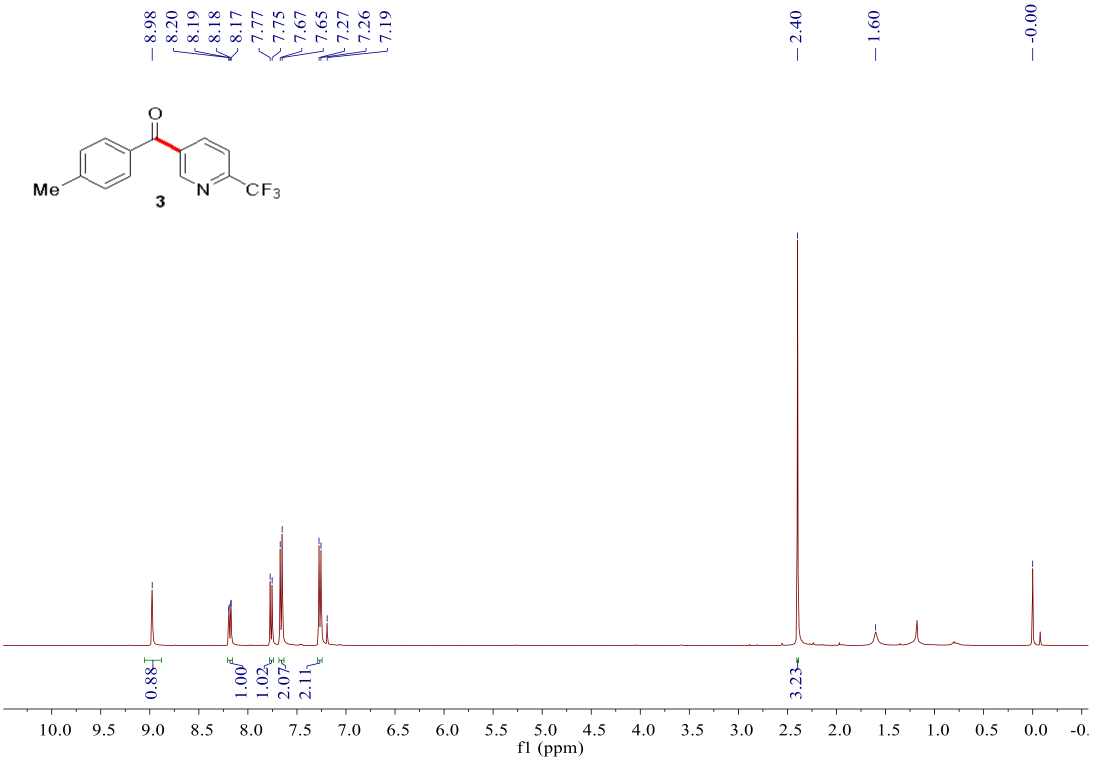

Supplementary Figure 13. ^{1}H NMR spectra for compound **3**

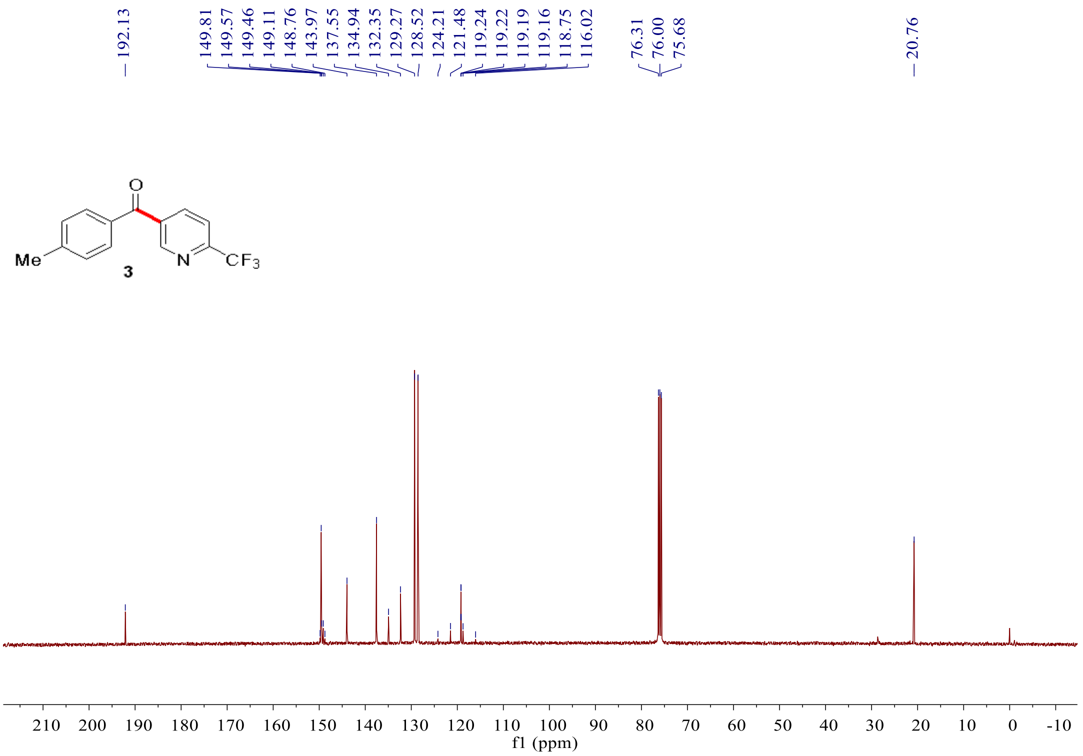

Supplementary Figure 14. ^{13}C NMR spectra for compound **3**

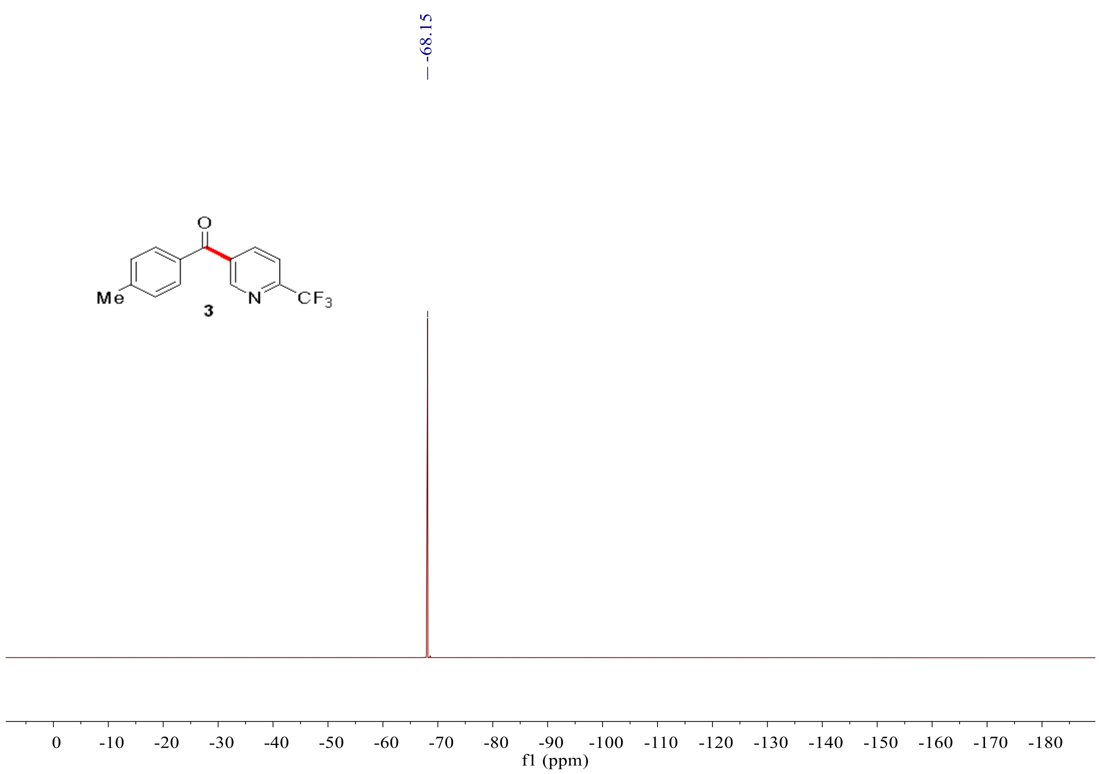

Supplementary Figure 15. ^{19}F NMR spectra for compound **3**

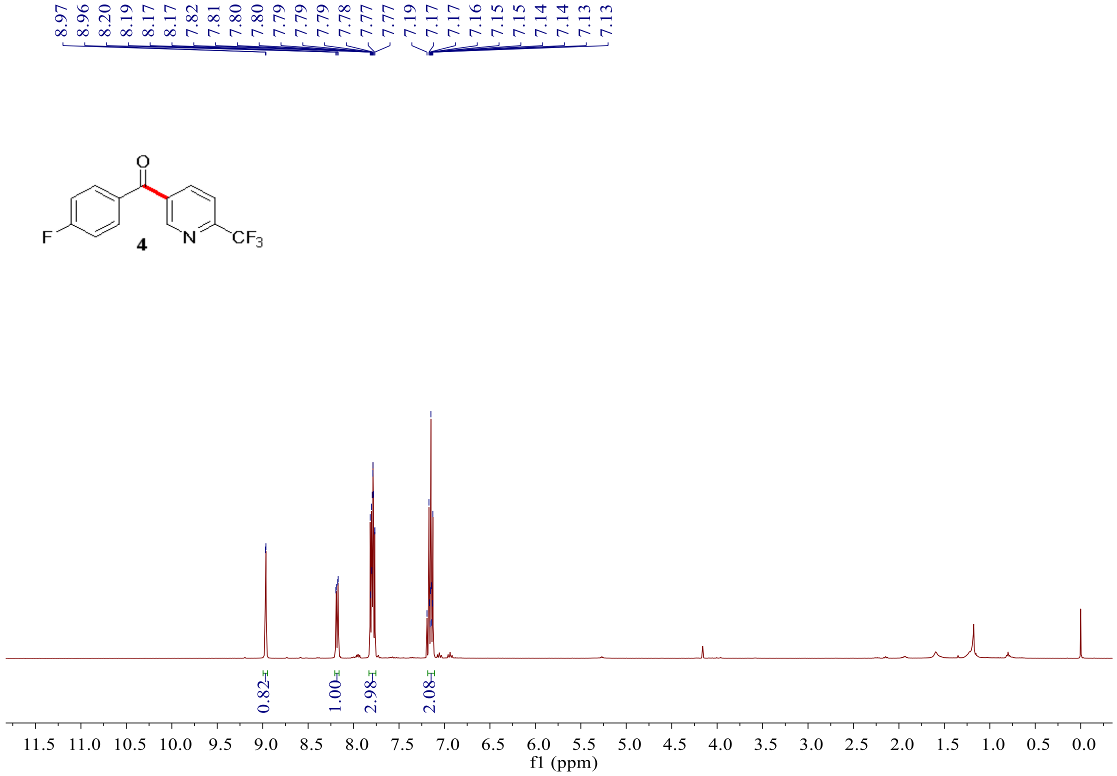

Supplementary Figure 16. ^{1}H NMR spectra for compound **4**

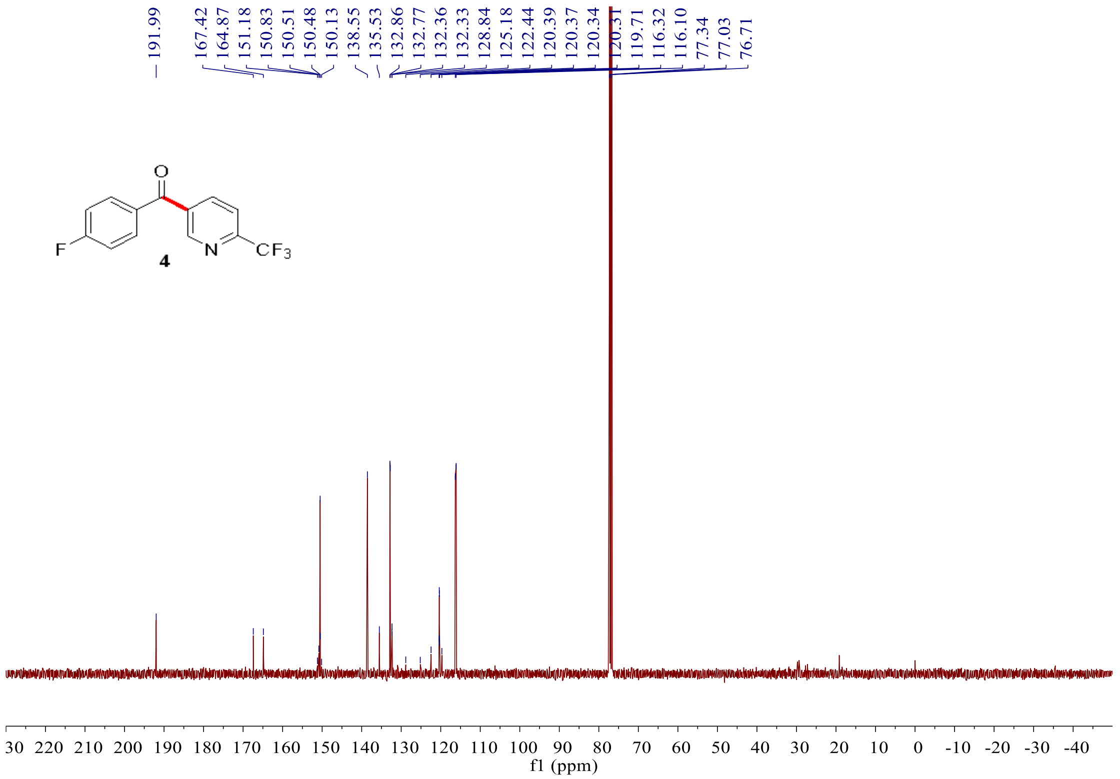

Supplementary Figure 17. ^{13}C NMR spectra for compound **4**

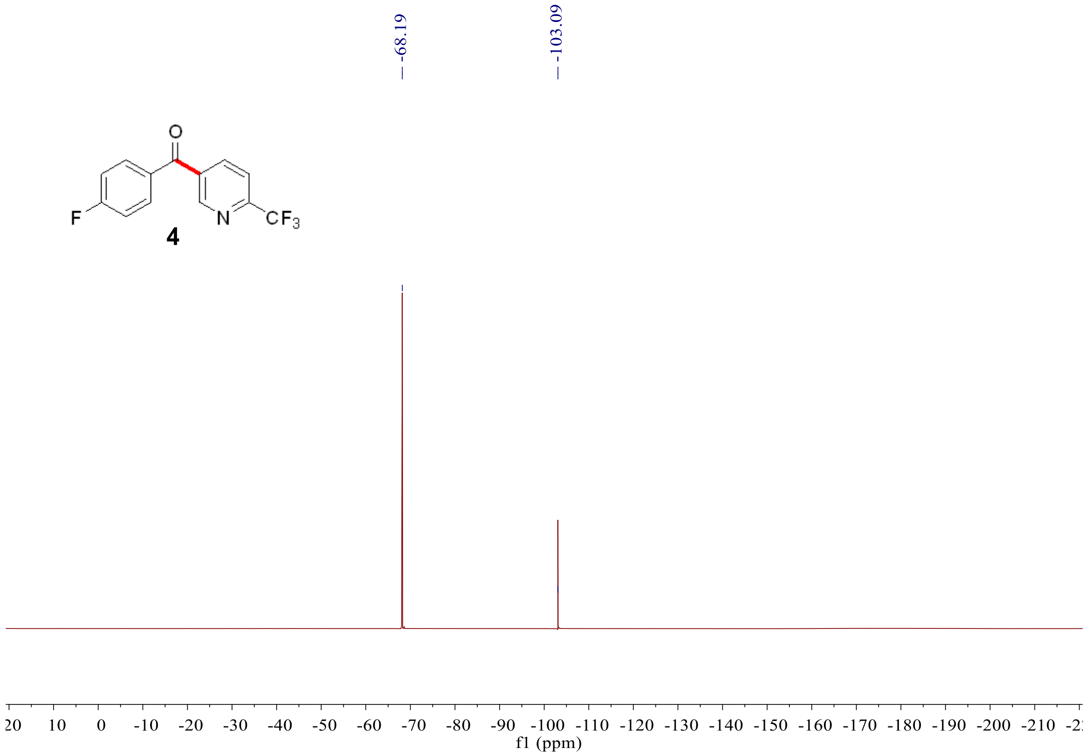

Supplementary Figure 18. ^{19}F NMR spectra for compound **4**

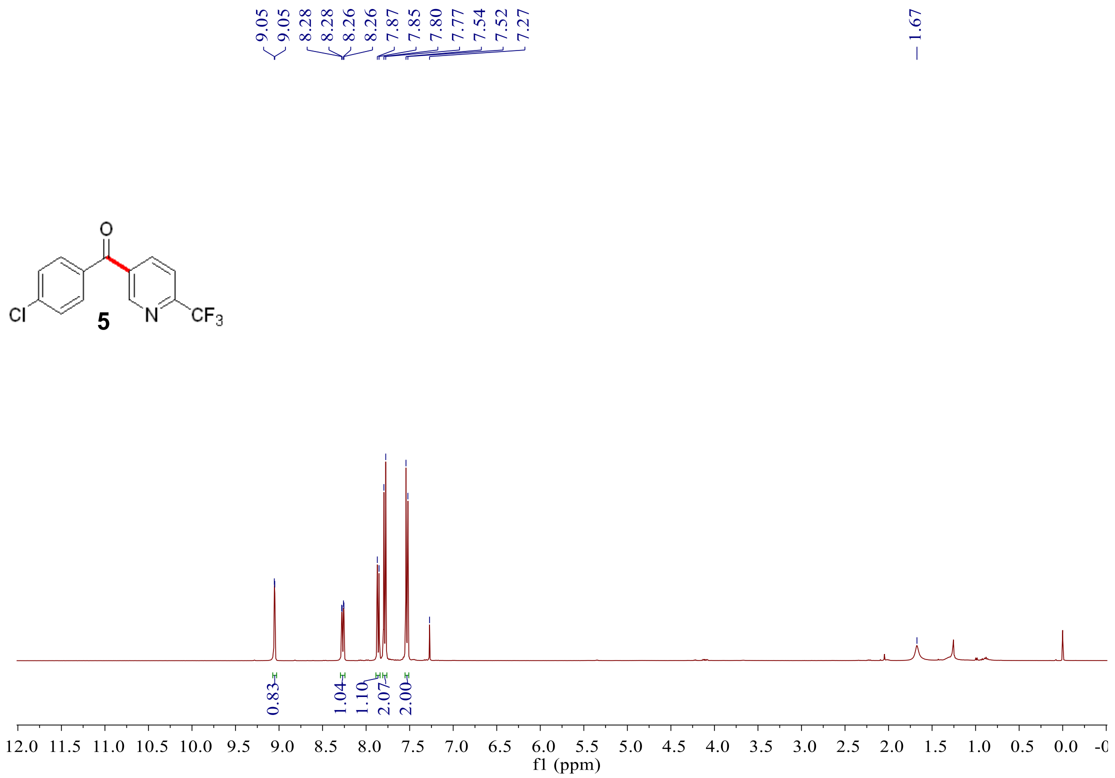

Supplementary Figure 19. ^{1}H NMR spectra for compound **5**

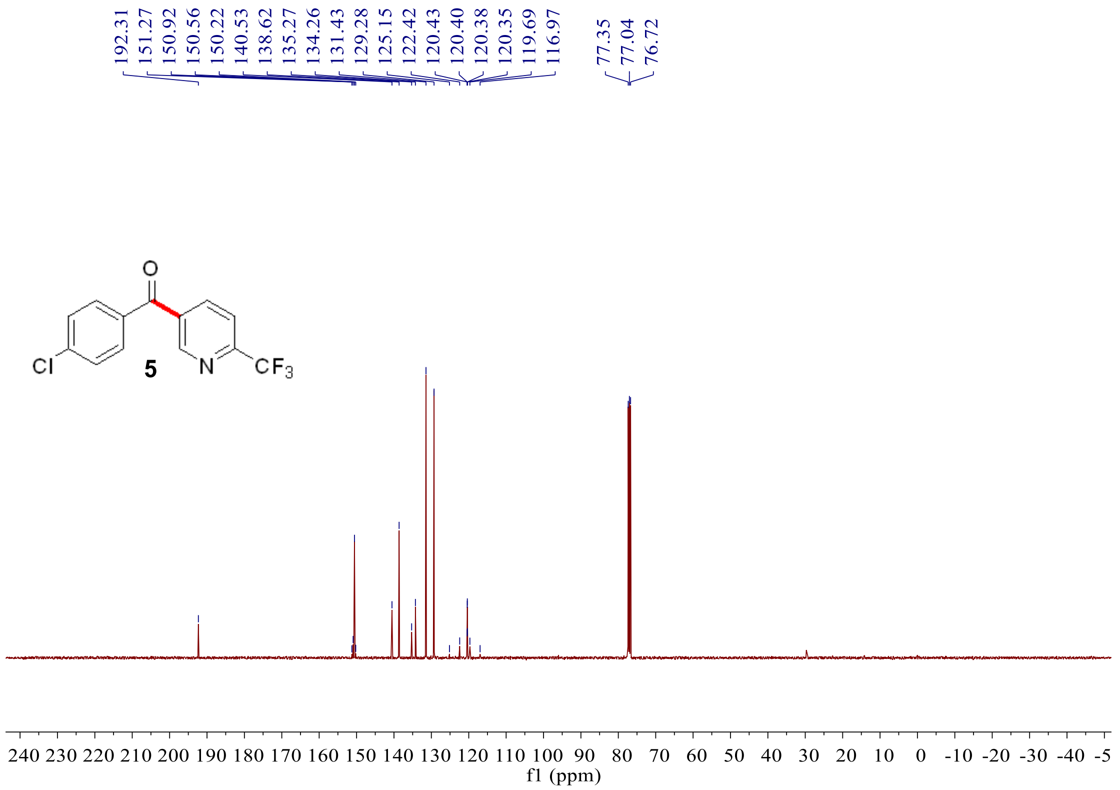

Supplementary Figure 20. ^{13}C NMR spectra for compound **5**

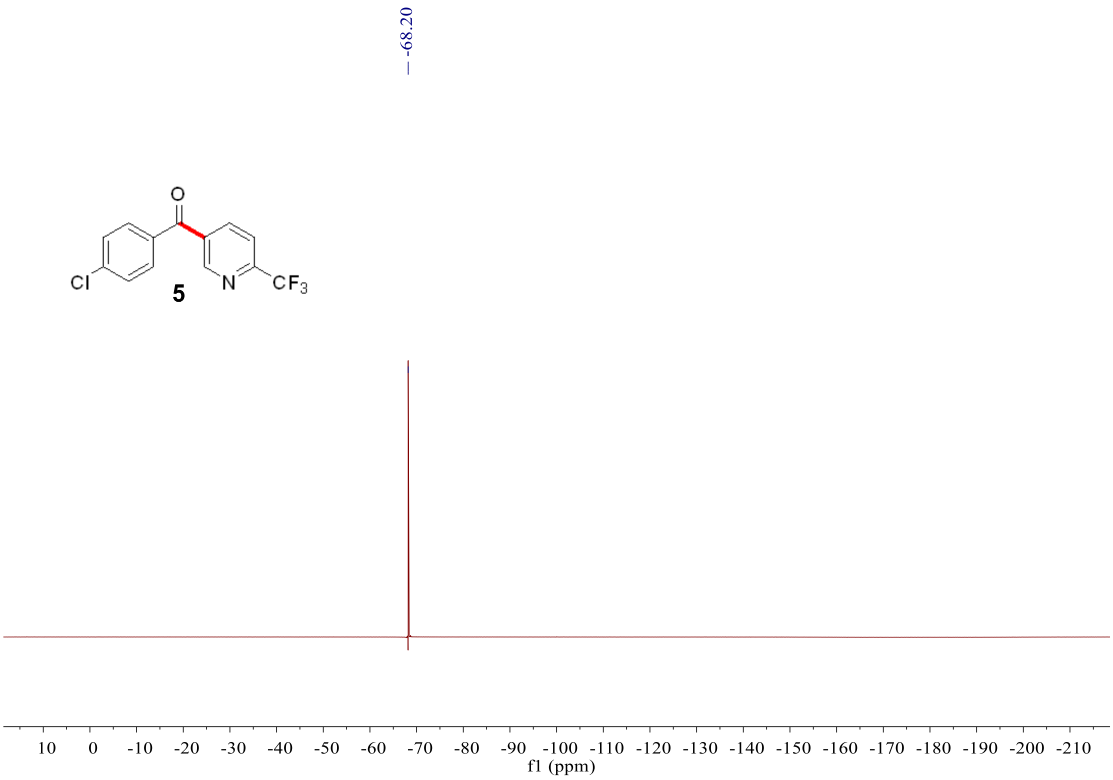

Supplementary Figure 21. ^{19}F NMR spectra for compound **5**

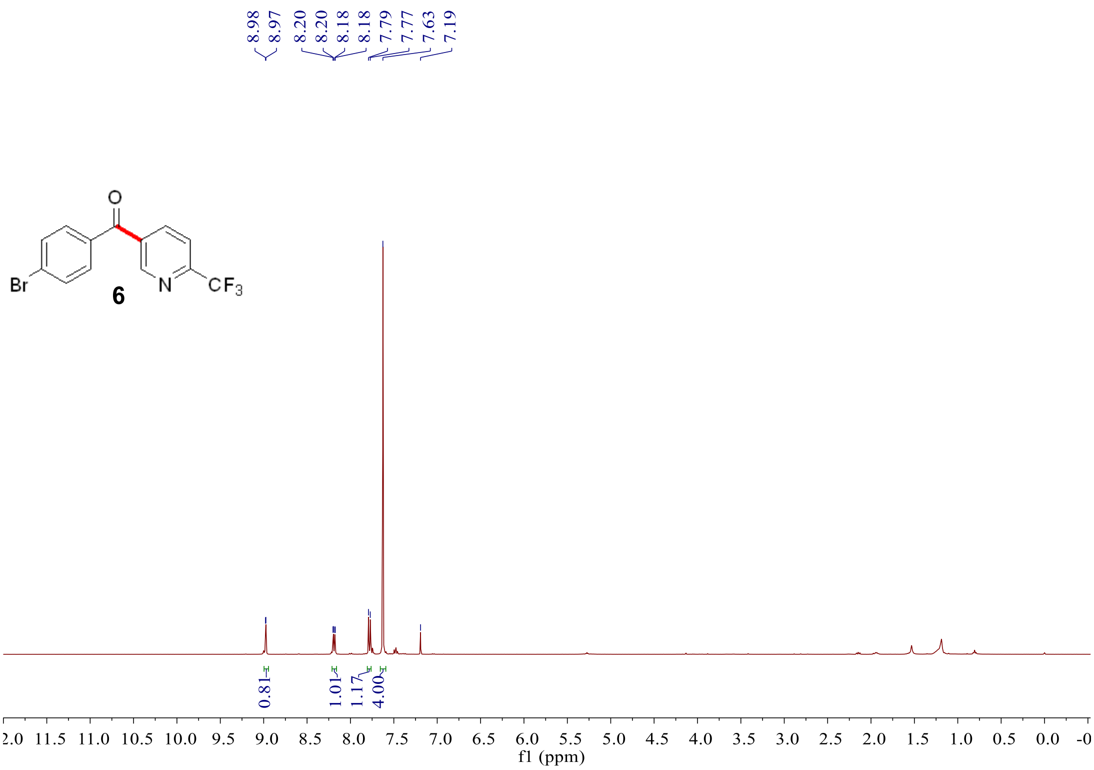

Supplementary Figure 22. ^{1}H NMR spectra for compound **6**

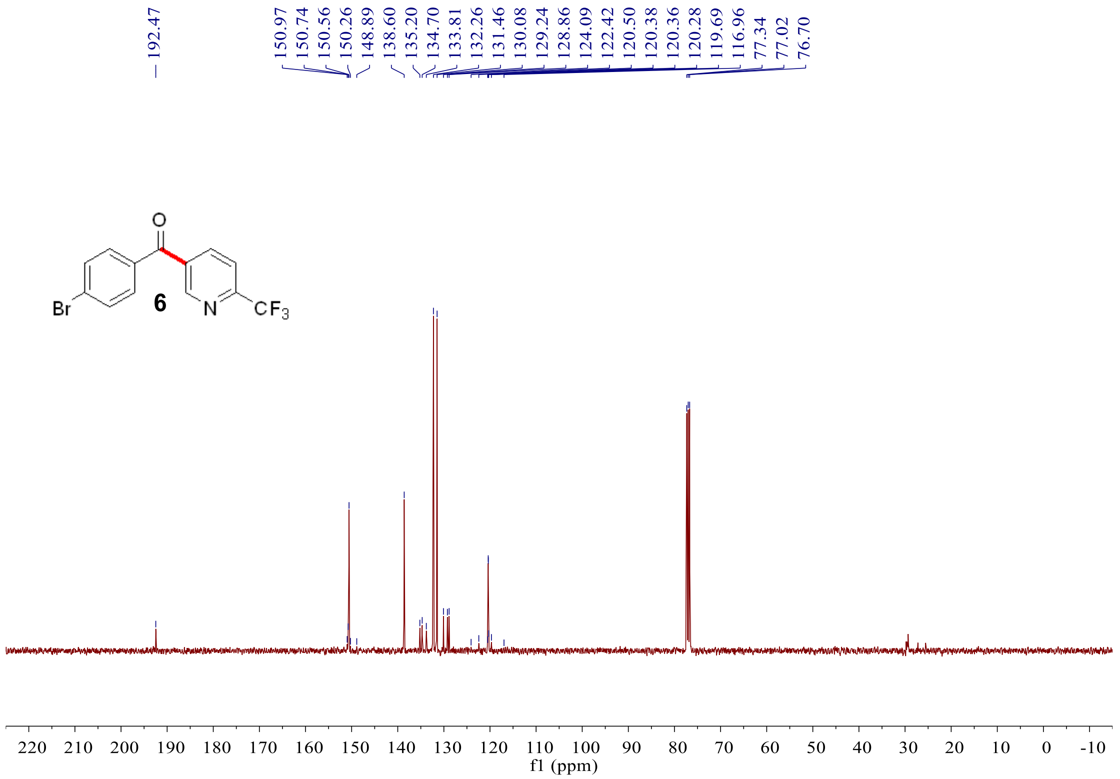

Supplementary Figure 23. ^{13}C NMR spectra for compound **6**

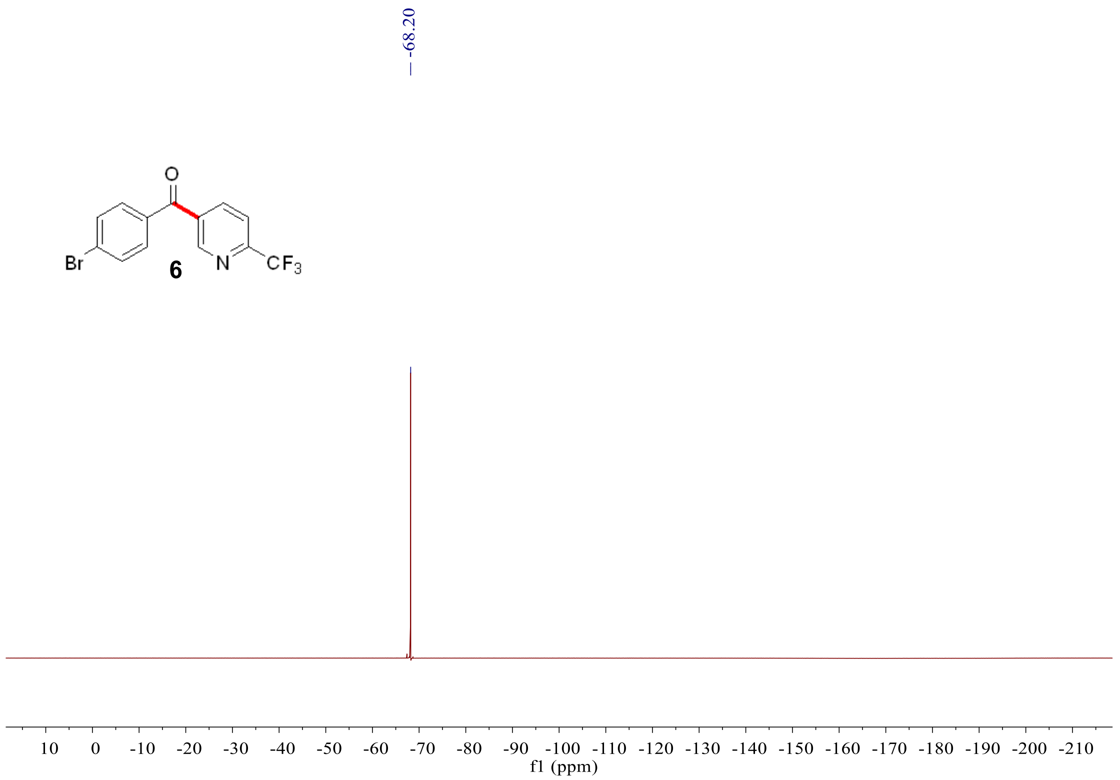

Supplementary Figure 24. ^{19}F NMR spectra for compound **6**

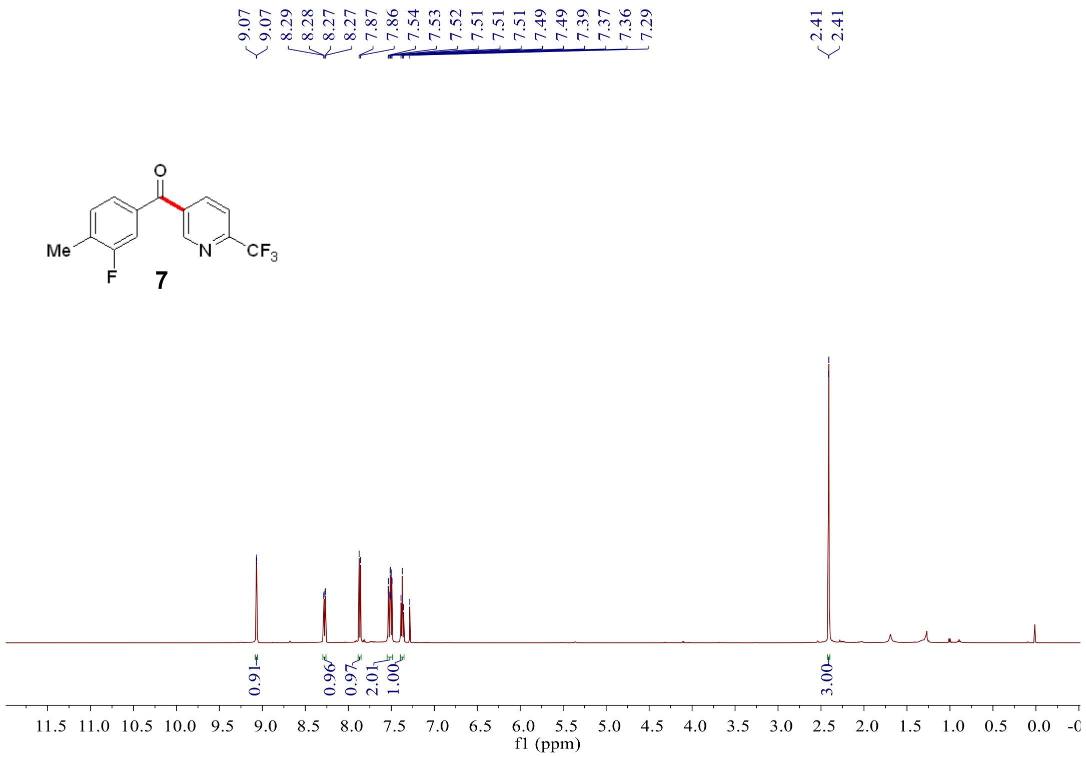

Supplementary Figure 25. ^{1}H NMR spectra for compound **7**

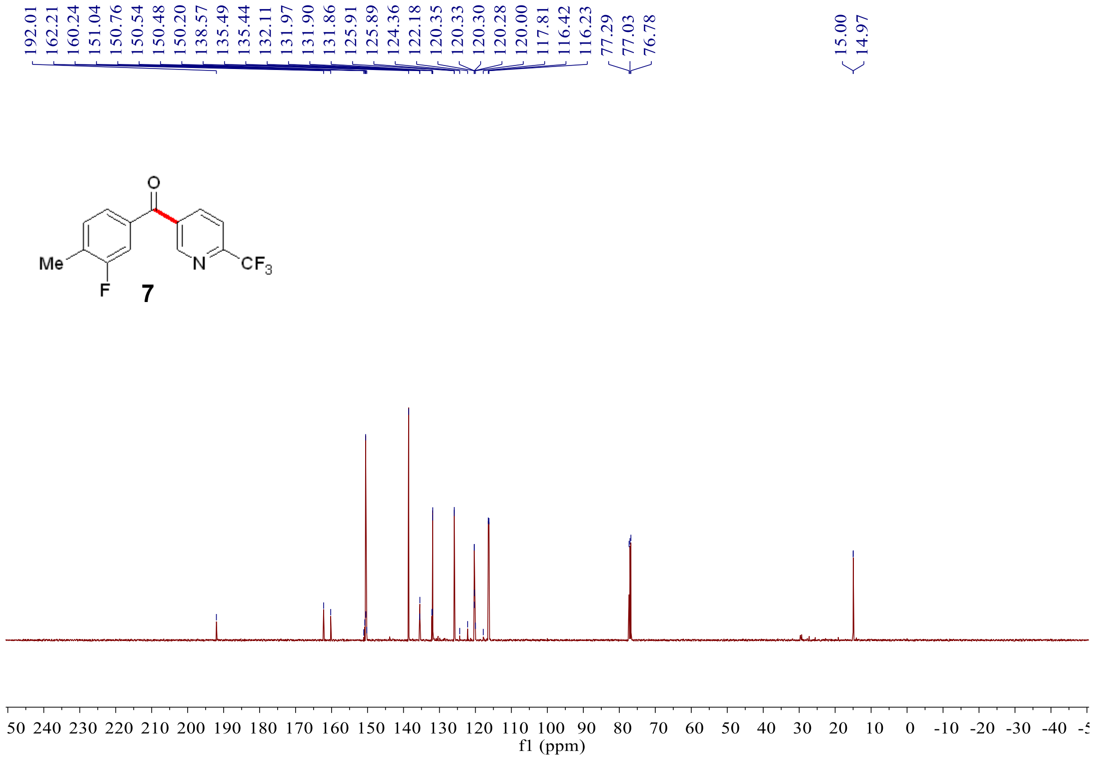

Supplementary Figure 26. ^{13}C NMR spectra for compound **7**

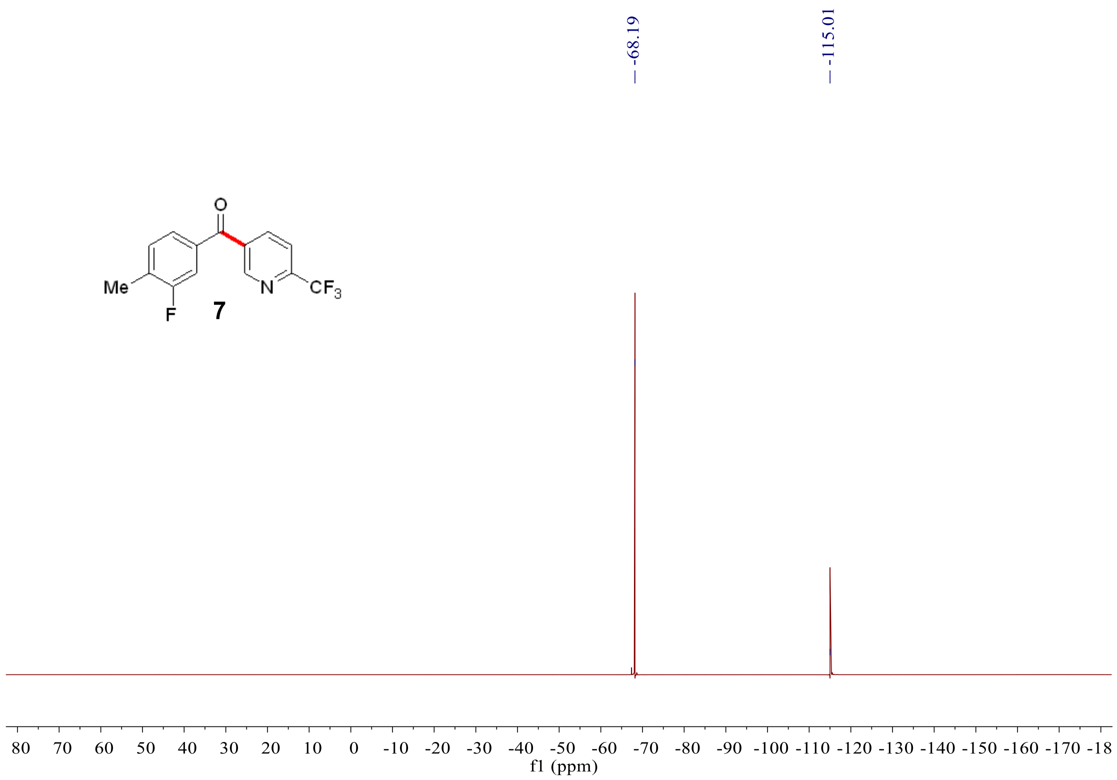

Supplementary Figure 27. ^{19}F NMR spectra for compound **7**

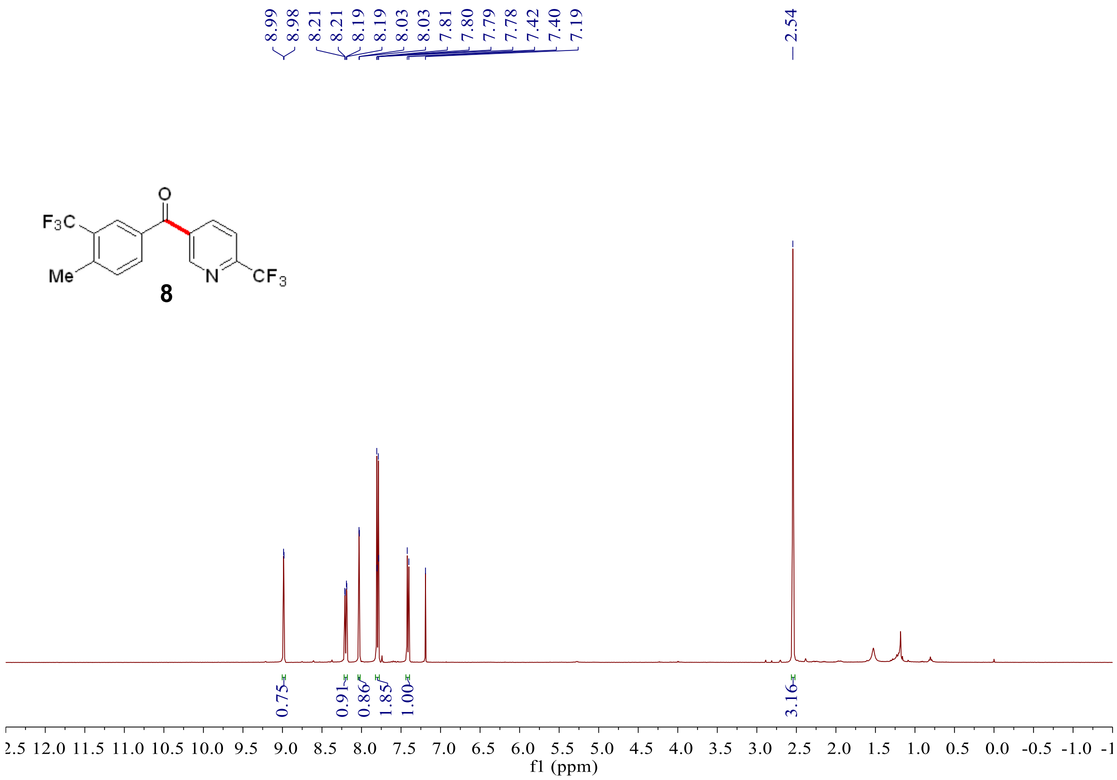

Supplementary Figure 28. ^{1}H NMR spectra for compound **8**

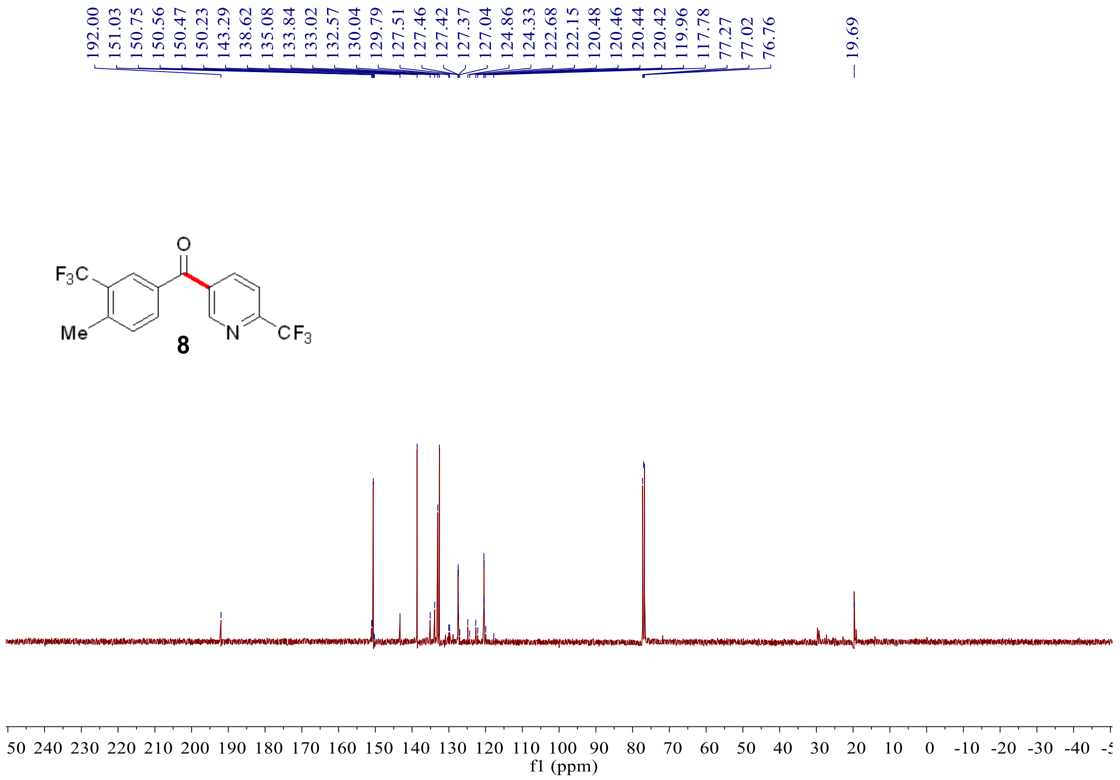

Supplementary Figure 29. ^{13}C NMR spectra for compound **8**

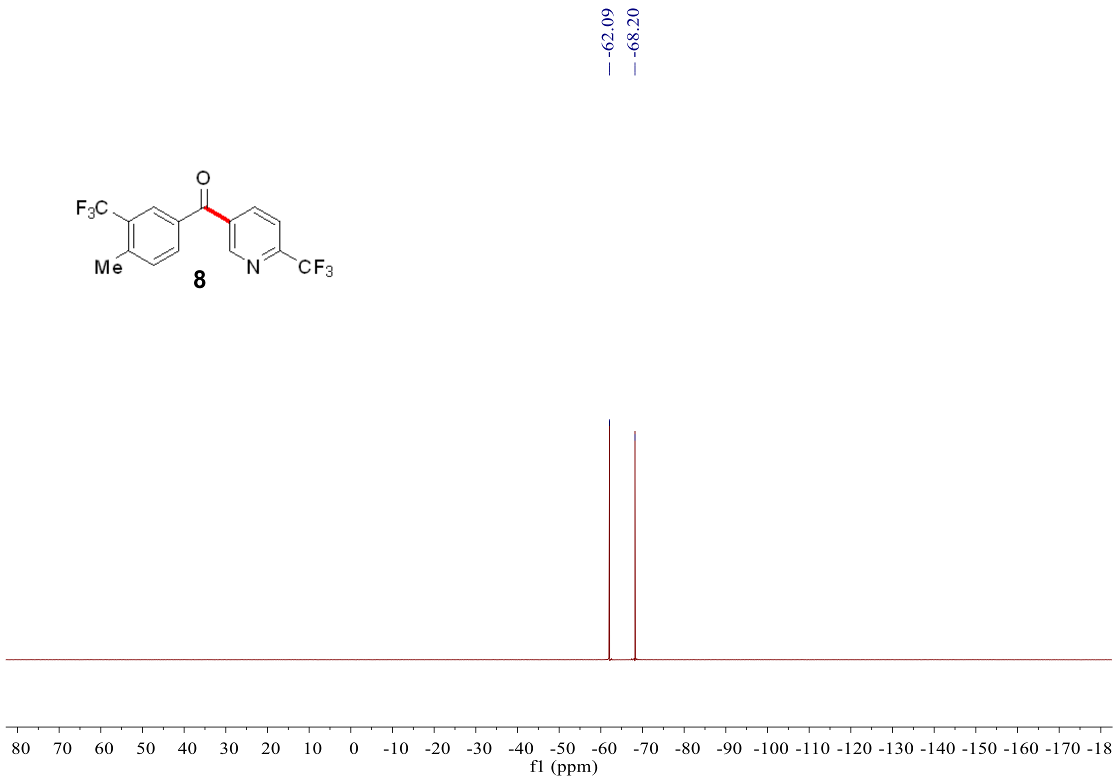

Supplementary Figure 30. ^{19}F NMR spectra for compound **8**

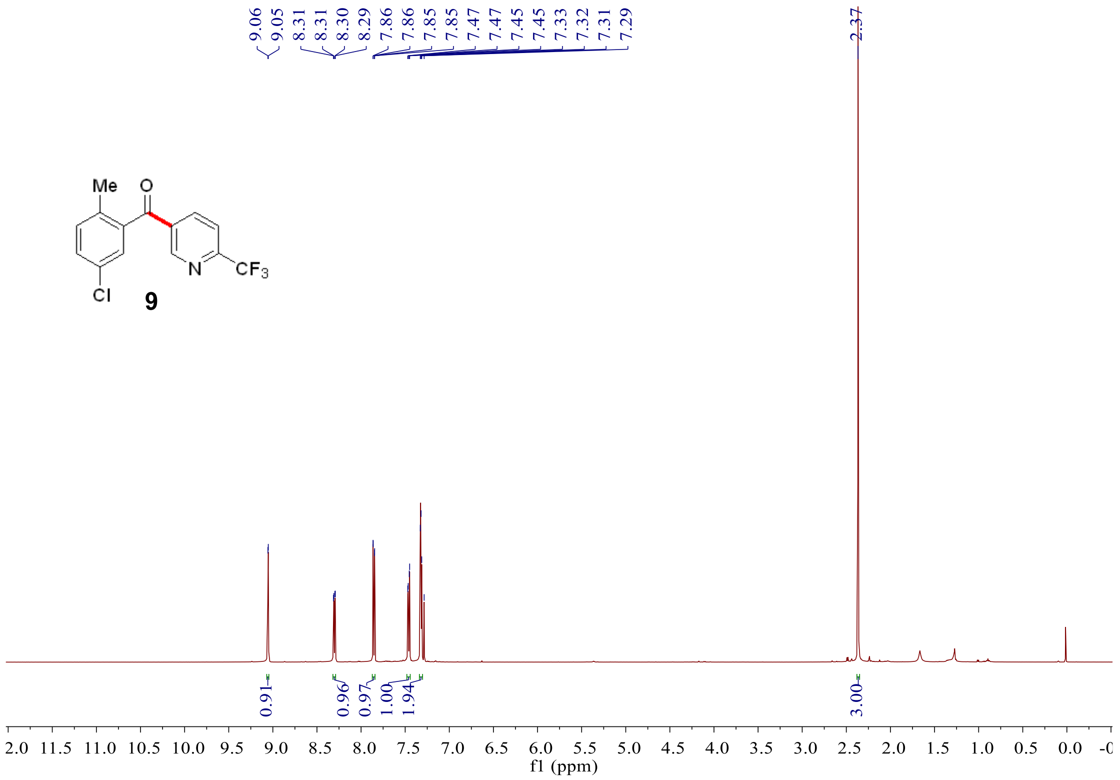

Supplementary Figure 31. ^{1}H NMR spectra for compound **9**

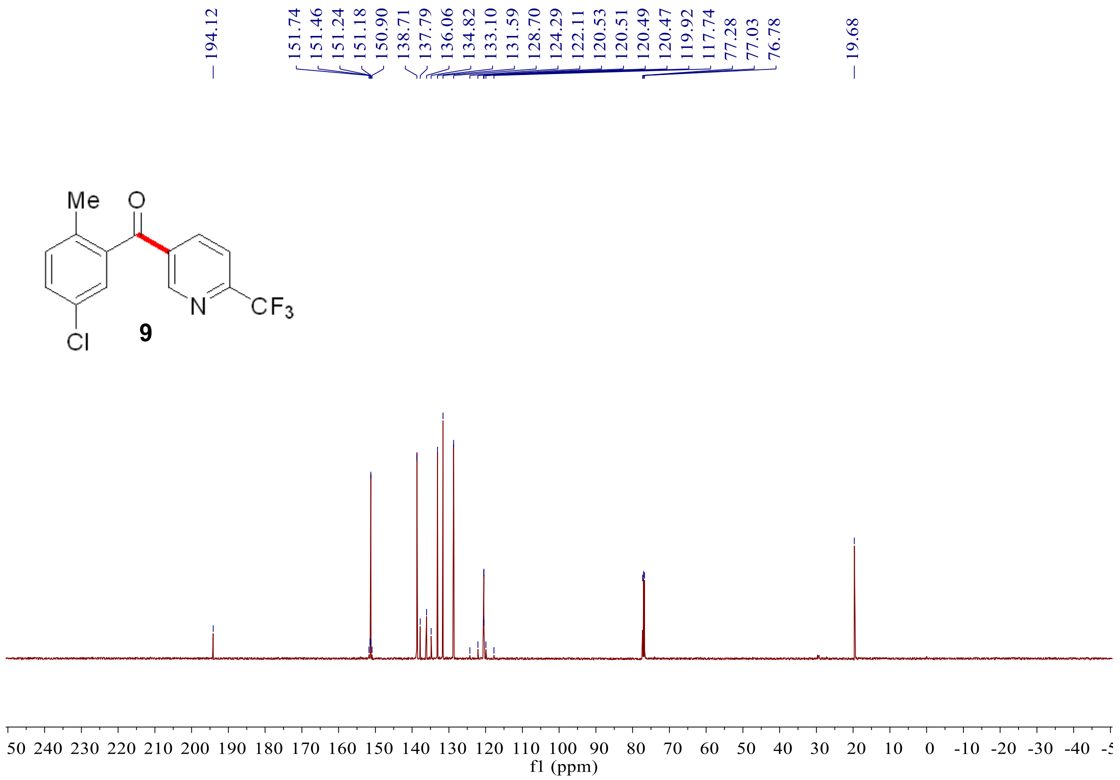

Supplementary Figure 32. ^{13}C NMR spectra for compound **9**

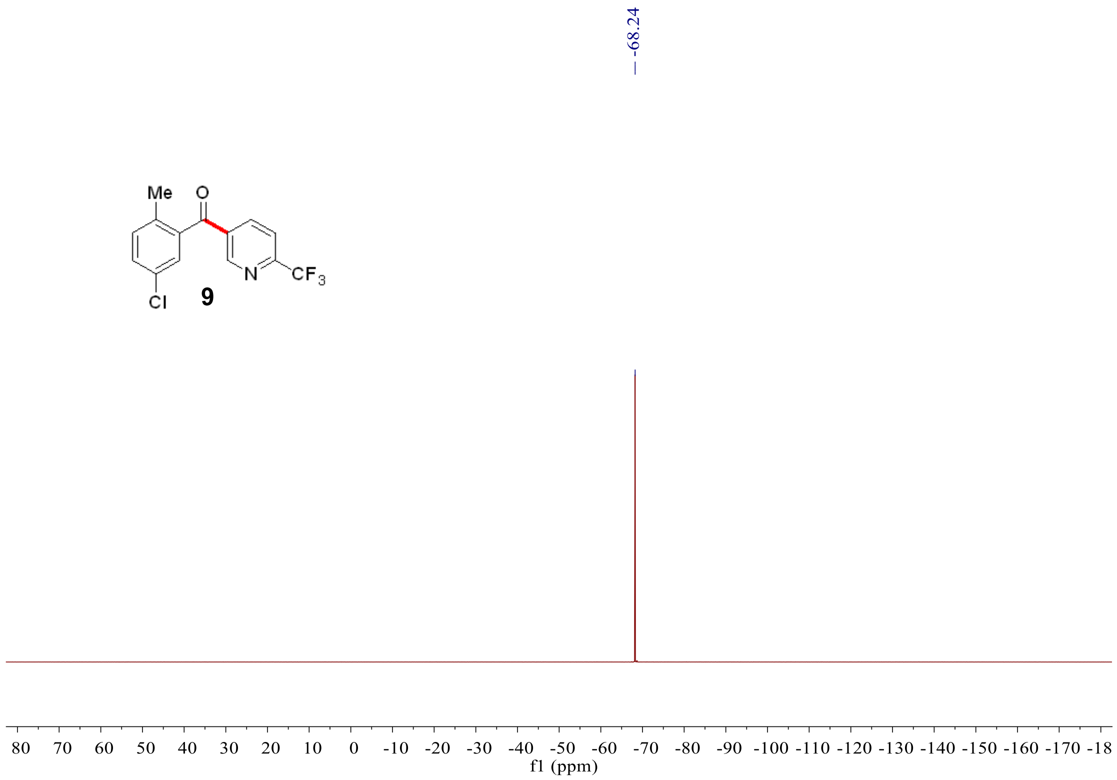

Supplementary Figure 33. ^{19}F NMR spectra for compound **9**

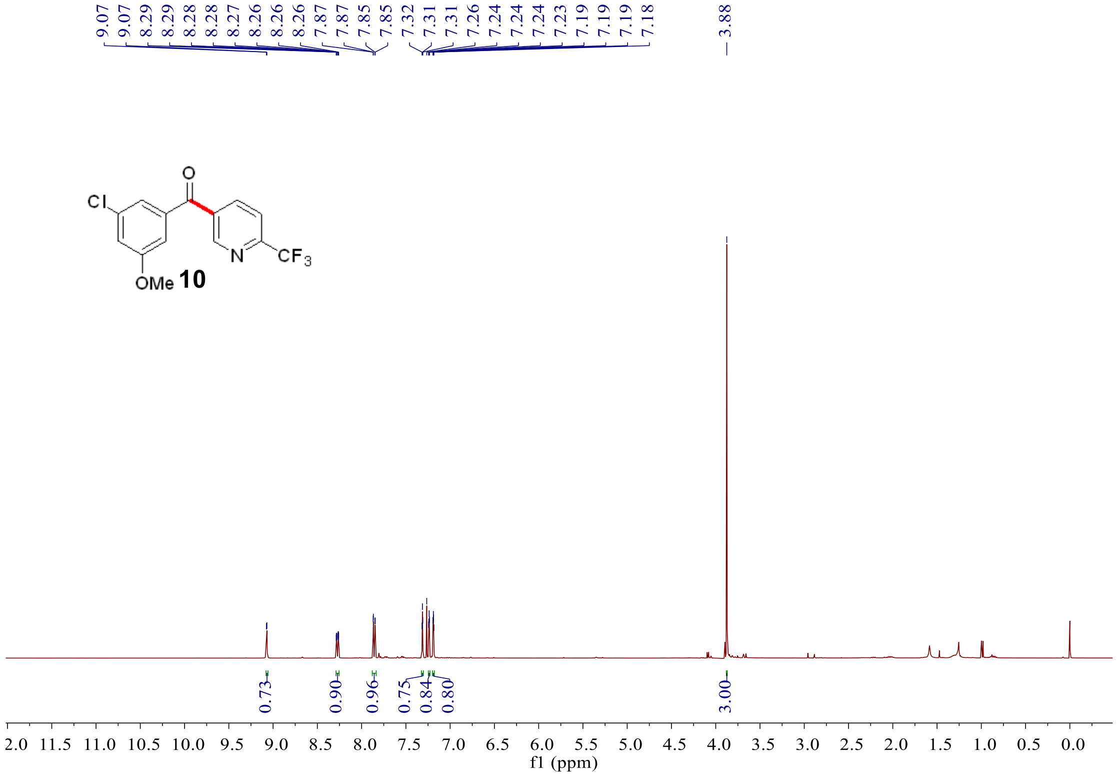

Supplementary Figure 34. ^{1}H NMR spectra for compound **10**

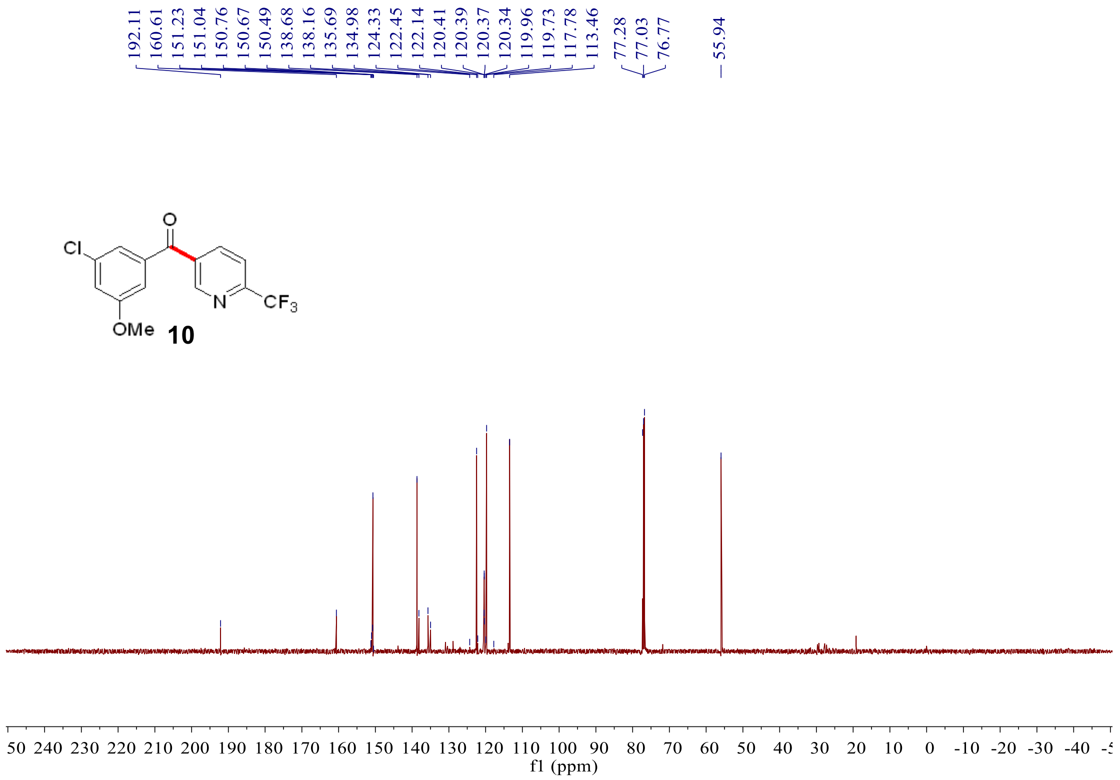

Supplementary Figure 35. ^{13}C NMR spectra for compound **10**

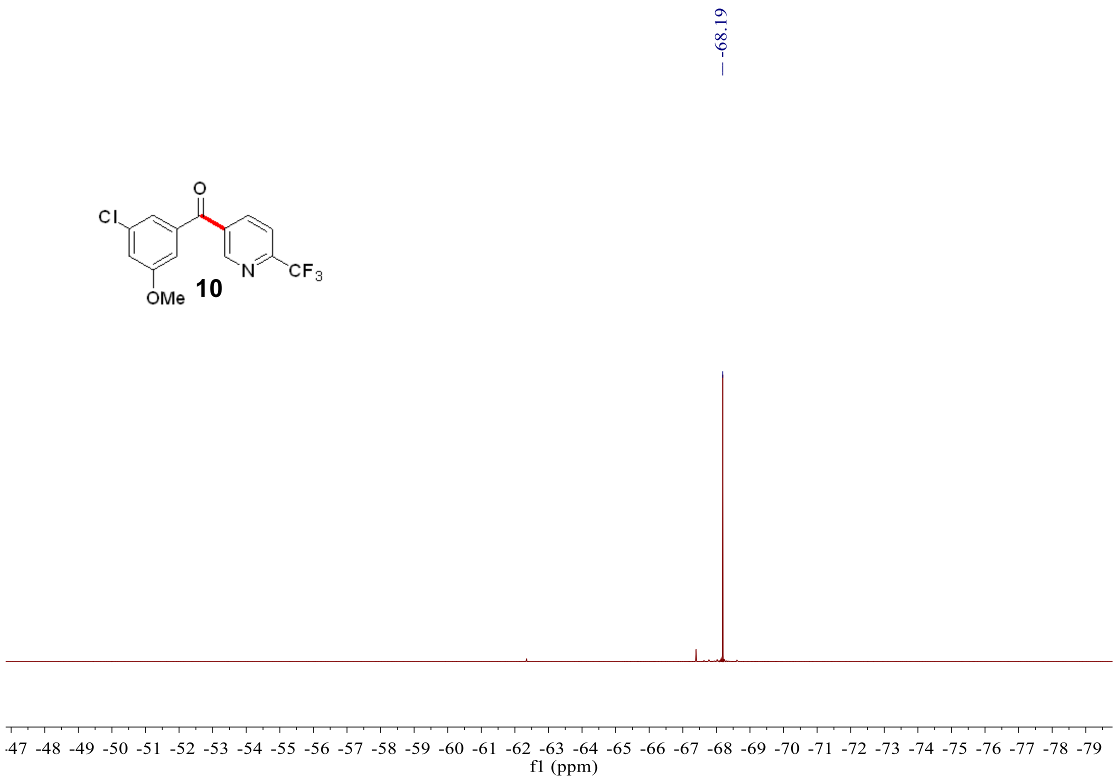

Supplementary Figure 36. ^{19}F NMR spectra for compound **10**

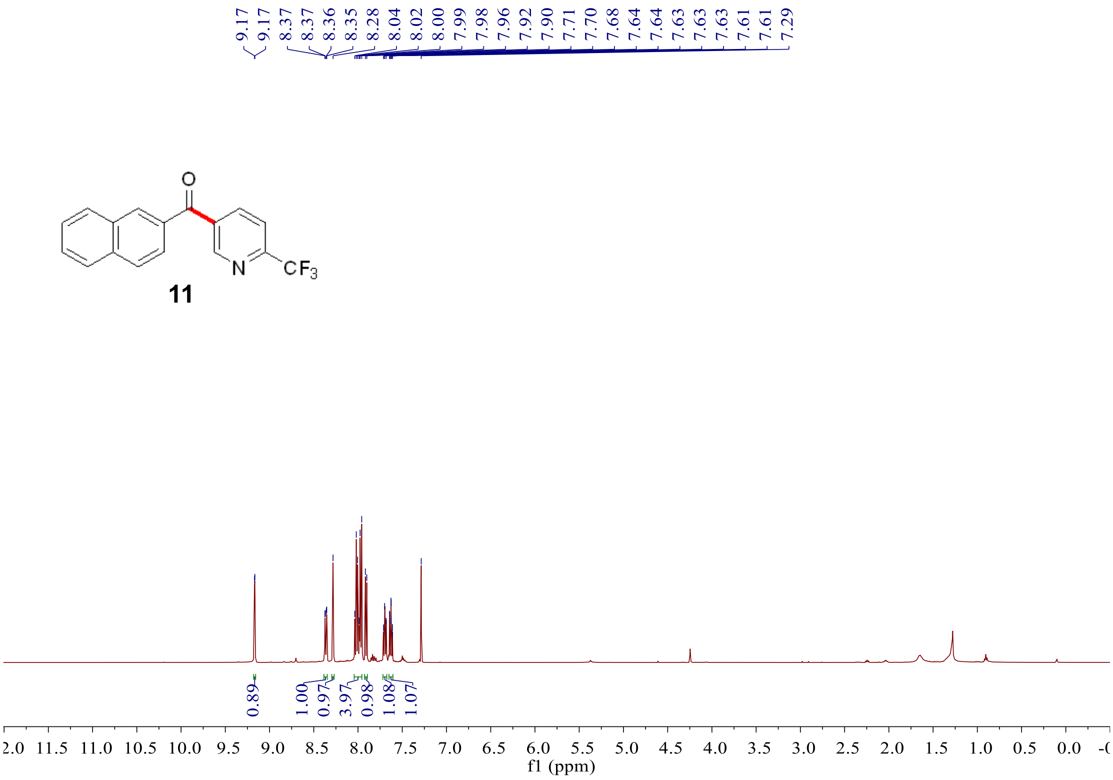

Supplementary Figure 37. ^{1}H NMR spectra for compound **11**

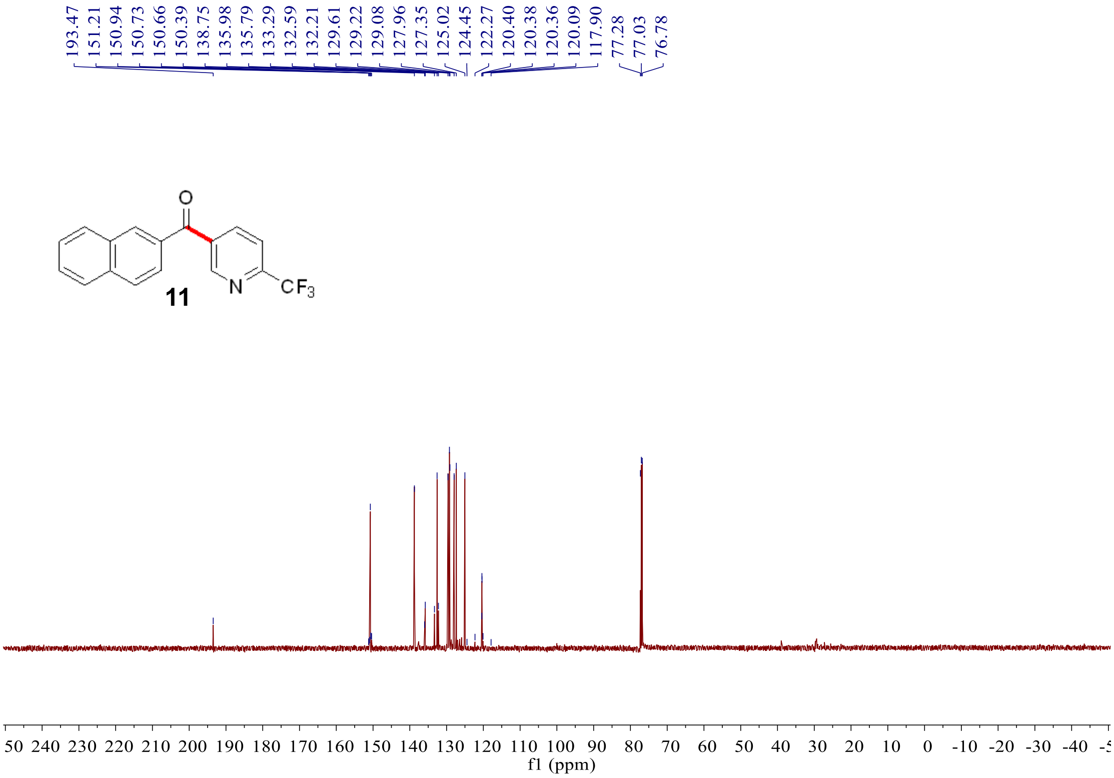

Supplementary Figure 38. ^{13}C NMR spectra for compound **11**

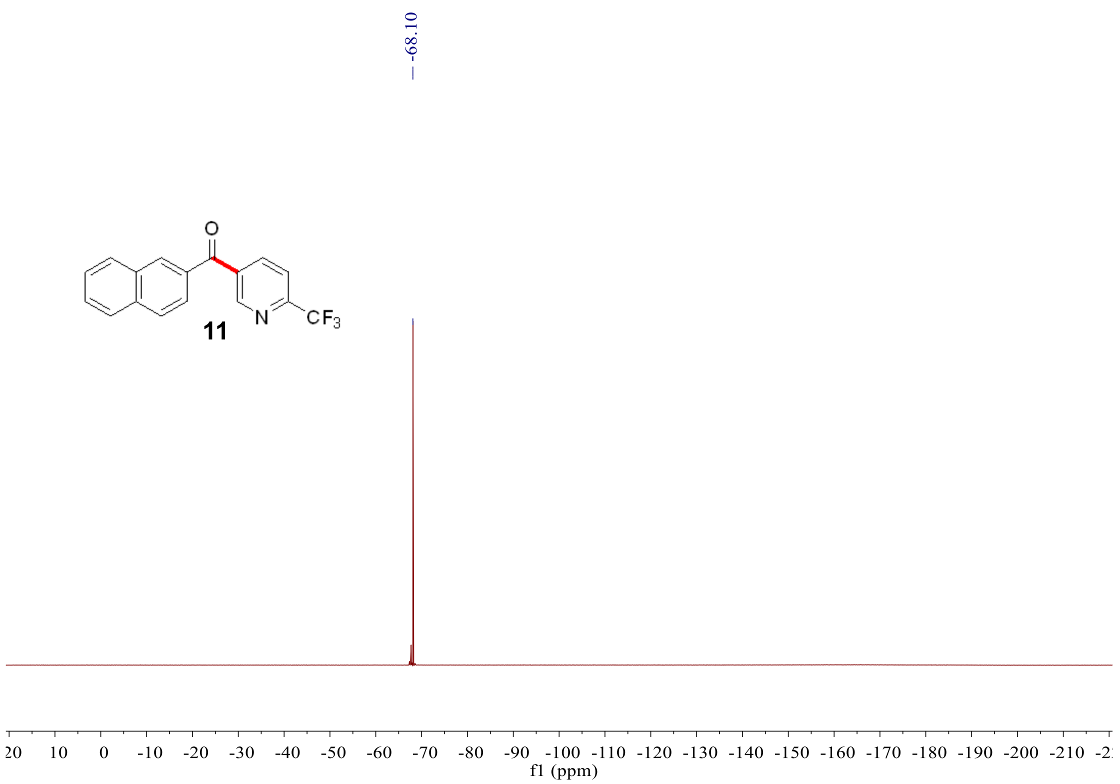

Supplementary Figure 39. ^{19}F NMR spectra for compound **11**

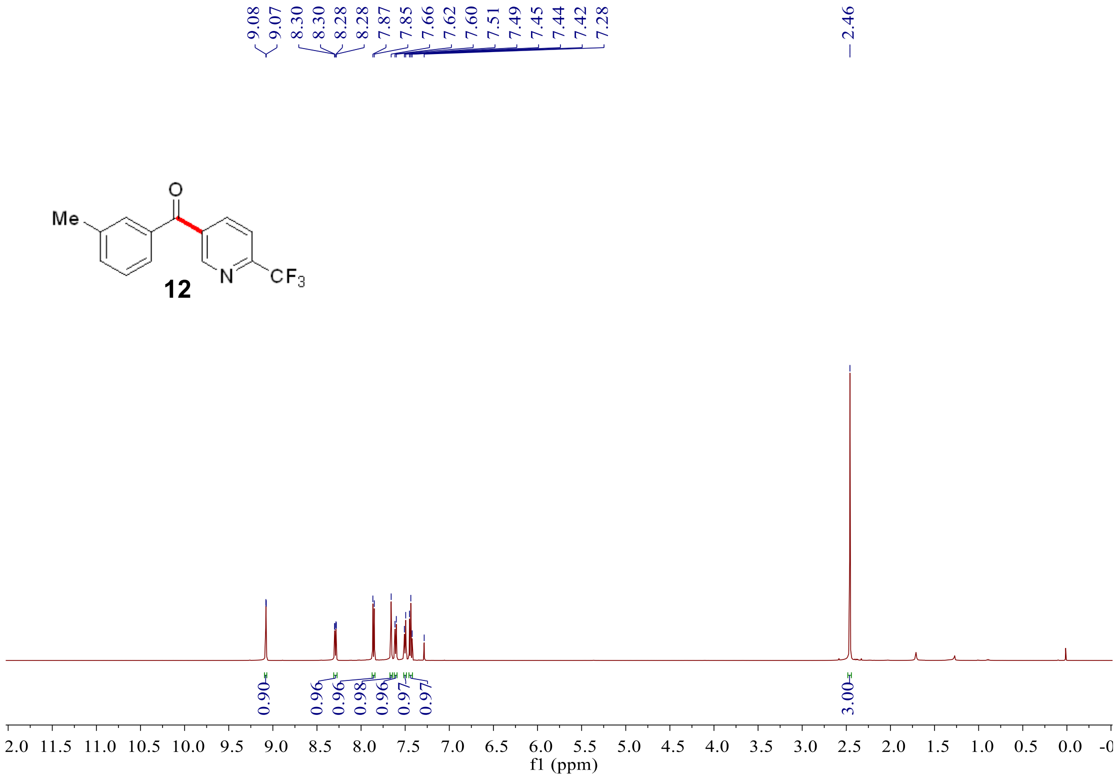

Supplementary Figure 40. ^{1}H NMR spectra for compound **12**

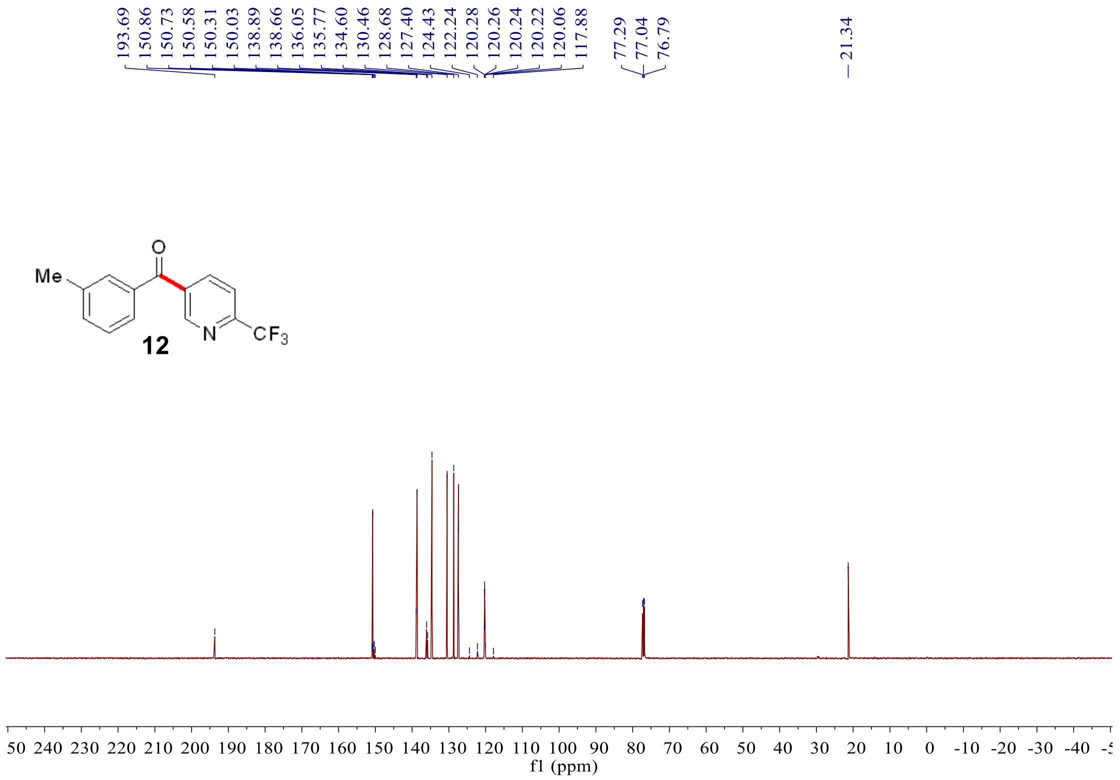

Supplementary Figure 41. ^{13}C NMR spectra for compound **12**

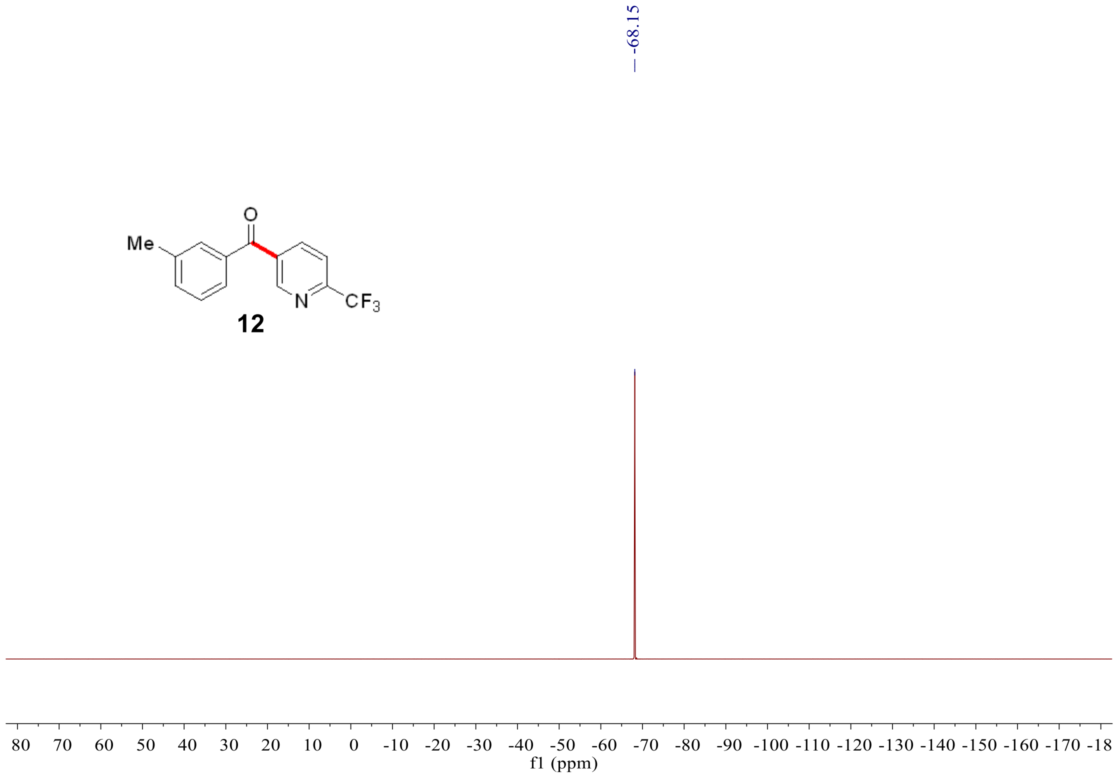

Supplementary Figure 42. ^{19}F NMR spectra for compound **12**

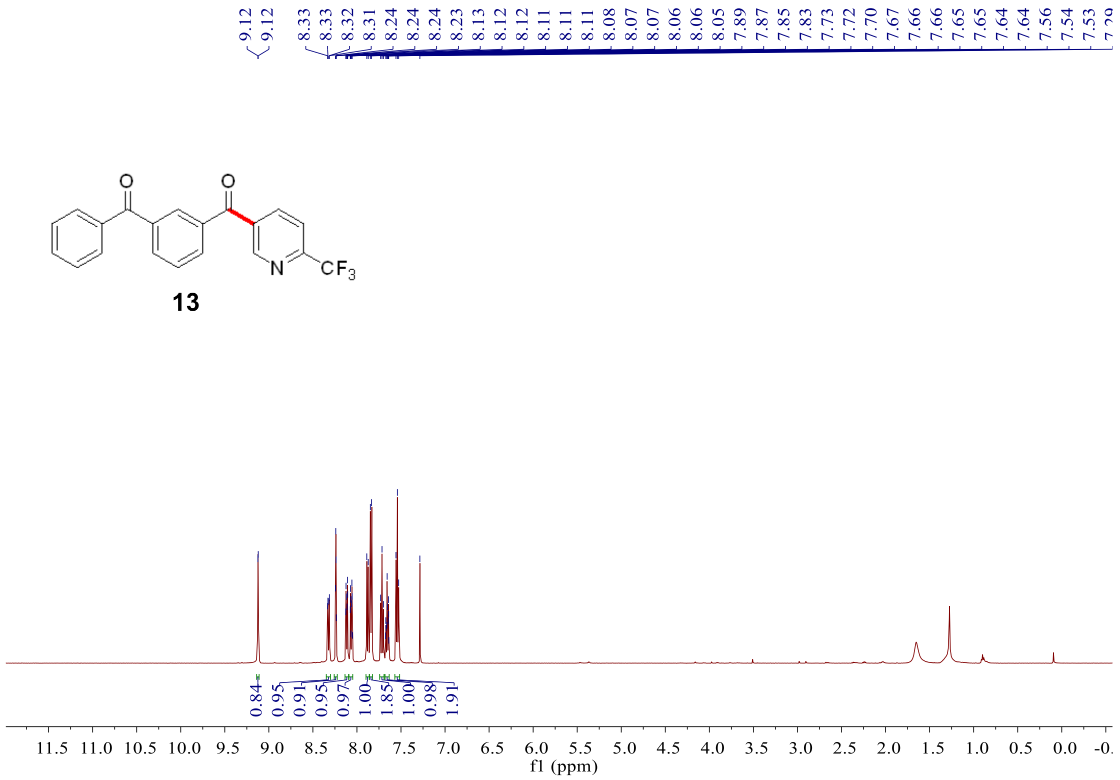

Supplementary Figure 43. ^{1}H NMR spectra for compound **13**

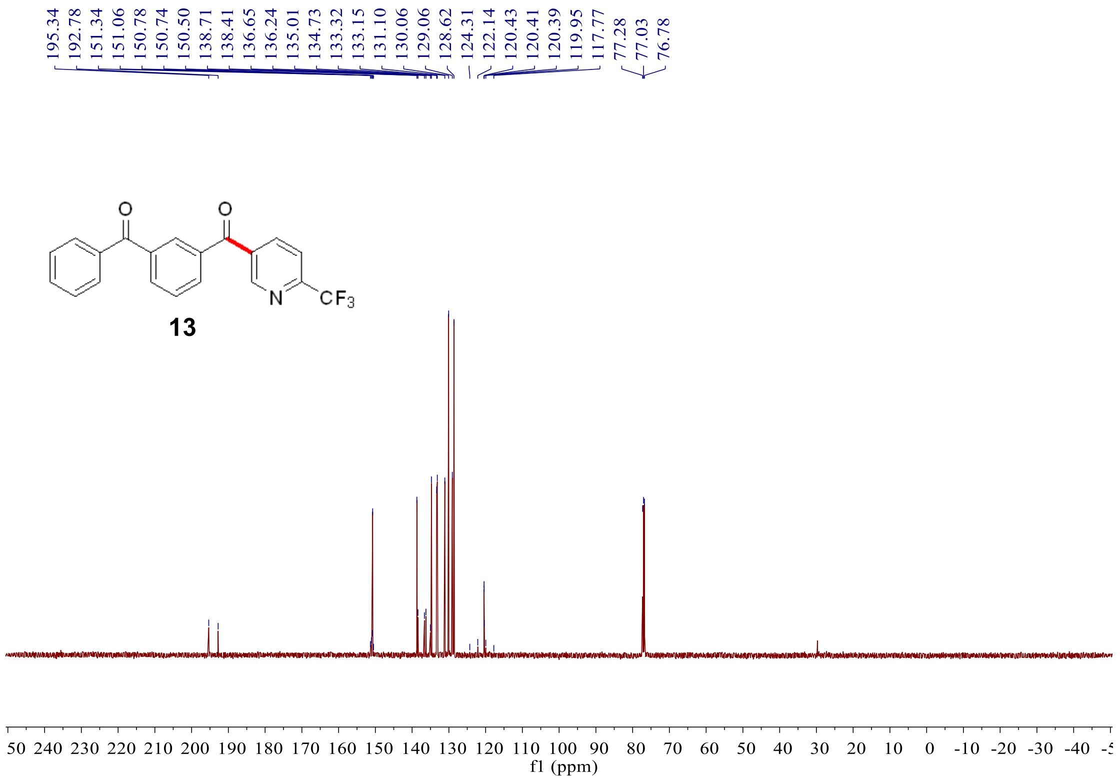

Supplementary Figure 44. ^{13}C NMR spectra for compound **13**

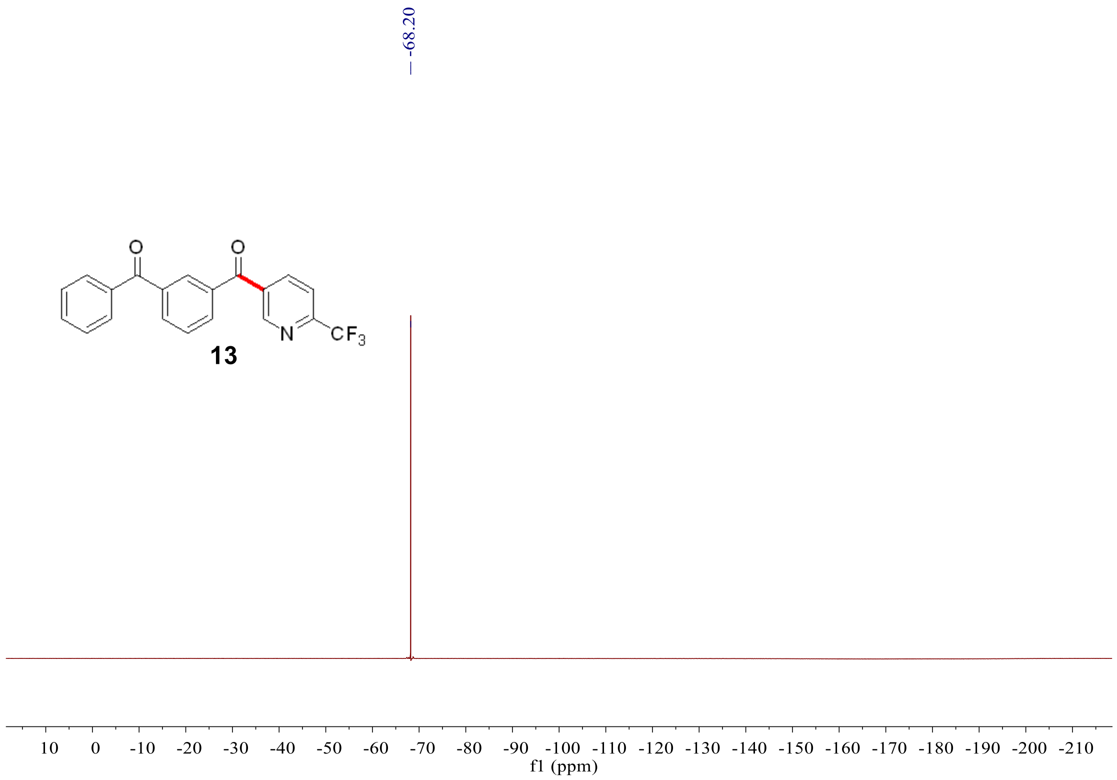

Supplementary Figure 45. ^{19}F NMR spectra for compound **13**

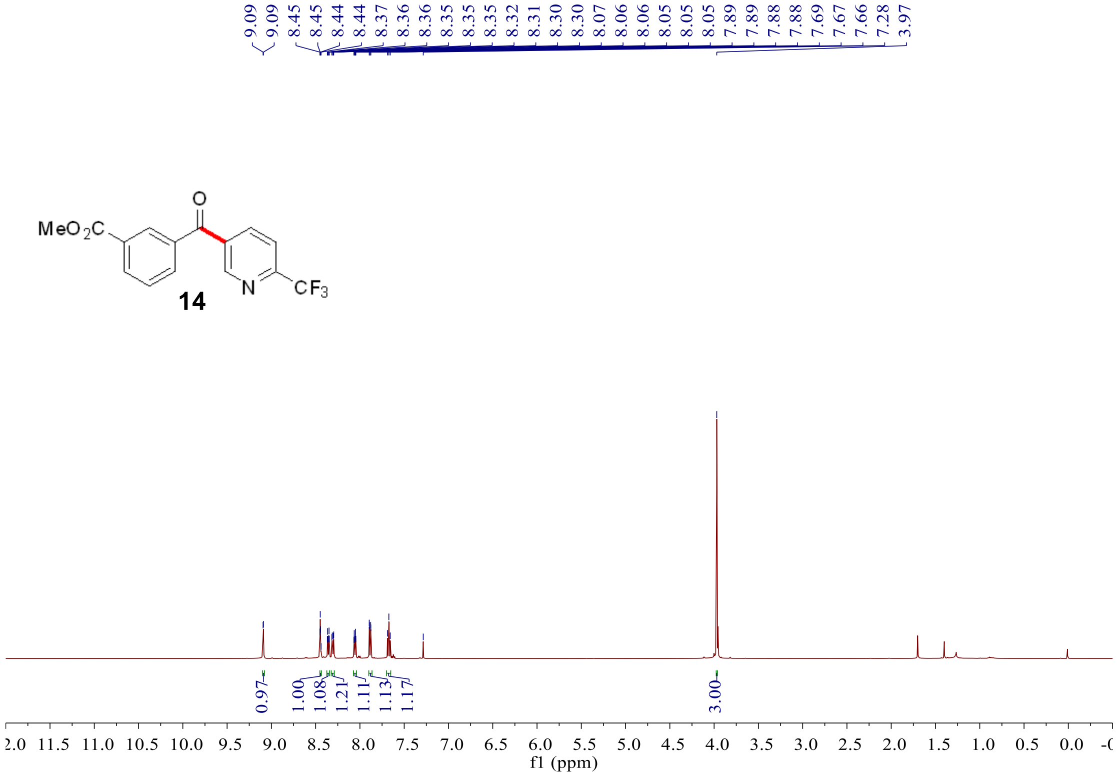

Supplementary Figure 46. ^{1}H NMR spectra for compound **14**

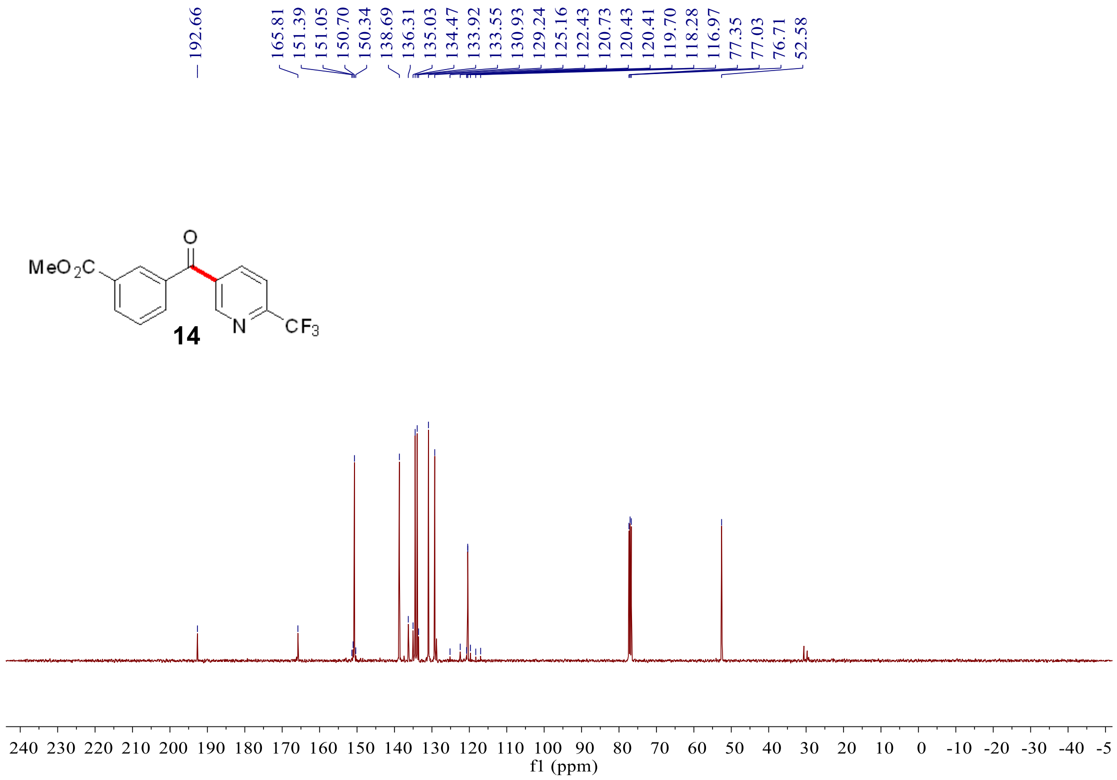

Supplementary Figure 47. ^{13}C NMR spectra for compound **14**

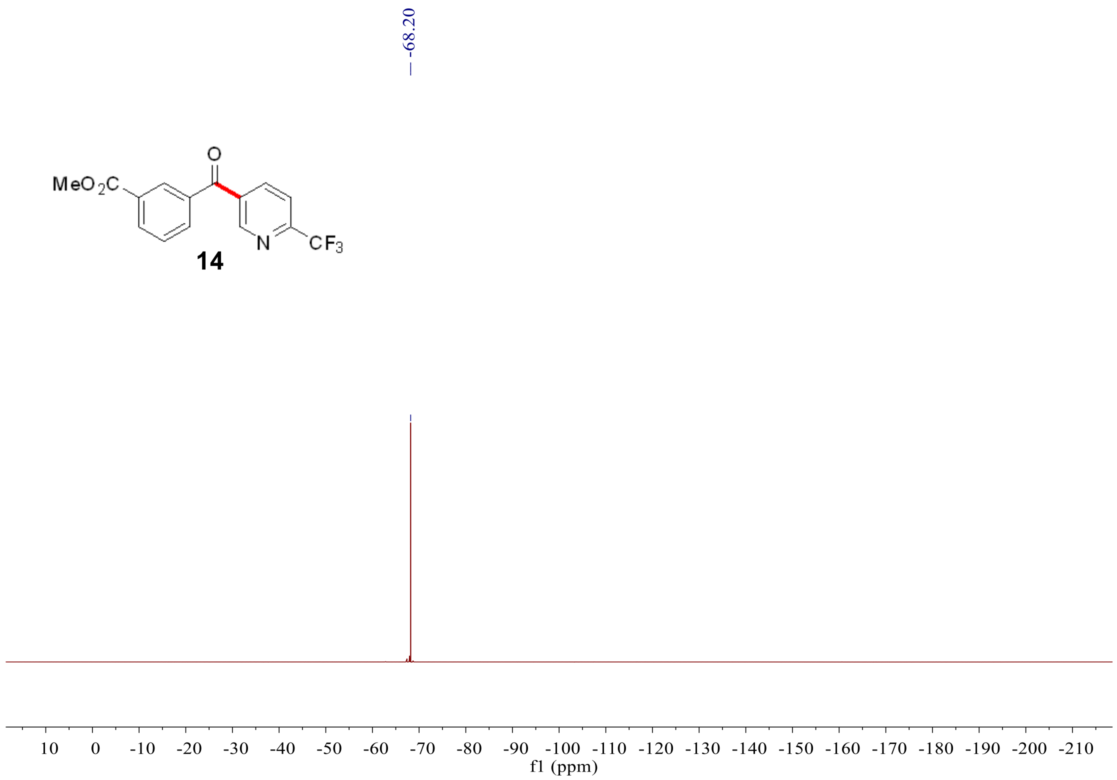

Supplementary Figure 48. ^{19}F NMR spectra for compound **14**

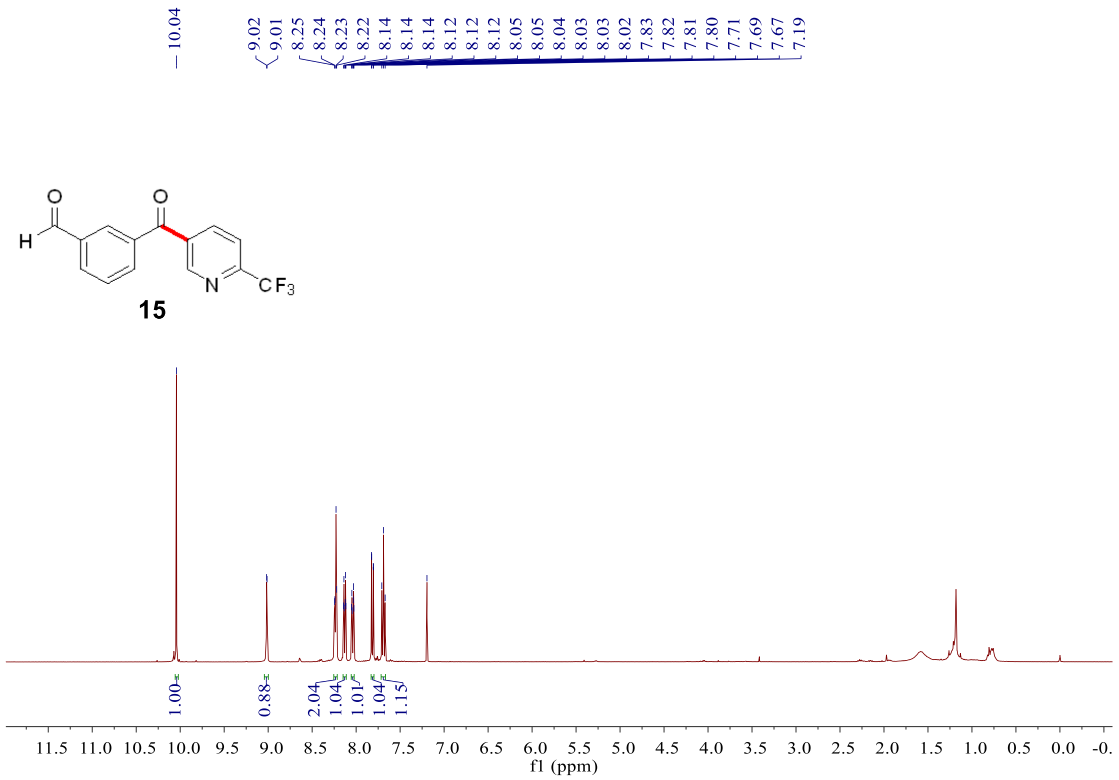

Supplementary Figure 49. ^{1}H NMR spectra for compound **15**

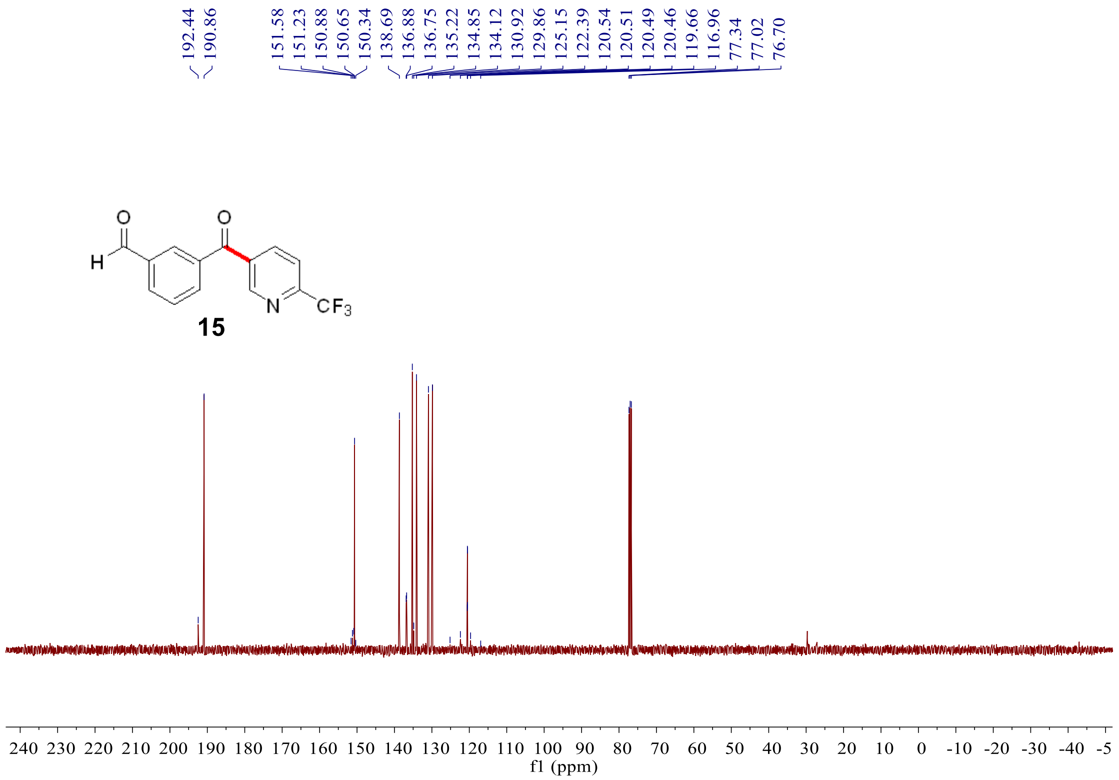

Supplementary Figure 50. ^{13}C NMR spectra for compound **15**

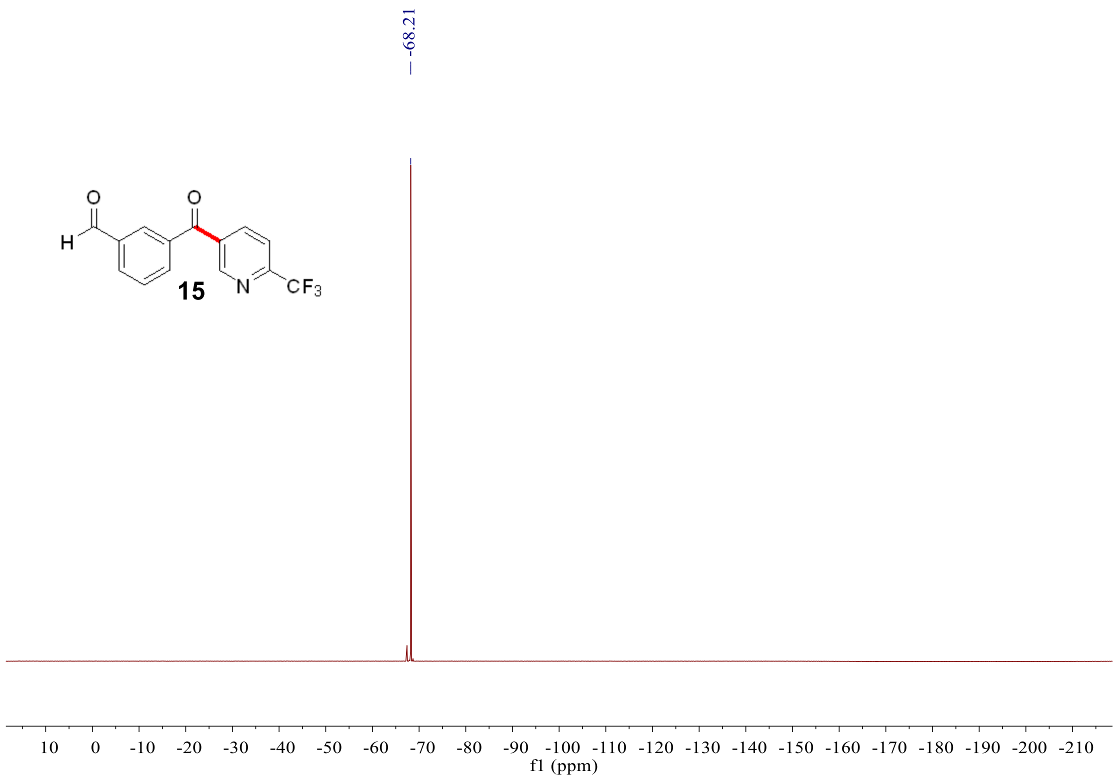

Supplementary Figure 51. ^{19}F NMR spectra for compound **15**

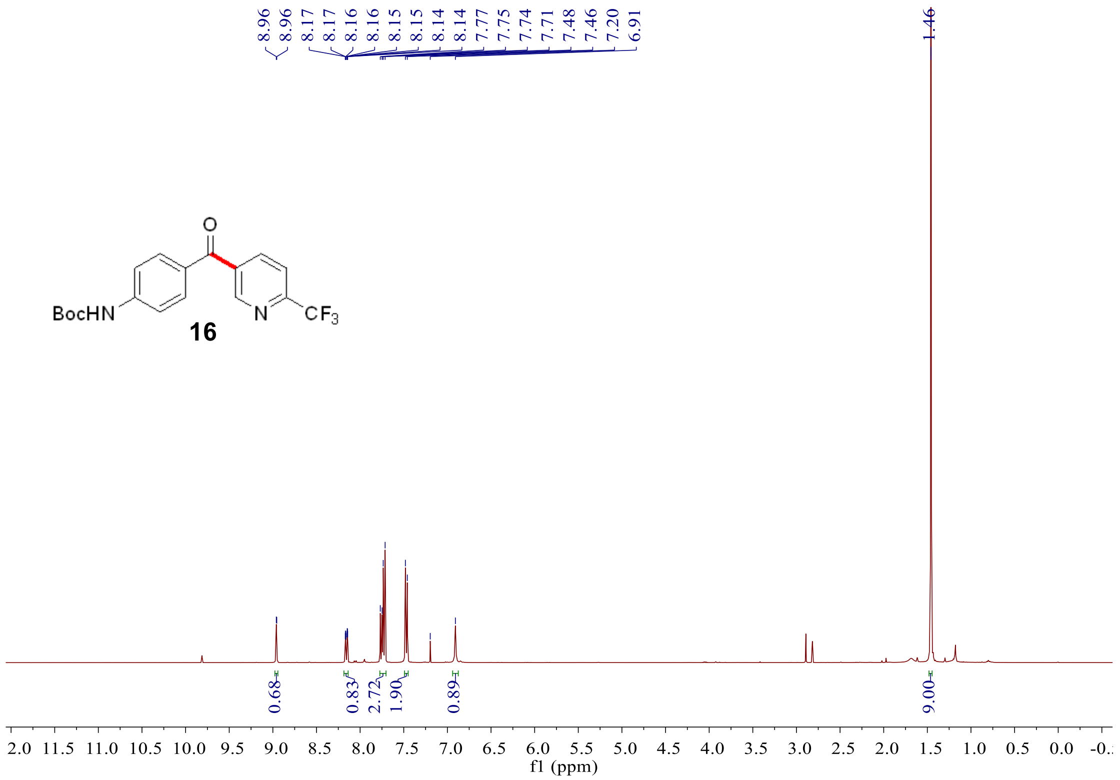

Supplementary Figure 52. ^{1}H NMR spectra for compound **16**

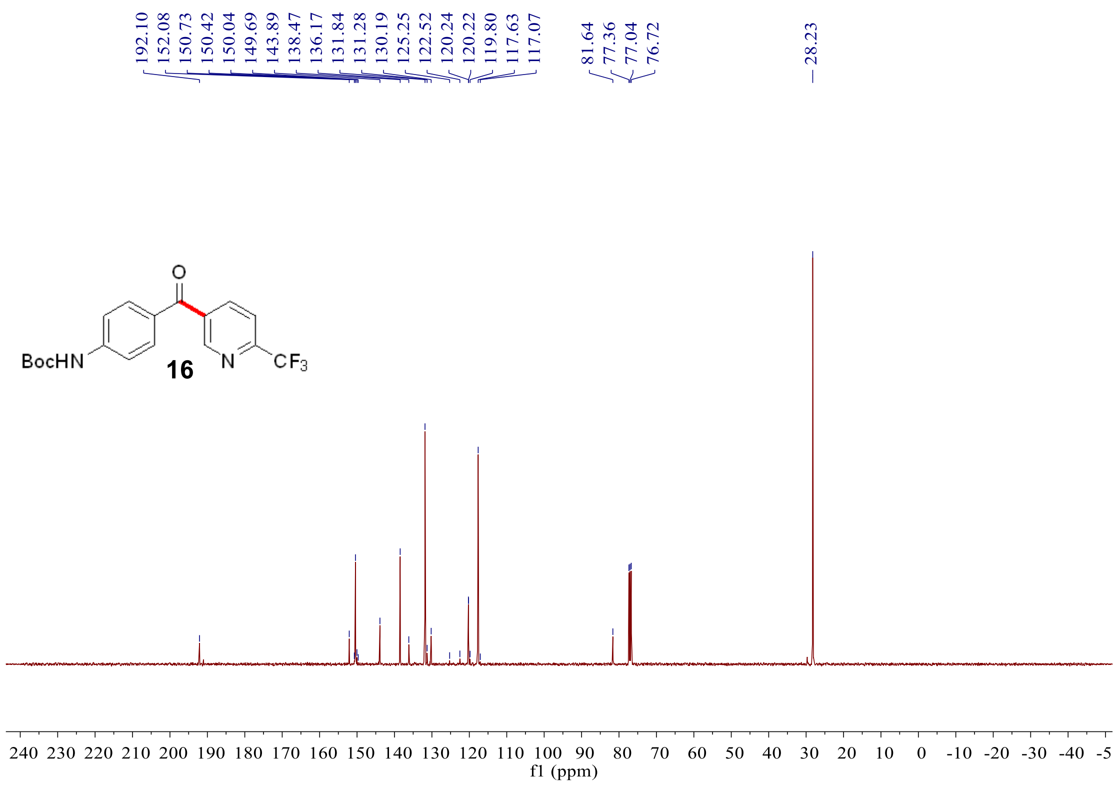

Supplementary Figure 53. ^{13}C NMR spectra for compound **16**

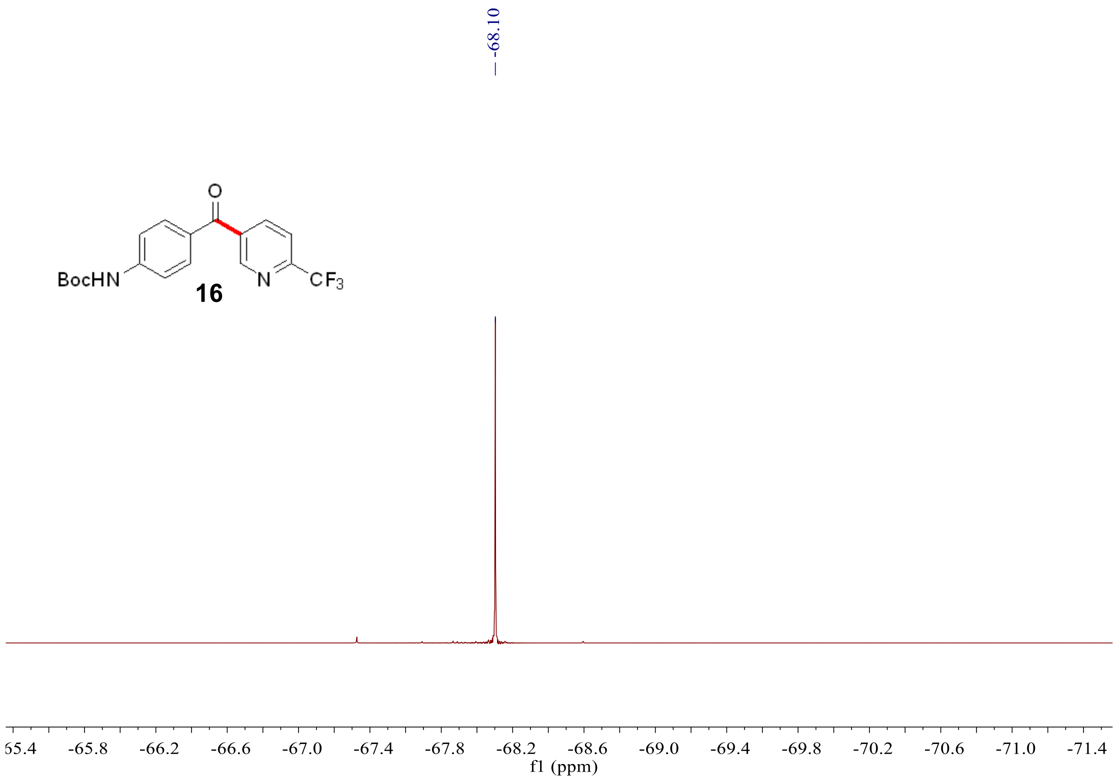

Supplementary Figure 54. ^{19}F NMR spectra for compound **16**

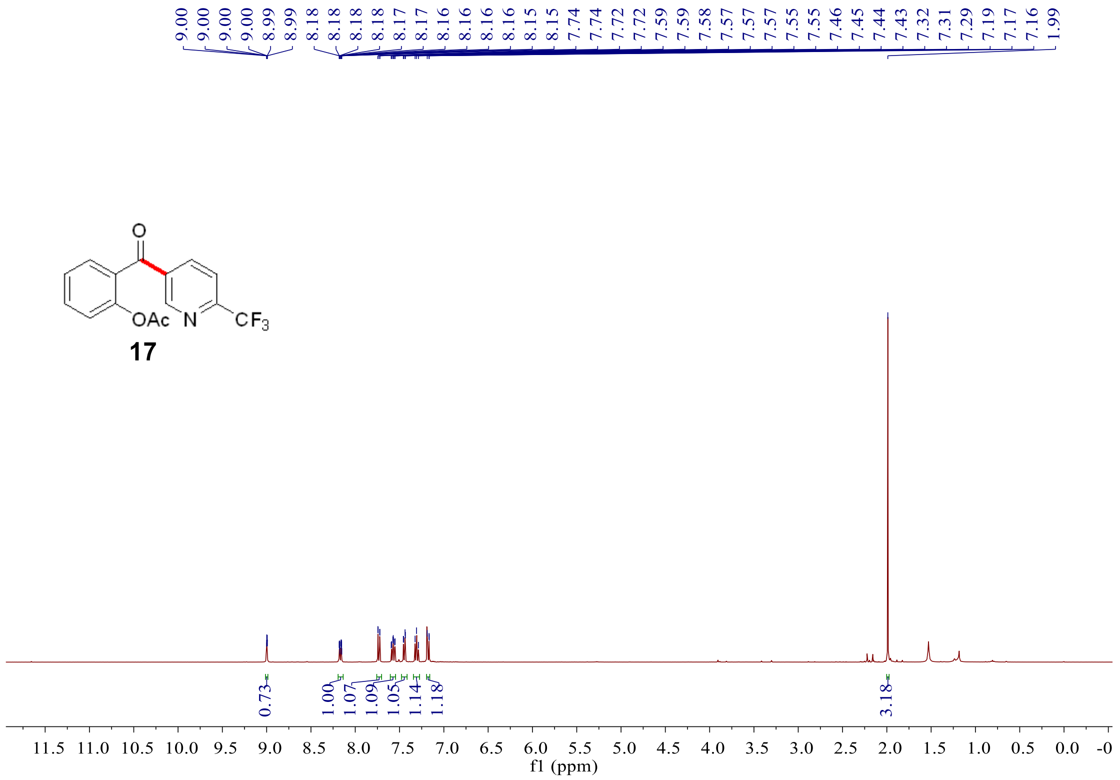

Supplementary Figure 55. ^{1}H NMR spectra for compound **17**

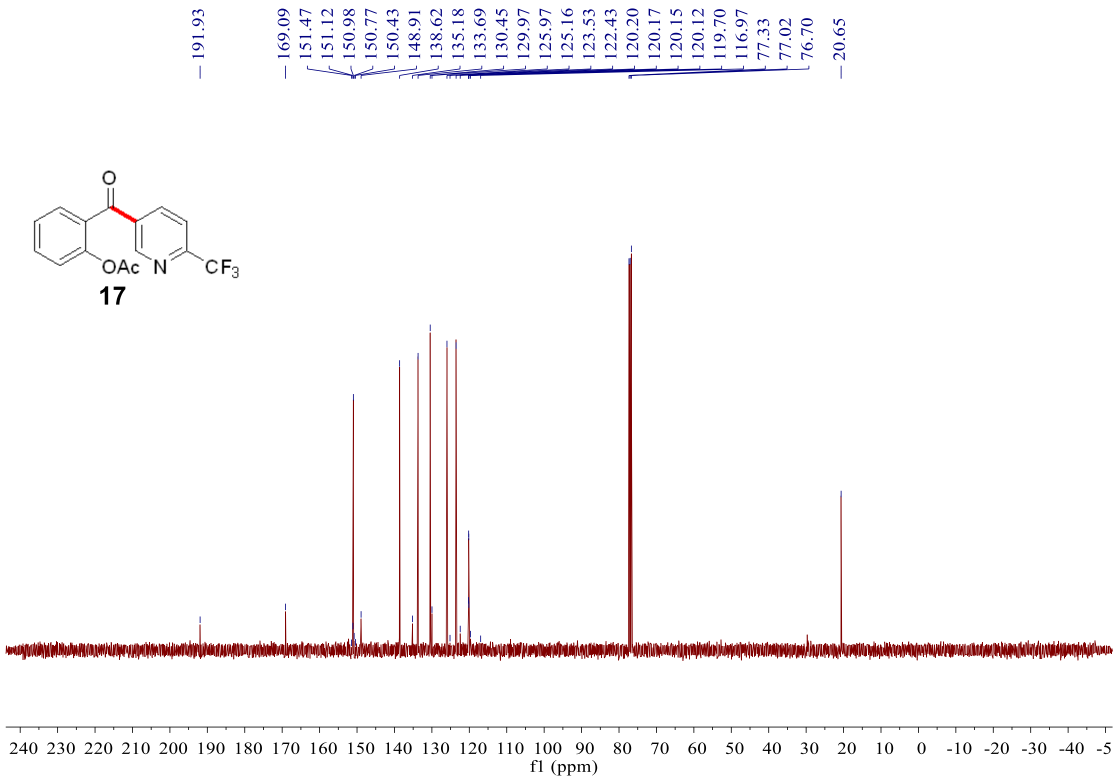

Supplementary Figure 56. ^{13}C NMR spectra for compound **17**

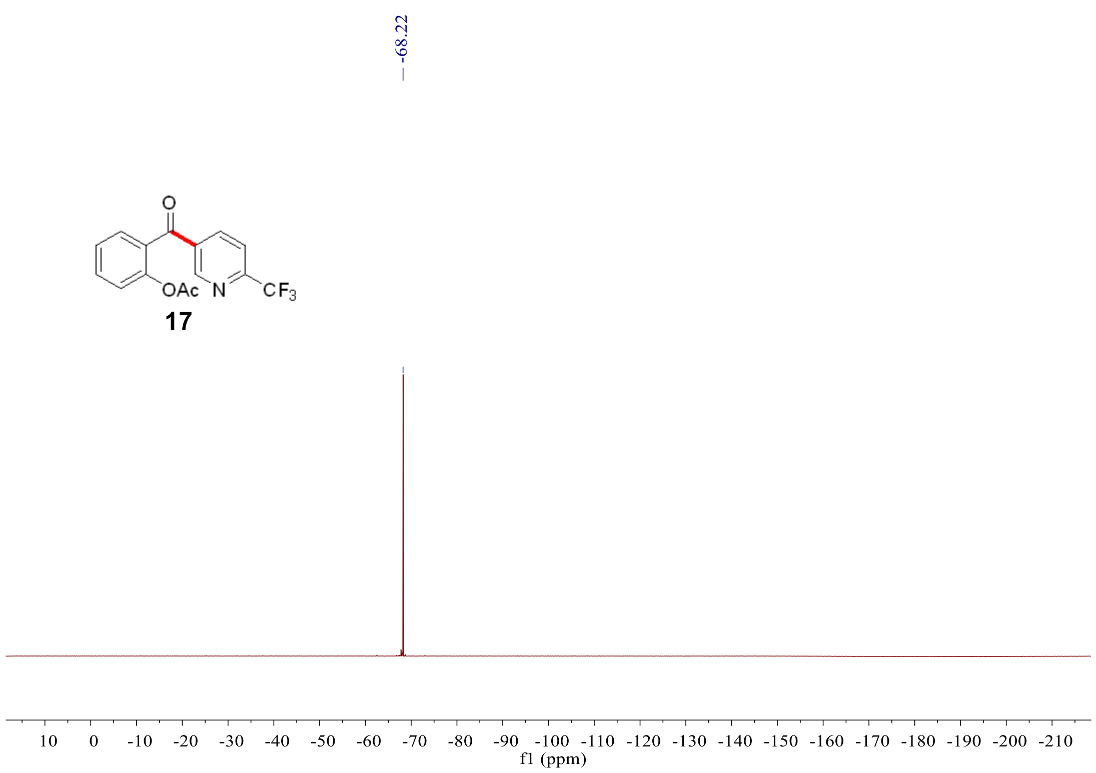

Supplementary Figure 57. ^{19}F NMR spectra for compound **17**

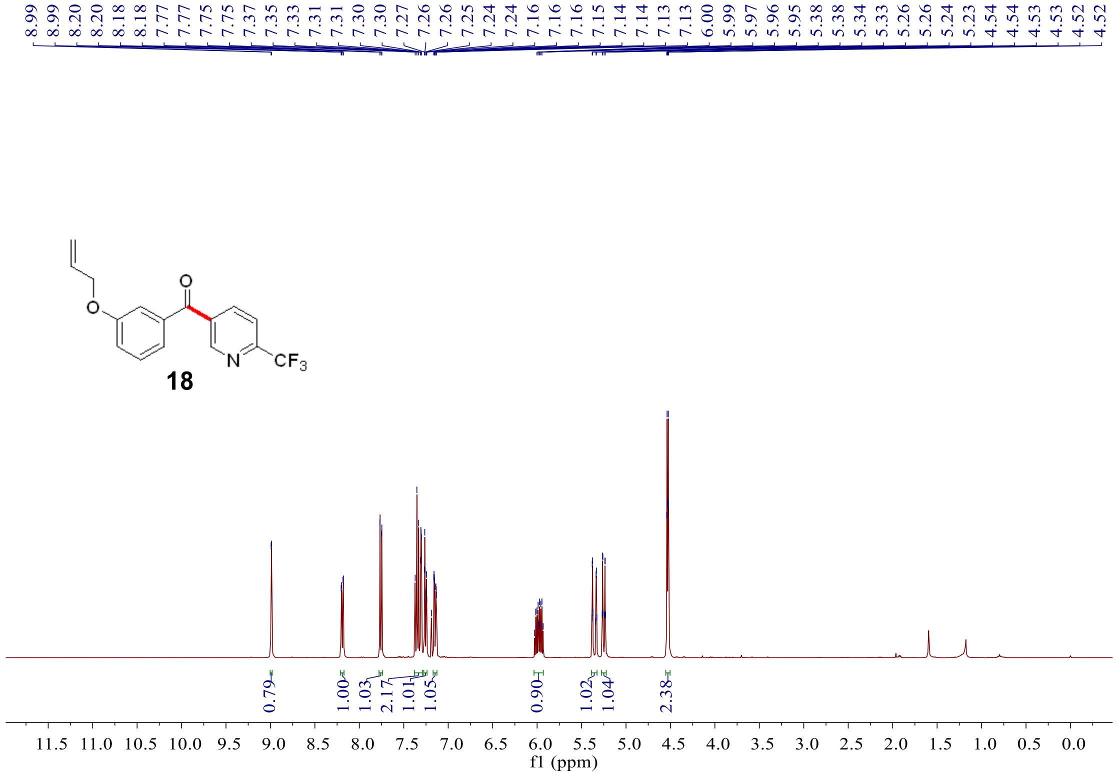

Supplementary Figure 58. ^{1}H NMR spectra for compound **18**

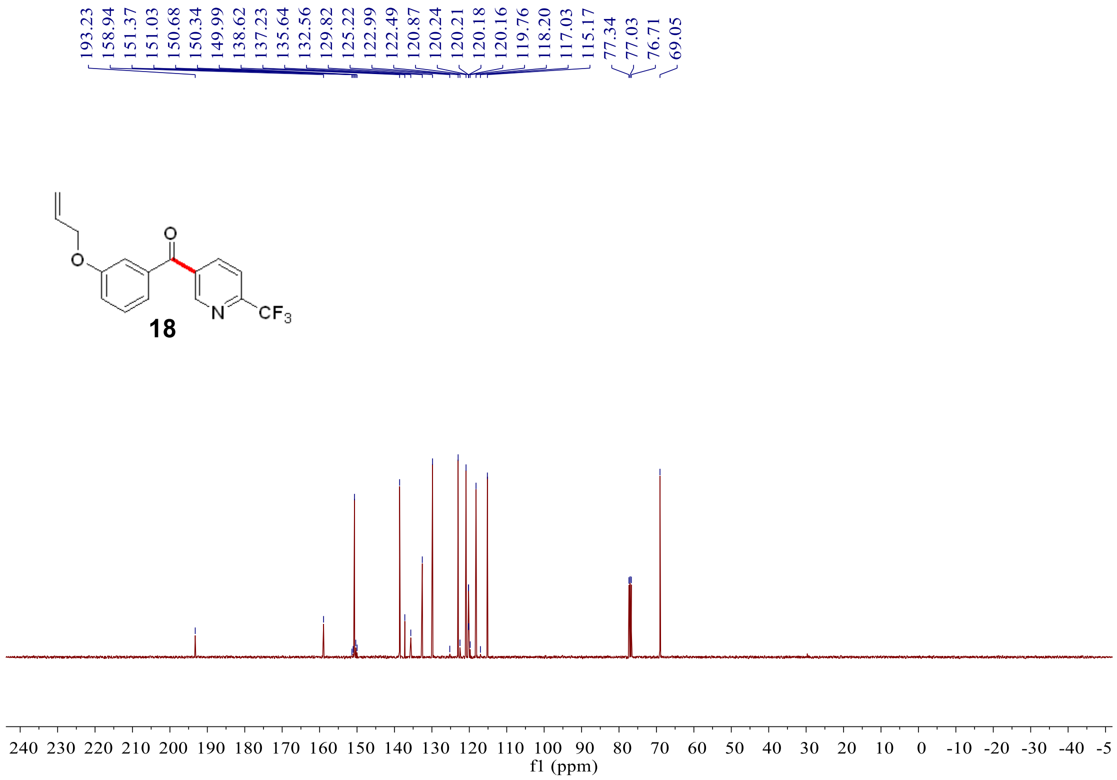

Supplementary Figure 59. ^{13}C NMR spectra for compound **18**

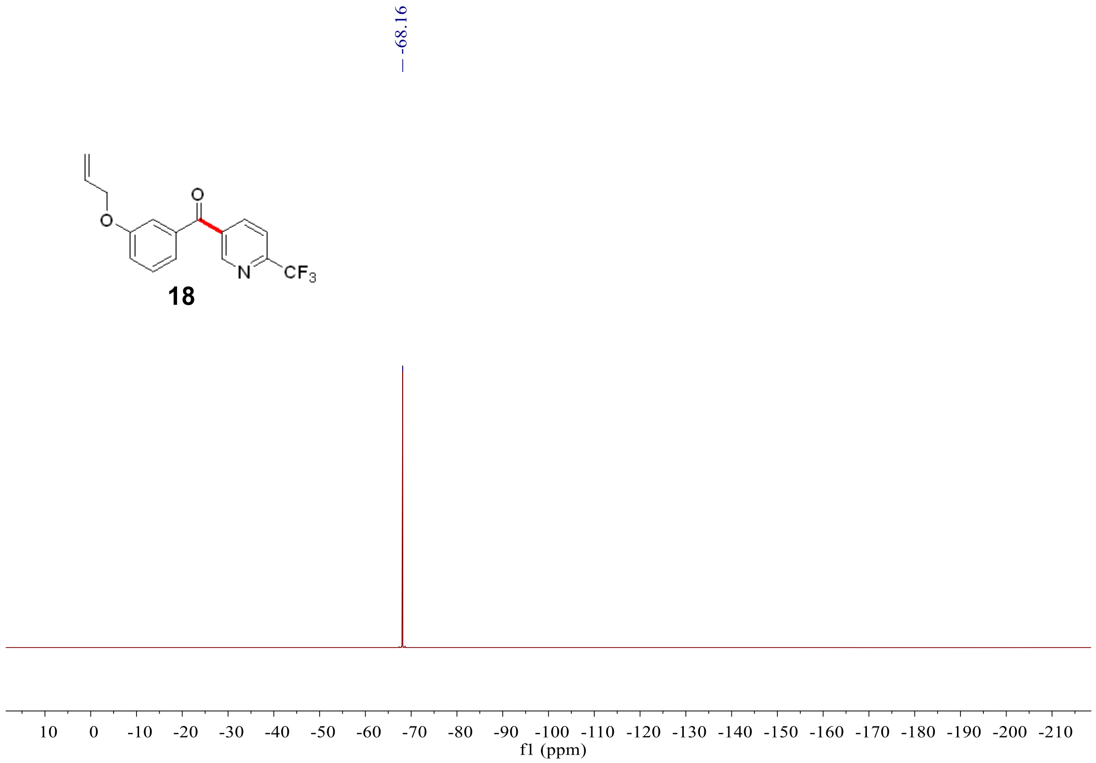

Supplementary Figure 60. ^{19}F NMR spectra for compound **18**

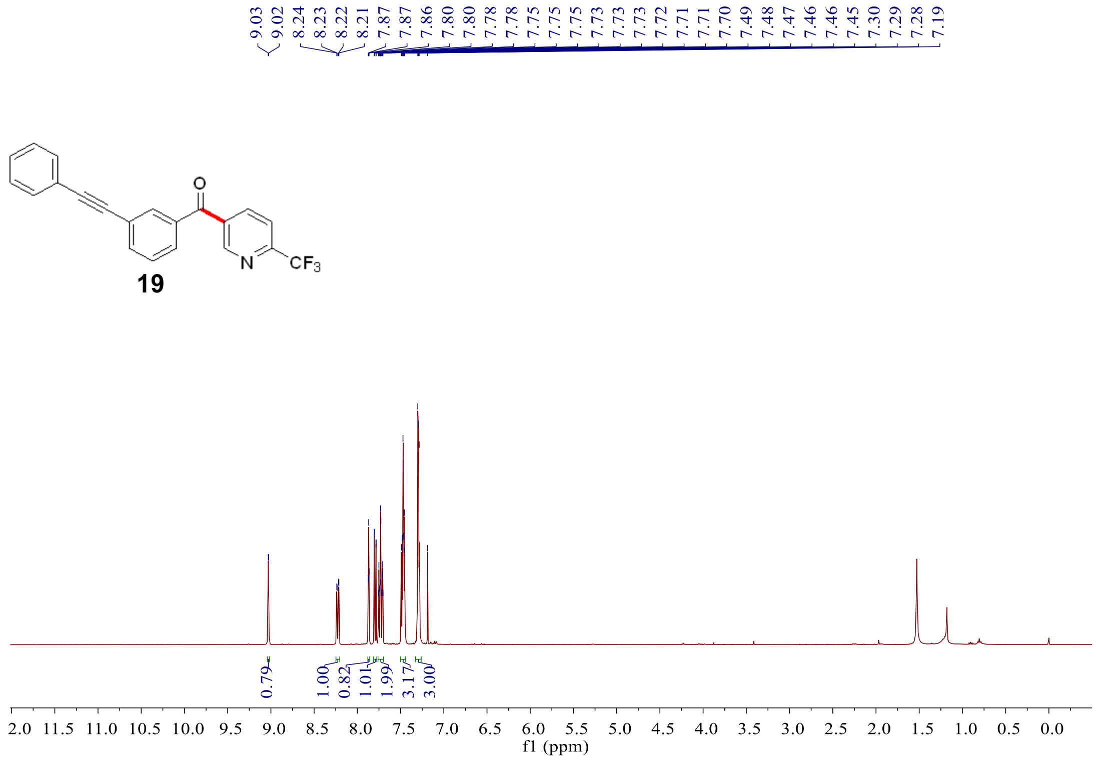

Supplementary Figure 61. ^{1}H NMR spectra for compound **19**

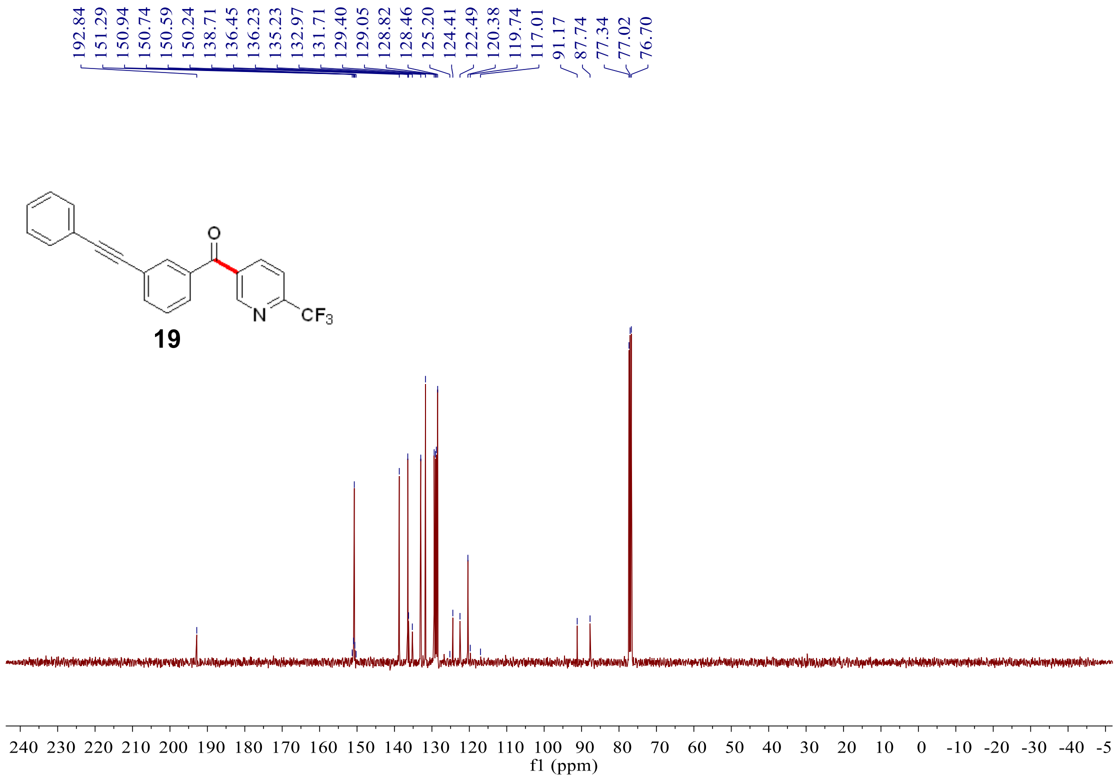

Supplementary Figure 62. ^{13}C NMR spectra for compound **19**

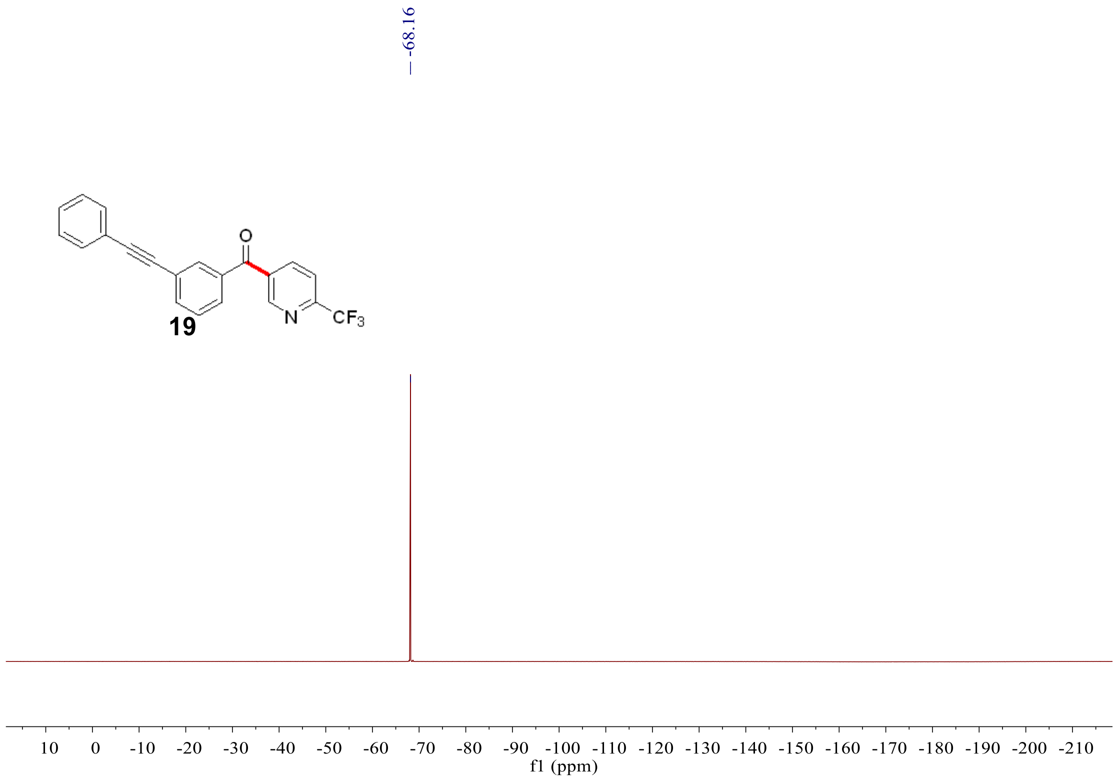

Supplementary Figure 63. ^{19}F NMR spectra for compound **20**

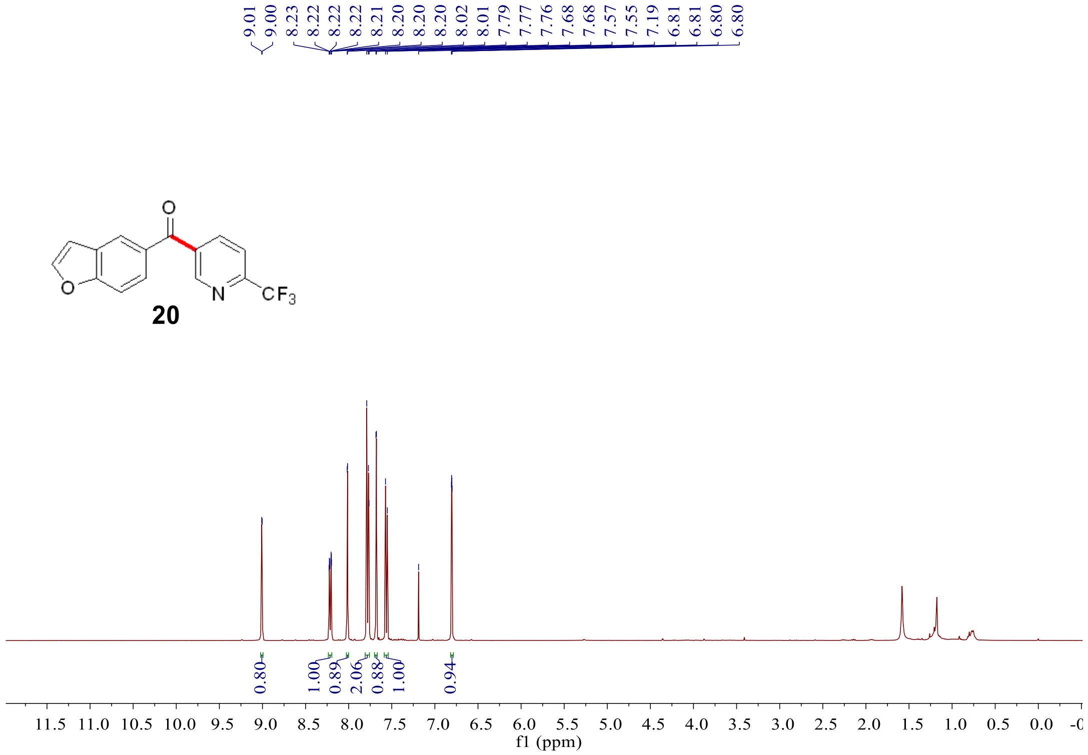

Supplementary Figure 64. ^{1}H NMR spectra for compound **20**

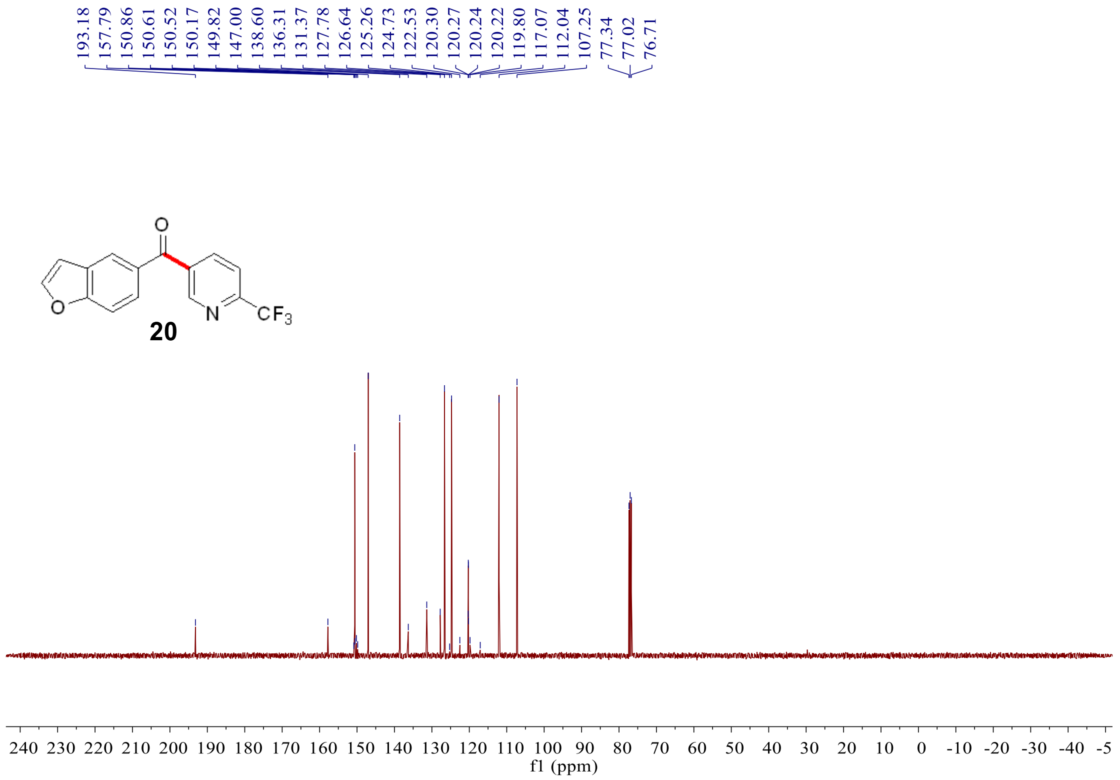

Supplementary Figure 65. ^{13}C NMR spectra for compound **20**

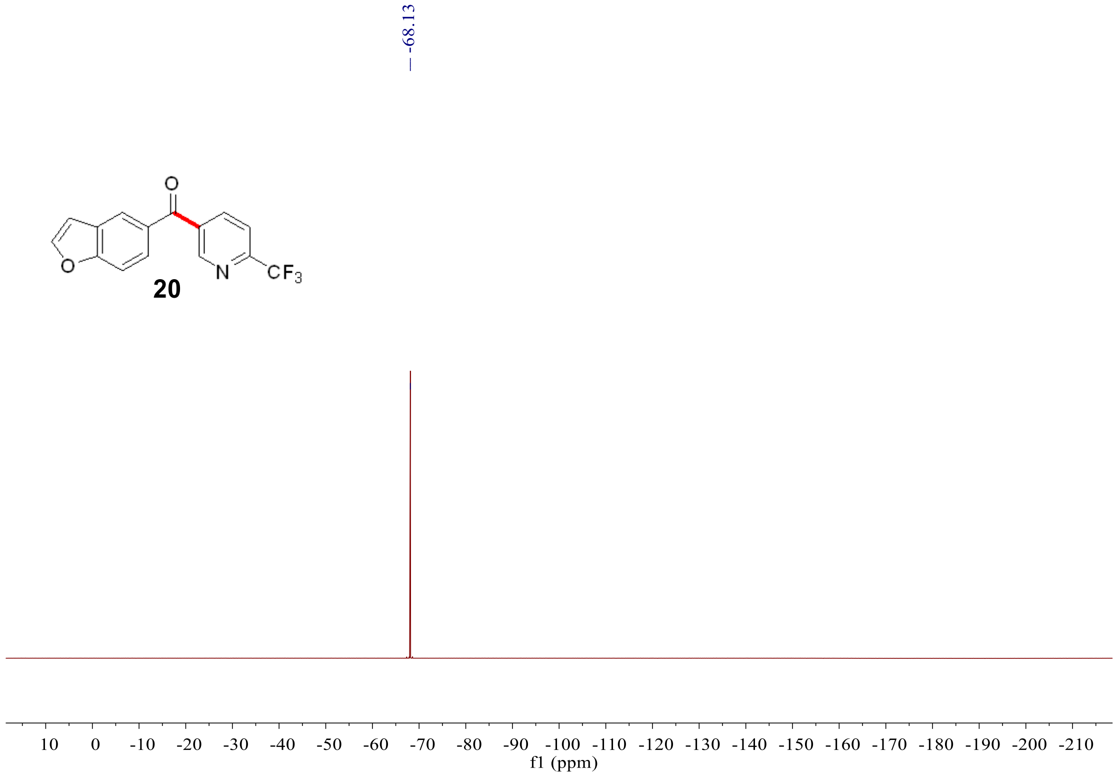

Supplementary Figure 66. ^{19}F NMR spectra for compound **20**

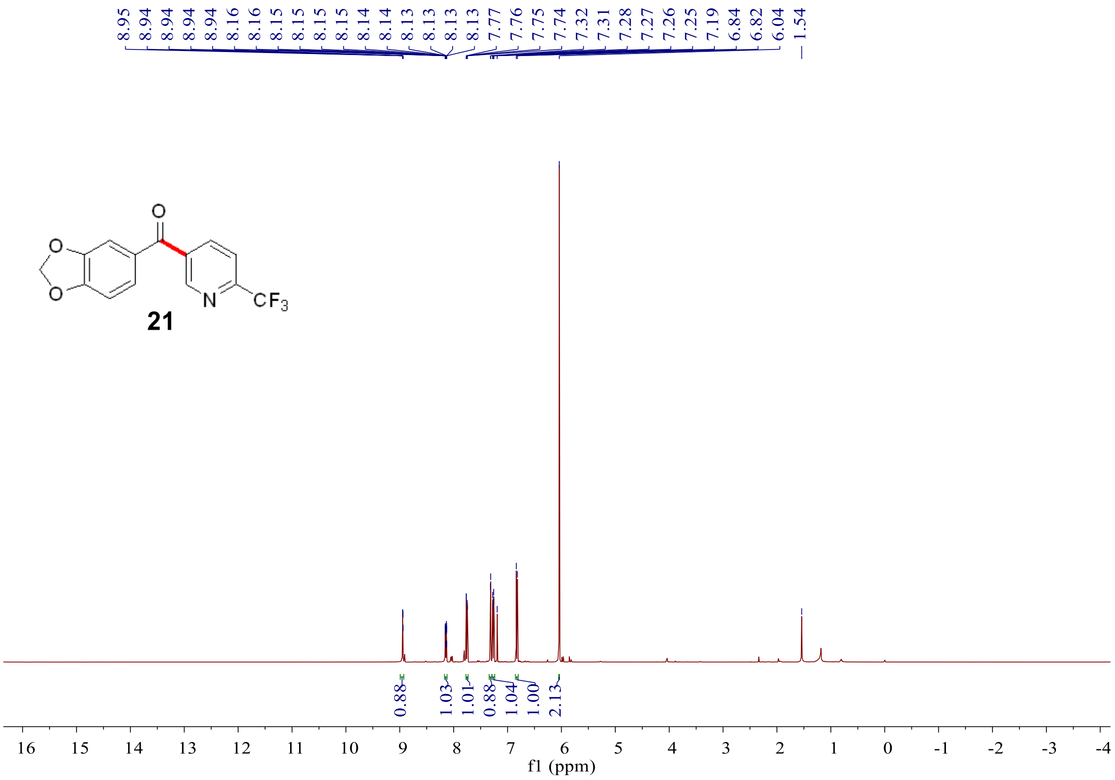

Supplementary Figure 67. ^{1}H NMR spectra for compound **21**

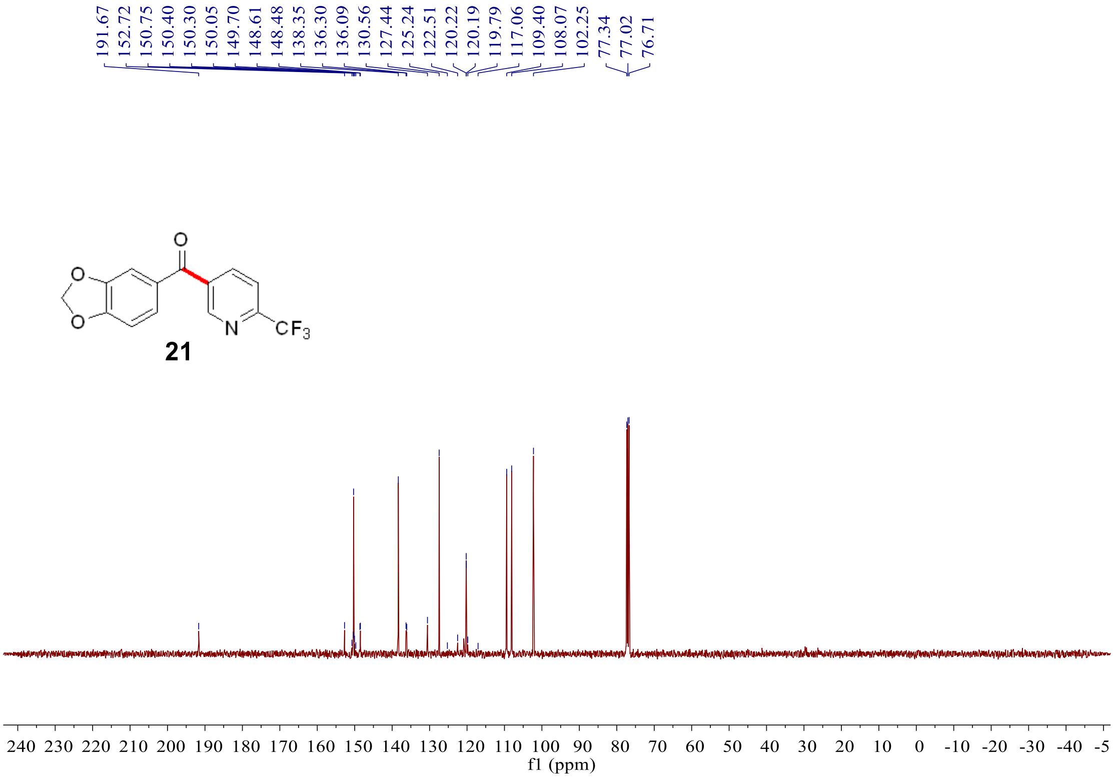

Supplementary Figure 68. ^{13}C NMR spectra for compound **21**

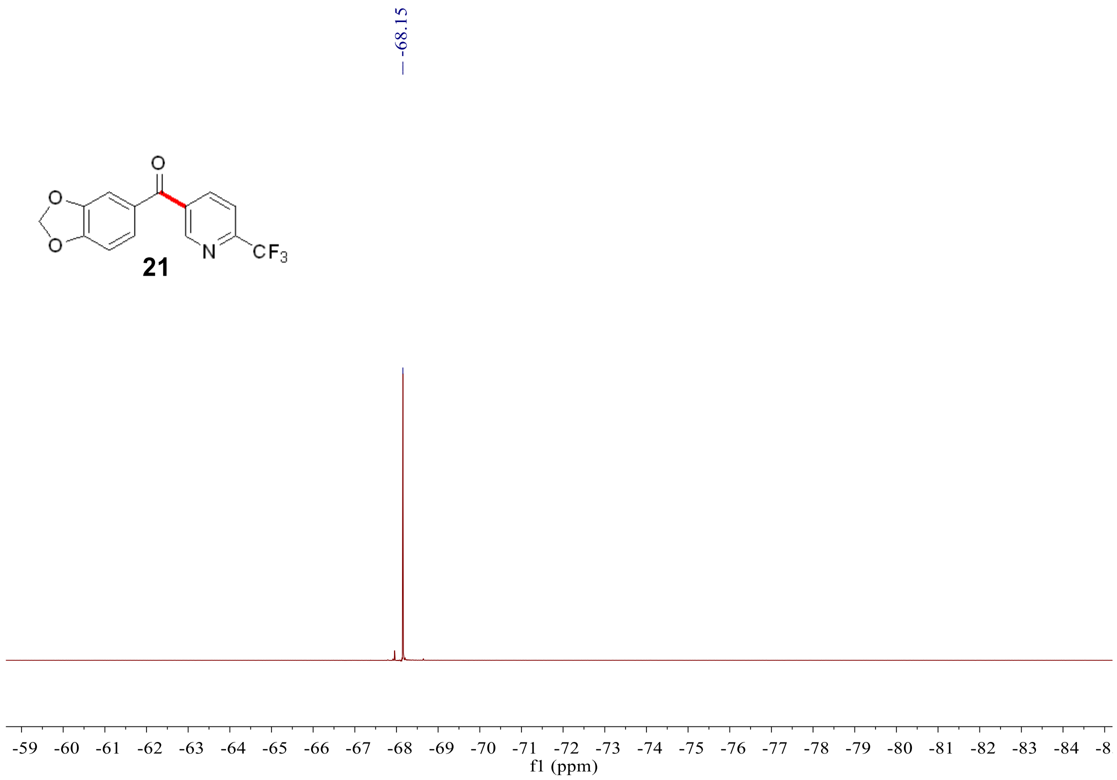

Supplementary Figure 69. ^{19}F NMR spectra for compound **21**

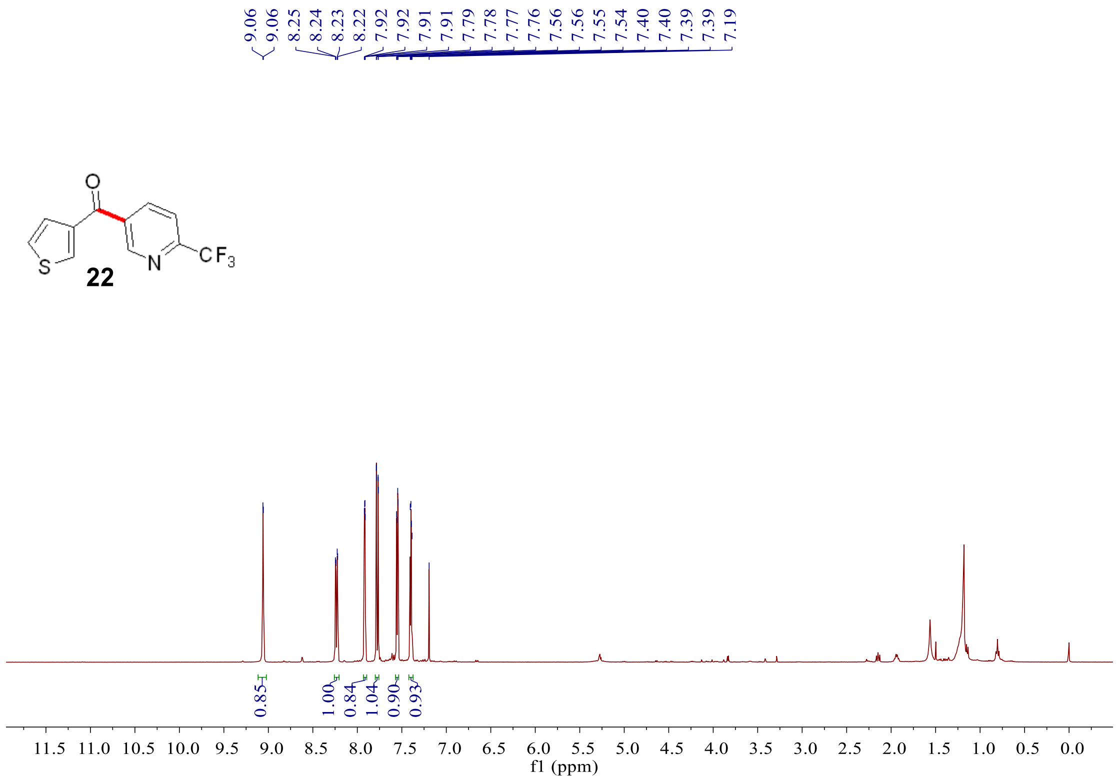

Supplementary Figure 70. ^{1}H NMR spectra for compound **22**

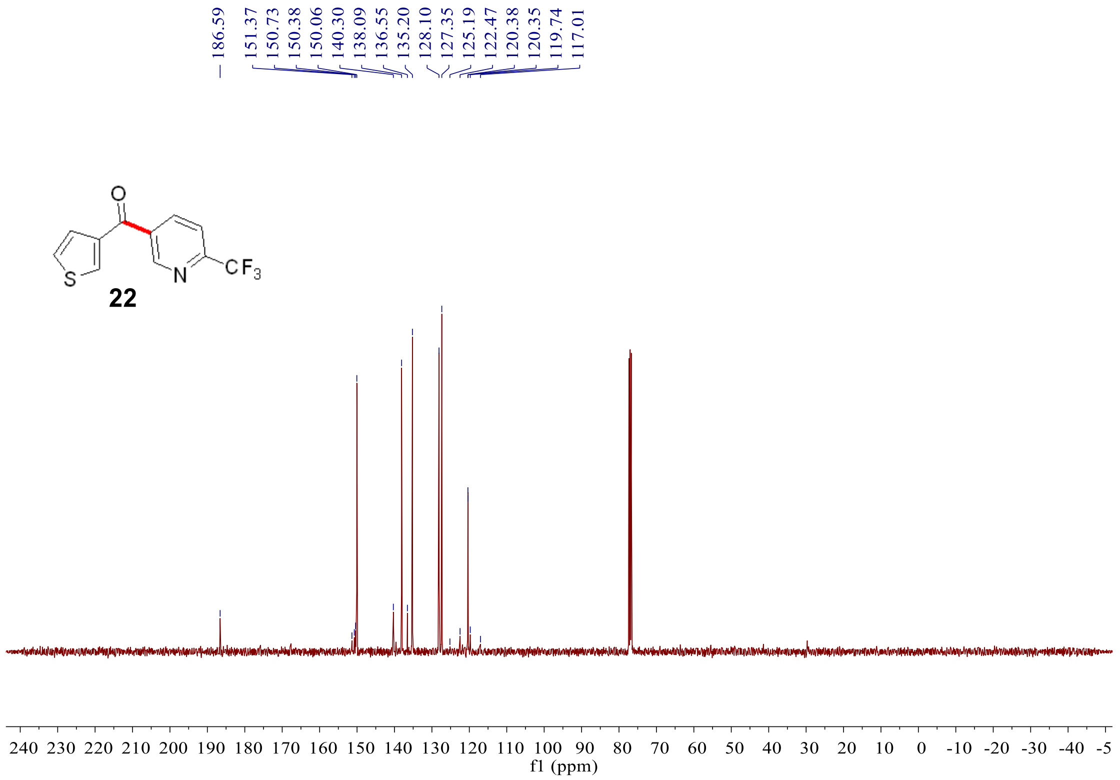

Supplementary Figure 71. ^{13}C NMR spectra for compound **22**

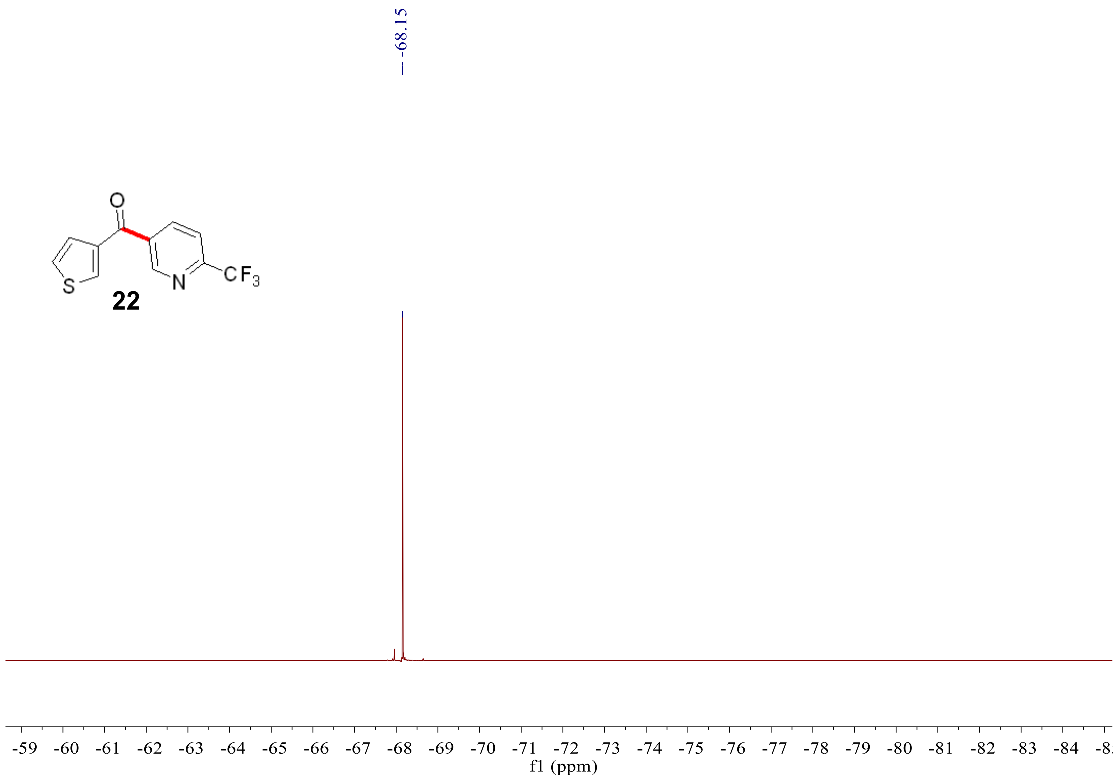

Supplementary Figure 72. ^{19}F NMR spectra for compound **22**

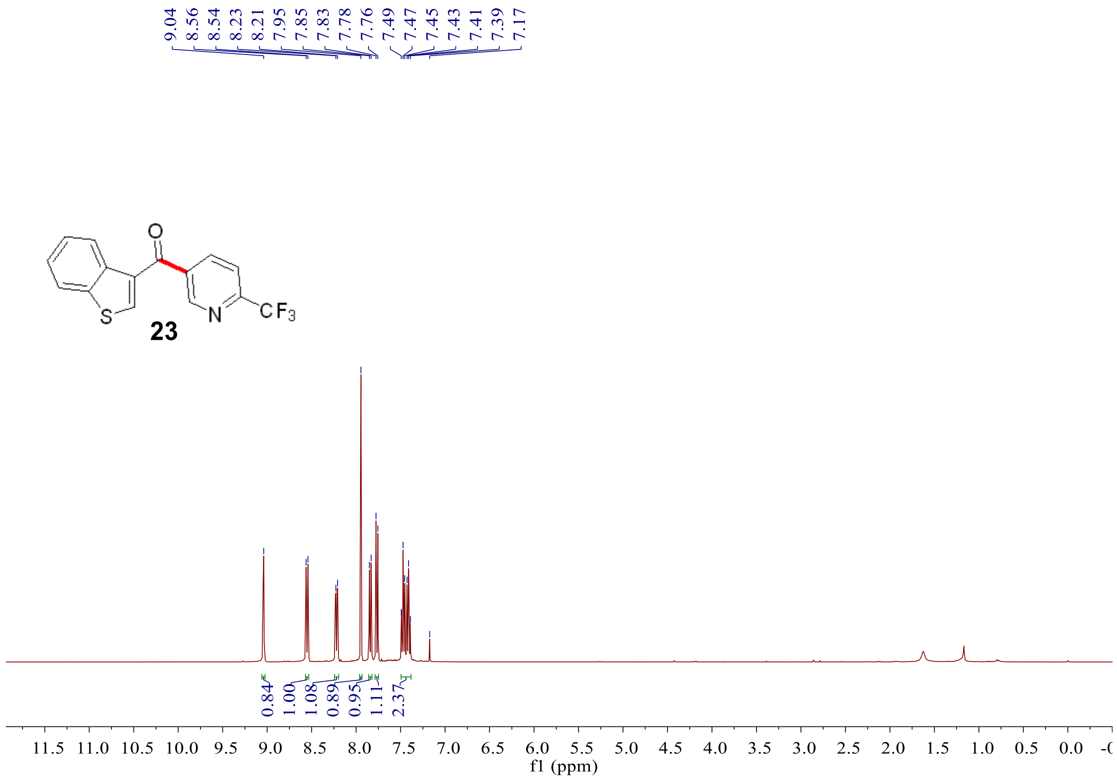

Supplementary Figure 73. ^{1}H NMR spectra for compound **23**

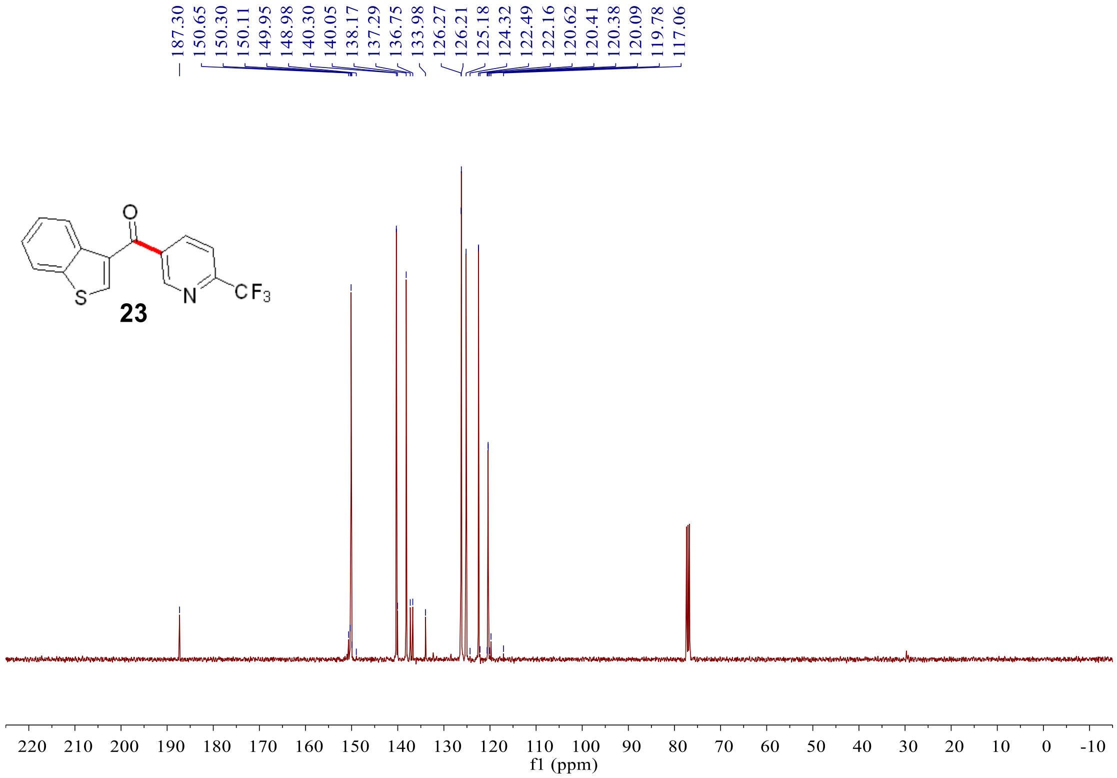

Supplementary Figure 74. ^{13}C NMR spectra for compound **23**

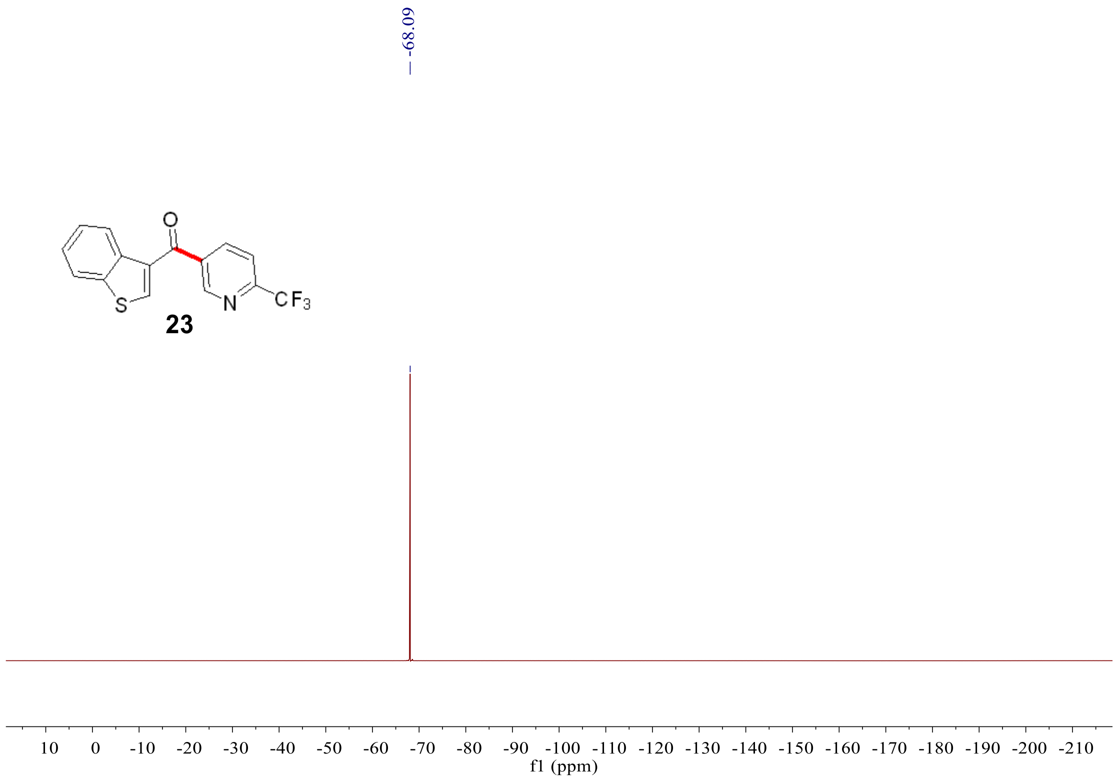

Supplementary Figure 75. ^{19}F NMR spectra for compound **23**

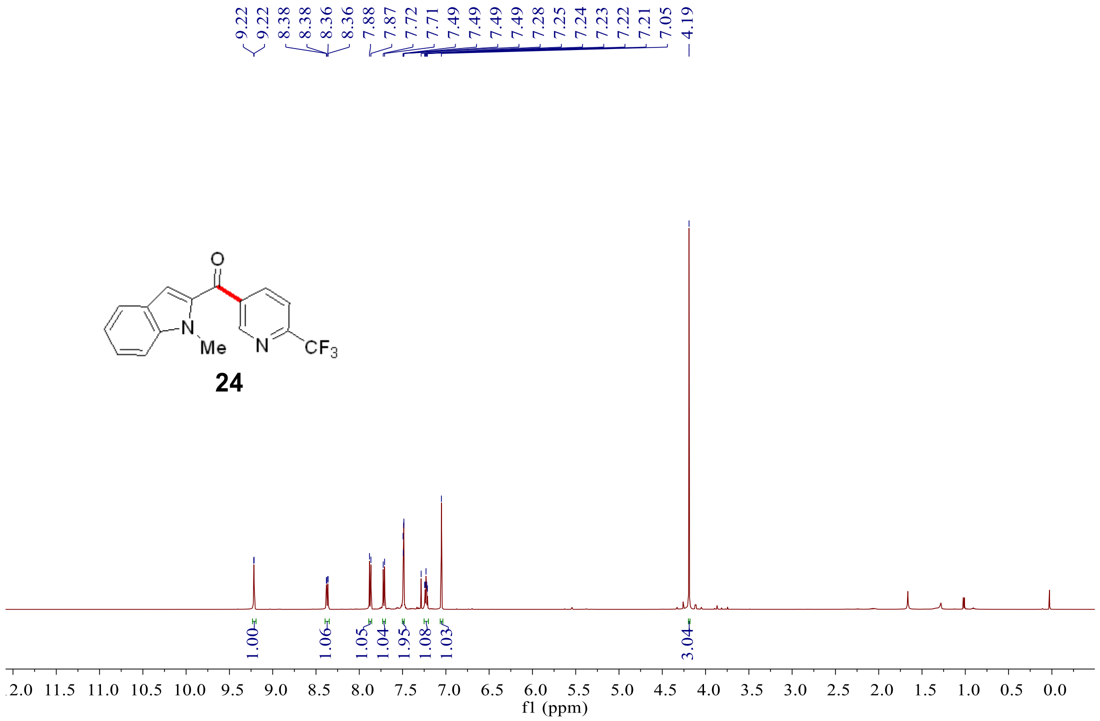

Supplementary Figure 76. ^{1}H NMR spectra for compound **24**

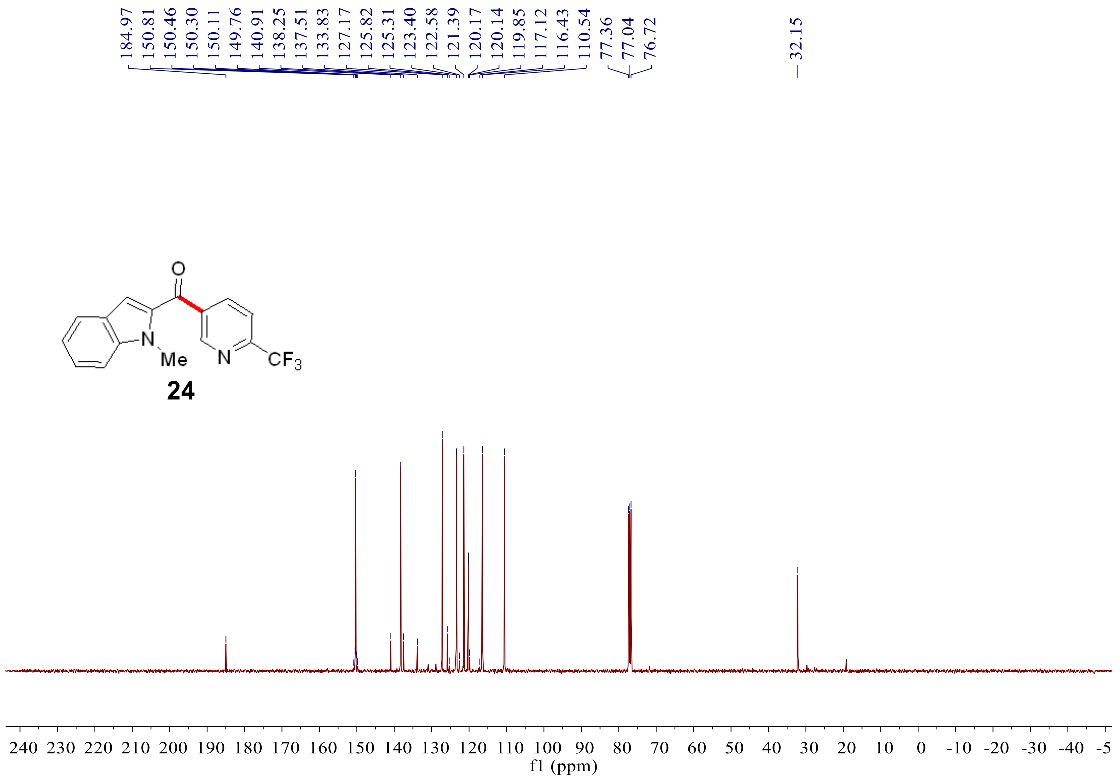

Supplementary Figure 77. ^{13}C NMR spectra for compound **24**

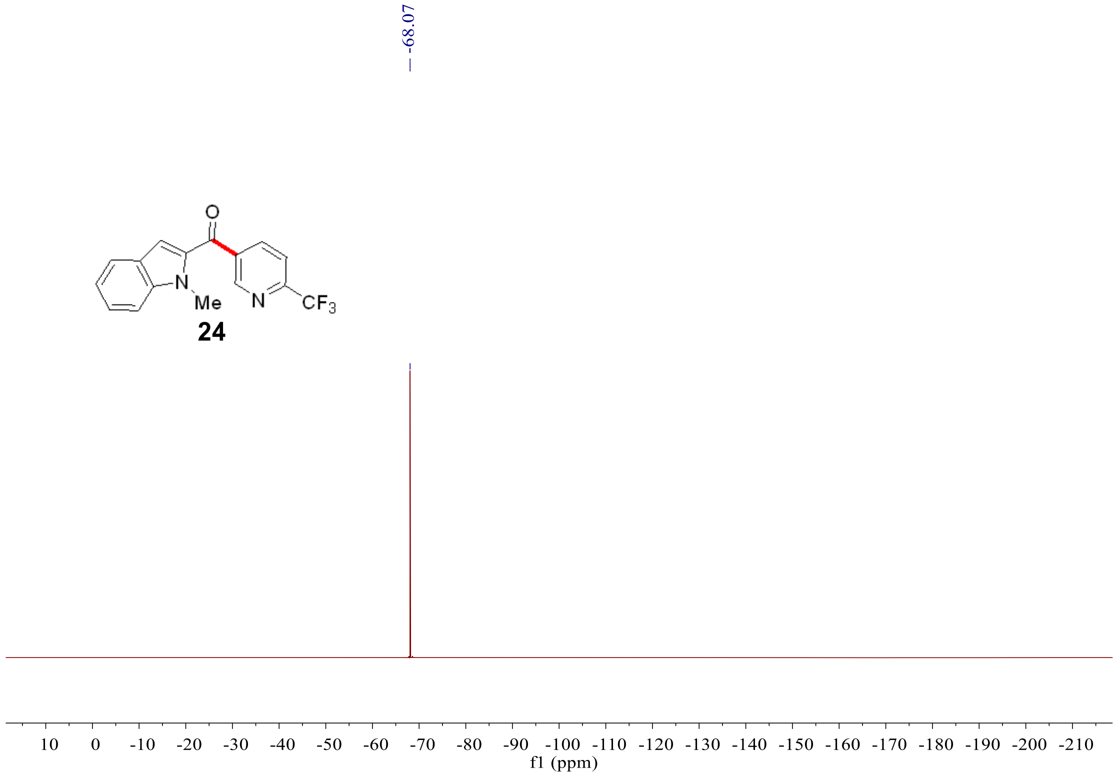

Supplementary Figure 78. ^{19}F NMR spectra for compound **24**

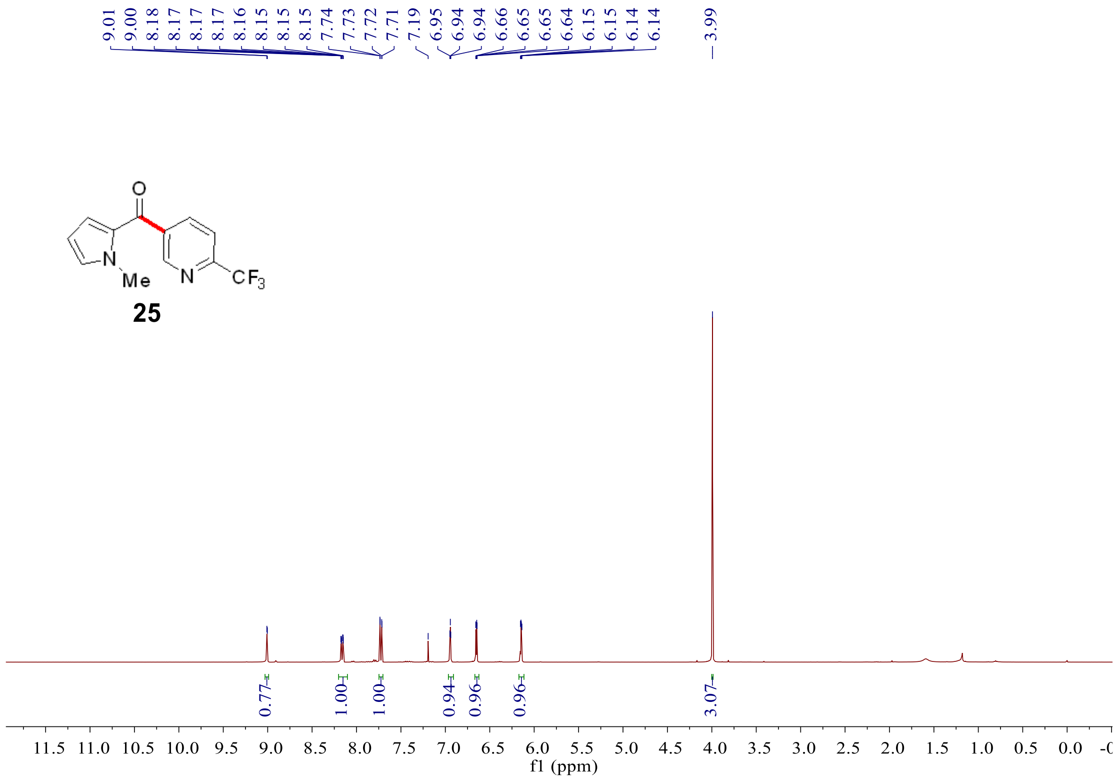

Supplementary Figure 79. ^{1}H NMR spectra for compound **25**

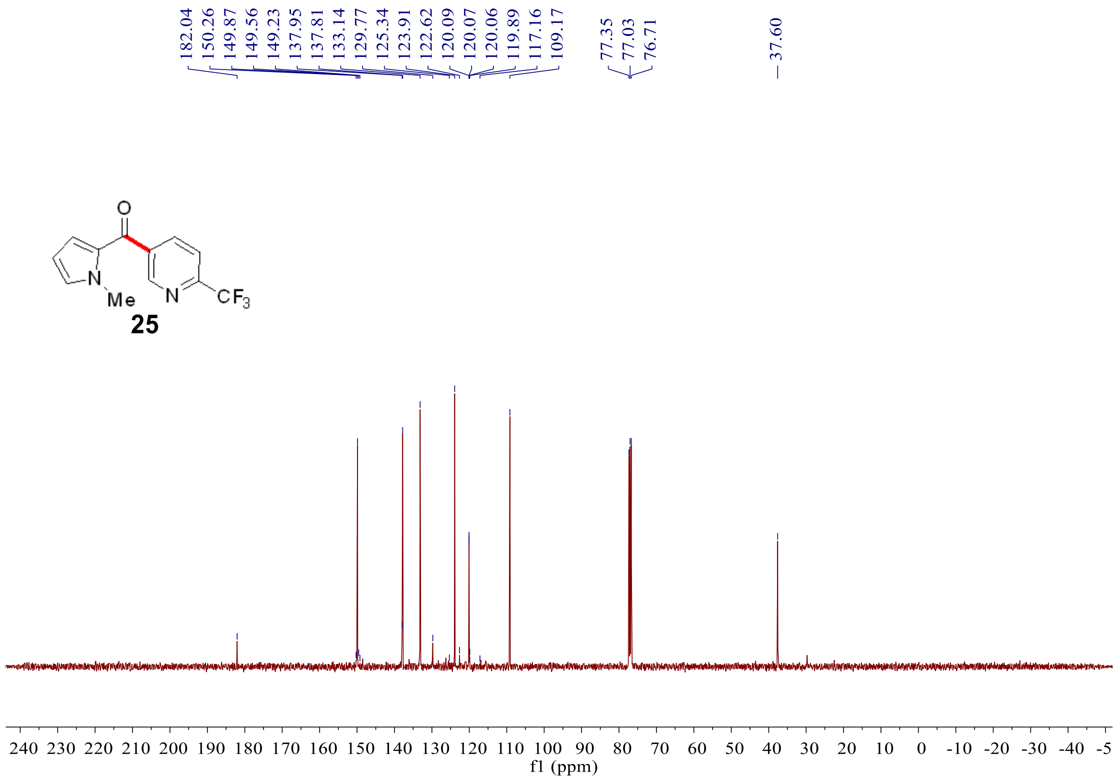

Supplementary Figure 80. ^{13}C NMR spectra for compound **25**

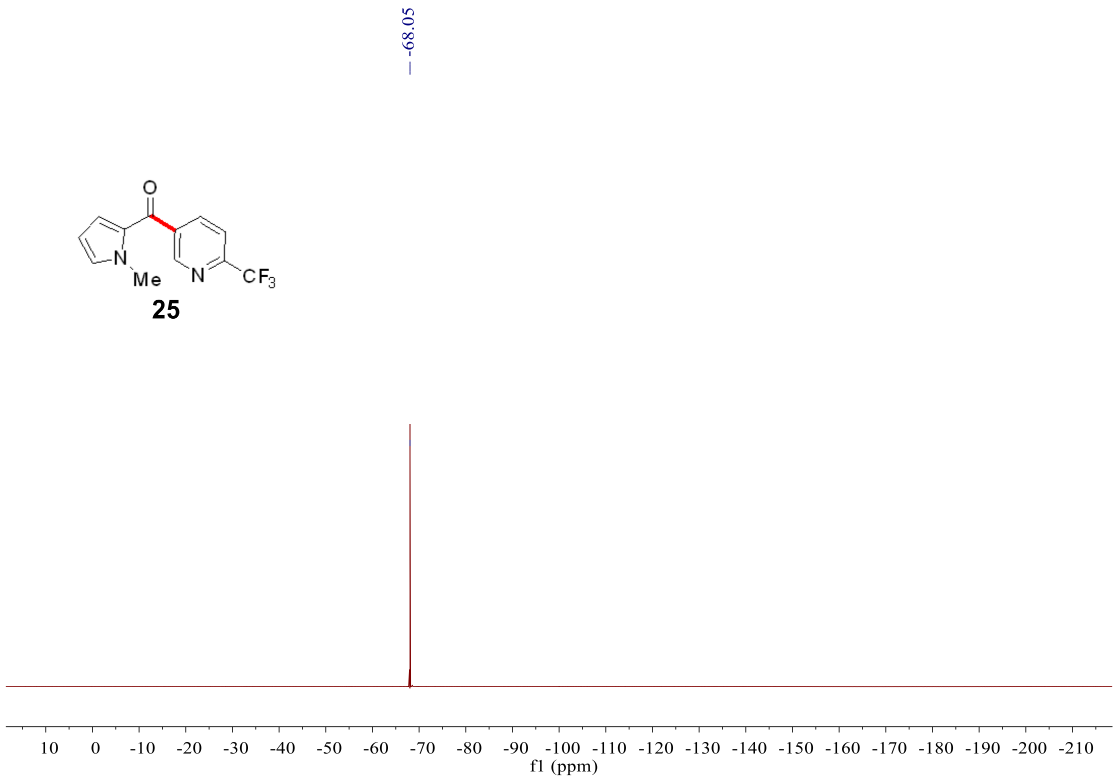

Supplementary Figure 81. ^{19}F NMR spectra for compound **25**

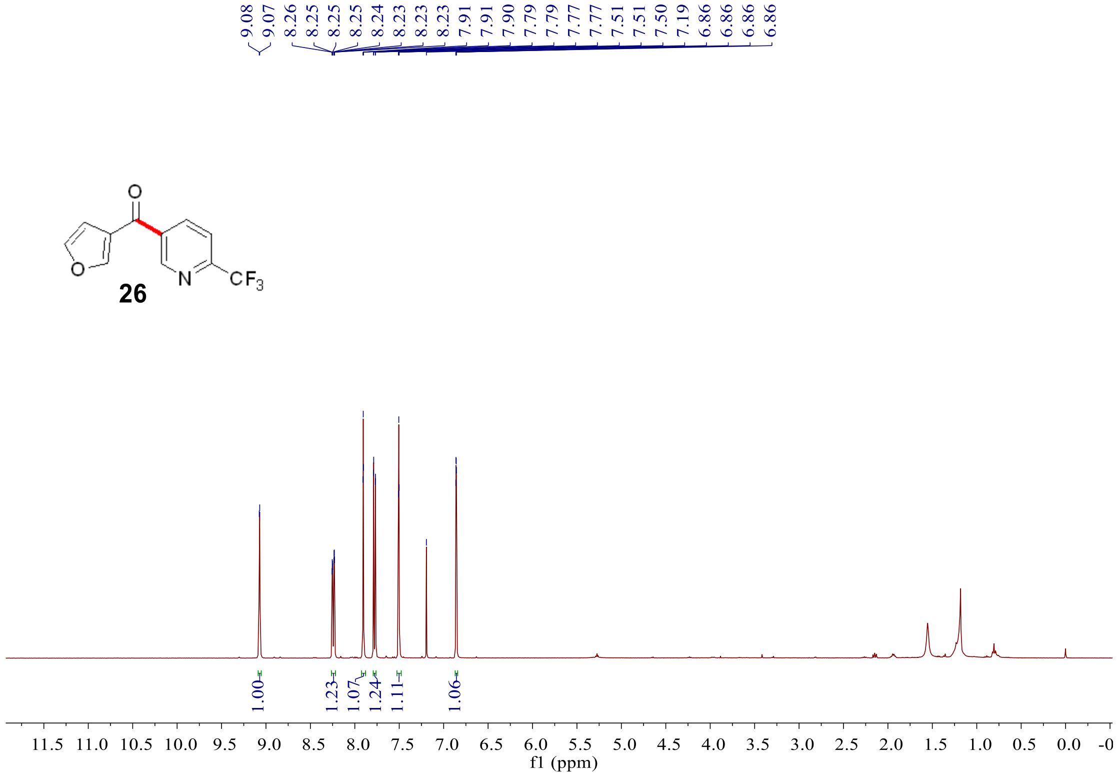

Supplementary Figure 82. ^{1}H NMR spectra for compound **26**

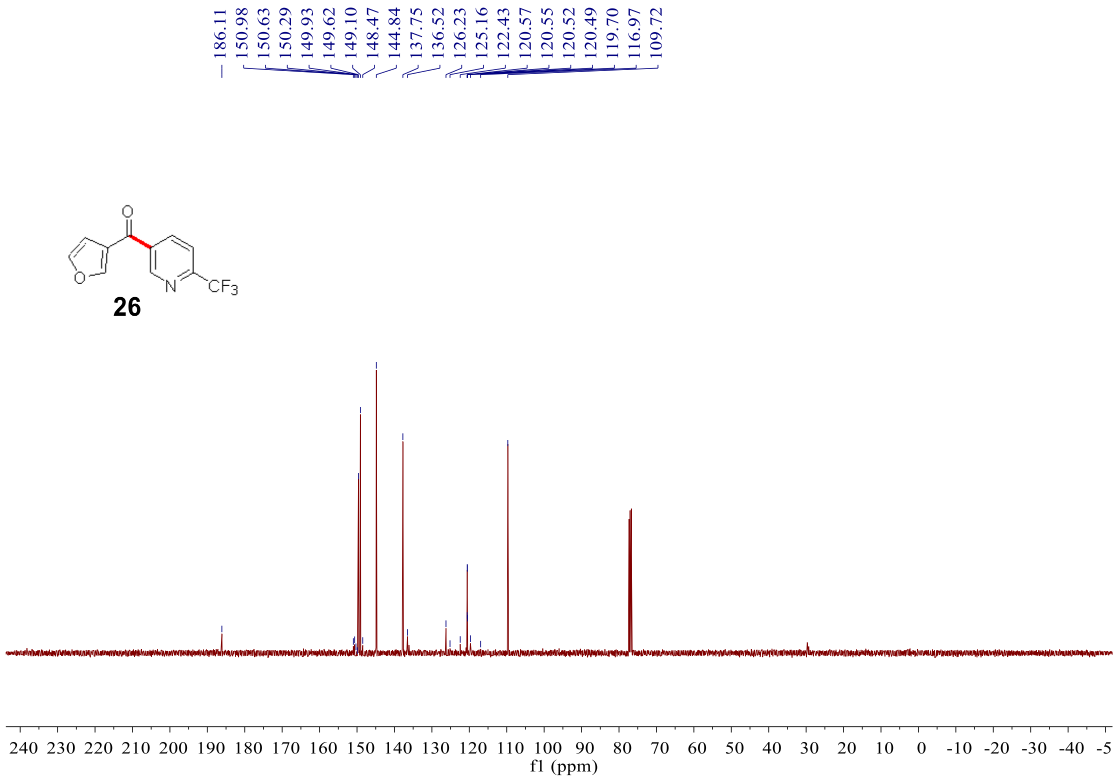

Supplementary Figure 83. ^{13}C NMR spectra for compound **26**

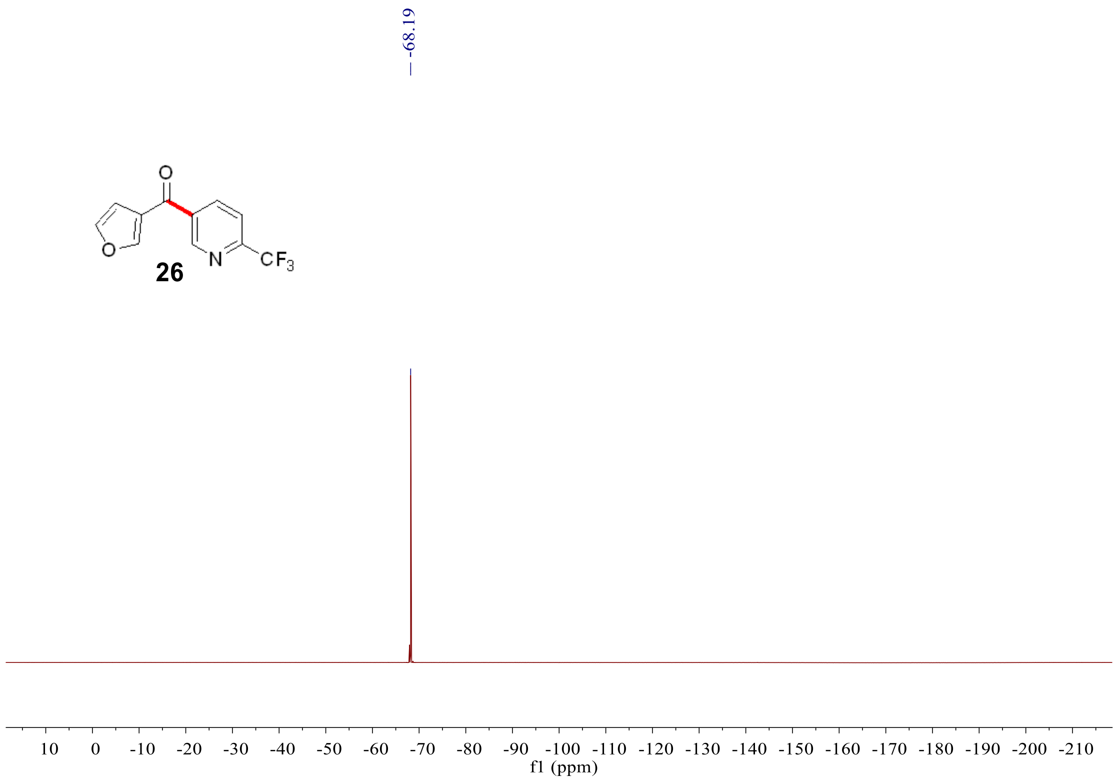

Supplementary Figure 84. ^{19}F NMR spectra for compound **26**

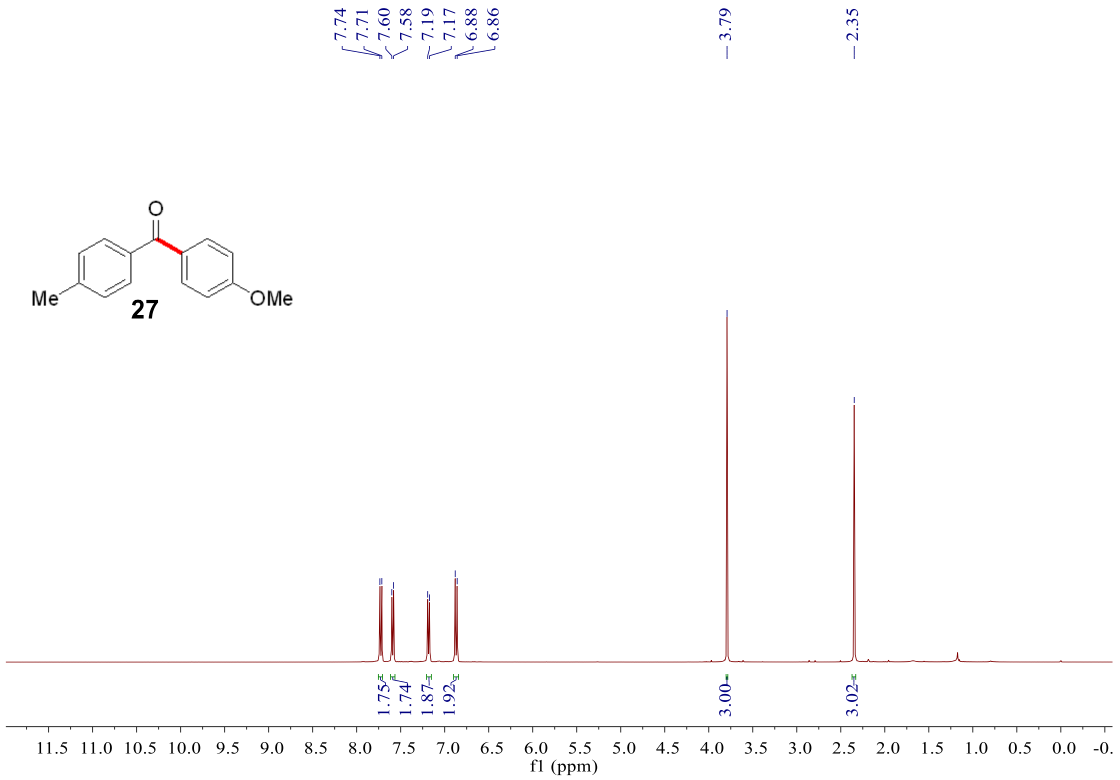

Supplementary Figure 85. ^{1}H NMR spectra for compound **27**

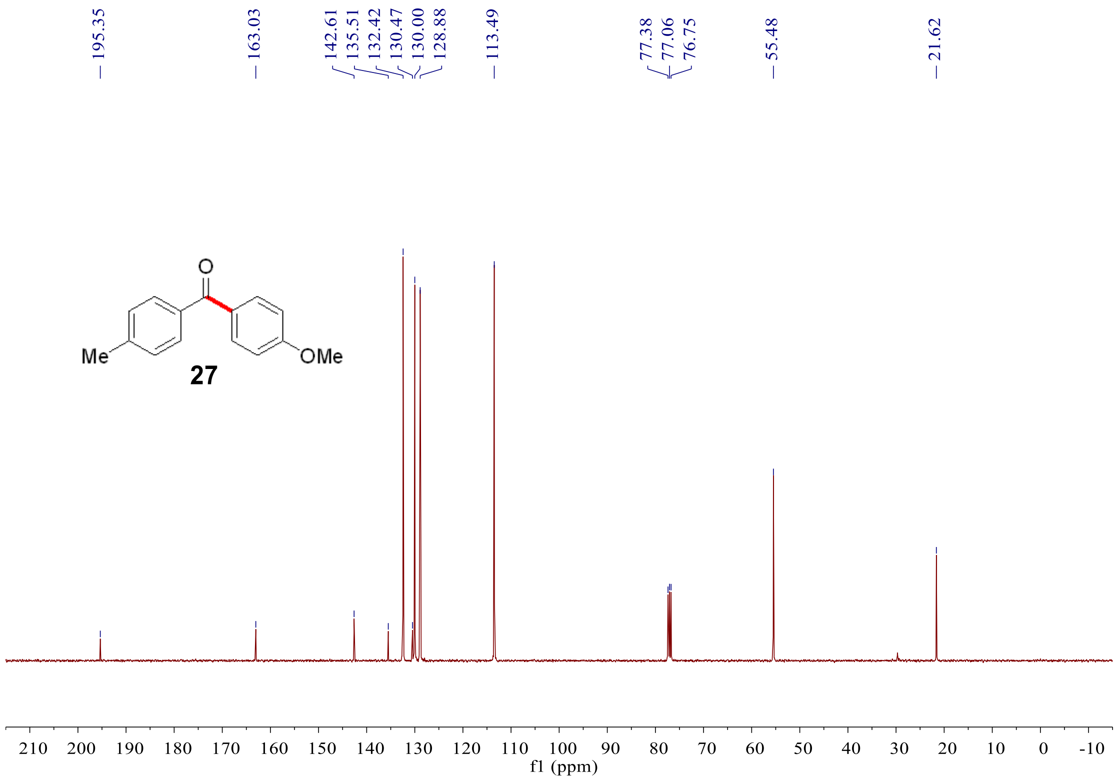

Supplementary Figure 86. ^{13}C NMR spectra for compound **27**

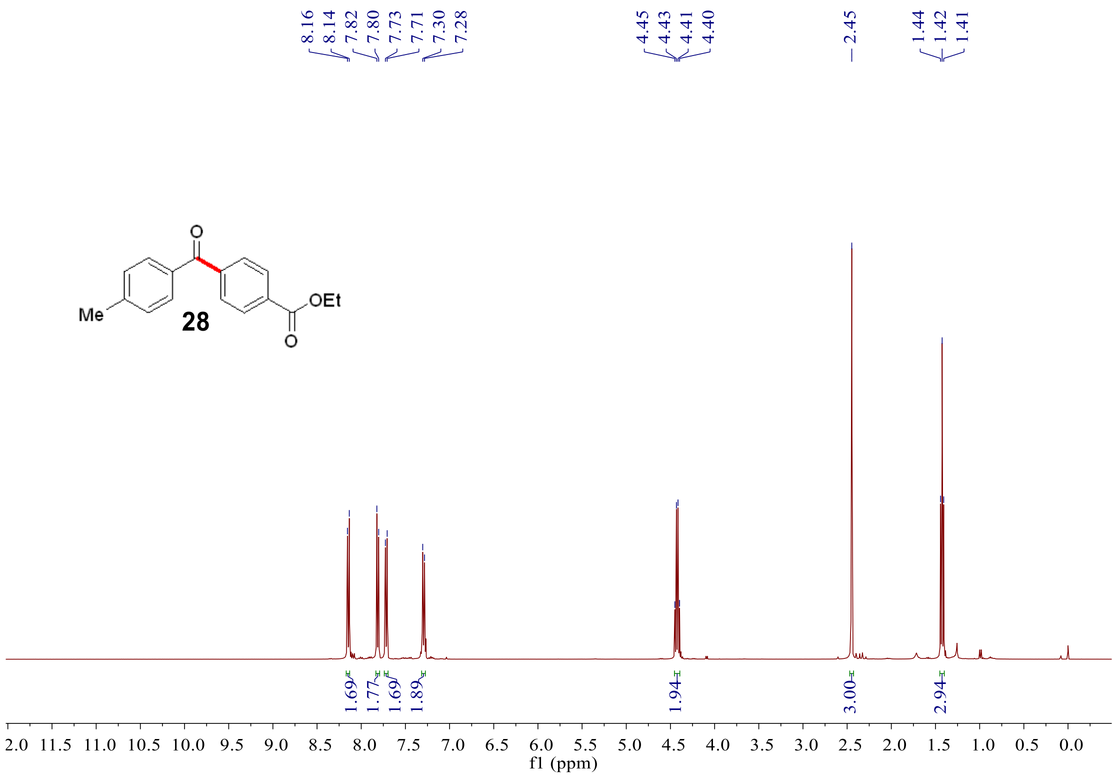

Supplementary Figure 87. ^{1}H NMR spectra for compound **28**

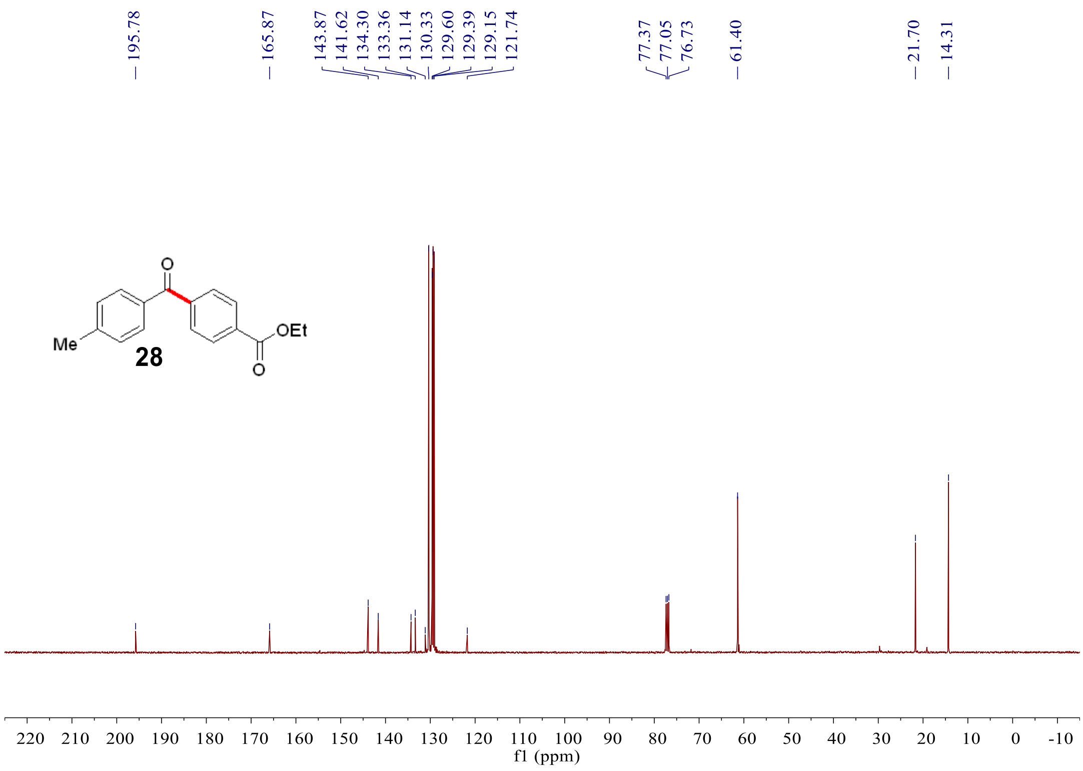

Supplementary Figure 88. ^{13}C NMR spectra for compound **28**

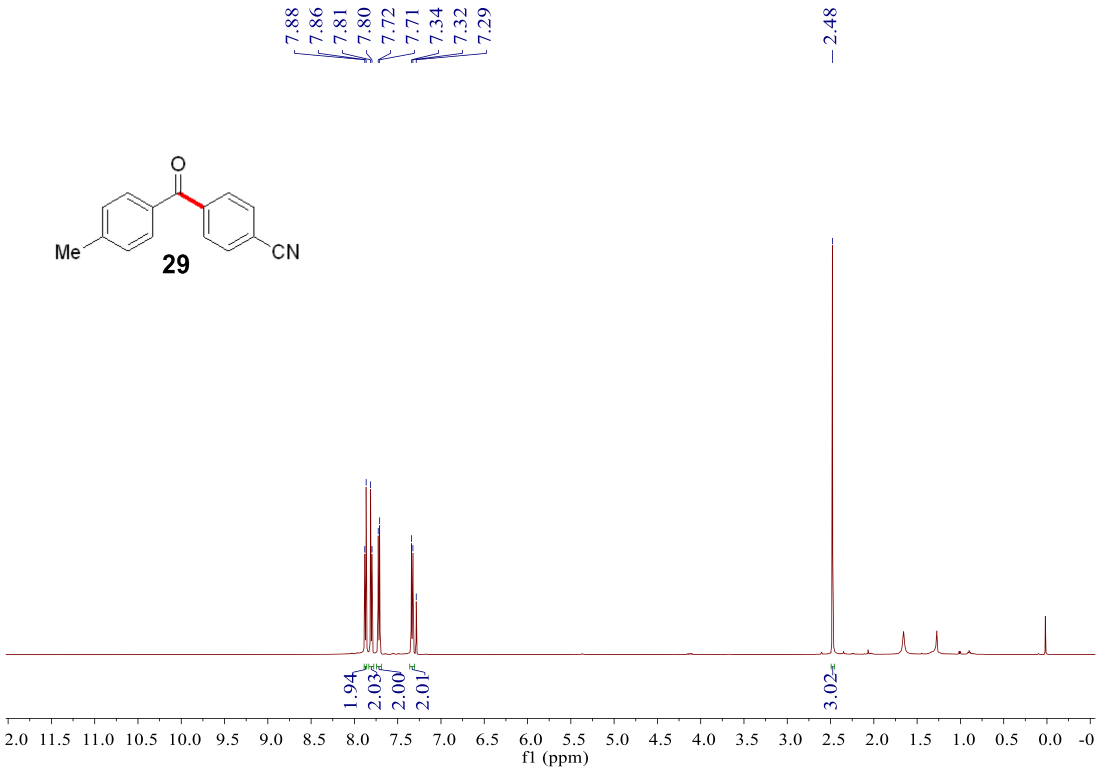

Supplementary Figure 89. ^{1}H NMR spectra for compound **29**

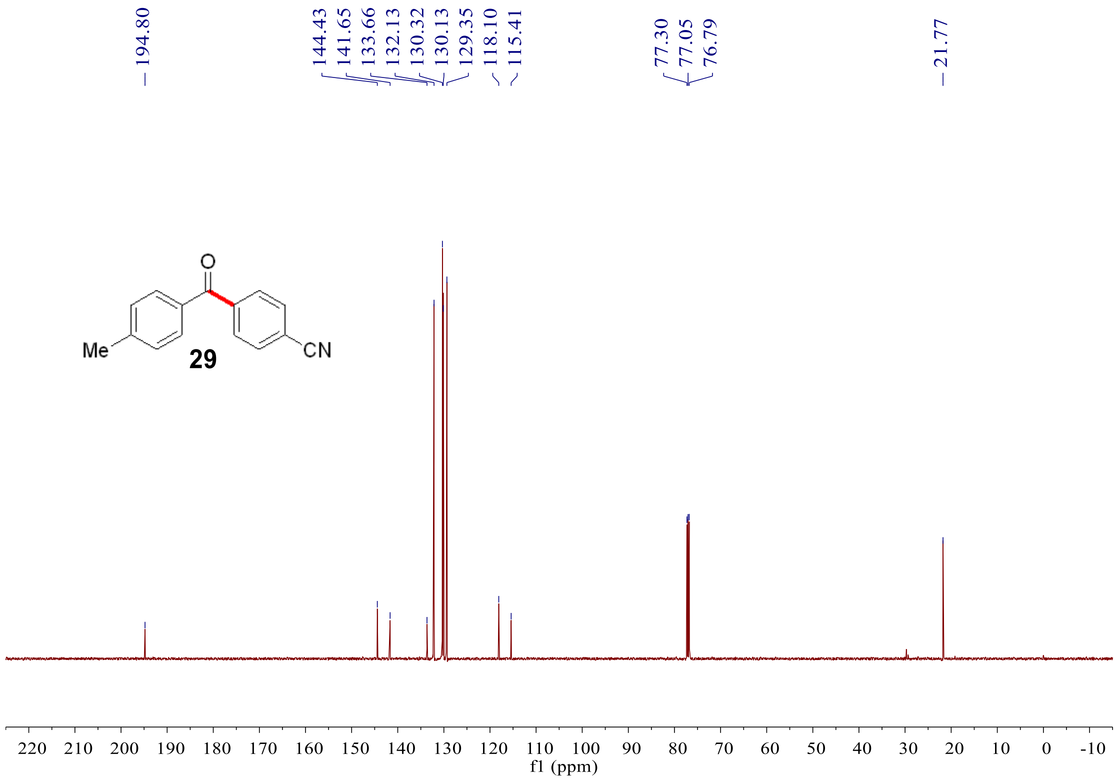

Supplementary Figure 90. ^{13}C NMR spectra for compound **29**

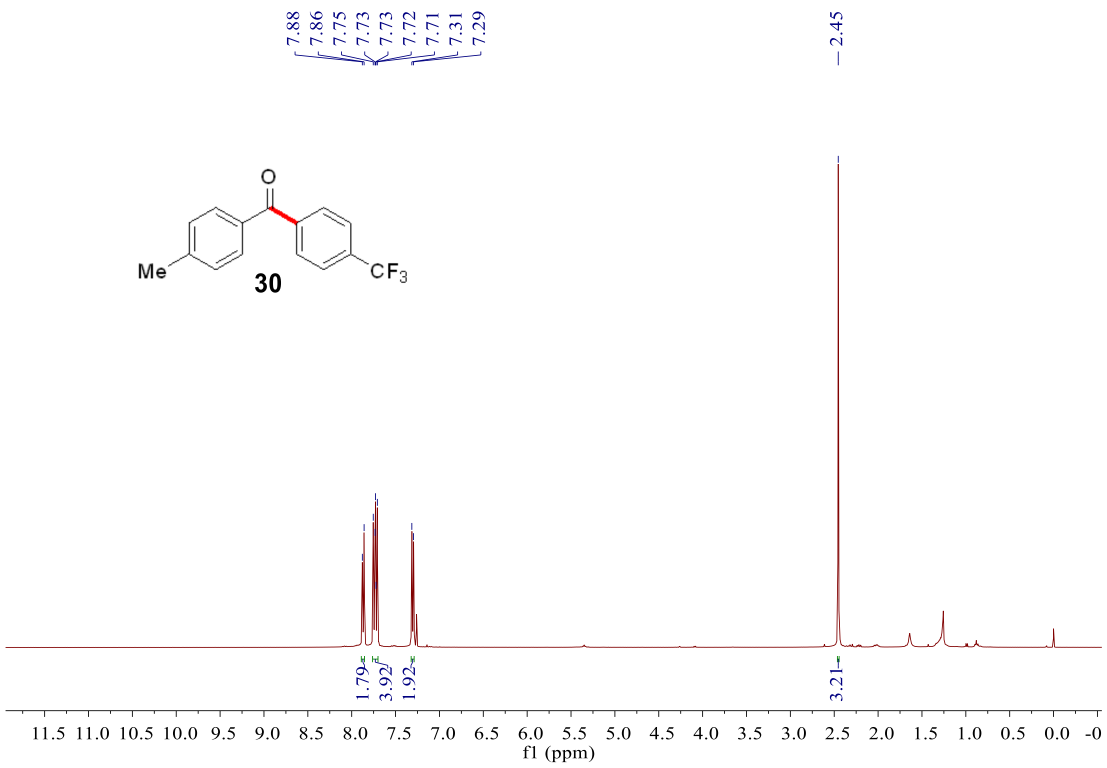

Supplementary Figure 91. ^{1}H NMR spectra for compound **30**

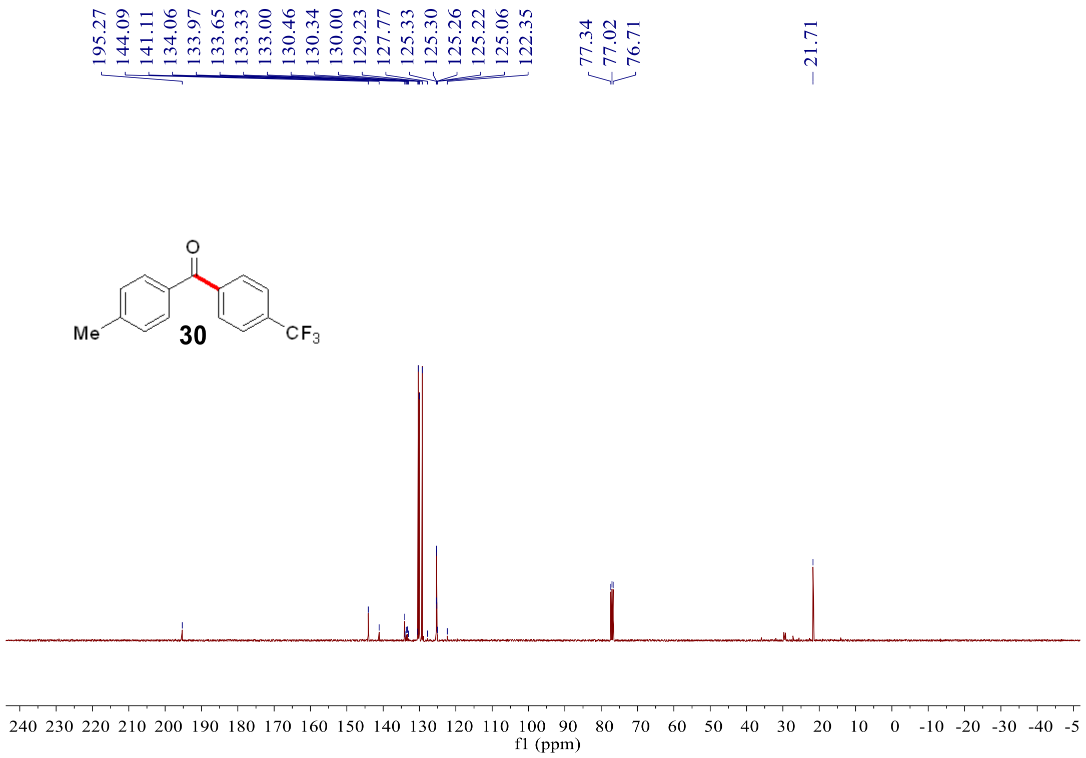

Supplementary Figure 92. ^{13}C NMR spectra for compound **30**

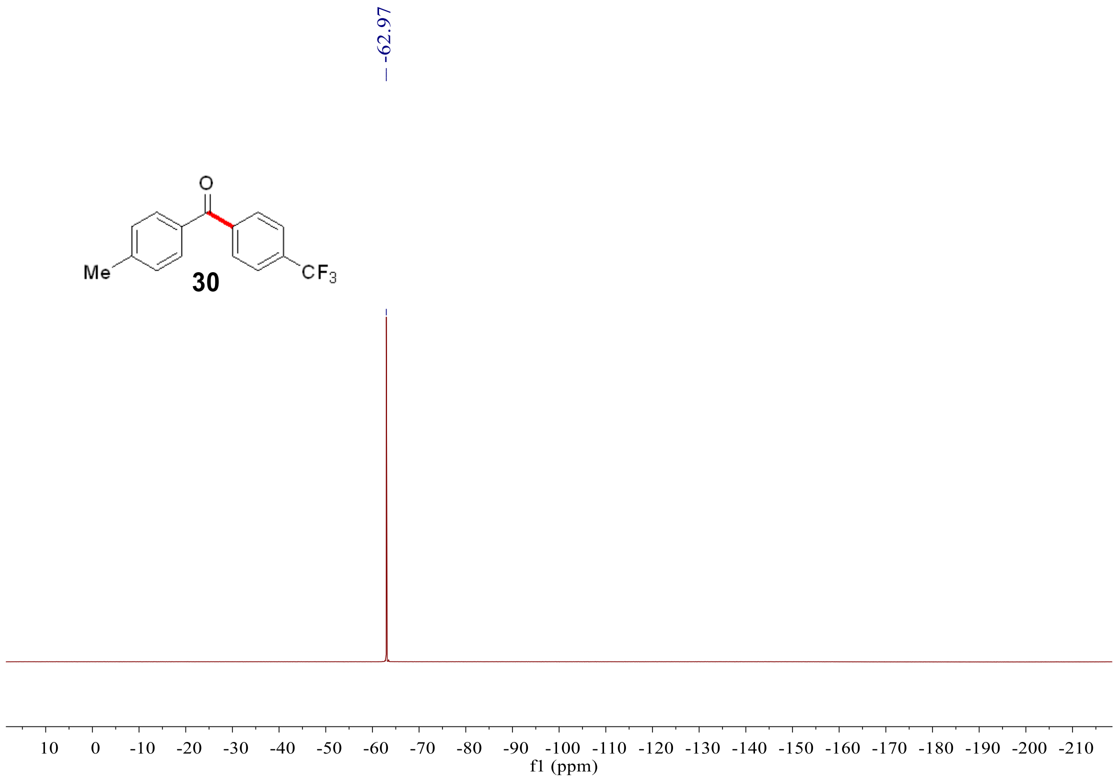

Supplementary Figure 93. ^{19}F NMR spectra for compound **30**

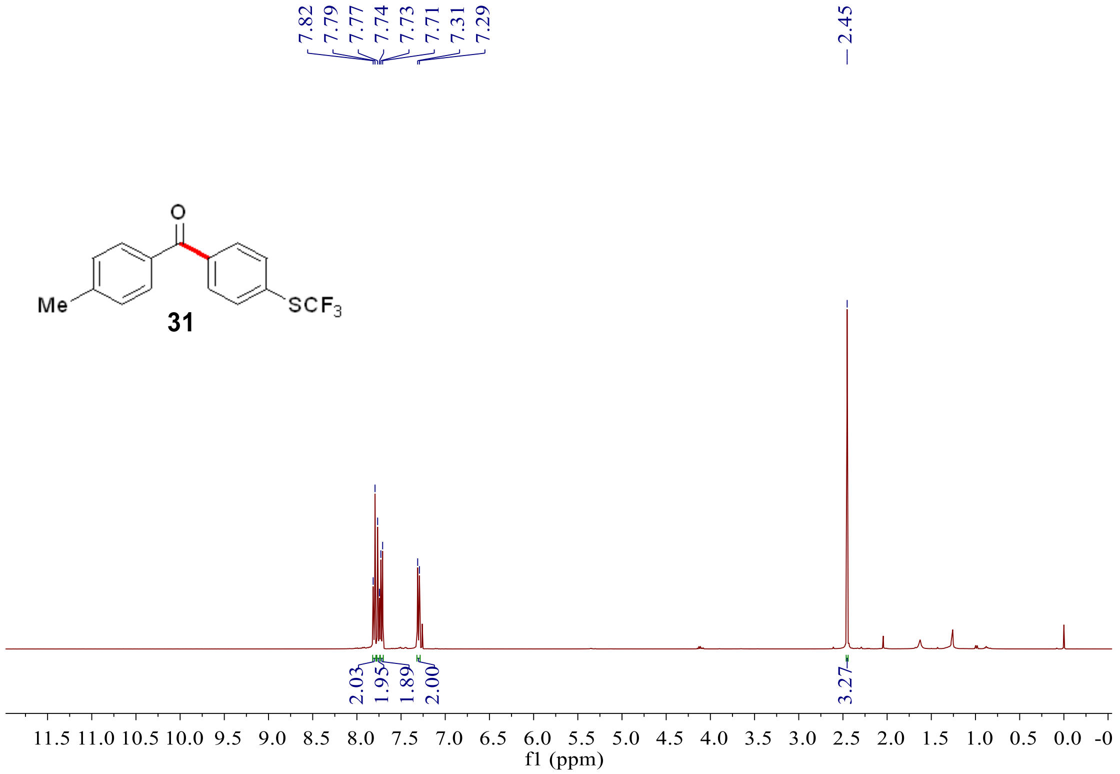

Supplementary Figure 94. ^{1}H NMR spectra for compound **31**

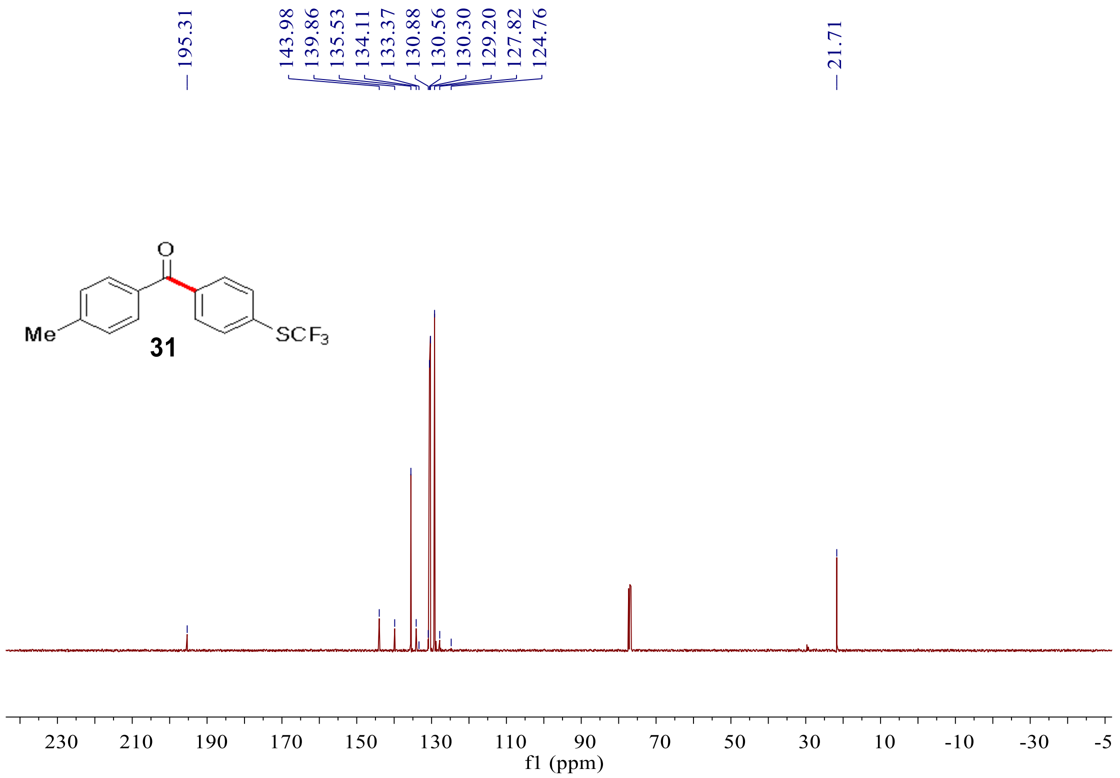

Supplementary Figure 95. ^{13}C NMR spectra for compound **31**

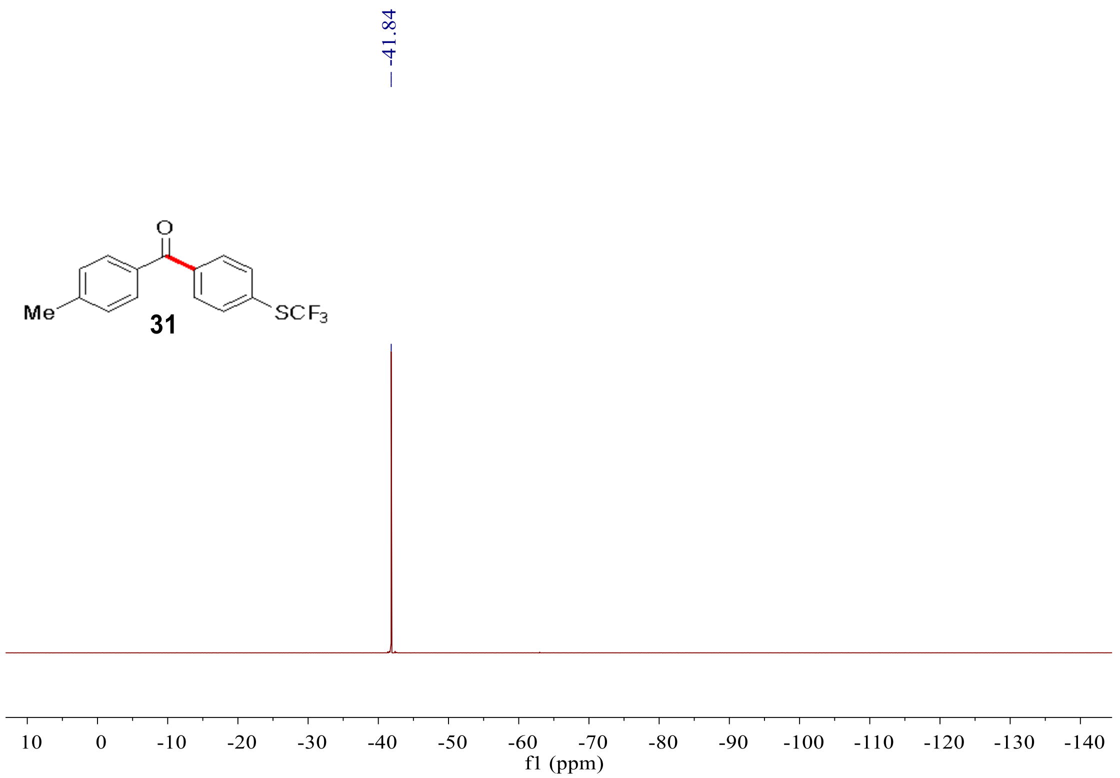

Supplementary Figure 96. ^{19}F NMR spectra for compound **31**

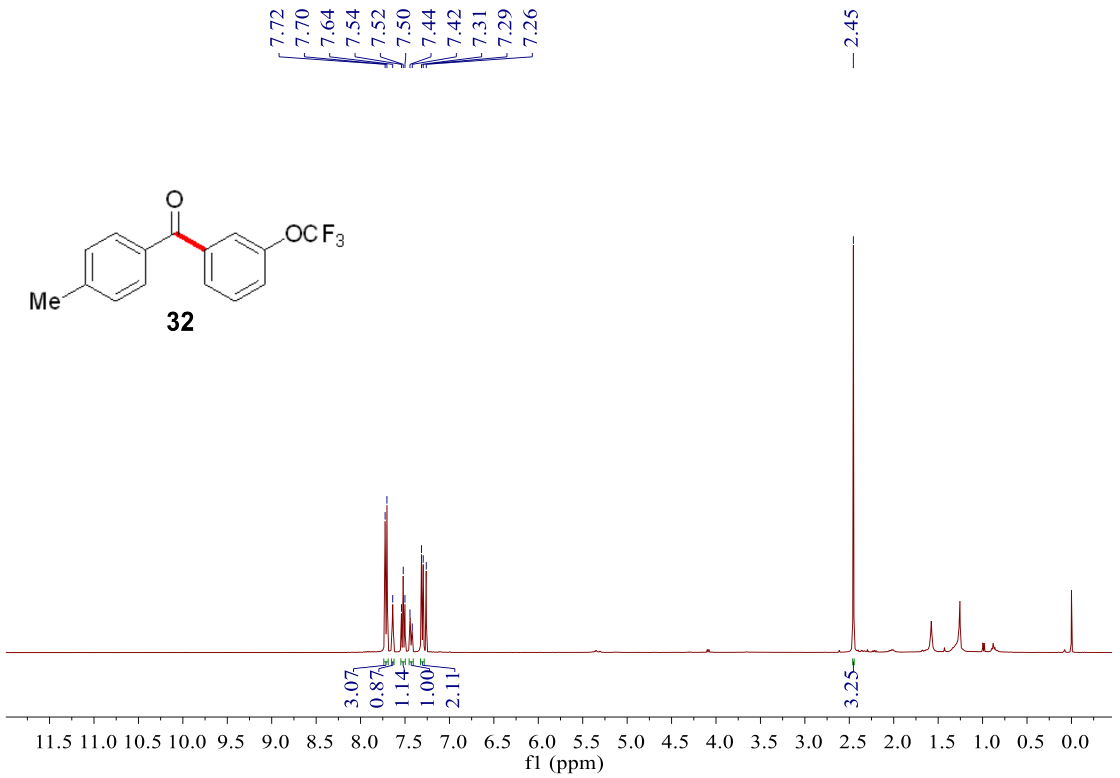

Supplementary Figure 97. ^{1}H NMR spectra for compound **32**

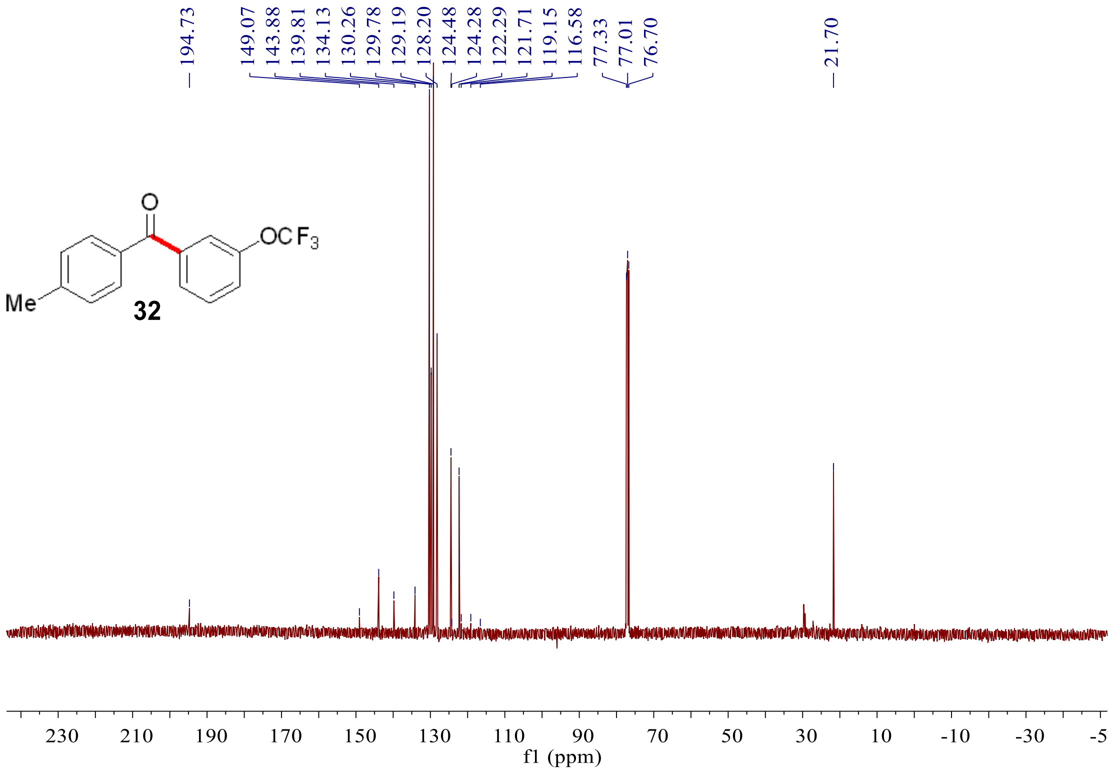

Supplementary Figure 98. ^{13}C NMR spectra for compound **32**

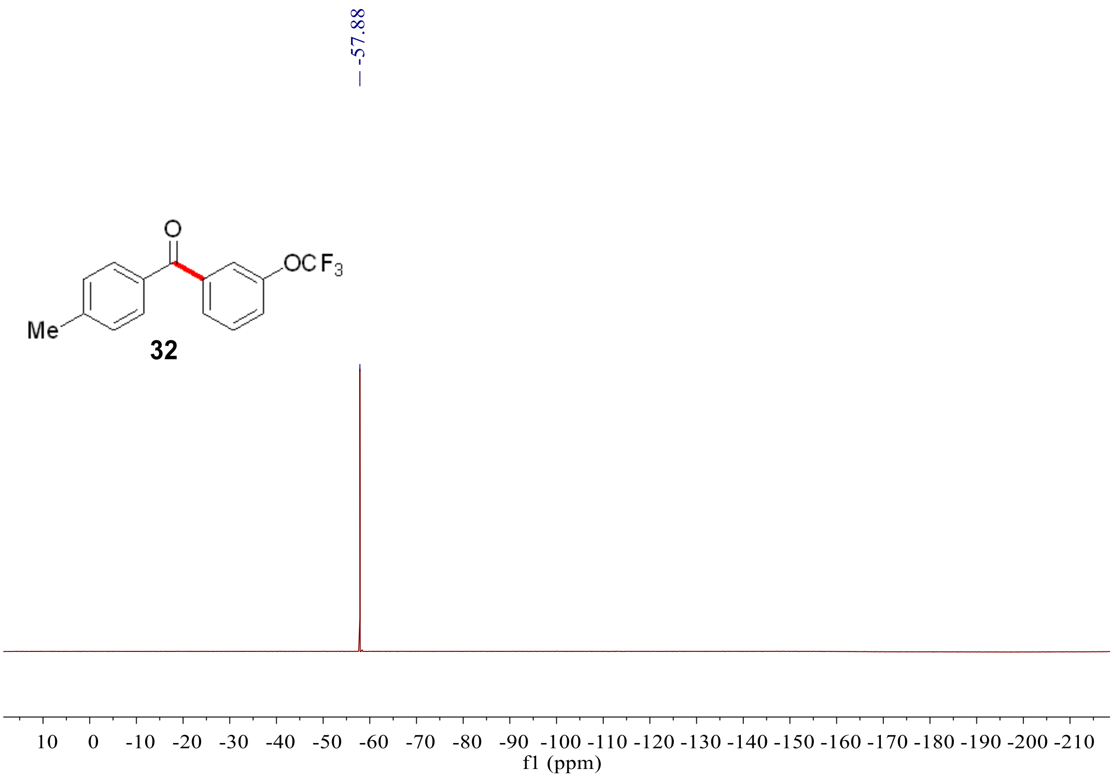

Supplementary Figure 99. ^{19}F NMR spectra for compound **32**

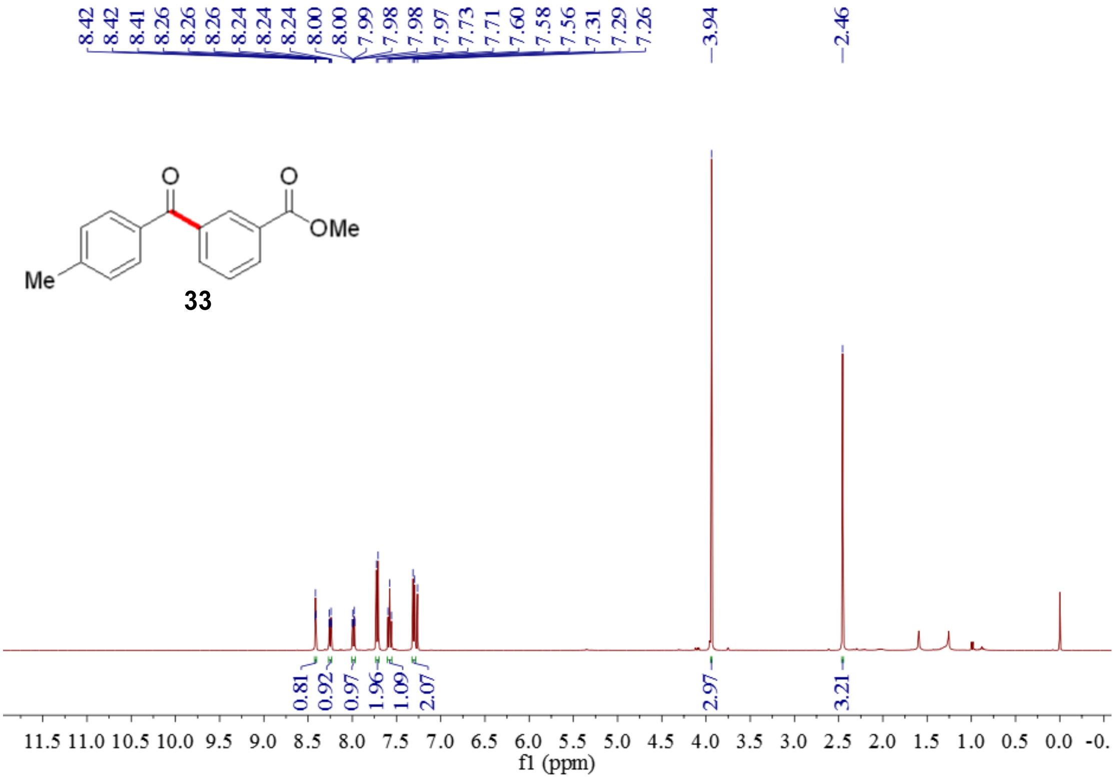

Supplementary Figure 100. ^{1}H NMR spectra for compound **33**

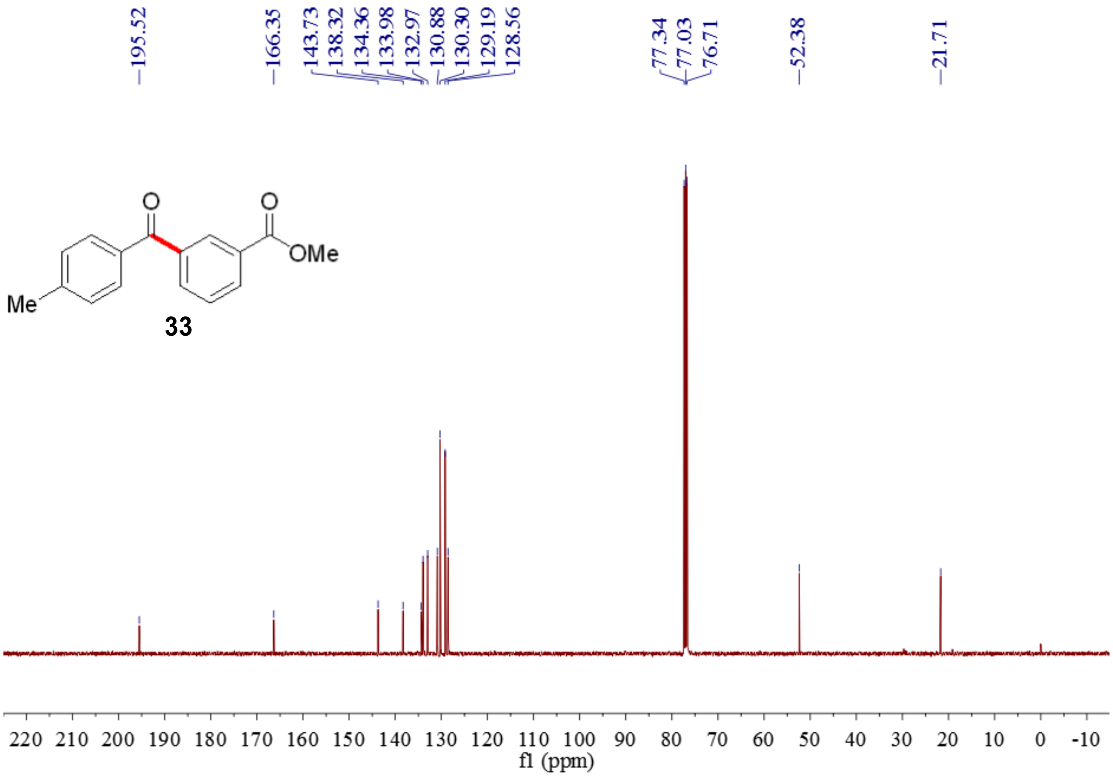

Supplementary Figure 101. ^{13}C NMR spectra for compound **33**

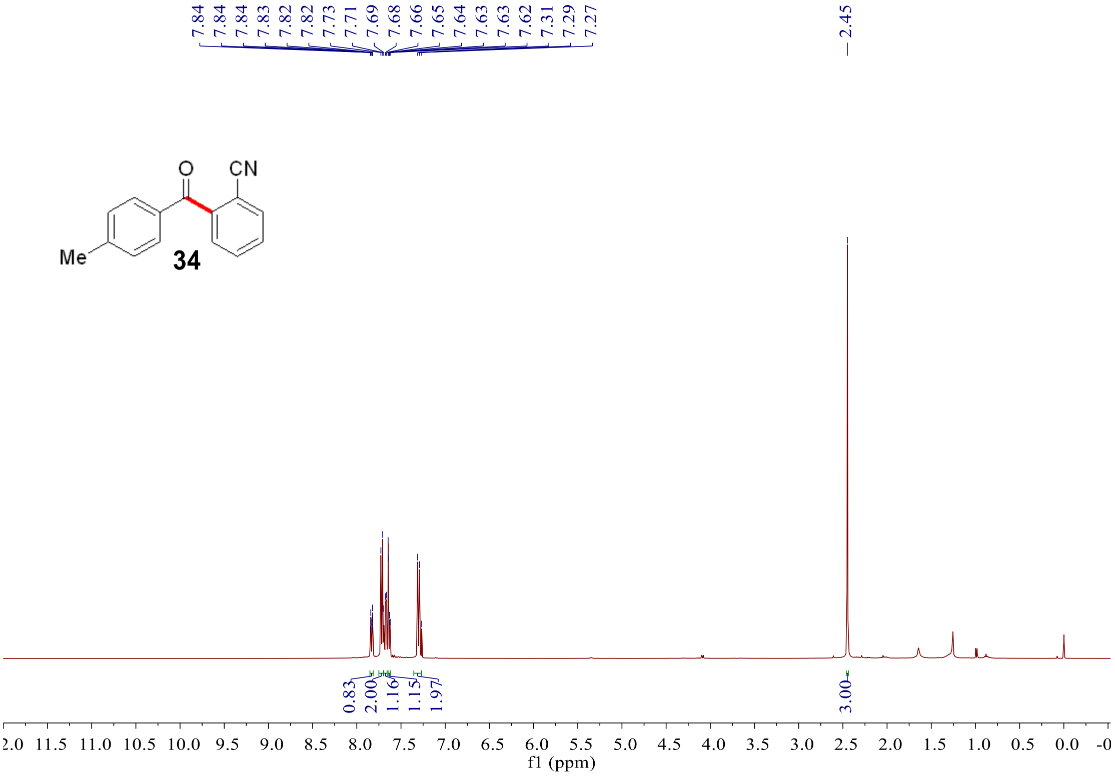

Supplementary Figure 102. ^{1}H NMR spectra for compound **34**

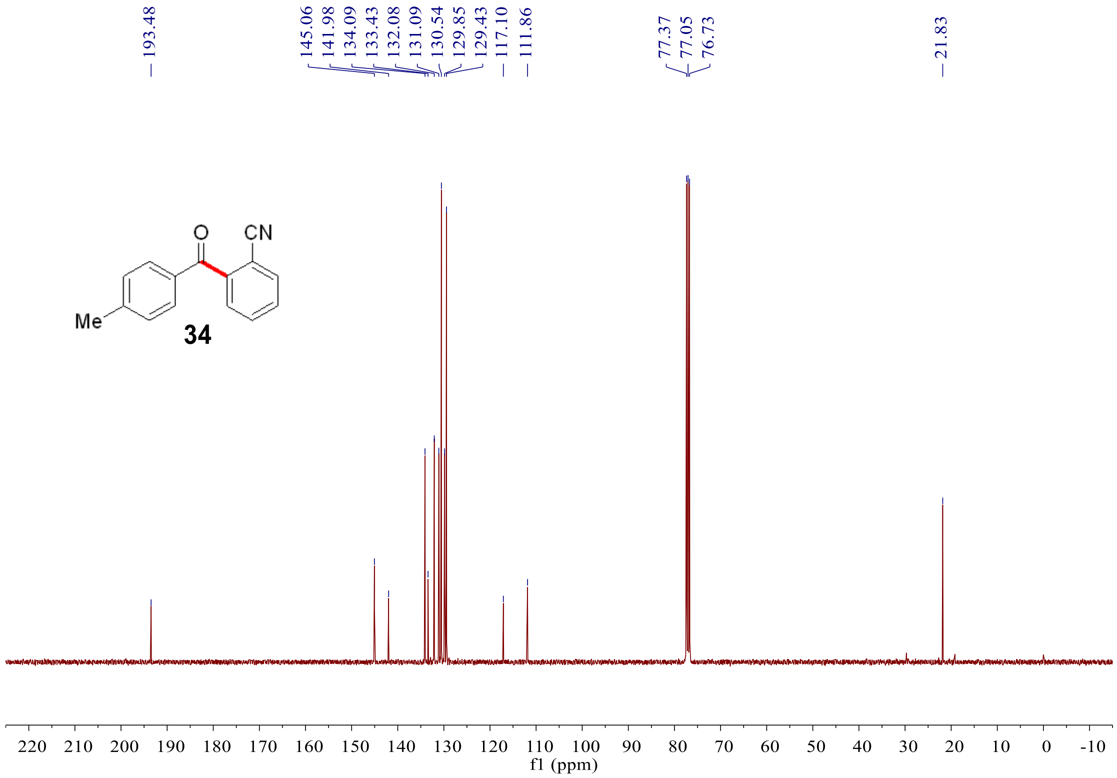

Supplementary Figure 103. ^{13}C NMR spectra for compound **34**

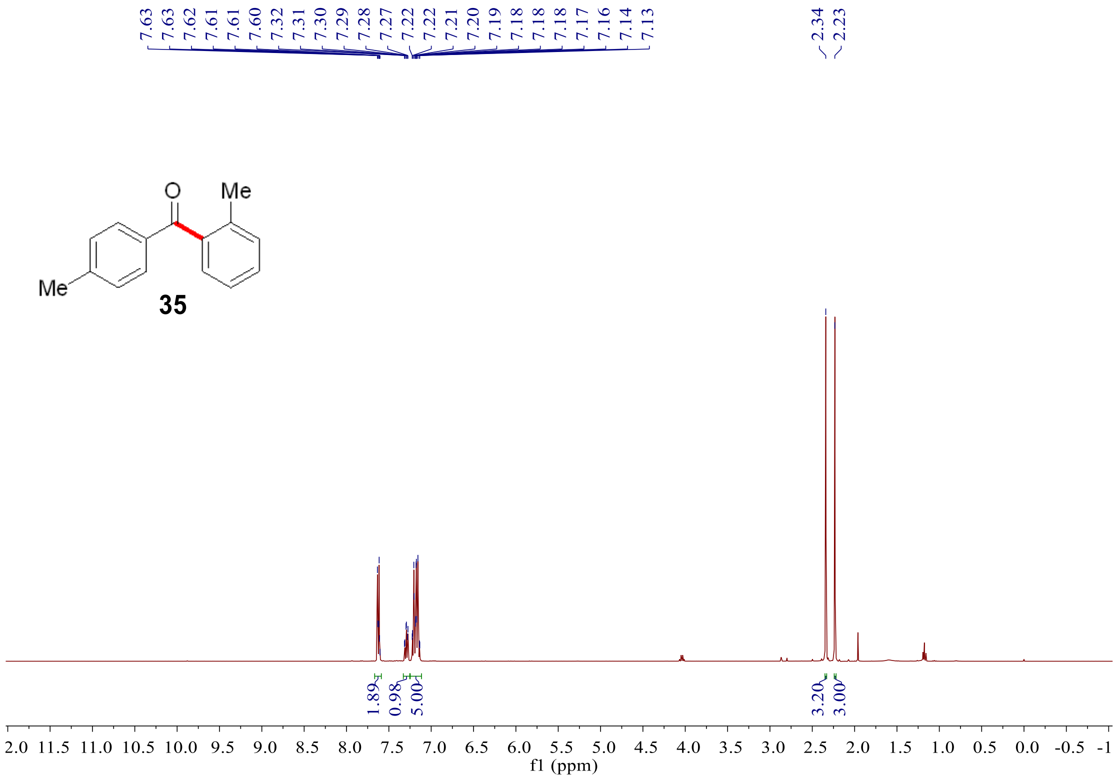

Supplementary Figure 104. ^{1}H NMR spectra for compound **35**

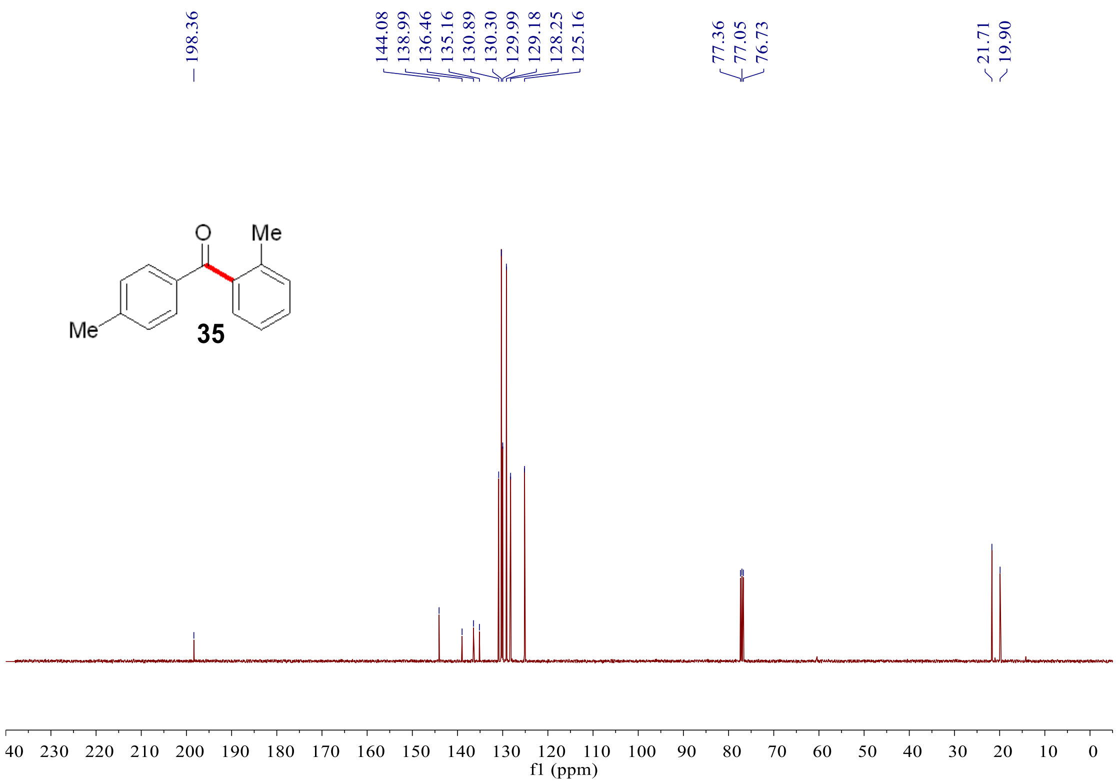

Supplementary Figure 105. ^{13}C NMR spectra for compound **35**

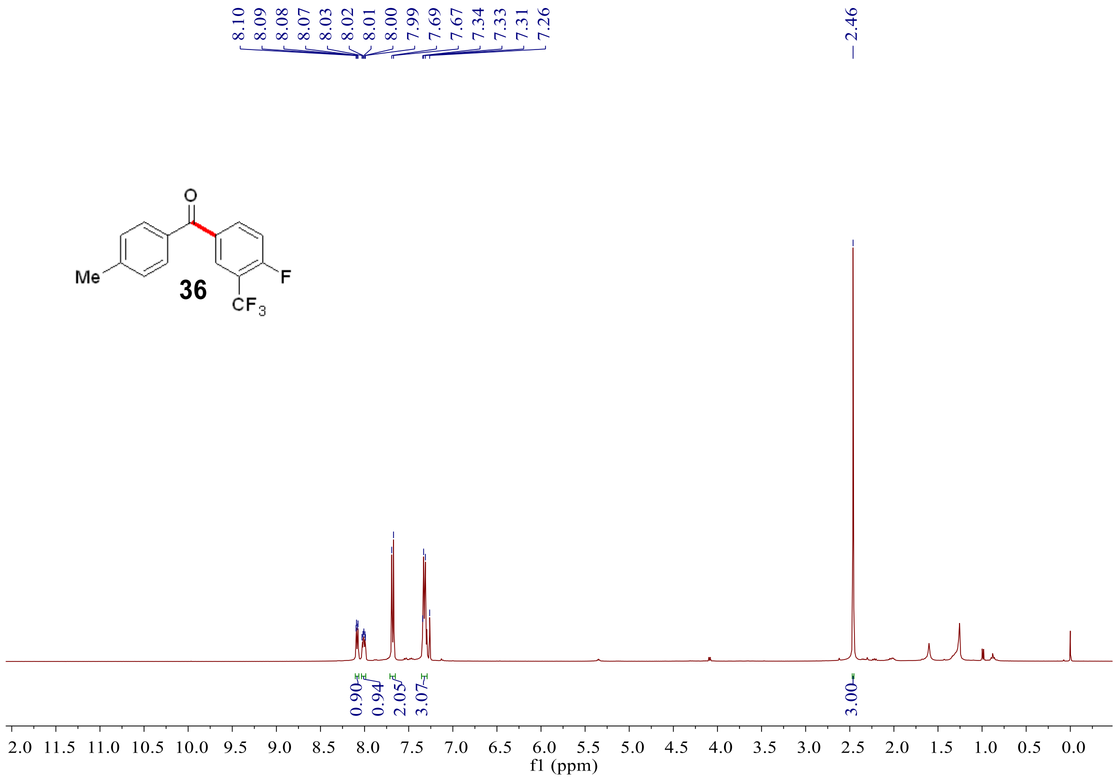

Supplementary Figure 106. ^{1}H NMR spectra for compound **36**

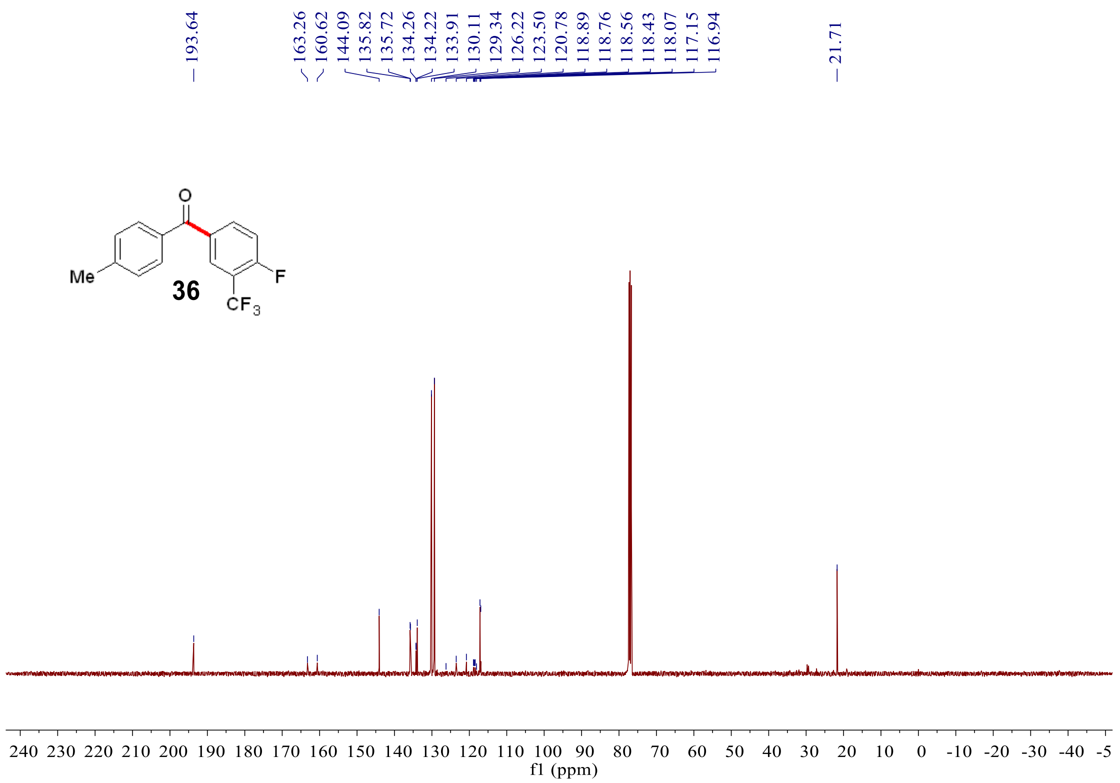

Supplementary Figure 107. ^{13}C NMR spectra for compound **36**

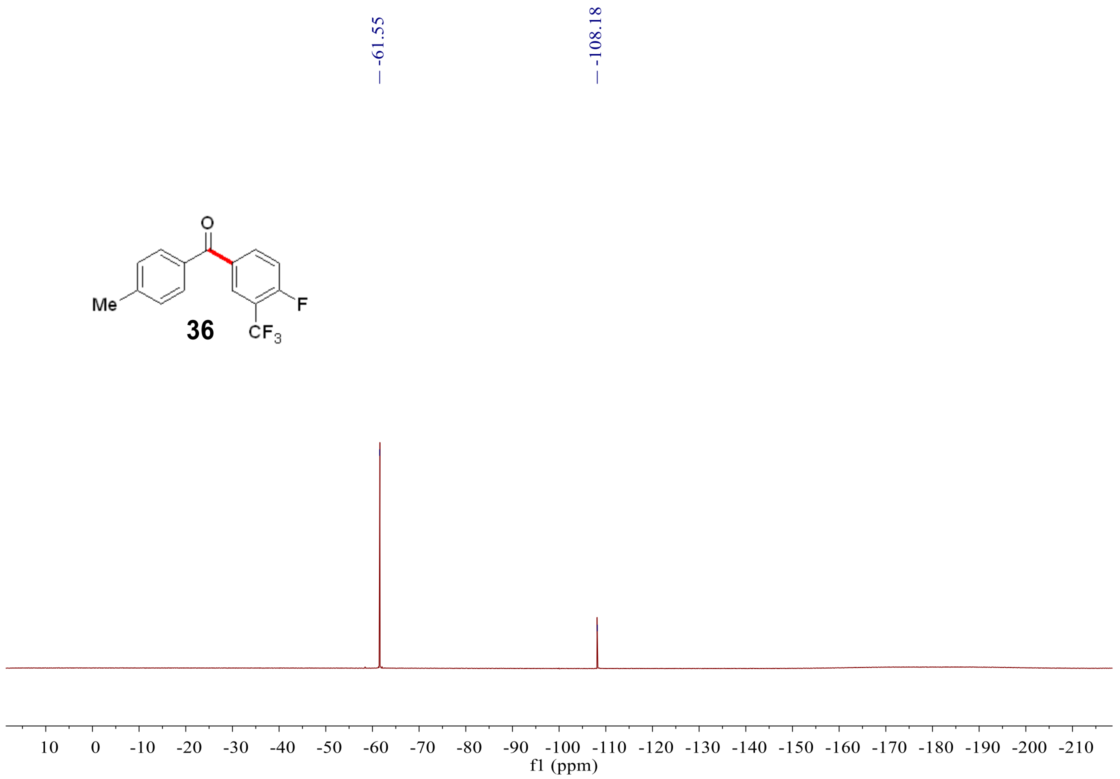

Supplementary Figure 108. ^{19}F NMR spectra for compound **36**

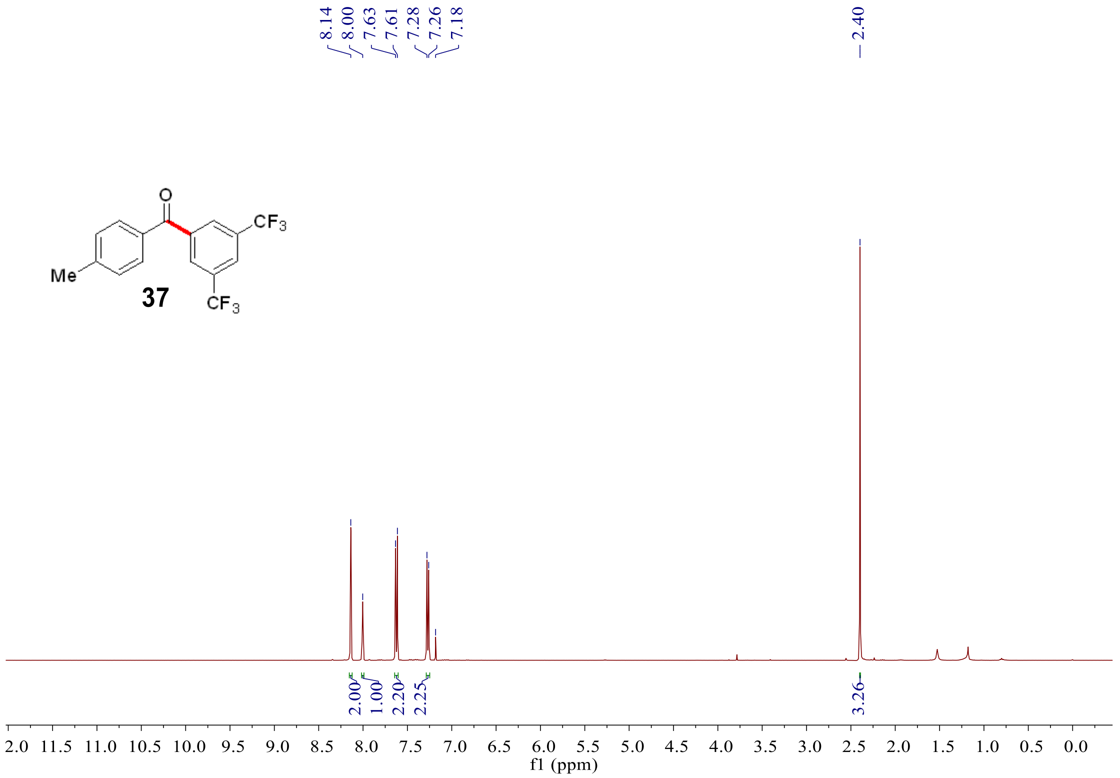

Supplementary Figure 109. ^{1}H NMR spectra for compound **37**

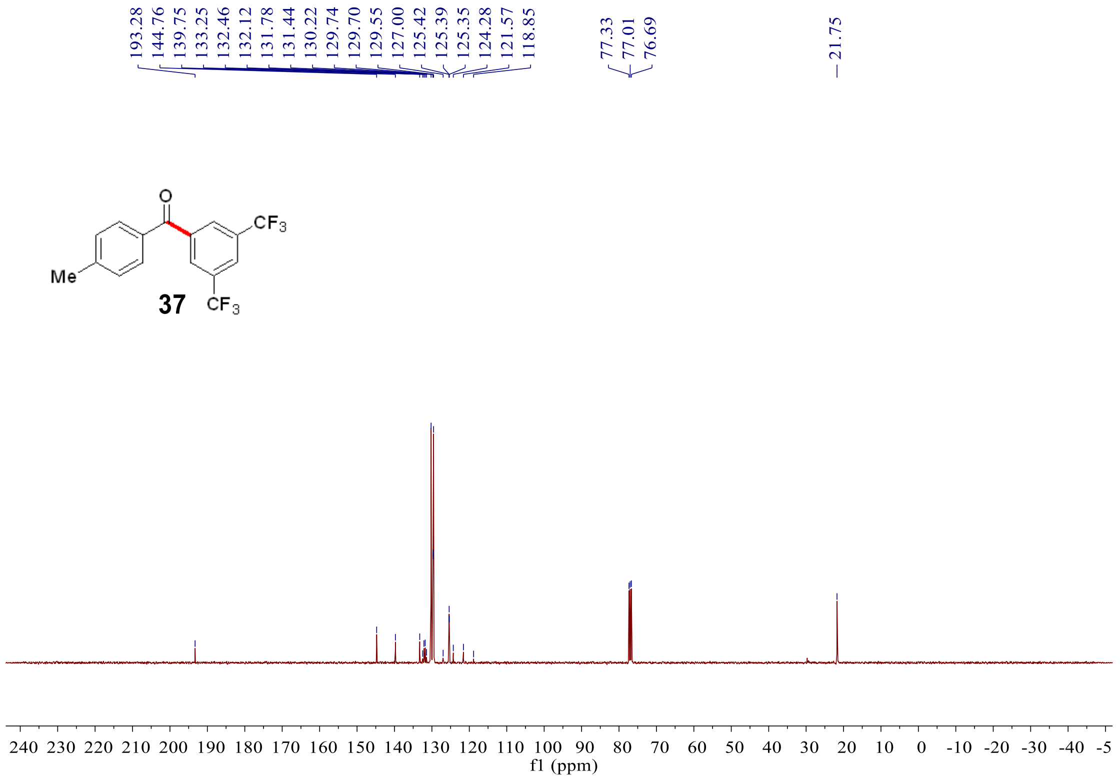

Supplementary Figure 110. ^{13}C NMR spectra for compound **37**

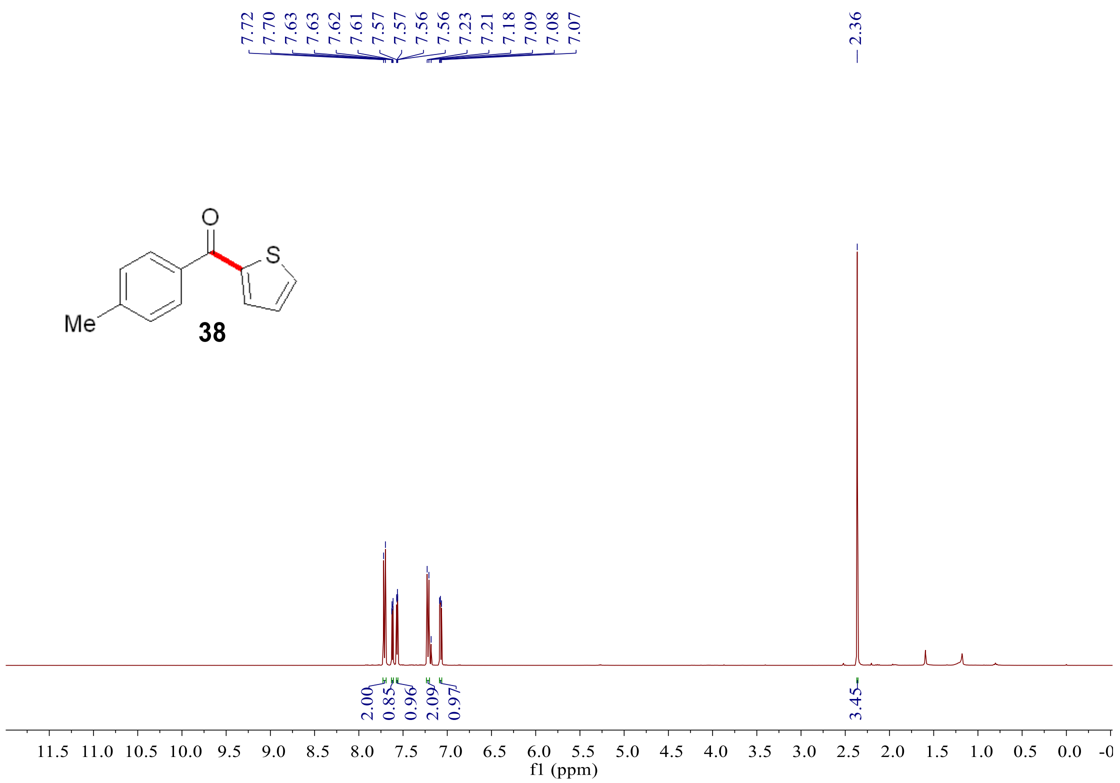

Supplementary Figure 111. ^{1}H NMR spectra for compound **38**

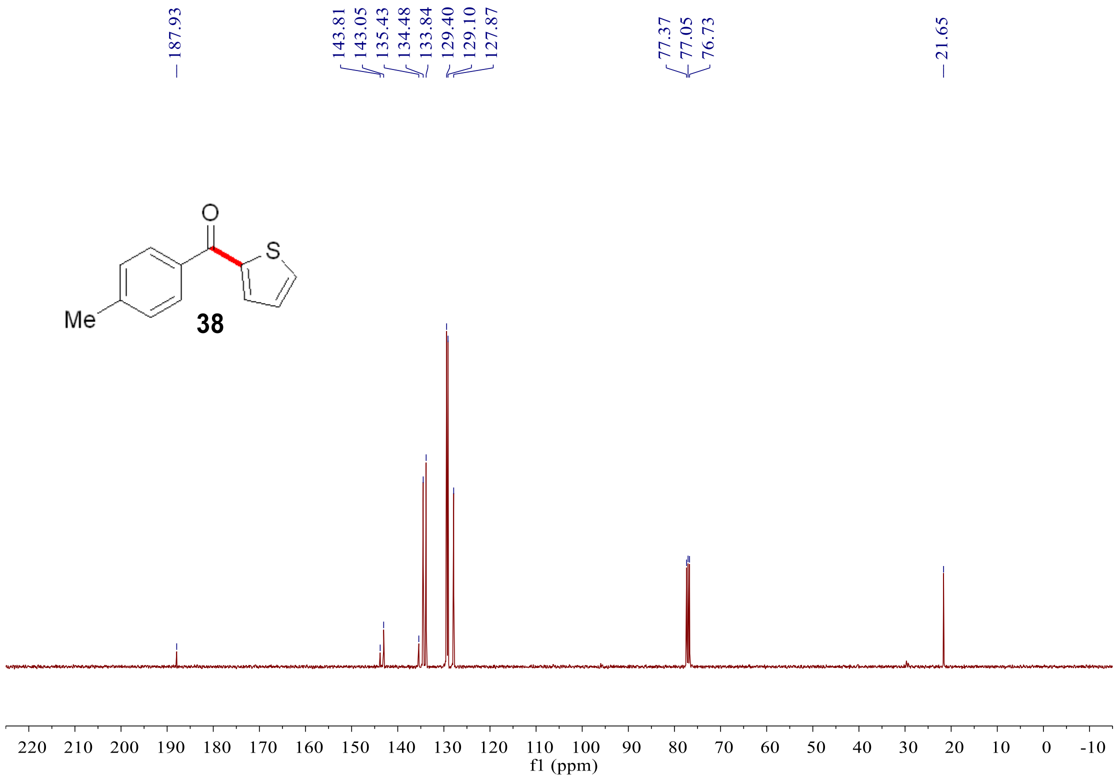

Supplementary Figure 112. ^{13}C NMR spectra for compound **38**

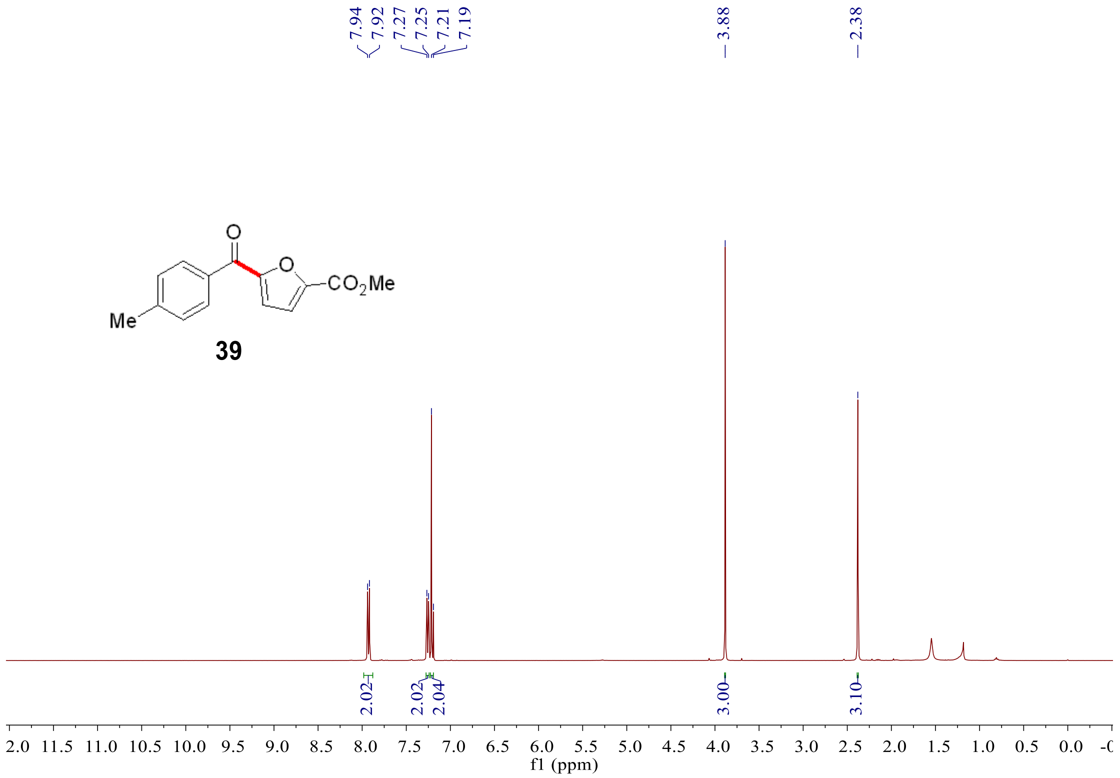

Supplementary Figure 113. ^{1}H NMR spectra for compound **39**

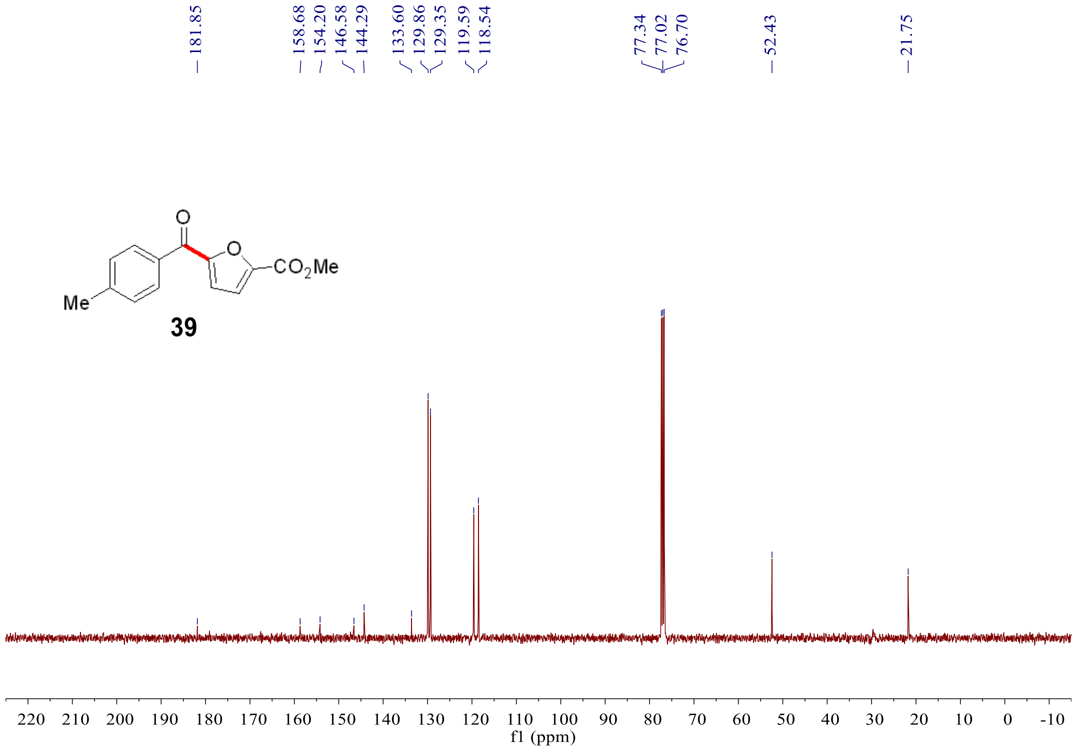

Supplementary Figure 114. ^{13}C NMR spectra for compound **39**

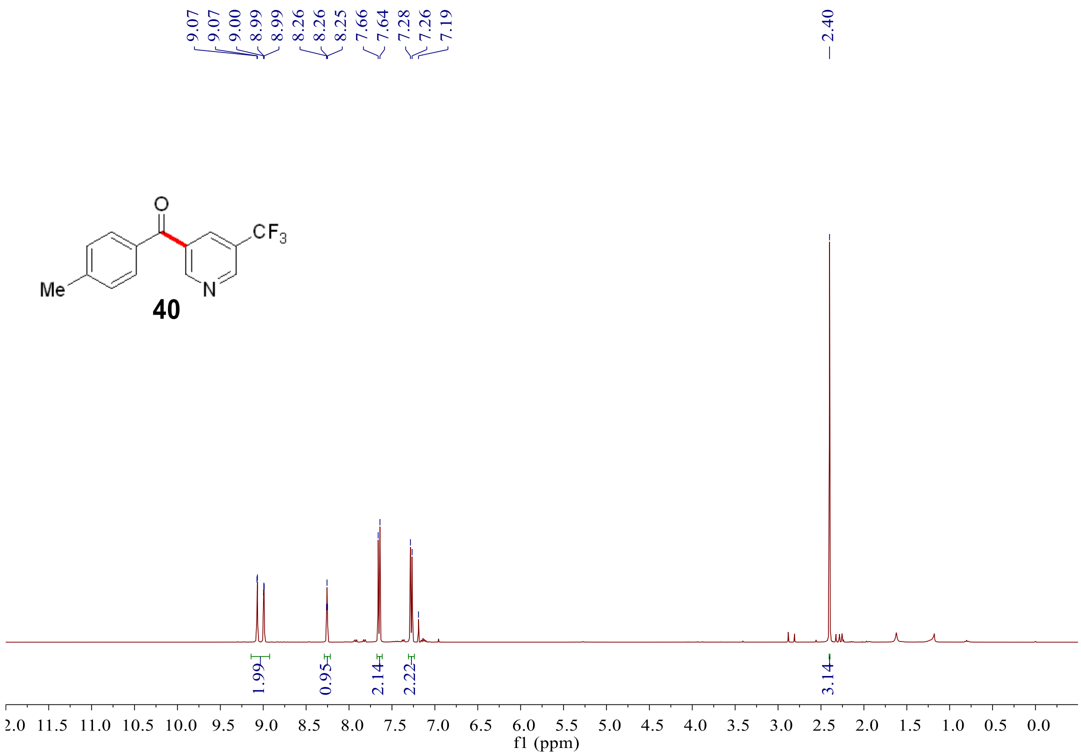

Supplementary Figure 115. ^{1}H NMR spectra for compound **40**

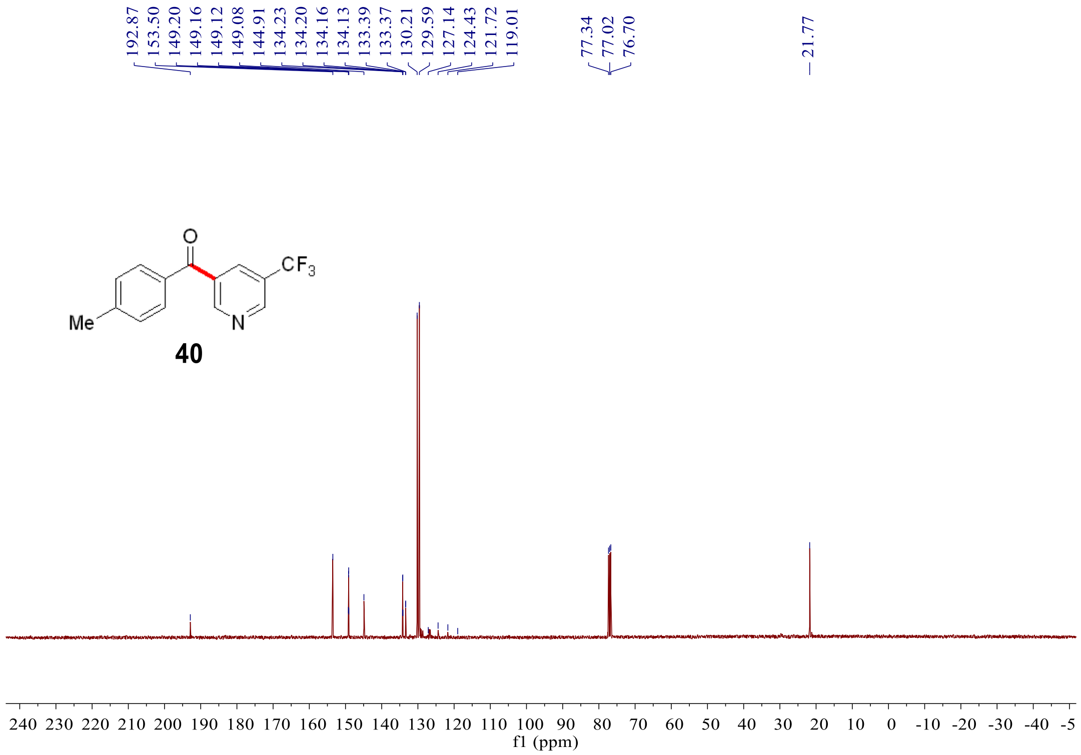

Supplementary Figure 116. ^{1}H NMR spectra for compound **40**

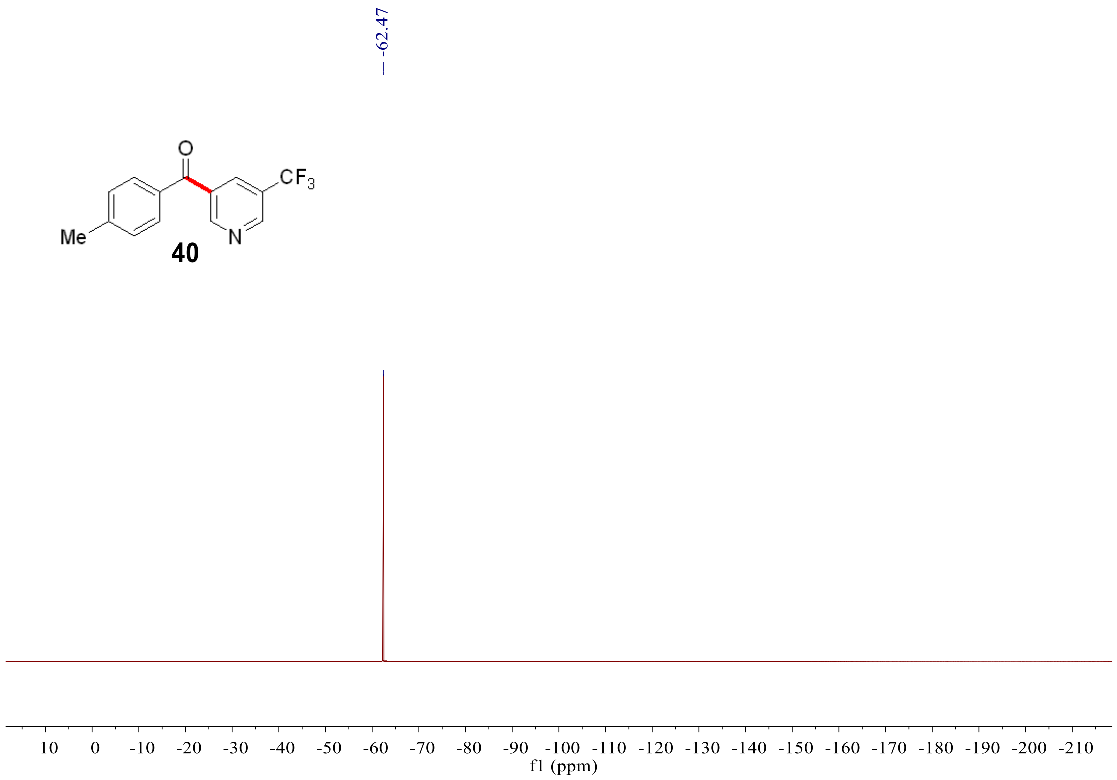

Supplementary Figure 117. ^{19}F NMR spectra for compound **40**

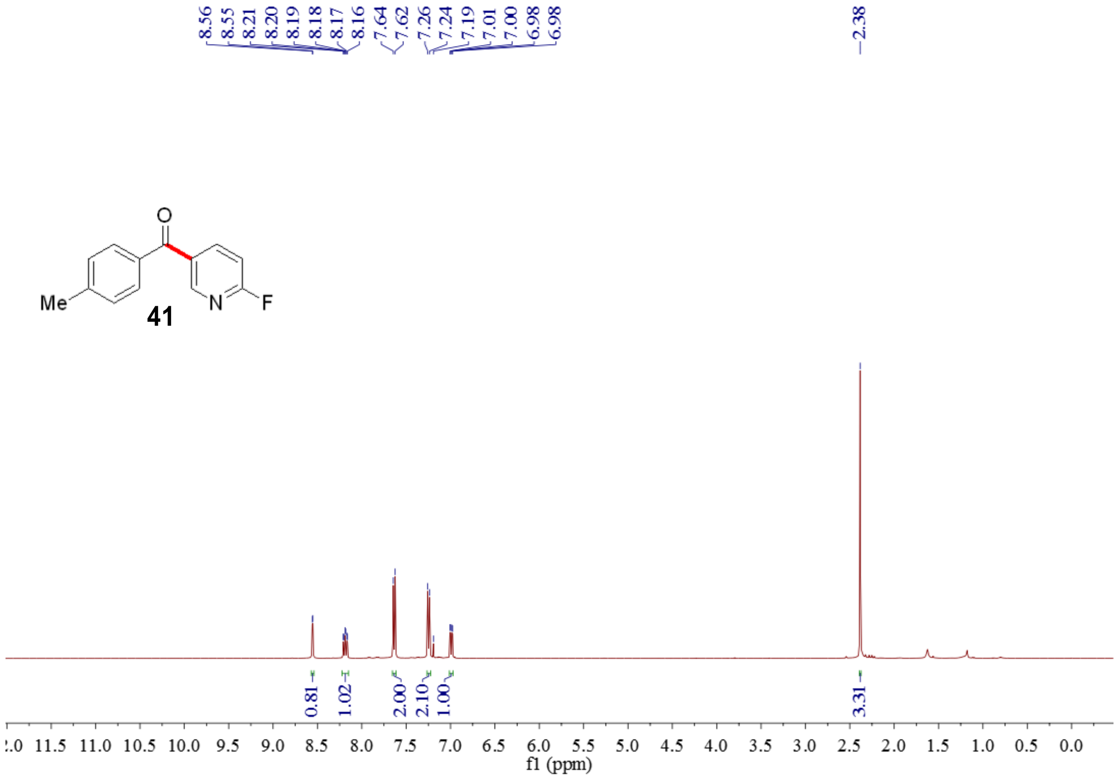

Supplementary Figure 118. ^{1}H NMR spectra for compound **41**

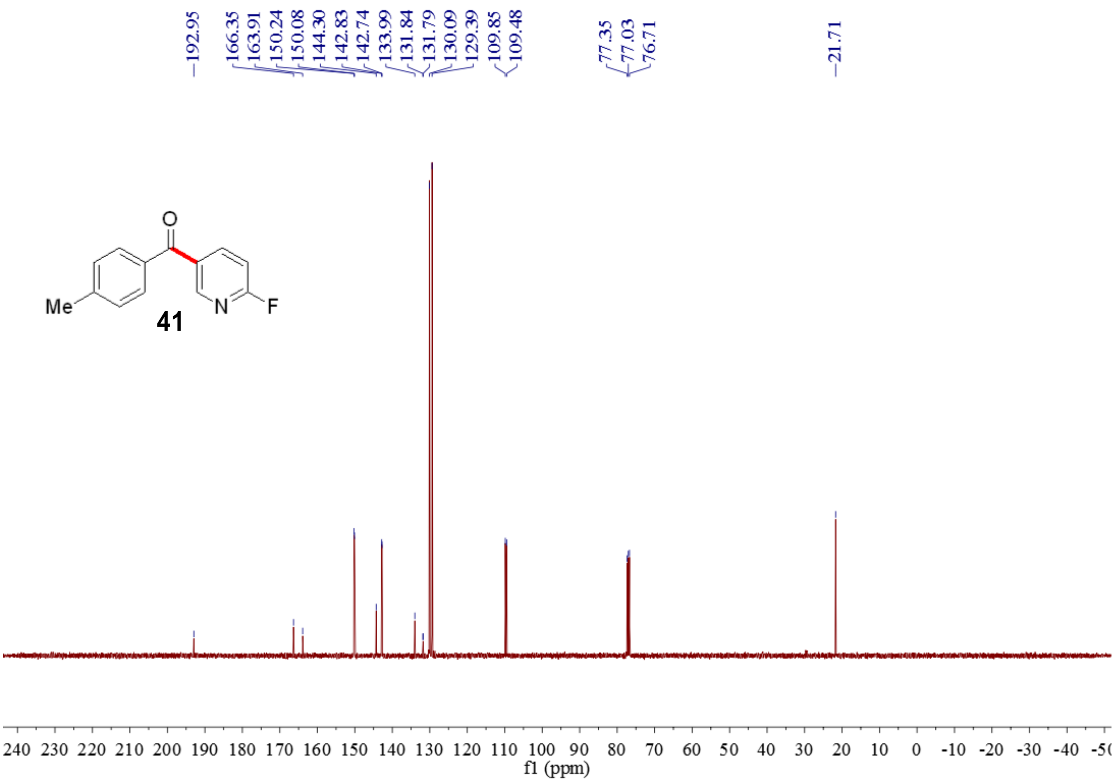

Supplementary Figure 119. ^{13}C NMR spectra for compound **41**

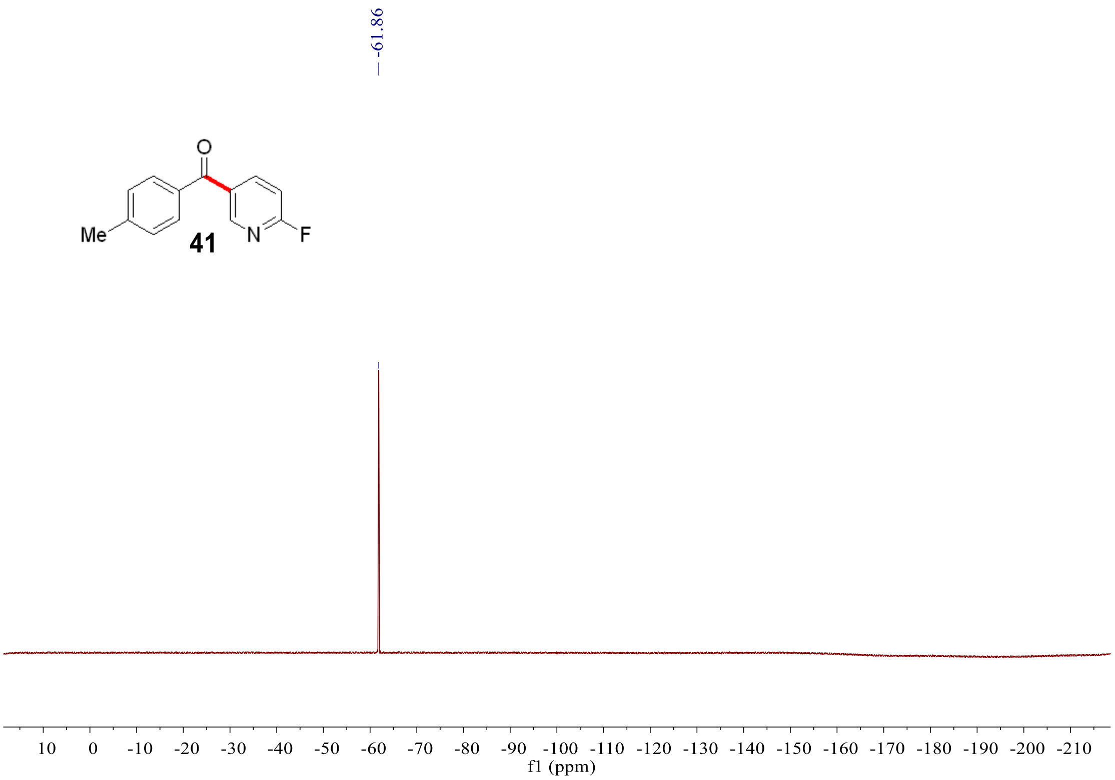

Supplementary Figure 120. ^{19}F NMR spectra for compound **41**

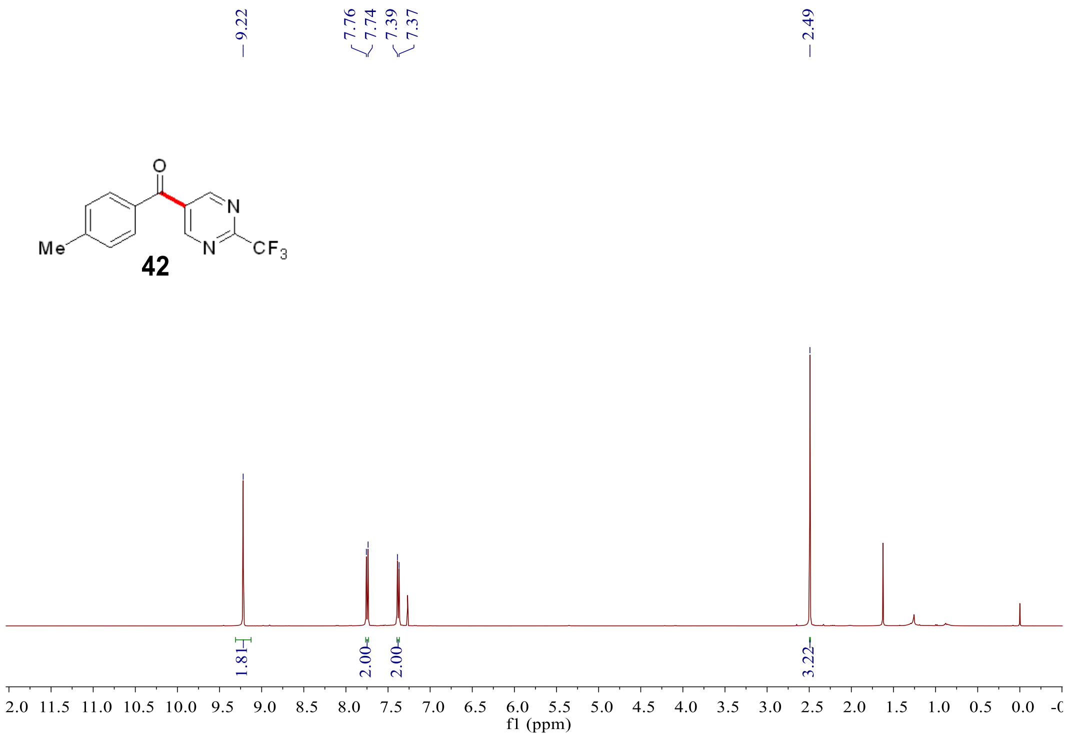

Supplementary Figure 121. ^{1}H NMR spectra for compound **42**

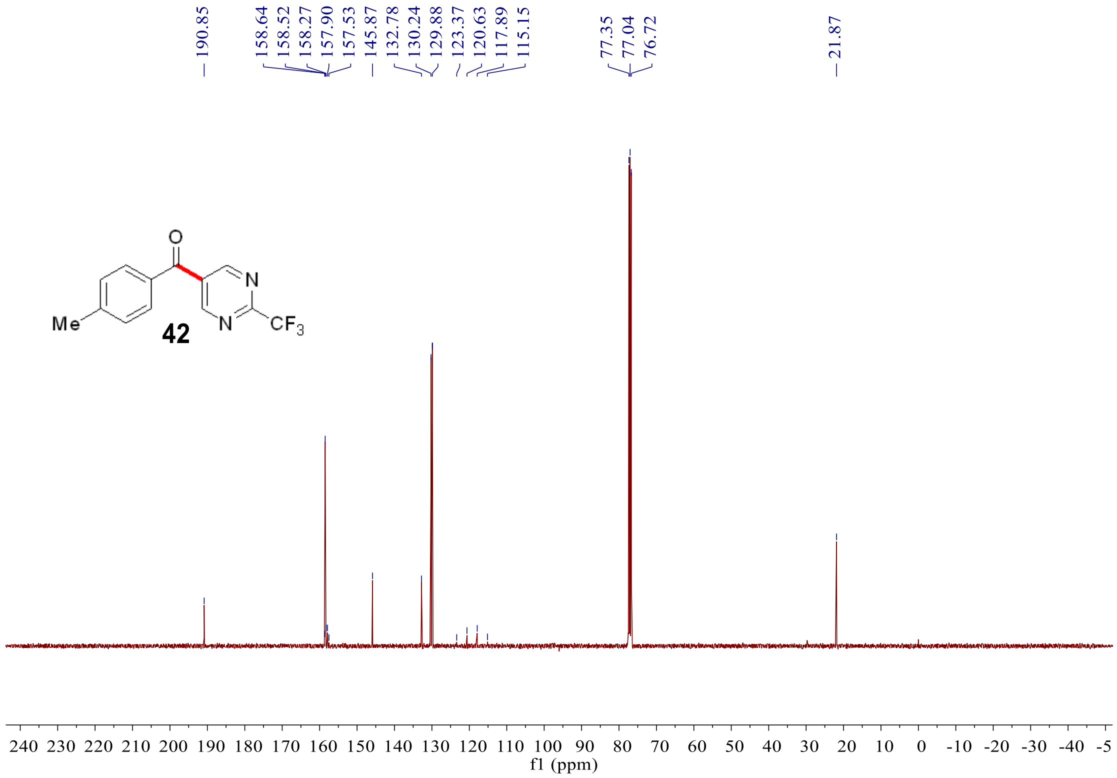

Supplementary Figure 122. ^{13}C NMR spectra for compound **42**

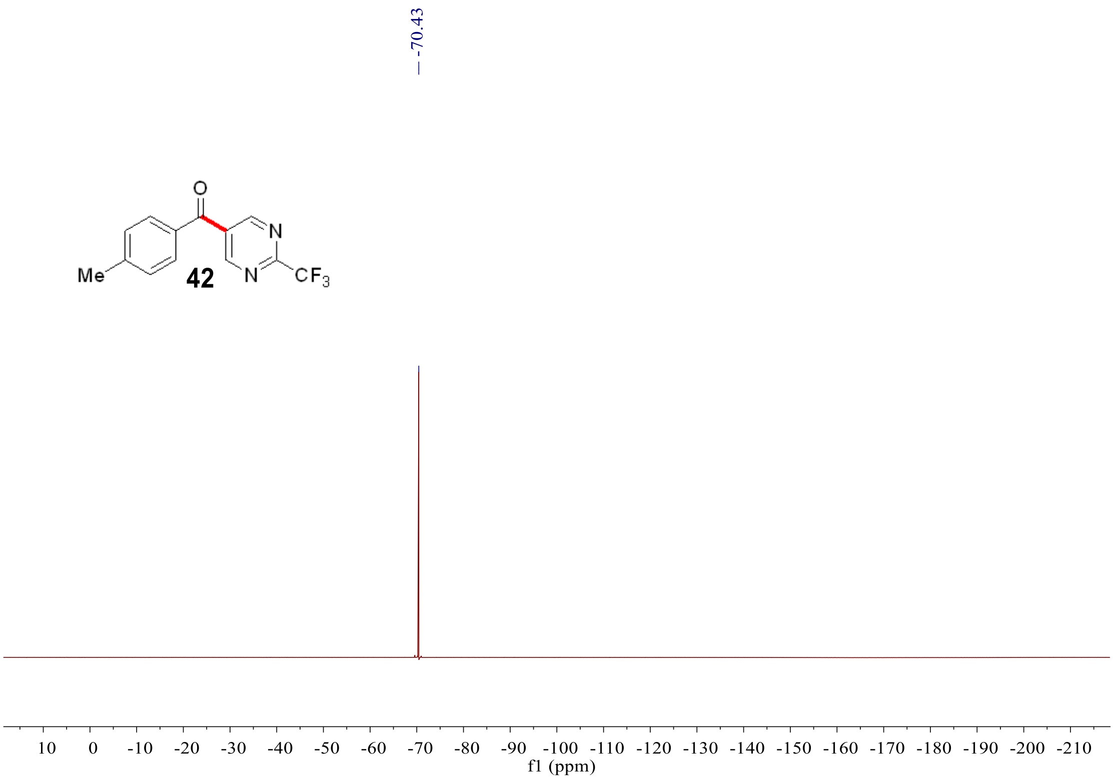

Supplementary Figure 123. ^{19}F NMR spectra for compound **42**

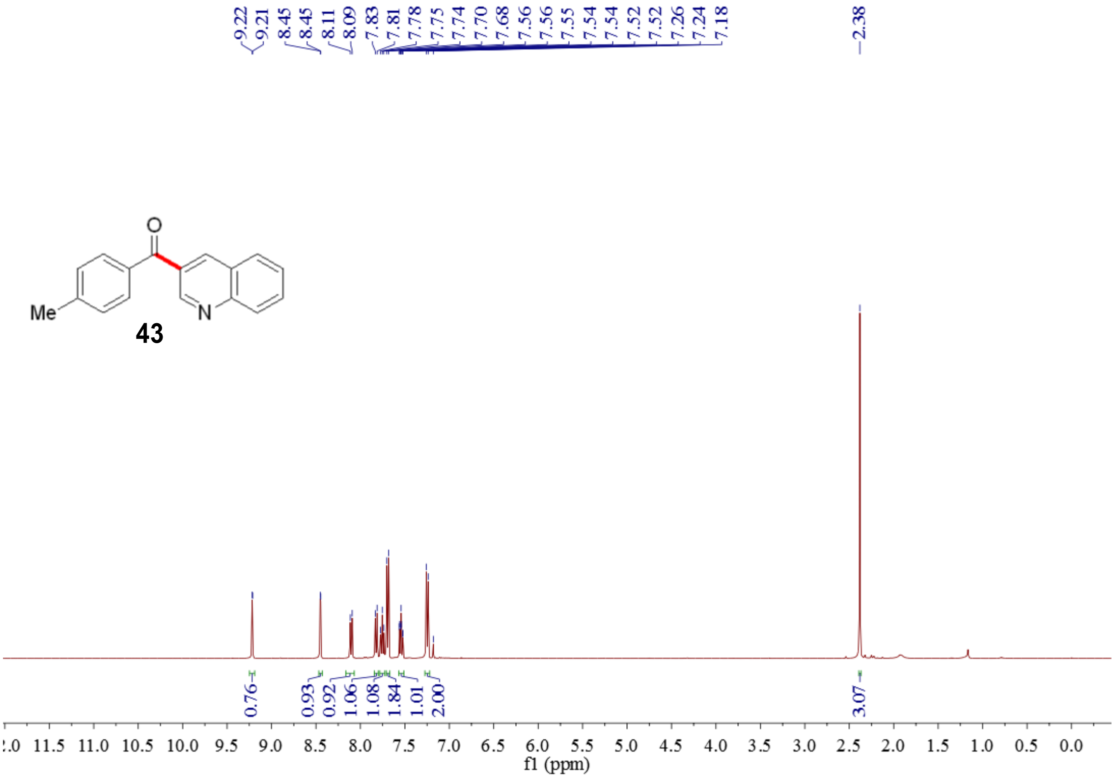

Supplementary Figure 124. ^{1}H NMR spectra for compound **43**

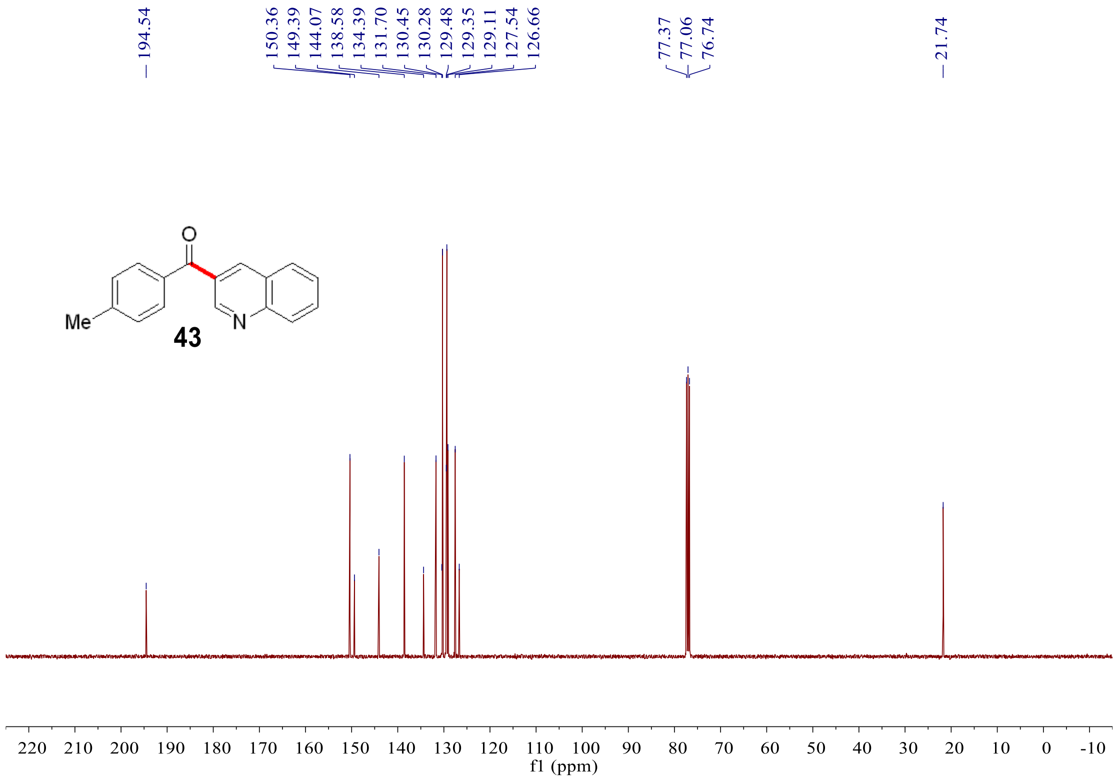

Supplementary Figure 125. ^{13}C NMR spectra for compound **43**

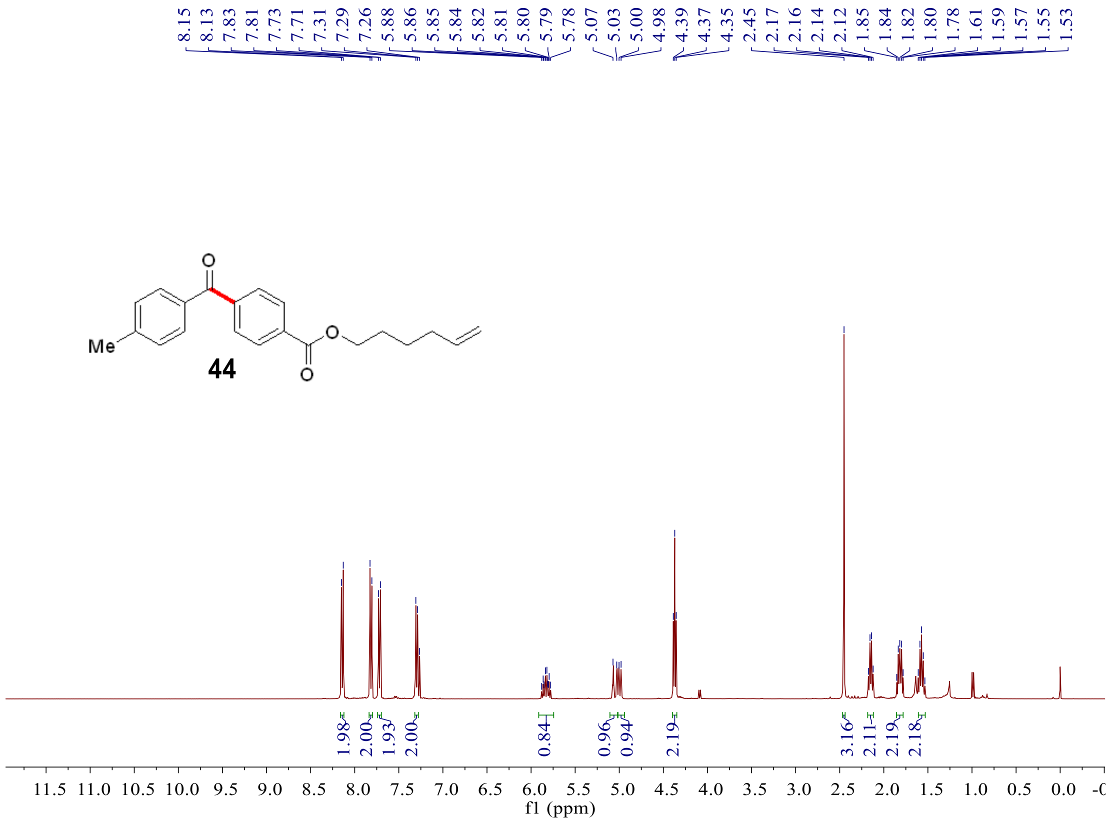

Supplementary Figure 126. ^{1}H NMR spectra for compound **44**

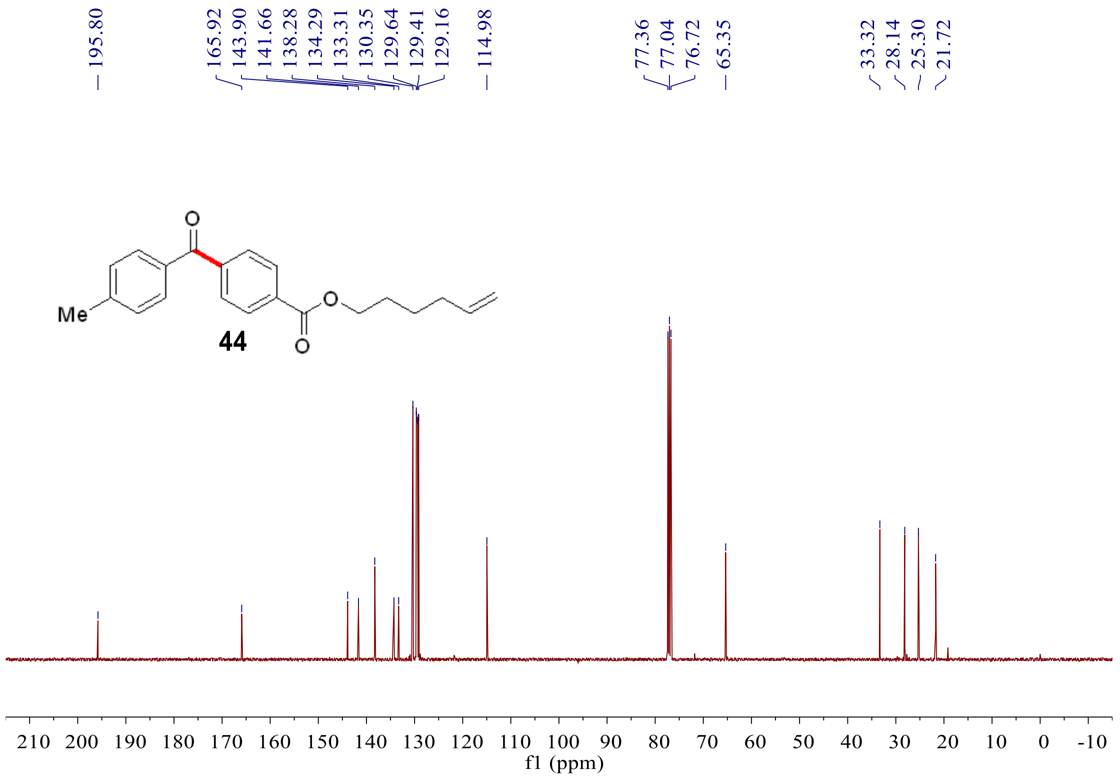

Supplementary Figure 127. ^{13}C NMR spectra for compound **44**

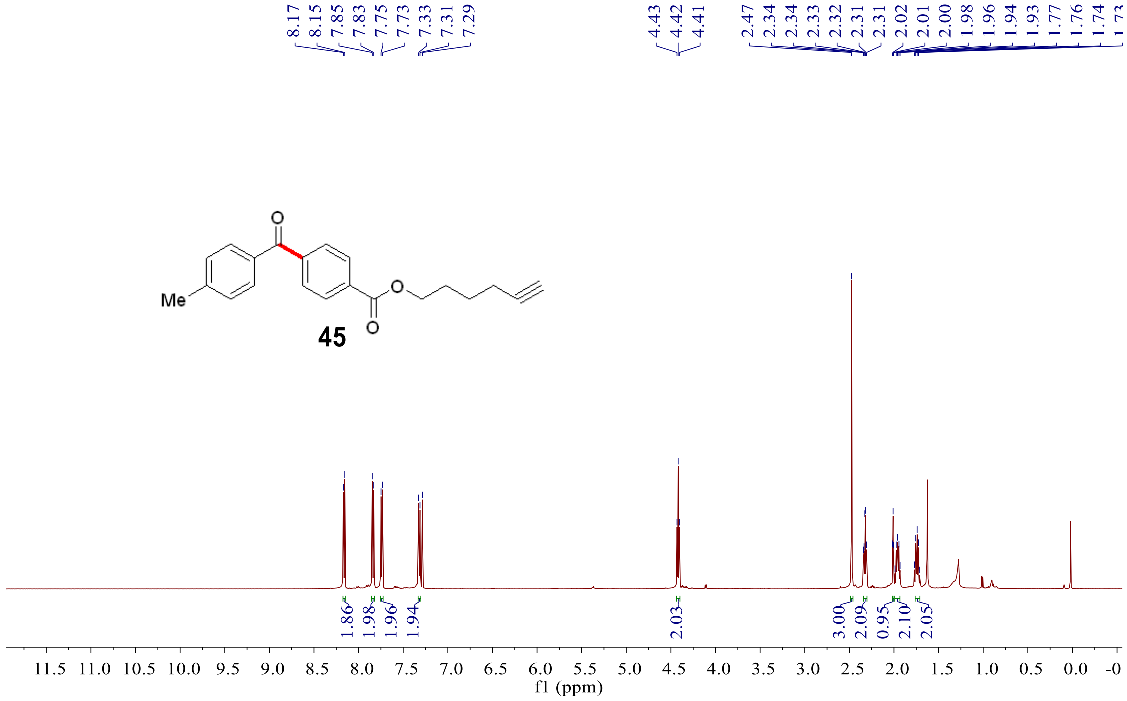

Supplementary Figure 128. ^{1}H NMR spectra for compound **45**

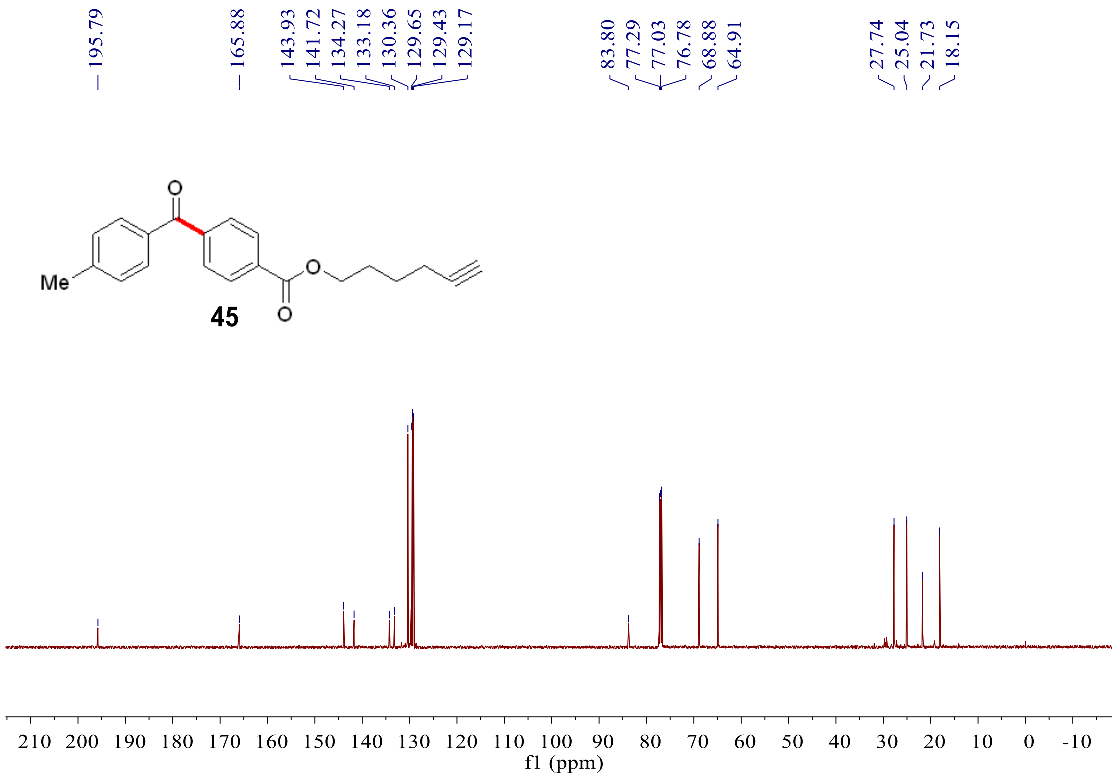

Supplementary Figure 129. ^{13}C NMR spectra for compound **45**

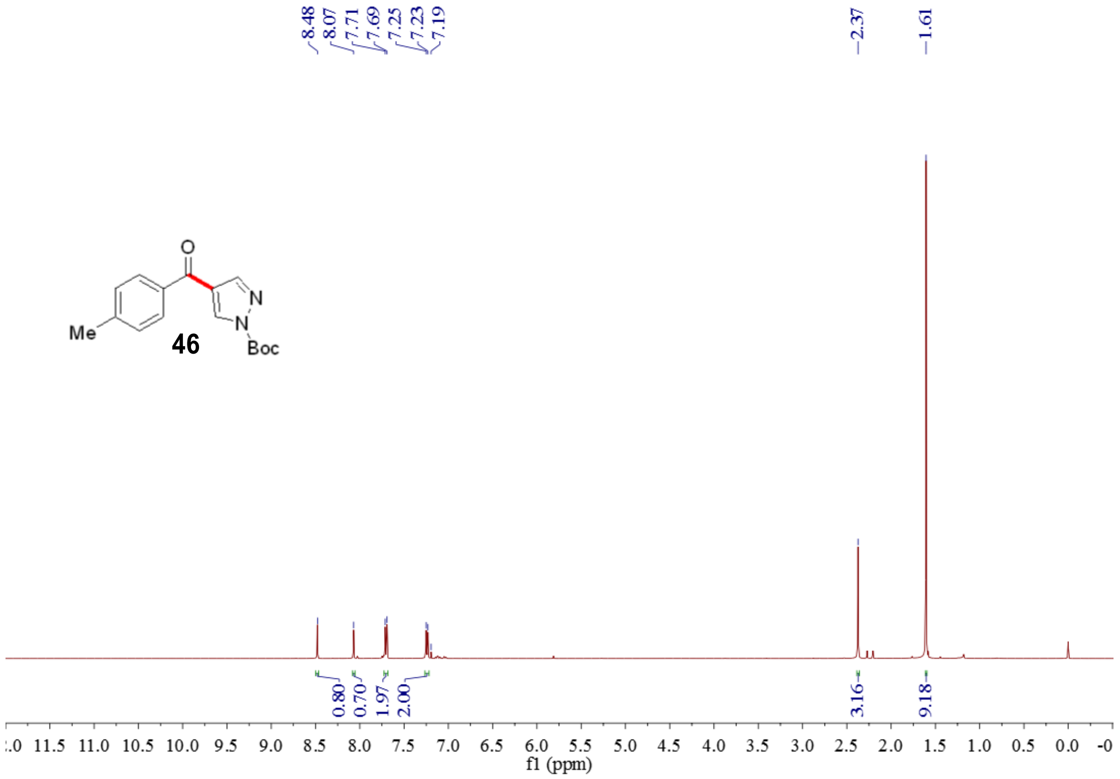

Supplementary Figure 130. ^{1}H NMR spectra for compound **46**

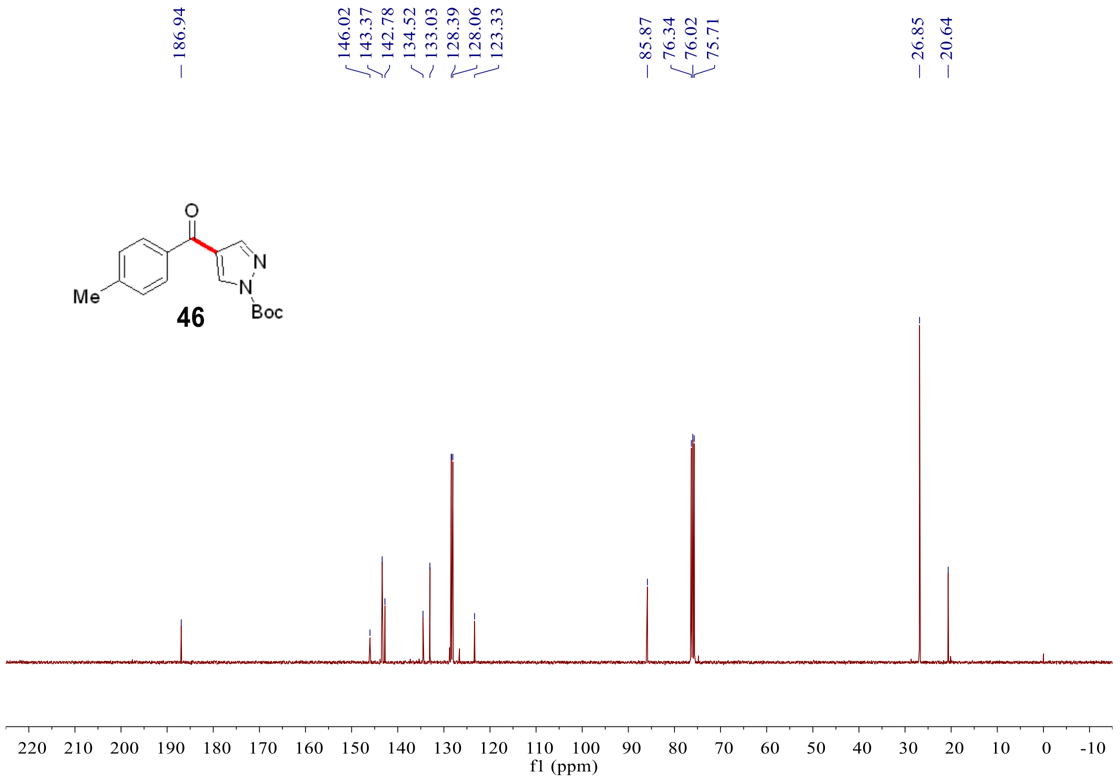

Supplementary Figure 131. ^{13}C NMR spectra for compound **46**

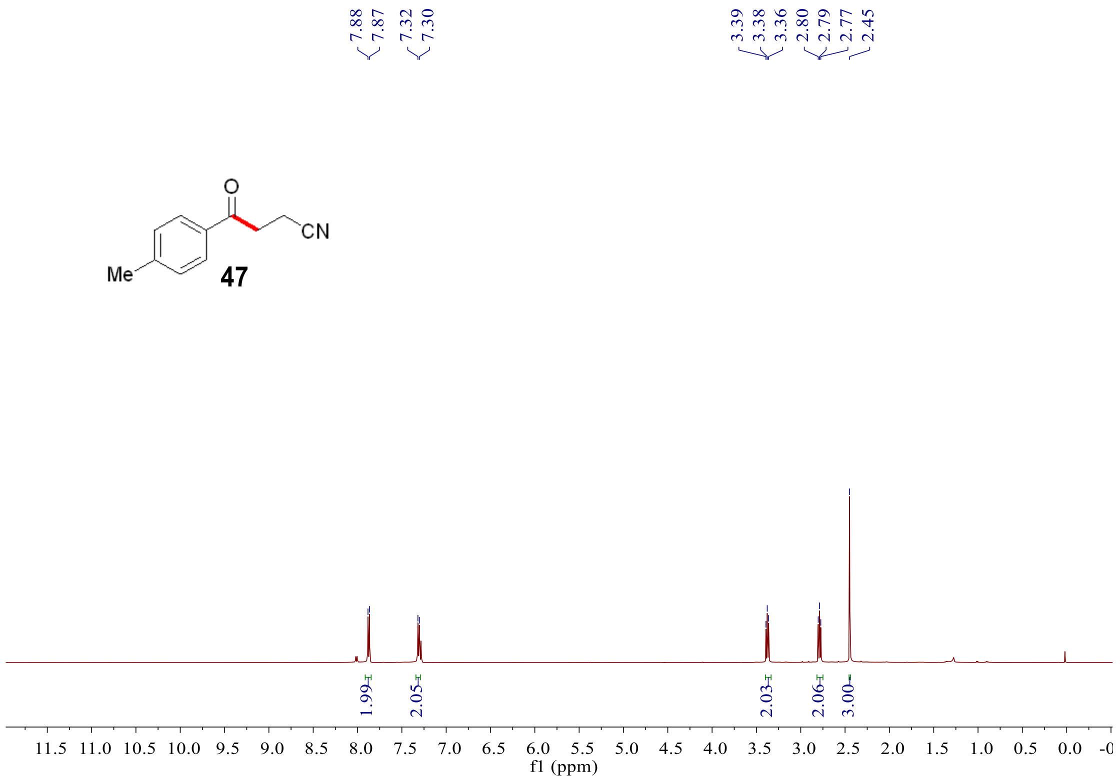

Supplementary Figure 132. ^{1}H NMR spectra for compound **47**

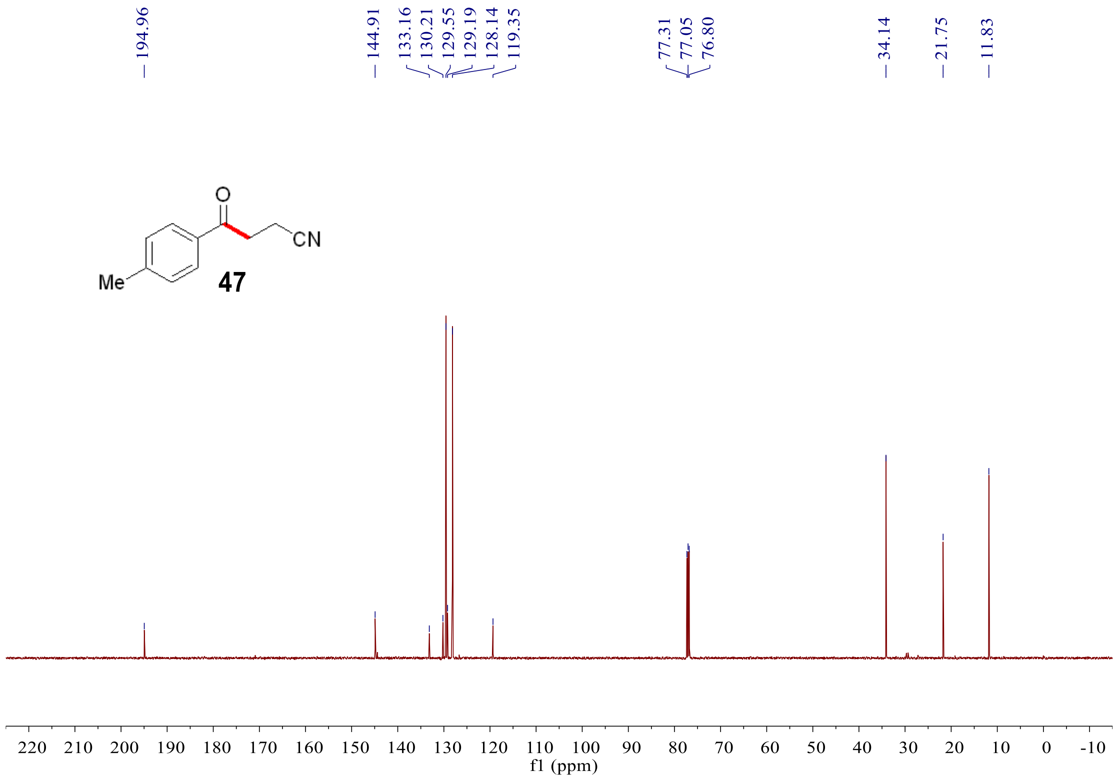

Supplementary Figure 133. ^{13}C NMR spectra for compound **47**

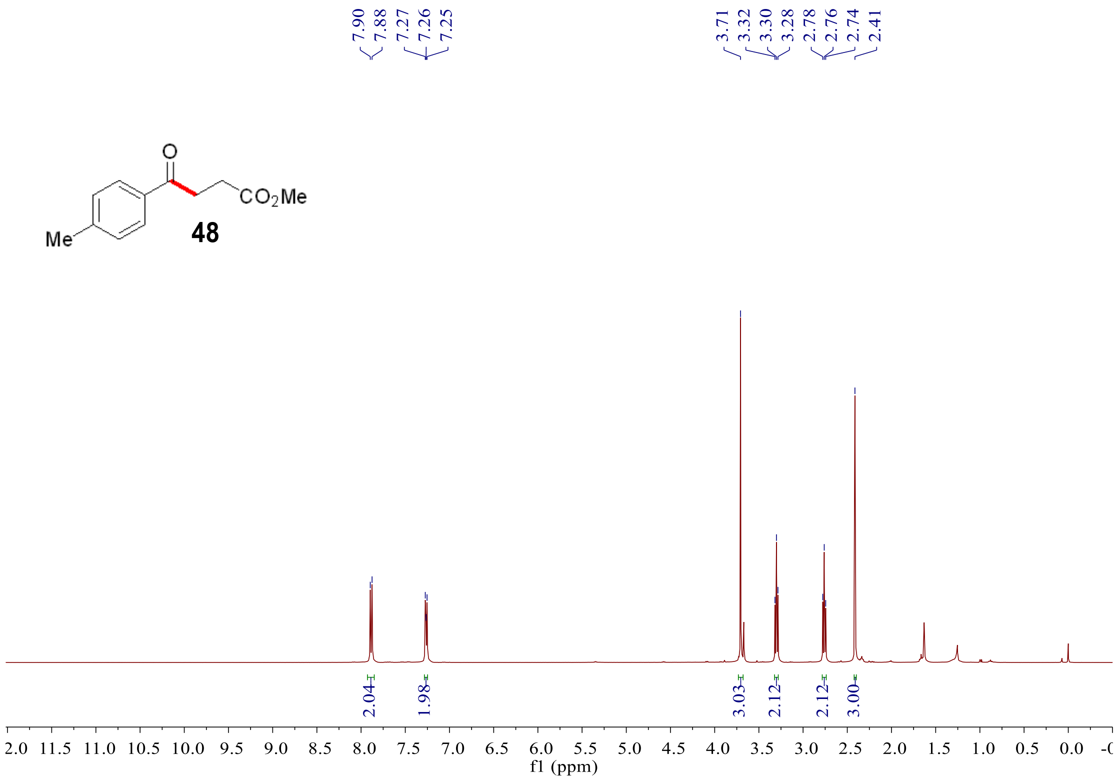

Supplementary Figure 134. ^{1}H NMR spectra for compound **48**

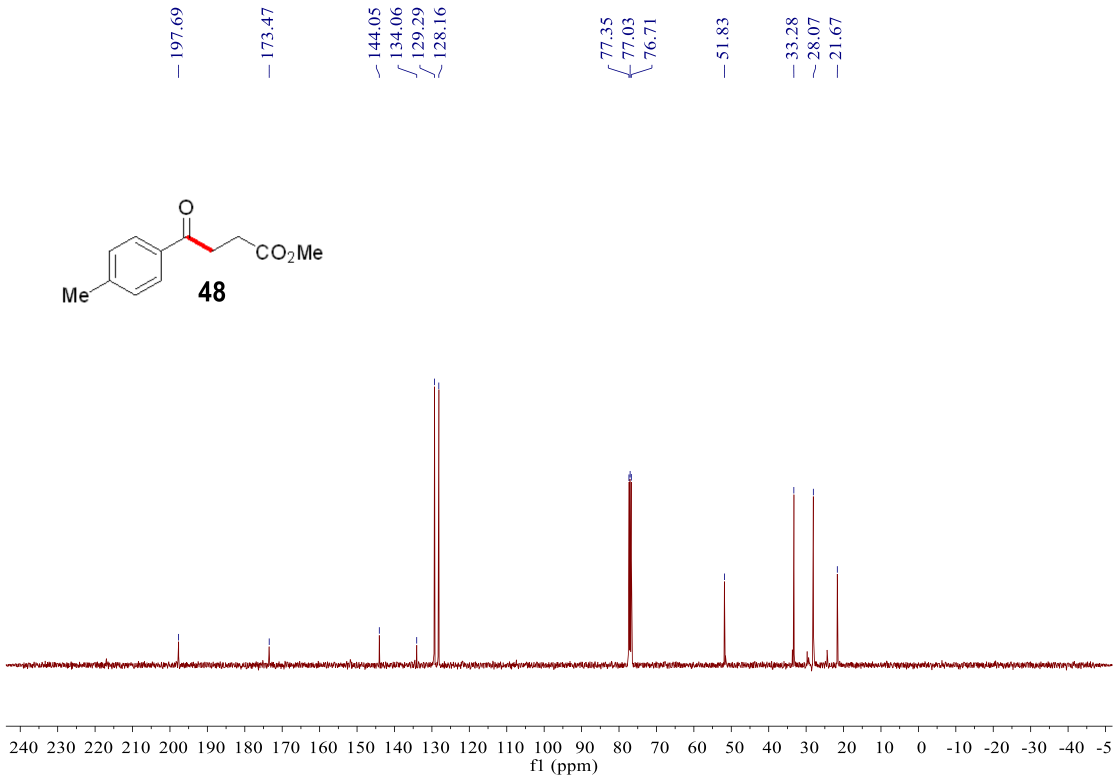

Supplementary Figure 135. ^{13}C NMR spectra for compound **48**

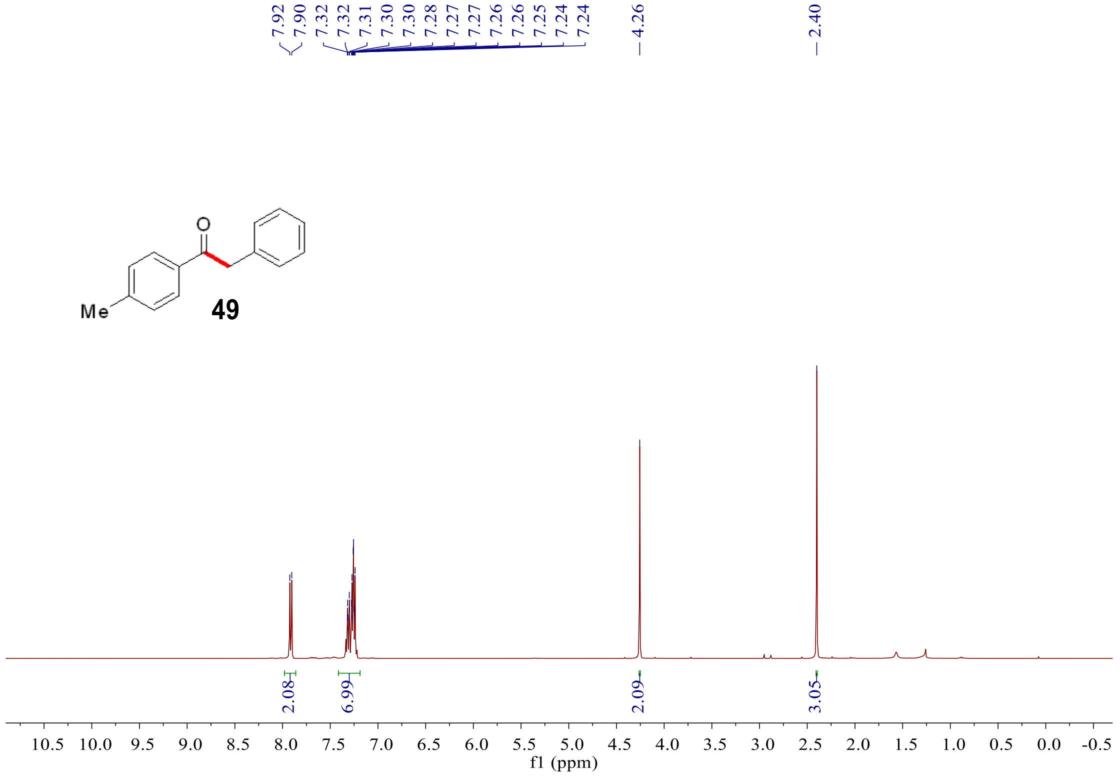

Supplementary Figure 136. ^{1}H NMR spectra for compound **49**

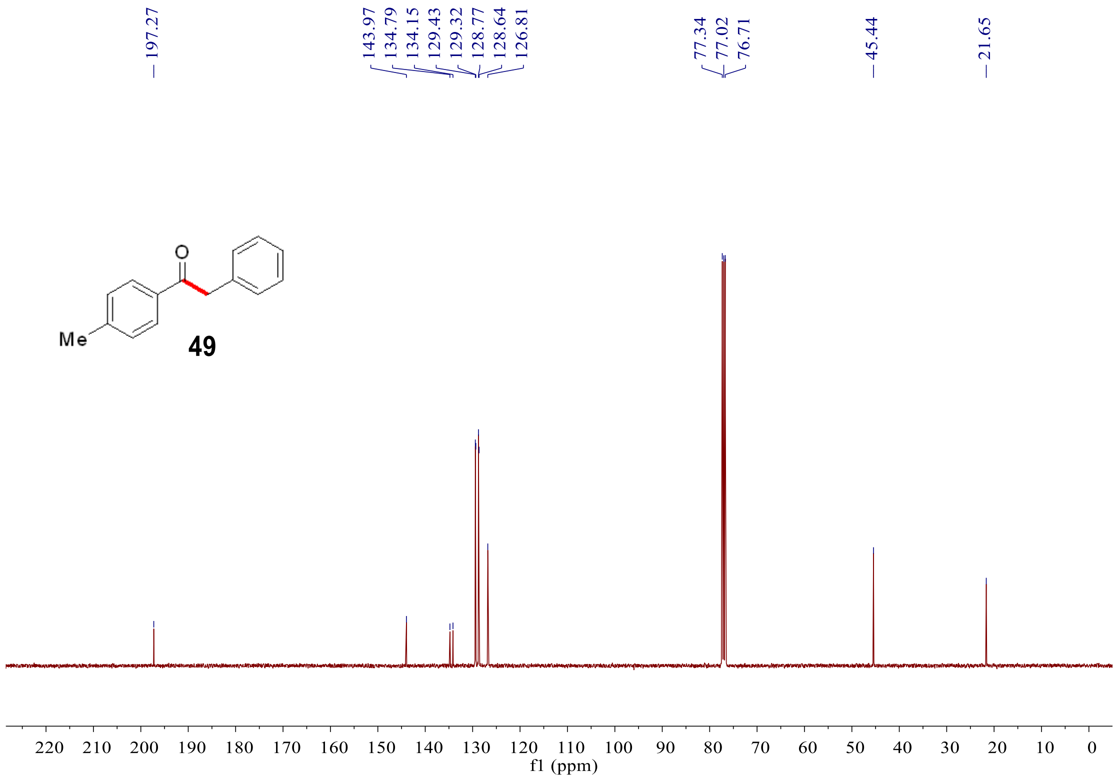

Supplementary Figure 137. ^{13}C NMR spectra for compound **49**

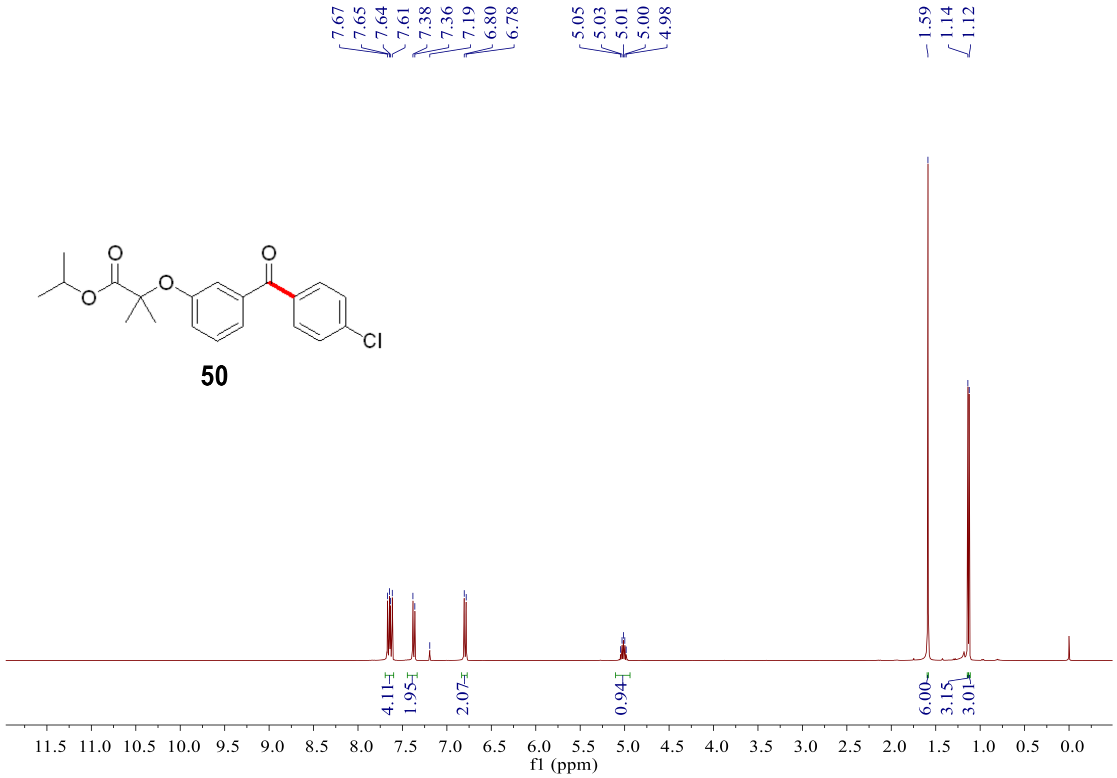

Supplementary Figure 138. ^{1}H NMR spectra for compound **50**

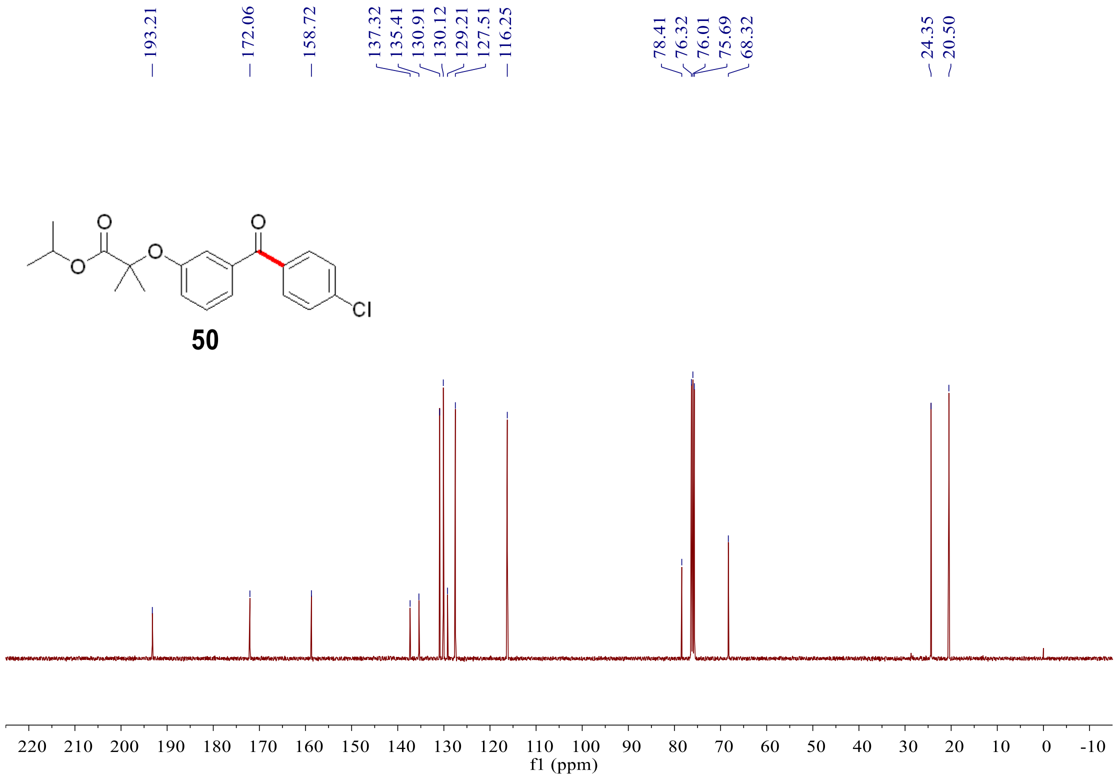

Supplementary Figure 139. ^{13}C NMR spectra for compound **50**

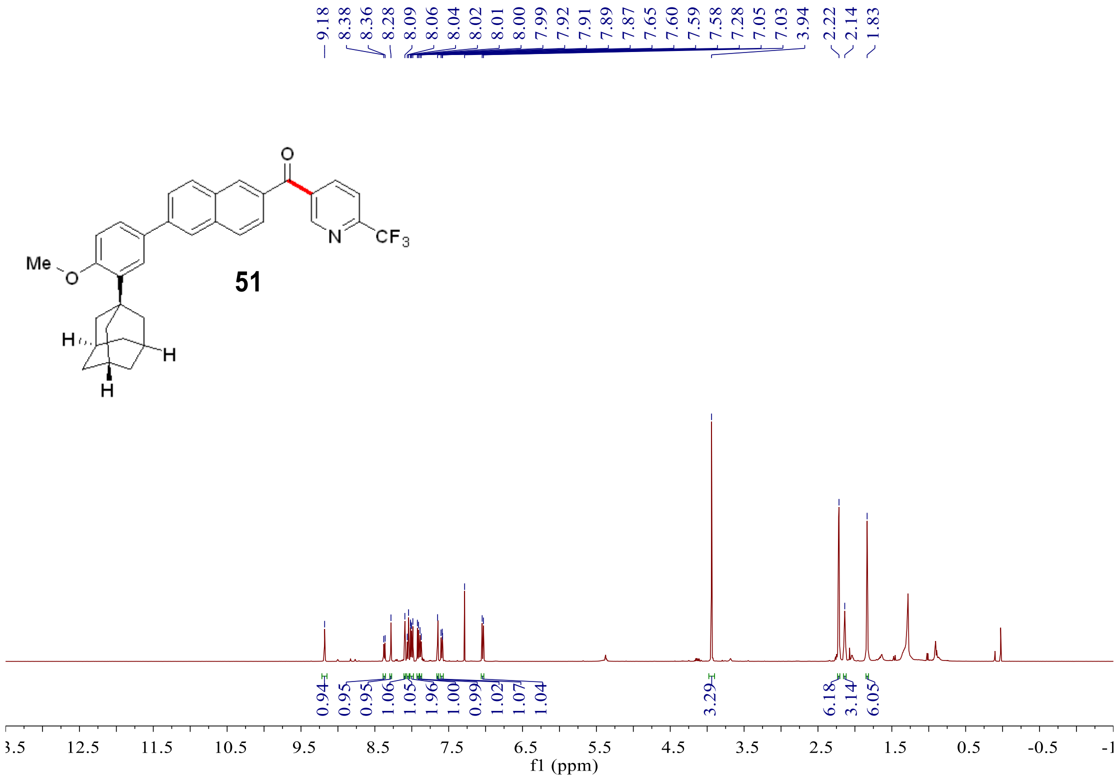

Supplementary Figure 140. ^{1}H NMR spectra for compound **51**

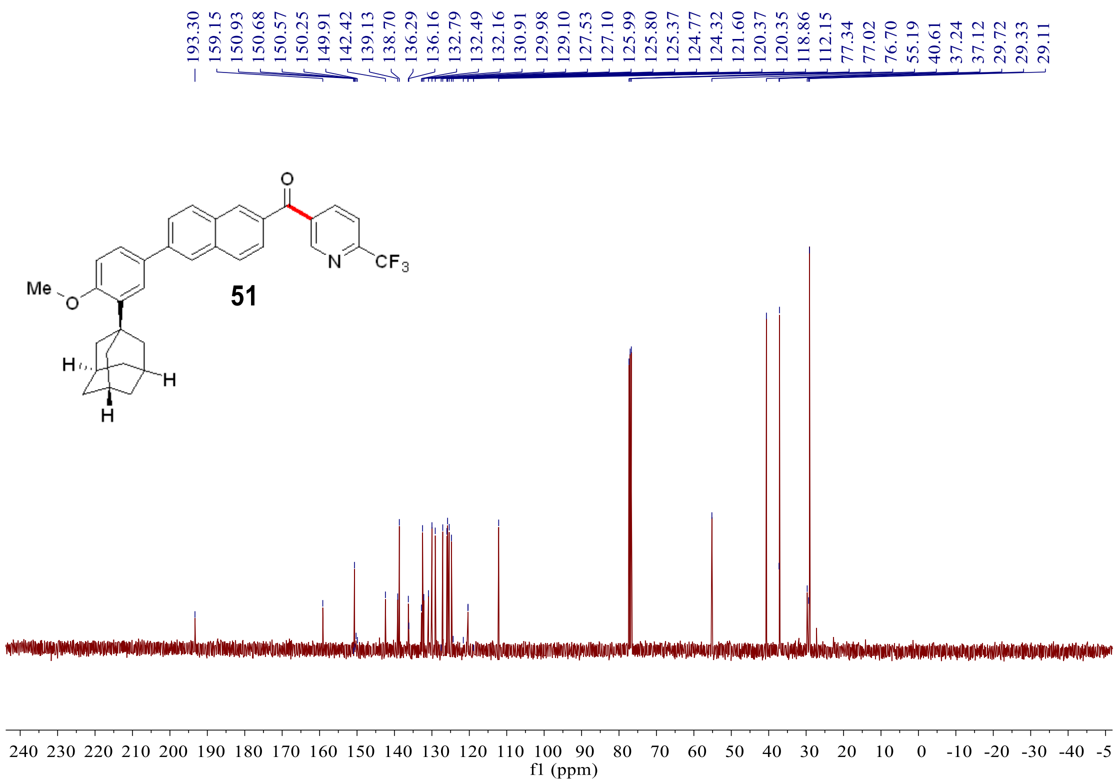

Supplementary Figure 141. ^{13}C NMR spectra for compound **51**

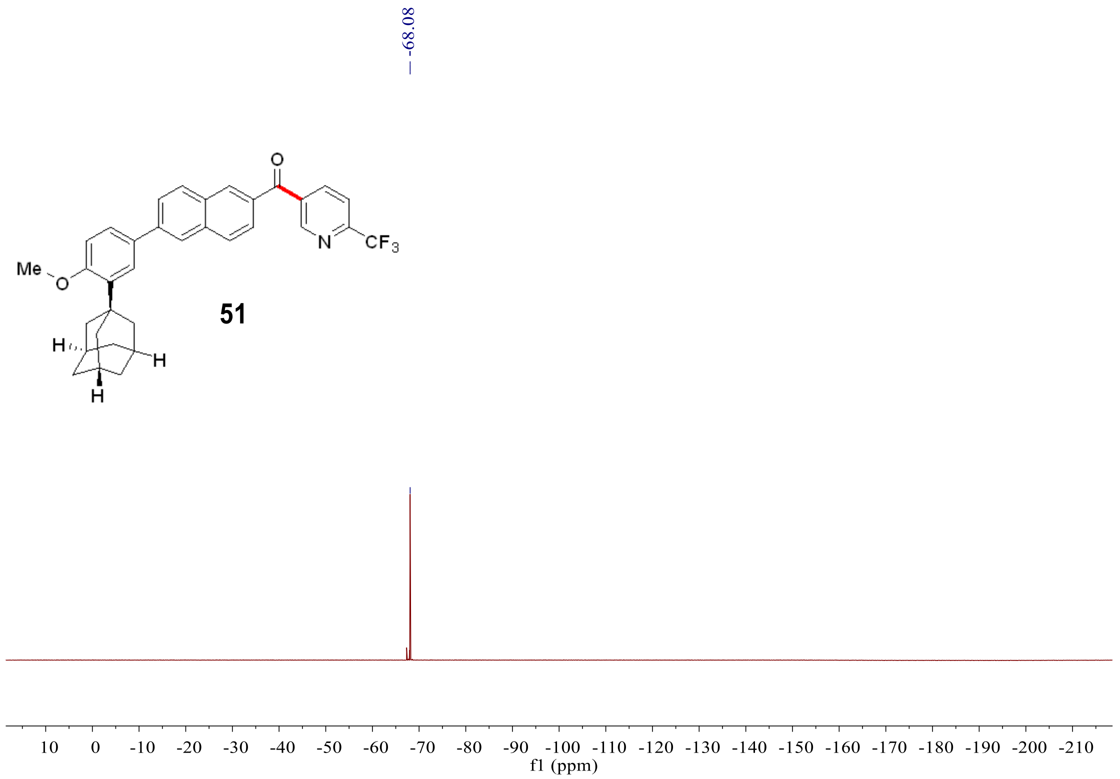

Supplementary Figure 142. ^{19}F NMR spectra for compound **51**

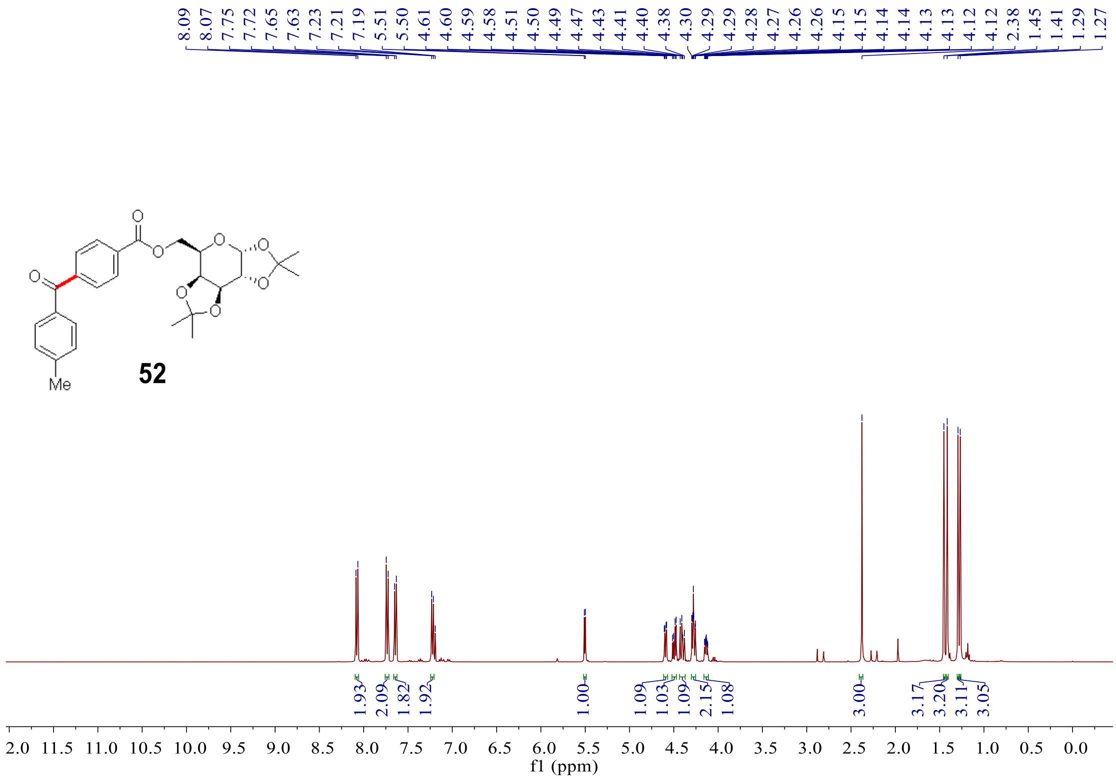

Supplementary Figure 143. ^{1}H NMR spectra for compound **52**

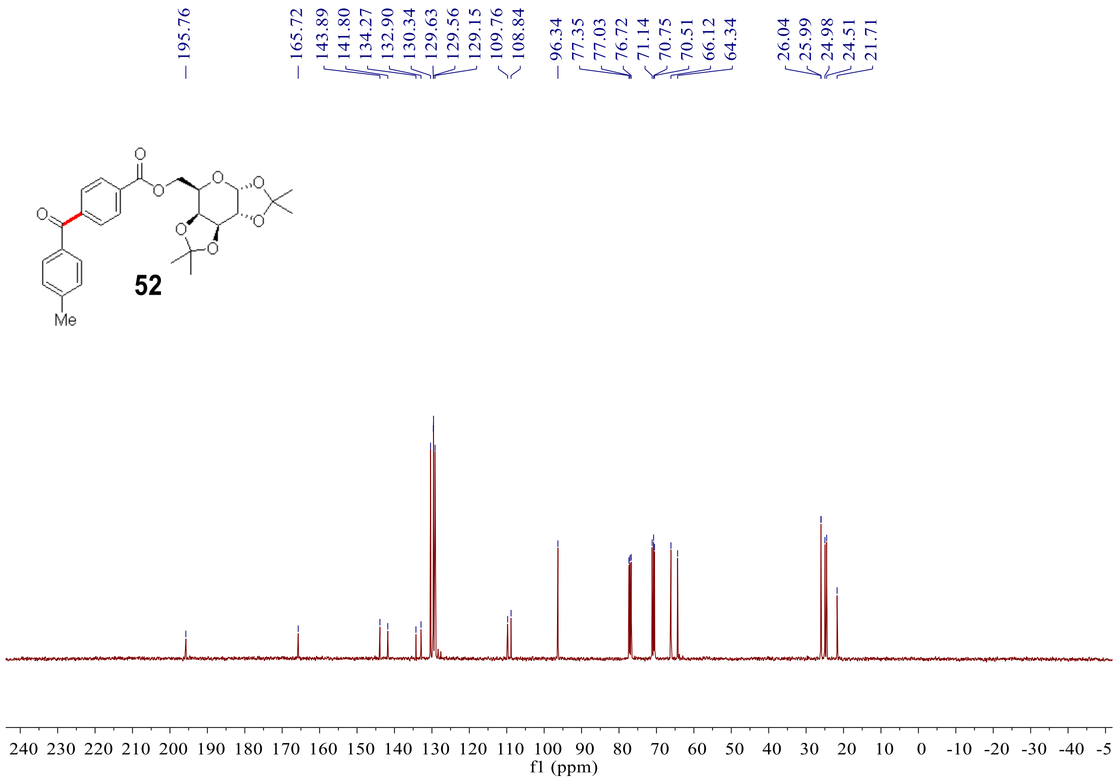

Supplementary Figure 144. ^{13}C NMR spectra for compound **52**

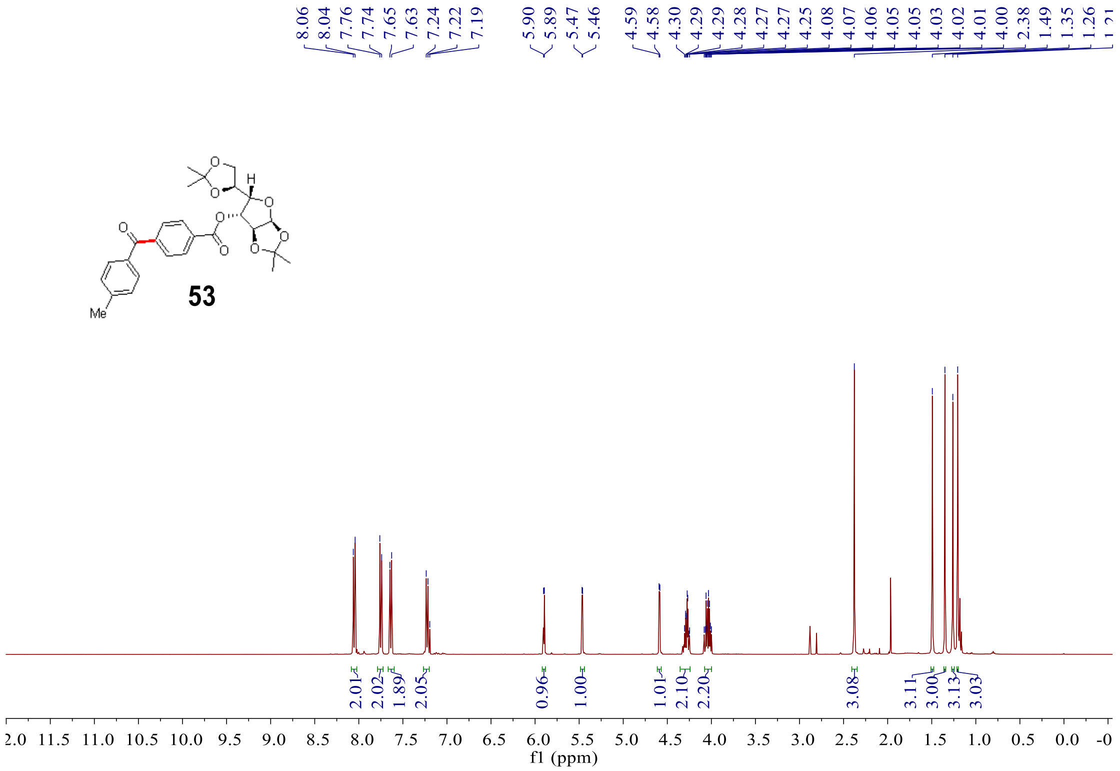

Supplementary Figure 145. ^{1}H NMR spectra for compound **53**

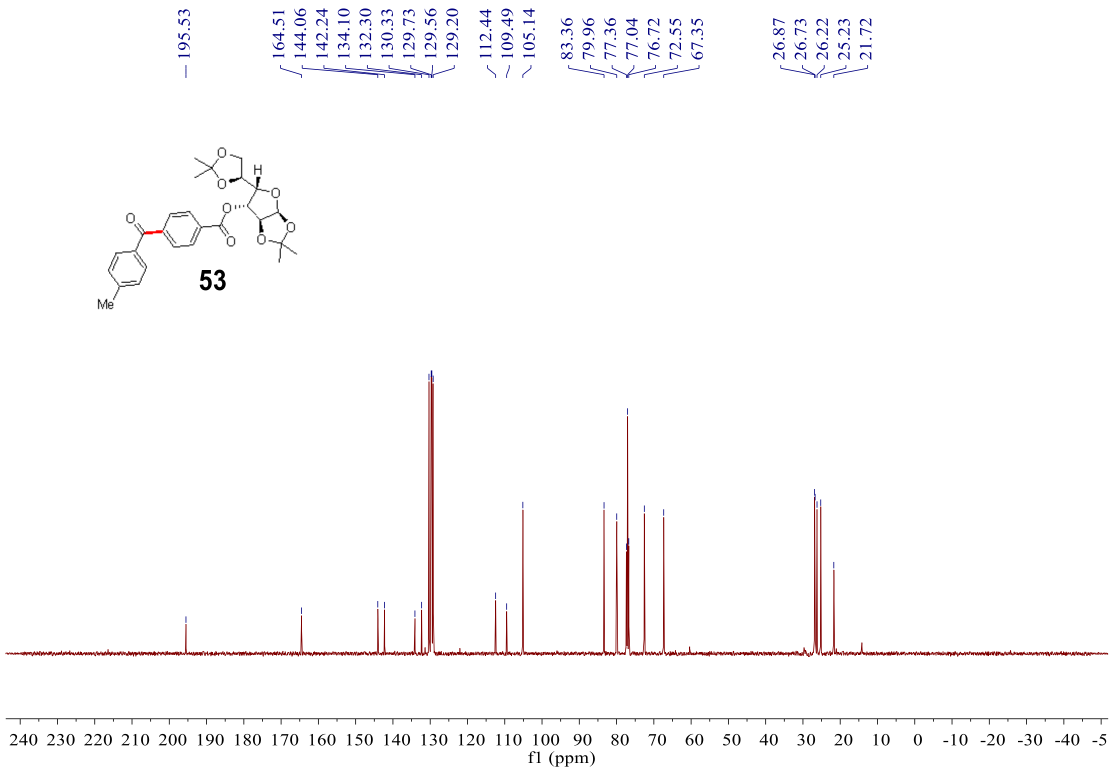

Supplementary Figure 146. ^{13}C NMR spectra for compound **53**

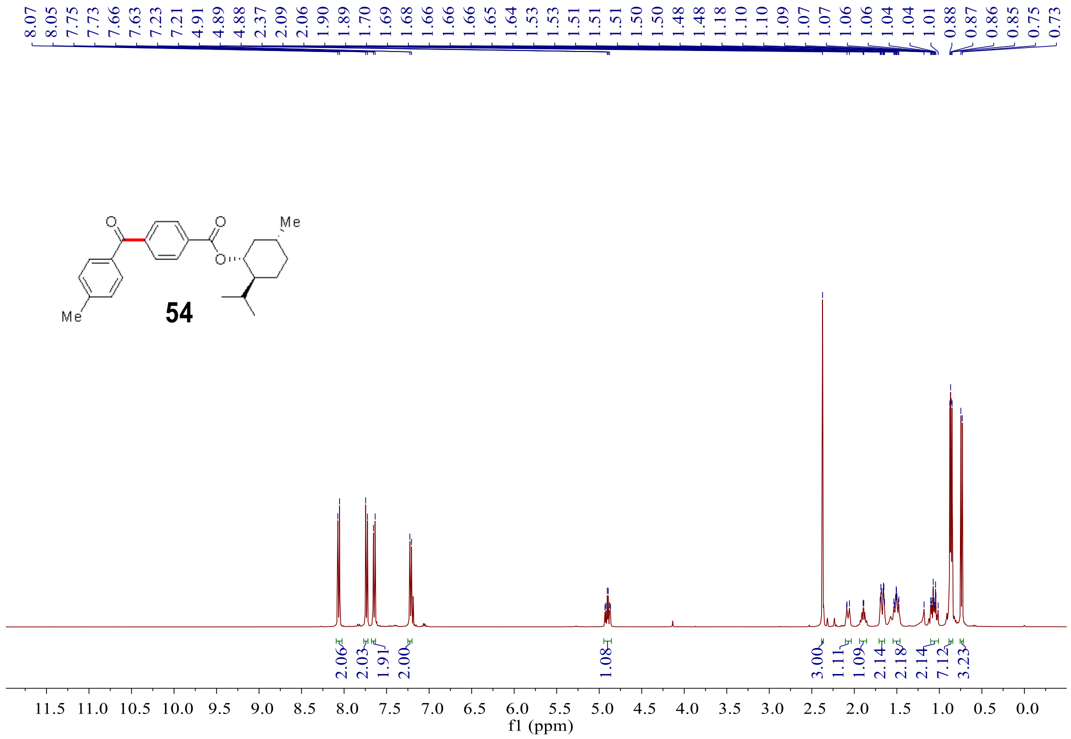

Supplementary Figure 147. ^{1}H NMR spectra for compound **54**

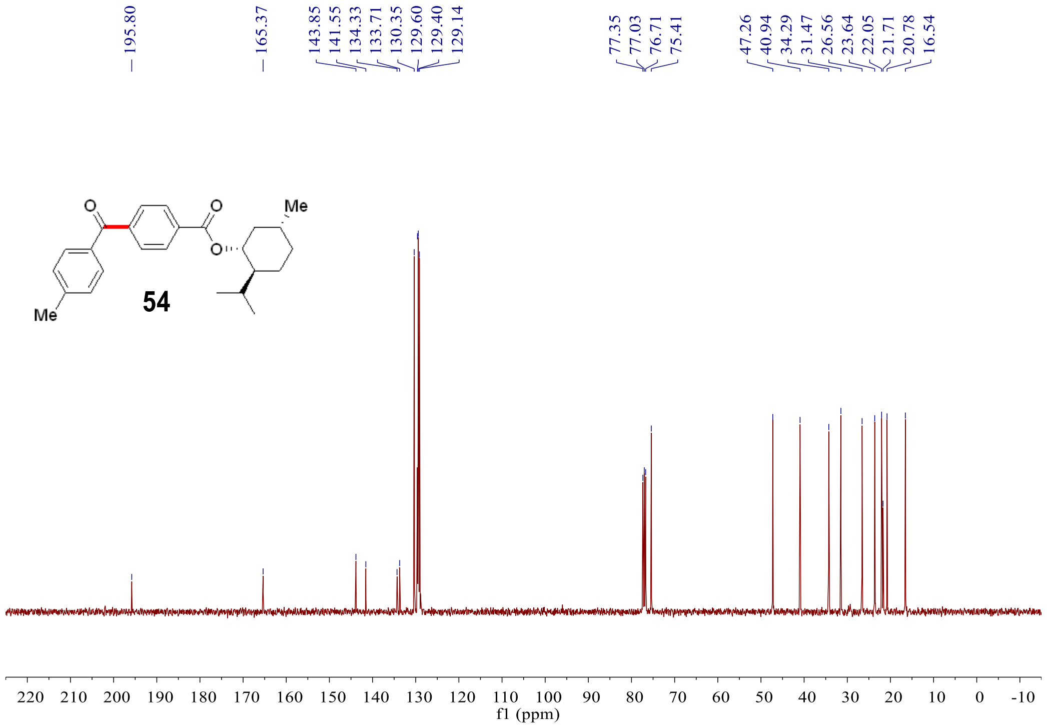

Supplementary Figure 148. ^{13}C NMR spectra for compound **54**

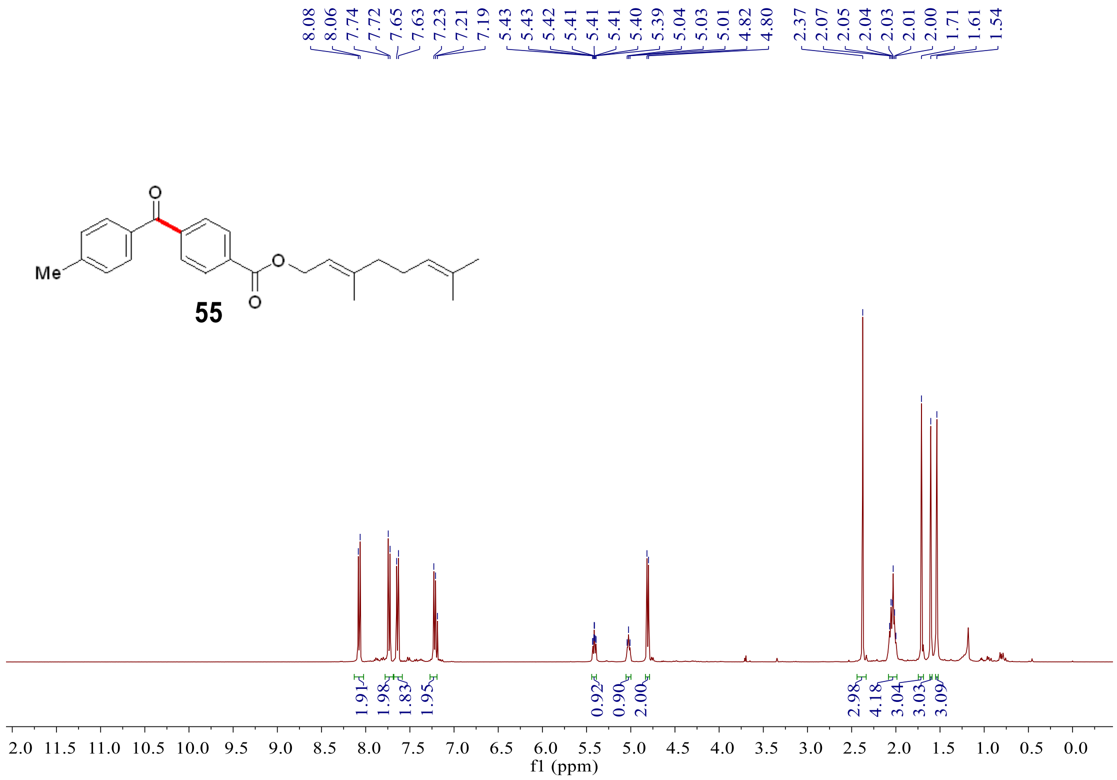

Supplementary Figure 149. ^{1}H NMR spectra for compound **55**

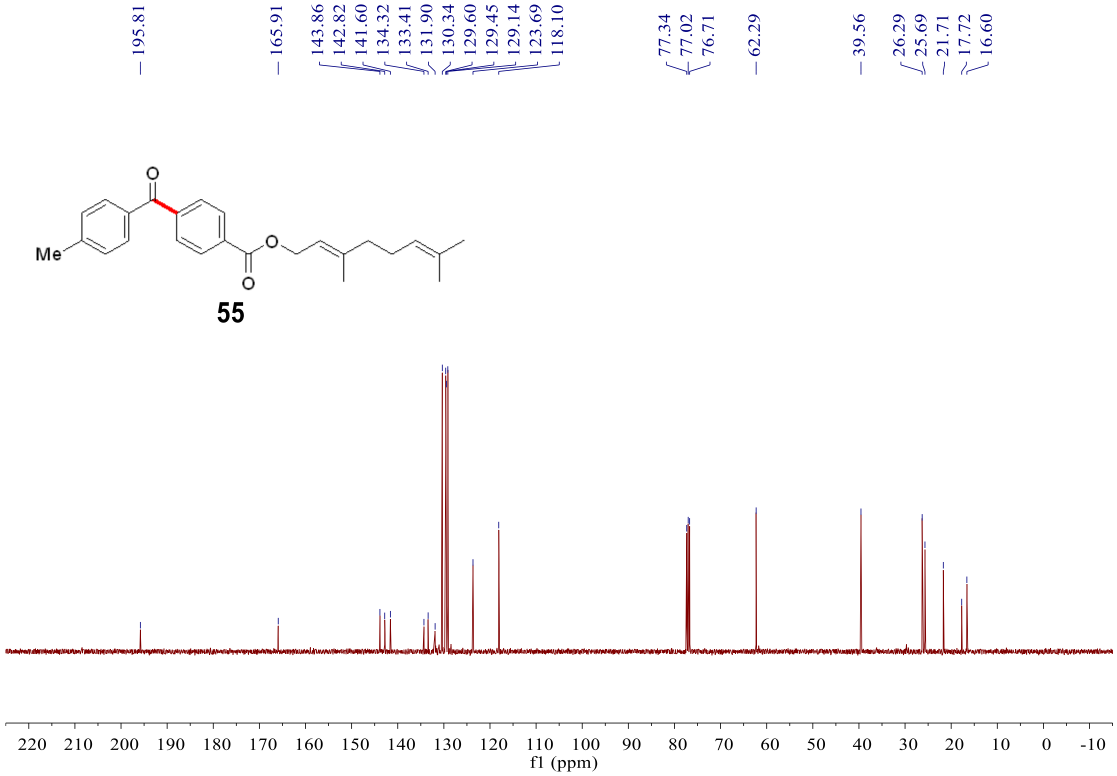

Supplementary Figure 150. ^{13}C NMR spectra for compound **55**

Supplementary References

1. Shields, B. J., Kudisch, B., Scholes, G. D. & Doyle, A. G. Long-Lived Charge-Transfer States of Nickel(II) Aryl Halide Complexes Facilitate Bimolecular Photoinduced Electron Transfer. *J. Am. Chem. Soc.* **140**, 3035-3039 (2018).
2. Si, S., Wang, C., Zhang, N. & Zou, G. Palladium-Catalyzed Room-Temperature Acylative Suzuki Coupling of High-Order Aryl Borons with Carboxylic Acids. *J. Org. Chem*. **81**, 4364-4370 (2016).
3. Gooβsen, L. J., Rudolphi, F., Oppel, C. & Rodríguez, N. Synthesis of Ketones from a-Oxocarboxylates and Aryl Bromides by Cu/Pd-Catalyzed Decarboxylative Cross-Coupling. *Angew. Chem. Int. Ed*. **47**, 3043-3045 (2008).
4. Dohi, S., Moriyama, K., Togo, H. Practical One-pot Preparation of Ketones from Aryl and Alkyl Bromides with Aldehydes and DIH via Grignard Reagents. *Tetrahedron.* **68**, 6557-6564 (2012).
5. Mallari, J. P., Shelat, A., Kosinski, A., Caffrey, C. R., Connelly, M., Zhu, F., McKerrow, J. H. & Guy, R. K. Discovery of Trypanocidal Thiosemicarbazone Inhibitors of Rhodesain and TbcatB. *Bioorg. Med. Chem. Lett.* **18**, 2883-2885 (2008).
6. Tang. S, Bricard. J, Martine. S, Frédéric. Bi. Fukuyama Cross-Coupling Approach to Isoprekinamycin: Discovery of the Highly Active and Bench-Stable Palladium Precatalyst POxAP. *Org. Lett*. **21**, 844−848 (2019).
7. Chu, L., Lipshultz, J. M. & MacMillan, D. W. Merging Photoredox and Nickel Catalysis: The Direct Synthesis of Ketones by the Decarboxylative Arylation of Alpha-Oxo Acids. *Angew. Chem. Int. Ed*. **54**, 7929-7933 (2015).
8. Shen, Y., Gu, Y. & Martin, R. Sp^3 C-H Arylation and Alkylation Enabled by the Synergy of Triplet Excited Ketones and Nickel Catalysts. *J. Am. Chem. Soc.* **140**, 12200-12209 (2018)
